# P(v) intermediate-mediated E1cB elimination for the synthesis of glycals†

**DOI:** 10.1039/d2sc01423h

**Published:** 2022-04-22

**Authors:** Fen Liu, Haiyang Huang, Longgen Sun, Zeen Yan, Xiao Tan, Jing Li, Xinyue Luo, Haixin Ding, Qiang Xiao

**Affiliations:** Key Laboratory of Organic Chemistry, Institute of Organic Chemistry, Jiangxi Science & Technology Normal University Nanchang 330013 Jiangxi Province China huanghaiyang1209@163.com xiaoqiang@tsinghua.org.cn

## Abstract

Glycals are highly versatile and useful building blocks in the chemistry of carbohydrate and natural products. However, the practical synthesis of glycals remains a long-standing and mostly unsolved problem in synthetic chemistry. Herein, we present an unprecedented approach to make a variety of glycals using phosphonium hydrolysis-induced, P(v) intermediate-mediated E1cB elimination. The method provides a highly efficient, practical and scalable strategy for the synthesis of glycals with good generality and excellent yields. Furthermore, the strategy was successfully applied to late-stage modification of complex drug-like molecules. Additionally, the corresponding 1-deuterium-glycals were produced easily by simple ^*t*^BuONa/D_2_O-hydrolysis–elimination. Mechanistic investigations indicated that the oxaphosphorane intermediate-mediated E1cB mechanism is responsible for the elimination reaction.

## Abstract 2

A novel glucosylphosphonium-hydrolysis induced E1cB-elimination provides a highly efficient, practical and scalable method for the synthesis of glycals with good compatibility and excellent yields.

## Graphical abstract

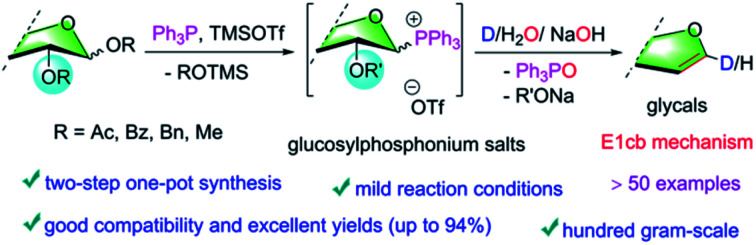

## Introduction

**Fig. 1:**
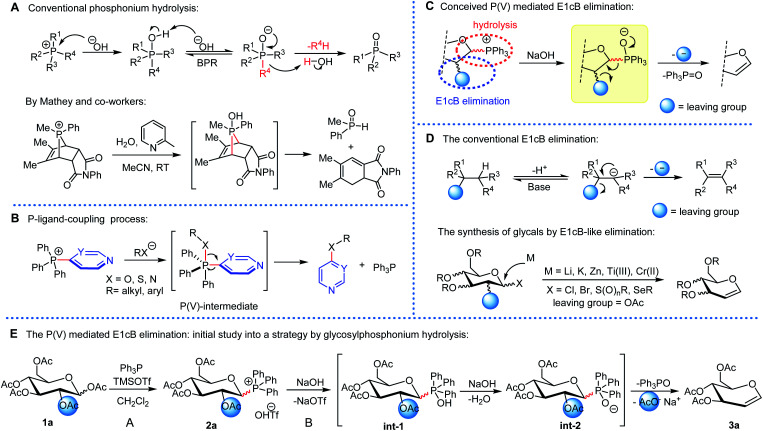
E1cB elimination and phosphonium hydrolysis. (a) The mechanism of phosphonium salt hydrolysis. (b) P-ligand coupling reaction *via* a P(v)-intermediate. (c) Our conceived P(v) mediated E1cB elimination. (d) The E1cB elimination and its application in the synthesis of glycals. (e) Test system for the synthesis of glycals *via* P(v)-mediated E1cB elimination.

As one of the most fundamental and well-established reactions in phosphorus chemistry, the hydrolysis of phosphonium salts is commonly recognized as proceeding *via* oxaphosphorane P(v)-intermediates, which typically results in a phosphine oxide and alkane or arene.^1^ Although an abnormal hydrolysis mode has been observed in specialized 7-phosphanorborenium salts (Fig. 1a),^2^ the application of alkaline hydrolysis of phosphonium salts in practical synthetic chemistry is rare.^3^ Fortunately, conventional P(v)-intermediates have demonstrated promising and unanticipated reactivities in recent years. McNally *et al.* described for the first time the phosphorus ligand-coupling reaction of oxaphosphorane P(v)-intermediates (Fig. 1b),^4^ which was further exploited for contractive C–C bonding reactions of pyridine derivatives.^5^ However, the more reactive deprotonated oxyanionic phosphorane can rapidly release a carbanion-like fragment that is quickly quenched by H_2_O in the hydrolysis system.^6^ Therefore, the use of a carbanion-like intermediate to facilitate further conversions has been a long-standing conundrum.^7^ We hypothesized that because the oxaphosphorane-anion intermediate can provide an adjacent carbanion-like center, an E1cB-type elimination^8^ could be triggered by a suitably leaving group at its β-position to form a double bond.^9^ We are aware that glucosylphosphonium 2a meets the structural requirements and the hydrolysis-mediated elimination could afford the corresponding glycal (Fig. 1c).

Glycals are a class of versatile chiral building blocks that have been extensively used in the synthesis of oligosaccharides and other biologically important molecules.^10,11^ Although considerable effort has been devoted to developing a practical synthetic methodology for glycals, several serious issues persist in the currently available protocols, including the use of a toxic and excessive amount of reducing metal, poor generality, and the complicated preparation of the corresponding appropriate precursor (Fig. S1†).^10,12^ Until now, the reductive metal-induced elimination reaction of peracetylated glycosyl bromide (Fischer–Zach method) has been the dominant approach for synthesizing glycals (Fig. 1d).^10,12*c*,13^ Therefore, the growing demand for diverse glycals necessitates the development of novel and scalable strategies for their preparation. In this article, we demonstrated how to synthesize glycals using an oxaphosphorane-anion-mediated E1cB-like elimination reaction of glucosylphosphonium (Fig. 1e).

## Results and discussion

### Development of an elimination reaction

Fig. 1e depicts the test system. This strategy is based on the alkaline hydrolysis of glucosylphosphonium salt 2a (Scheme S2†), which firsts form a C1-anion-like center followed by an E1cB-like elimination to afford glycal. Indeed, the formation of the C=C π-bond *via* phosphonium hydrolysis has been previously observed, but the reported abnormal process is a result of a high tension of 7-phosphanorbornenium salts (Fig. 1a).^2^ 1,2,3,4,6-Penta-*O*-acetyl-β-d-glucopyranose 1a was chosen as the model substrate since it is commercially accessible (Table S1†). Initially, we developed a facile synthesis of β-d-glucosylphosphonium salt 2a (previously prepared using corresponding glycosyl bromide and PPh_3_ with moderate yield^14^), by treating compound 1a and PPh_3_ with trimethylsilyl trifluoromethanesulfonate (TMSOTf, 1.1 equivalents) in CH_2_Cl_2_ at room temperature (stage A).^15^ Following that, alkaline hydrolysis of phosphonium ion 2a resulted in the formation of glycal 3a*via* P(v) intermediates Int-1 and Int-2 (stage B). The reaction solvents, temperatures, and duration of the reaction were evaluated in detail. To our delight, the desired product 3a could be obtained in two steps under the optimized conditions in 94% total isolated yield (Table S1,† entry 10). The screening results indicated that THF, toluene, or CH_3_CN all inhibit the synthesis of phosphonium 2a primarily because TMSOTf and Ph_3_P can precipitate as a [TMSOTf·Ph_3_P] complex in these solvents. Additionally, we discovered that increasing the temperature (80 °C) and concentration of NaOH (3 M) may deprotect the acetyl groups, resulting in a lower yield. Therefore, the optimal conditions are dichloromethane (CH_2_Cl_2_) as the solvent, aqueous NaOH (1 M) as the base, and room temperature (entry 10) for 2 hours in a mild two-phase hydrolysis system.

### Mechanistic investigation

**Fig. 2:**
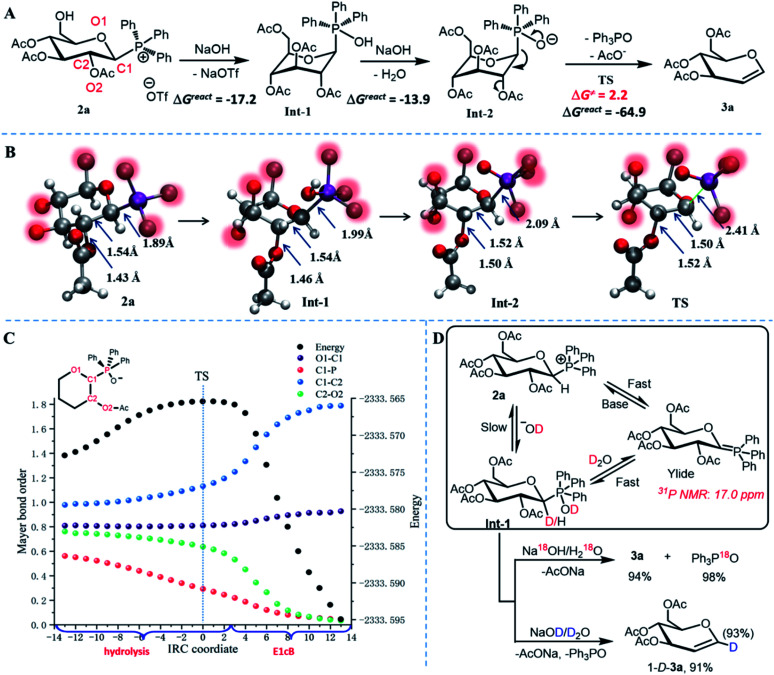
Computational and experimental analysis of P(v) the intermediate-mediated E1cB elimination of glucosyl triphenylphosphoniums. (a) The DFT technique was used to determine the proposed reaction process and activation barrier of E1cB elimination at the m06-2x/6-311+G(d, p)/IEF-PCM_DCM_//b3lyp/6-31+G(d, p) level. The energy is expressed as kcal mol^−1^. (b) Optimized structures for 2a, Int-1, Int-2, and TS demonstrate a stepwise evolution of the main bond lengths and the coordination configurations of the P-group. (c) Calculated variation in the main Mayer bond order and total energy along the IRC coordinate during the elimination process at the B3LYP/6-31G(d,p) level. (d) Hydrolysis conditions for the isotope-labeling experiments are base/H_2_O^18^ or D_2_O. The glycosylphosphonium-ylide species can be detected under dry alkaline conditions.

We performed a series of experimental and computational studies to gain insight into the phosphonium hydrolysis-mediated E1cB-like process. Firstly, we attempted to monitor the hydrolysis process using ^31^P NMR spectroscopy, however, due to its high reactivity, no P(v) intermediates were detected (Fig. S4 and S5†). Therefore, we hypothesized that decomposition of the P(v) intermediate is a rapid process rather than a rate-determining step. Computational studies revealed that the P(v) intermediate Int-2 underwent P–C bond cleavage through the transition states with a very low barrier (Δ*G*^≠^), 2.2 kcal mol^−1^ for TS (β-configurations, Fig. 2a) and 6.6 kcal mol^−1^ for TS′ (α-configurations, Fig. S6†). These findings indicate that the addition of NaOH/H_2_O to glycosylphosphonium is the rate-limiting step. We discovered that the rate of hydrolysis was proportional to the base concentration and depended on the type of solvent, confirming the preceding result. The Δ*G* values indicated that each step of the process was significantly exergonic.

Fig. 2b depicts the computed structures of glycosylphosphonium 2a, P(v) intermediates, and transition state TS. The hydrolysis reaction was characterized by an increase in the length of the P–C1 bond and C2–O bonds, but a decrease in the C1–C2 bond length as the reactions proceeded from 2a to TS. However, the magnitude of the changes in P–C1 bonds was significantly greater than the other two parameters, implying that the carbanion center (C1 of the sugar ring) was formed first with the P(v)-group leaving, followed by the E1cB process (Video S1 and S2†). In the intermediate structure Int-2, the glycosyl as a nucleofugality is located at an apical position with a weaker, longer bond (*d*_P–C(glycosyl)_ = 2.09 Å), whereas anionic oxygen which acts as an electron-donating ligand is placed in an equatorial position, theoretically explaining the observed P–C_(glycosyl)_ cleavage. Additionally, the intrinsic reaction coordinate (IRC; Fig. 2c, Fig. S7 and S8†) demonstrated the formation of the C1=C2 π-bond, the breaking of leaving groups (the P-fragment and anionic OAc group), and a slight variation in the C1–O1 bond. With steady expulsion of the P-group, the Mayer bond order (MBO) values of C1–C2 and C2–O2 bonds evolve slowly at first and then reduce significantly, demonstrating that the oxaphosphorane-anion induces the formation of an anomeric carbanion-like center and then triggers E1cB elimination. Additionally, the relatively low MBO values of C1–O1 and C2–O2 in the intermediate Int-2 imply that electron-withdrawing oxygen disperses the partial anionic electron to enhance glycosyl nucleofugality, resulting in a favored P–C_(glycosyl)_ bond cleavage.

To elucidate the mechanism of the reaction further, we performed isotope-labeling (D and O^18^) experiments (Fig. S4 and S5†). The deuteration reactions demonstrated that the 1,2-elimination of glycosylphosphonium salt to form glycosylphosphonium-ylide species (^31^P NMR = 17.0 ppm, Fig. S5†) was easier and faster than the straightforward nucleophilic attack by a hydroxyl anion in the presence of base/H_2_O, which is consistent with previous reports (Fig. 2d).^16^ This kinetically regulated process results in the synthesis of 1-D-glycal with an extremely high deuterated ratio (93% for 1-D-3a, Scheme S3 and Fig. S2†). Additionally, the findings of the treatment of phosphonium 2a with NaO^18^H/H_2_O^18^ (generated from NaO^*t*^Bu and H_2_O^18^*in situ*) demonstrated that the oxygen in triphenylphosphine oxide is derived from H_2_O^18^ (Scheme S4 and Fig. S3†), confirming that the novel elimination reaction is mediated by a phosphonium-mediated hydrolysis mechanism (Scheme S5†).

### Exploration of the substrate scope

To examine the scope tolerance of P(v)-mediated E1cB elimination, we used a series of commercially available 1-*O*-acetyl sugars as substrates (Table 1). We first examined the general applicability of pyranoses for this transformation under optimized conditions (Table 1a). We were ecstatic to discover that a wide variety of pyranoid glycals containing various protected groups, including acetyl, benzyl, benzoyl, sulfonyl, and ester groups, were obtained in excellent yields (3a–3l). Notably, substrates with BnO- or MeO-groups as adjacent leaving groups were also feasible (3e, 3f), demonstrating that the stable ether linkage does not impair reactivity. In addition, we found that the other configuration at the C2-position, such as penta-*O*-acetyl mannose (3a^c^) and penta-*O*-benzoyl mannose (3ag^c^ in Table 2), had little effect on the rate and yield of the reaction, implying that the E1cB process is suitable for different C-2 configurations as leaving groups. Following that, we extended our reaction to the ever-challenging synthesis of furanoid glycals (Table 1b), which are difficult to synthesize using the Fischer–Zach method.^10,13^ The different protected furanoid glycals can be easily synthesized using this alkali hydrolysis-induced E1cB process (3m–3r). Meanwhile, we discovered that some of the generated furanoid glycals can easily undergo further elimination to form furans (3m, 3p) during workup. This additional elimination, however, can be avoided by using benzyls or methyl as protecting groups (3n, 3o, and 3q).

**Table 1:** Substrate scope of P(v)-mediated E1cB elimination of 1-*O*-acetyl sugars^a^

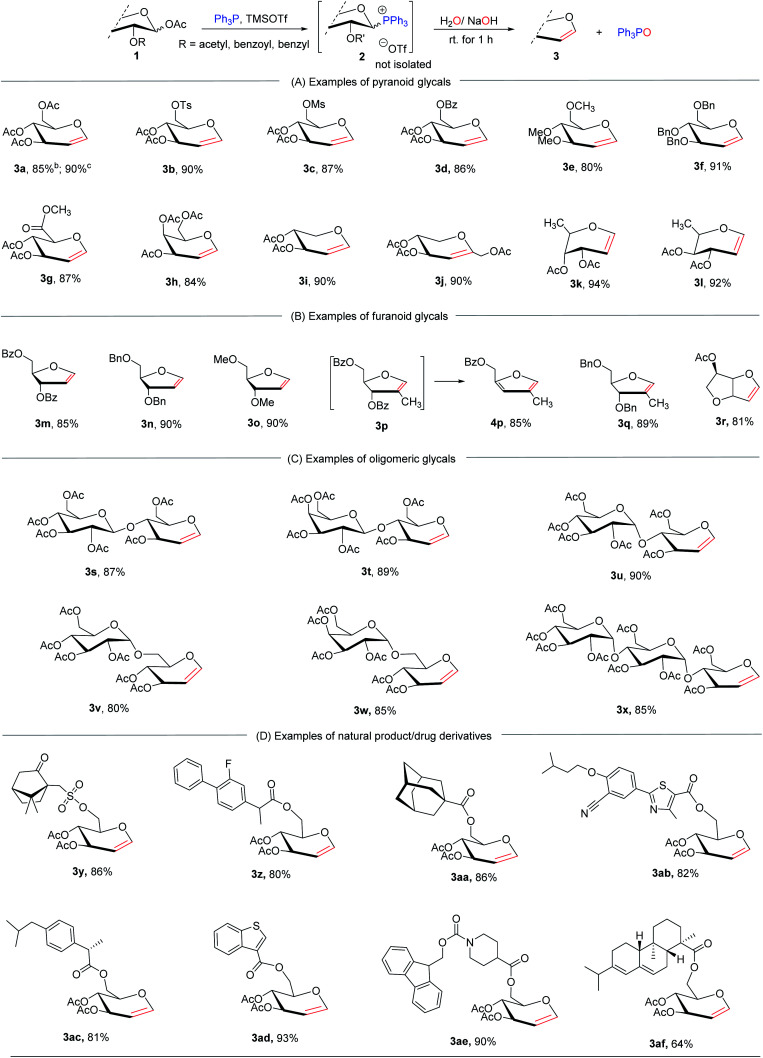

a Reactions conditions: compounds 1 (1 mmol), Ph_3_P (1.1 mmol) and TMSOTf (1.1 mmol) in CH_2_Cl_2_ (15 ml) at rt for 5 h, then the aqueous NaOH/H_2_O (1 M, 0.5 ml) at rt for 2 h; Isolated yields.

b Penta-*O*-acetyl glucose as the substrate.

c Penta-*O*-acetyl mannose as the substrate.

**Table 2:** Substrate scope for the elimination of other common sugar derivatives^a^

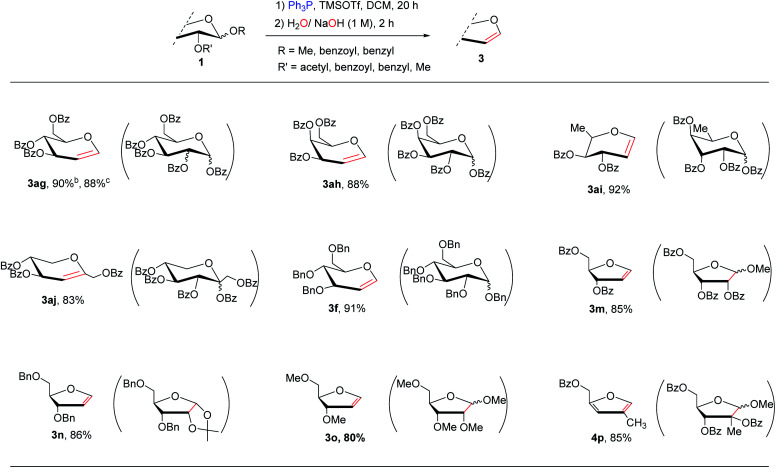

a Reactions conditions: compounds 1 (1 mmol), Ph_3_P (1.1 mmol) and TMSOTf (1.1 mmol) in CH_2_Cl_2_ (15 ml) at 50 °C for 10 h, then the aqueous NaOH/H_2_O (1 M, 0.5 ml) at rt for 2 h; Isolated yields; the starting materials in parentheses.

b Penta-*O*-acetyl glucose as the substrate.

c Penta-*O*-acetyl mannose as the substrate.

To further test the method's efficacy, some selected per-acetylated oligosaccharides, including cellobiose, lactose, maltose, isomaltose, melibiose, and maltotriose, were examined to obtain 3s–3t respectively (Table 1c). Although their yields were not affected, a longer reaction time (24–36 h) was required to prepare the corresponding phosphonium, probably due to their greater steric hindrance. Additionally, the synthetic value of this process was further enhanced by its suitability for natural product- and drug-conjugated sugar derivatives (Table 1d). For example, under slightly adjusted conditions, the 6-*O*-ester of (+)-10-camphorsulfonic acid, flurbiprofen, adamantanecarboxylic acid, febuxostat, (+)-ibuprofen, benzothiophene, Fmoc-isonipecotic acid, and abietic acid smoothly converted to the desired products 3y–3af (phosphoniuming at 80 °C for 12 h and subsequent elimination using 3 M aqueous NaOH for 3 h).

Next, we examine whether this methodology could be applied to other common sugar derivatives with more stable protecting groups on the anomeric carbon, as well as *O*-glycosides (Table 2). The results indicated that under standard conditions, per-benzoyl sugars can react smoothly to form pyranoid and furanoid glycals. Although the acetonide-, 1-benzyloxy- and methoxy-sugar derivatives required a slightly higher reaction temperature (50–60 °C) to complete the corresponding phosphonation, all of the desired products were obtained in good yields (3ag–4p). Thus, our protocol demonstrated a high degree of generality for the synthesis of a variety of glycals at excellent yields under mild metal-free conditions.

### Synthesis of 1-deuterium-glycals

Due to their importance in chemical, environmental, optical, and biological fields, deuterium-labeled compounds have been extensively studied.^17^ Particularly, deuteration using D_2_O as the deuterium source has been proposed as one of the most promising and efficient methods due to its economic and environmental benefits.^18^ However, the reported synthesis of 1-deuterium-glycals is based on the hydrogen–deuterium exchange of alkyl lithium treatment and so requires a multi-step process (at least 7 steps for 1-D-3f, 8 steps for 1-D-3f) and a strict requirement on the protecting group (Scheme S1†).^19^ Therefore, we anticipated that this protocol can be directly used for the regioselective synthesis of a variety of 1-deuterium-glycals *via*^*t*^BuONa/D_2_O-hydrolysis–elimination (D_2_O as the deuterium source). The scope of 1-D-glycal synthesis is shown in Table 3. We investigated the utility of direct one-step synthesis of 1-deuterium-glycals using a variety of different sugar derivatives, including pyranoid and furanoid monosaccharides, disaccharides, trisaccharides, and drug-conjugated sugar derivatives. Therefore, we demonstrated a powerful and facile approach for the synthesis of 1-deuterium-glycals, which may be potentially useful in future chemical and biological applications.

**Table 3:** The synthesis of 1-deuterium-glycals^a^

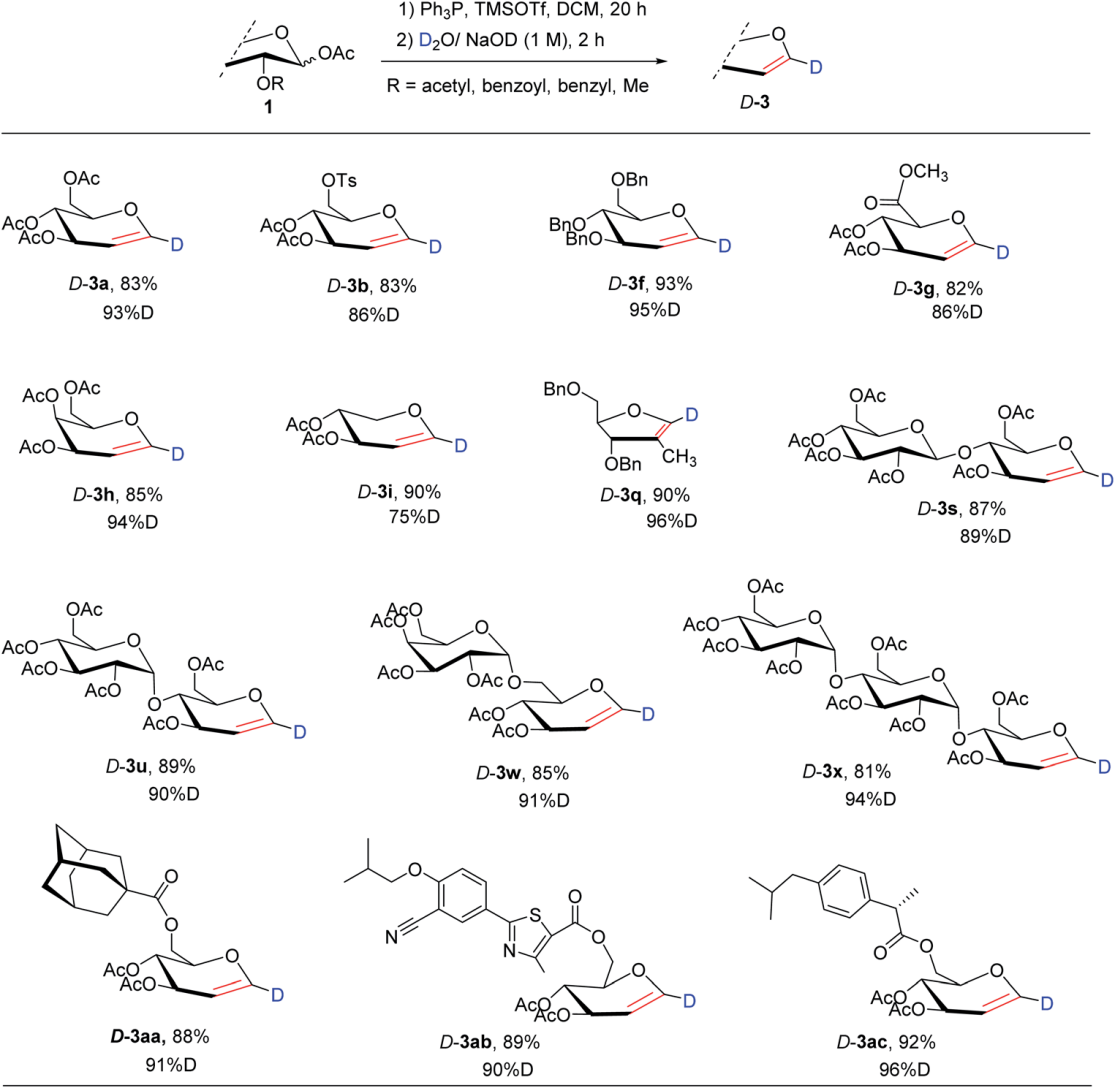

a Reactions conditions: compounds 1 (1 mmol), Ph_3_P (1.1 mmol) and TMSOTf (1.1 mmol) in CH_2_Cl_2_ (15 ml) at rt for 5 h, then the aqueous NaOH/H_2_O (1 M, 0.5 ml) at rt for 2 h; isolated yields; the deuterated ratios given by ^1^H NMR.

### Scale-up reaction

To further evaluate the practicability of the currently developed method, 200 grams per-*O*-acetyl glucose 1a was used as a model substrate (Fig. S9†). Although the yield was unaffected by the air atmosphere, somewhat longer reaction times for phosphoniuming (10 h) and hydrolysis–elimination (5 h) were required. Fortunately, pure glucosylphosphonium 2a (370 g, 94% yield) can be obtained by simple filtration and crystallization. The glycal 3a (131 g) was achieved in 94% yield after hydrolysis of the obtained phosphonium, as long as the insoluble triphenyl-phosphinoxide in diethyl ether was removed. We anticipate that this method developed into a commercially viable method for the synthesis of glycals.

## Conclusions

In summary, we present a novel oxyanion phosphorane-mediated E1cB-like elimination method for the synthesis of glycals and 1-deuterium-glycals. This method overcomes the major limitations of conventional approaches by virtue of the metal-free, mild base-induced conditions and compatibility with pyranoid and furanoid derivatives, particularly complex oligosaccharides and drug-like molecules. Additionally, this method can be easily scaled up to a hundred-gram-scale, allowing for large-scale preparation. This method is advantageous in medicinal chemistry due to its simple protocols, readily available reagents, broad applicability, and scale-up synthesis. Additionally, the currently developed hydrolysis-mediated elimination method enables a new application of anionic phosphorane in synthetic chemistry and encourages chemists to explore further reactivity of phosphoranes.

## Data availability

All experimental and characterization data are available in the ESI.† Crystallographic data for compound 2a′ have been deposited in the Cambridge Crystallographic Data Centre under the accession number CCDC: 2142375.

## Author contributions

H. H. and Q. X. directed the project and co-wrote the manuscript; H. H. conceived the work, designed the experiments, conducted the DFT calculation and analysed the data; F. L., L. S., Z. Y., X. T., J. L., X. L., and H. D. performed the experiments and collected experimental data; all authors have given approval regarding the final version of the manuscript.

## Conflicts of interest

There are no conflicts to declare.

## Supplementary Material

SC-013-D2SC01423H-s001

SC-013-D2SC01423H-s002

SC-013-D2SC01423H-s003

SC-013-D2SC01423H-s004

SC-013-D2SC01423H-s005
