# Supplementary material for: P(v) intermediate-mediated E1cB elimination for the synthesis of glycals
Source: Chem Sci. 2022 Apr 22;13(19):5588–96. doi: 10.1039/d2sc01423h (PMC9116453; doi:10.1039/d2sc01423h)
Supplement: SC-013-D2SC01423H-s001 [file SC-013-D2SC01423H-s001.pdf]

Electronic Supplementary Material (ESI) for Chemical Science.
This journal is © The Royal Society of Chemistry 2022

2a

Zero-point correction = 0.610194 (Hartree/Particle)

Thermal correction to Energy = 0.650375

Thermal correction to Enthalpy = 0.651319

Thermal correction to Gibbs Free Energy = 0.536874

Sum of electronic and zero-point Energies = -2257.298705

Sum of electronic and thermal Energies = -2257.258524

Sum of electronic and thermal Enthalpies = -2257.257580

Sum of electronic and thermal Free Energies = -2257.372025

P	1.77113	-0.25396	0.05646
O	-0.72128	-2.45085	-0.72501
O	-3.99214	1.00528	-0.71722
O	-3.3357	-1.64819	0.13948
O	-0.36436	1.15873	-0.17673
O	-1.00578	3.97156	-0.77339
O	-0.35312	-3.44885	1.28042
O	-4.82287	1.74132	1.26444
O	-4.03433	-2.52608	-1.83937
C	-2.41419	-0.77307	-0.52089
H	-2.52931	-0.88956	-1.60367
C	1.72116	0.12043	1.82421
C	-0.99181	-1.18593	-0.10579
H	-0.94015	-1.28633	0.98064
C	0.01218	-0.13273	-0.61451
H	0.07248	-0.19307	-1.71351
C	-0.50324	-3.53956	0.08197
C	2.5108	-1.87703	-0.29065
O	0.40454	4.23115	0.99044
C	-2.73923	0.66337	-0.10704
H	-2.8327	0.73689	0.98052
C	-1.65498	1.62801	-0.61572
H	-1.67882	1.66129	-1.71538
C	2.78435	0.95832	-0.84662
C	2.51733	-2.33564	-1.62156
H	2.02408	-1.77129	-2.40732
C	-4.98704	1.51001	0.08916
C	-0.48418	-4.80592	-0.72494
H	-1.46537	-4.96258	-1.18415
H	-0.23739	-5.64605	-0.0769
H	0.24409	-4.72616	-1.53633
C	-1.8201	3.03064	-0.04946
H	-2.85177	3.36144	-0.17833
H	-1.55382	3.04374	1.00939
C	5.01545	1.74751	-1.38076

H	6.09063	1.67877	-1.25033
C	2.23787	1.92275	-1.70741
H	1.16546	2.02131	-1.81919
C	-4.12001	-2.47233	-0.63496
C	2.10677	1.39878	2.26209
H	2.4551	2.14642	1.55909
C	4.18002	0.87265	-0.6886
H	4.61797	0.12361	-0.03575
C	3.17572	-3.51974	-1.94619
H	3.1806	-3.86681	-2.97467
C	1.22383	-0.8247	2.7441
H	0.89557	-1.8078	2.42118
C	0.04365	4.55282	-0.12061
C	2.01449	1.7213	3.61621
H	2.30459	2.71264	3.94806
C	4.46929	2.69861	-2.24808
H	5.12205	3.37129	-2.79563
C	3.83661	-4.25051	-0.95196
H	4.35318	-5.1701	-1.20892
C	1.53781	0.78036	4.53151
H	1.46813	1.03609	5.5843
C	1.14052	-0.48755	4.09475
H	0.76021	-1.21674	4.80301
C	3.08517	2.78164	-2.41126
H	2.65921	3.5179	-3.08538
C	3.18194	-2.60699	0.70225
H	3.20133	-2.25898	1.72839
C	-6.24688	1.73274	-0.70005
H	-6.06628	2.46848	-1.49016
H	-7.03099	2.09132	-0.03425
H	-6.55776	0.80427	-1.18754
C	0.64762	5.65401	-0.95173
H	0.58664	5.4327	-2.01907
H	1.68022	5.82064	-0.64401
H	0.08034	6.57399	-0.77106
C	3.83902	-3.79387	0.36693
H	4.35455	-4.35528	1.13945
C	-5.06267	-3.26276	0.23044
H	-4.50566	-3.81516	0.99287
H	-5.63499	-3.94999	-0.39166
H	-5.74157	-2.58396	0.75594

2a′

Zero-point correction = 0.610617 (Hartree/Particle)

Thermal correction to Energy = 0.650778
Thermal correction to Enthalpy = 0.651722
Thermal correction to Gibbs Free Energy = 0.537421
Sum of electronic and zero-point Energies = -2257.294272
Sum of electronic and thermal Energies = -2257.254112
Sum of electronic and thermal Enthalpies = -2257.253168
Sum of electronic and thermal Free Energies = -2257.367468

O	1.020197	-2.23714	-0.62506
O	3.164179	1.416578	-1.10656
O	3.481791	-1.07603	0.126621
O	0.268091	1.38014	-0.87185
O	0.187943	4.072049	0.52844
O	1.003682	-2.61605	-2.85998
O	5.07274	1.941357	0.008942
O	3.188916	-2.03379	2.165027
C	2.241018	-0.37225	0.253137
H	1.823544	-0.57953	1.240015
C	1.30484	-0.85189	-0.85552
H	1.815992	-0.7513	-1.8162
C	0.010116	-9.3E-05	-0.97511
C	0.980611	-3.05577	-1.73438
O	-0.63721	4.605804	-1.51951
C	2.494684	1.130376	0.131564
H	3.123059	1.464112	0.962188
C	1.153948	1.86197	0.166271
H	0.698261	1.687636	1.149904
C	4.489398	1.784706	-1.03837
C	0.912882	-4.50156	-1.3356
H	1.826036	-4.7791	-0.79977
H	0.807814	-5.11756	-2.22784
H	0.070895	-4.67017	-0.65878
C	1.301582	3.363514	-0.04618
H	1.383474	3.591208	-1.10991
C	3.872893	-1.86481	1.179027
C	-0.6791	4.70891	-0.31314
C	5.06505	1.954555	-2.416
H	6.060687	2.390301	-2.34046
H	4.417226	2.587106	-3.02878
H	5.12915	0.97759	-2.90636
C	-1.65195	5.554356	0.465148
H	-2.02834	5.017878	1.339443
H	-1.13414	6.449228	0.826951
C	5.237939	-2.44258	0.935284
H	5.442235	-3.21743	1.673507

H	5.983104	-1.64542	1.031071
H	5.315953	-2.84463	-0.07799
P	-1.51122	-0.47665	0.086907
H	-2.47308	5.858109	-0.18353
C	-2.10647	-2.14472	-0.34617
C	-2.41171	-3.09785	0.637505
C	-2.36837	-2.42862	-1.69906
C	-2.96444	-4.32683	0.26698
H	-2.23361	-2.88939	1.686241
C	-2.91247	-3.66054	-2.05977
H	-2.16415	-1.69693	-2.47491
C	-3.21272	-4.61029	-1.07773
H	-3.20464	-5.05713	1.033295
H	-3.10731	-3.87333	-3.10596
H	-3.64471	-5.56506	-1.36095
C	-2.86512	0.648486	-0.38854
C	-4.15273	0.314648	0.077736
C	-2.69369	1.766704	-1.22166
C	-5.24902	1.097215	-0.27739
H	-4.30553	-0.55757	0.706467
C	-3.80442	2.536159	-1.58108
H	-1.71512	2.057173	-1.58095
C	-5.07659	2.207663	-1.11119
H	-6.23663	0.833668	0.087544
H	-3.66029	3.394759	-2.22906
H	-5.93389	2.810227	-1.39511
C	-1.18731	-0.35265	1.869122
C	-0.30958	-1.26063	2.493436
C	-1.78391	0.6737	2.622187
C	-0.03357	-1.13502	3.854569
H	0.16729	-2.05499	1.92862
C	-1.50288	0.787153	3.985023
H	-2.46071	1.378925	2.152674
C	-0.62982	-0.11329	4.600699
H	0.655181	-1.83021	4.32312
H	-1.96631	1.580696	4.562525
H	-0.4119	-0.01876	5.659953
H	-0.37626	-0.14705	-1.99029
H	2.187839	3.719004	0.486101

ylide

Zero-point correction = 0.596741 (Hartree/Particle)

Thermal correction to Energy = 0.636898

Thermal correction to Enthalpy = 0.637842

Thermal correction to Gibbs Free Energy = 0.523229
Sum of electronic and zero-point Energies = -2256.821474
Sum of electronic and thermal Energies = -2256.781317
Sum of electronic and thermal Enthalpies = -2256.780373
Sum of electronic and thermal Free Energies = -2256.894986

P	1.76559	0.31699	-0.01878
O	0.20859	-2.55953	-0.19194
O	-3.94129	-0.25376	-0.20478
O	-2.56273	-2.70088	0.51133
O	-0.63438	0.8566	0.90892
O	-2.1999	3.4482	0.67874
O	1.01877	-3.4662	1.72902
O	-5.22145	-0.07151	1.66233
O	-2.89928	-3.52663	-1.583
C	-1.91802	-1.50216	0.01893
H	-1.9345	-1.51291	-1.07239
C	2.96334	0.31713	1.41442
C	-0.45968	-1.48282	0.50625
H	-0.4383	-1.70721	1.58017
C	0.11121	-0.12051	0.16491
C	0.86639	-3.50214	0.52349
C	2.54416	-0.75438	-1.28548
O	-3.95168	3.52087	-0.76066
C	-2.71373	-0.31073	0.55513
H	-2.9543	-0.47219	1.60996
C	-1.95103	1.02019	0.38066
H	-1.89833	1.26295	-0.68915
C	1.83686	2.05944	-0.59577
C	1.8236	-1.06862	-2.45034
H	0.8034	-0.71485	-2.55331
C	-5.12317	-0.12839	0.45362
C	1.39464	-4.58428	-0.38429
H	0.58236	-5.00304	-0.98536
H	1.86487	-5.36339	0.21542
H	2.12558	-4.15638	-1.07736
C	-2.62657	2.14973	1.15129
H	-3.71125	2.08584	1.0791
H	-2.32064	2.10713	2.19847
C	2.35459	3.71214	-2.29928
H	2.71528	3.94658	-3.29657
C	1.43981	3.09275	0.26878
H	1.07906	2.86114	1.26441
C	-3.00848	-3.6248	-0.37914
C	4.02615	1.23042	1.52

H	4.18824	1.97207	0.74381
C	2.29464	2.37944	-1.88247
H	2.61099	1.59534	-2.56193
C	2.40946	-1.84436	-3.45139
H	1.8407	-2.08594	-4.34446
C	2.76794	-0.62401	2.43758
H	1.95404	-1.34042	2.37735
C	-2.95519	4.02825	-0.28535
C	4.88113	1.19641	2.62432
H	5.69854	1.90868	2.69514
C	1.95784	4.73562	-1.43568
H	2.00995	5.77166	-1.75845
C	3.71947	-2.31164	-3.30301
H	4.17439	-2.91427	-4.08406
C	4.68238	0.25214	3.63621
H	5.3452	0.22921	4.49659
C	3.62642	-0.65766	3.53996
H	3.46441	-1.39356	4.3221
C	1.49837	4.42196	-0.15298
H	1.18687	5.21128	0.52486
C	3.85725	-1.22757	-1.141
H	4.42412	-0.99938	-0.24511
C	-6.26589	-0.03757	-0.52373
H	-6.23026	0.9412	-1.0143
H	-7.2102	-0.14224	0.01044
H	-6.17348	-0.80086	-1.3005
C	-2.39294	5.37161	-0.67786
H	-2.31592	6.01879	0.20099
H	-3.03958	5.83091	-1.4252
H	-1.38436	5.2443	-1.08276
C	4.44038	-2.00412	-2.14665
H	5.45578	-2.36881	-2.02198
C	-3.65943	-4.77817	0.34529
H	-2.96381	-5.20908	1.07142
H	-3.96698	-5.53394	-0.37717
H	-4.5303	-4.42048	0.9035

Int-1

Zero-point correction = 0.624823 (Hartree/Particle)

Thermal correction to Energy = 0.666194

Thermal correction to Enthalpy = 0.667138

Thermal correction to Gibbs Free Energy = 0.551181

Sum of electronic and zero-point Energies = -2333.239108

Sum of electronic and thermal Energies = -2333.197736

Sum of electronic and thermal Enthalpies = -2333.196792
Sum of electronic and thermal Free Energies = -2333.312749

P	1.7742	-0.39754	-0.35485
O	-0.95943	-2.40523	-1.41287
O	-4.21569	0.7826	-0.29748
O	-3.34525	-1.94606	0.11159
O	-0.56439	1.0256	-0.1664
O	-1.39101	3.83662	-0.5214
O	0.27352	-3.67857	-0.00086
O	-4.89262	0.9931	1.86255
O	-4.5307	-2.44413	-1.76631
C	-2.56926	-0.92953	-0.55872
H	-2.86847	-0.90197	-1.61041
C	3.42702	-1.32825	0.06533
C	-1.09005	-1.32854	-0.45897
H	-0.87584	-1.70258	0.54587
C	-0.14642	-0.1608	-0.83067
H	-0.178	-0.01199	-1.91671
C	-0.25335	-3.50594	-1.08323
C	2.4105	1.22519	-0.98018
O	-0.10969	4.41142	1.26491
C	-2.87238	0.41624	0.08825
H	-2.81384	0.35421	1.17783
C	-1.88718	1.4671	-0.44667
H	-2.02961	1.58093	-1.53352
C	1.39845	-0.20991	1.51921
C	2.3843	1.43116	-2.36952
H	1.97545	0.66124	-3.01483
C	-5.14018	1.01573	0.67668
C	-0.20809	-4.47453	-2.23676
H	-1.20201	-4.60357	-2.67296
H	0.18853	-5.43116	-1.89652
H	0.44246	-4.06269	-3.01534
C	-2.07758	2.81579	0.23011
H	-3.13448	3.09072	0.23084
H	-1.69941	2.79035	1.25333
C	1.12446	-1.32707	3.6767
H	1.14872	-2.24484	4.25768
C	1.03335	0.99833	2.14301
H	0.94616	1.91425	1.57544
C	-4.28031	-2.63771	-0.59643
C	3.49562	-2.73425	0.03285
H	2.61559	-3.31761	-0.21961
C	1.43082	-1.37013	2.31346

H	1.68702	-2.32772	1.87619
C	2.89816	2.60303	-2.92975
H	2.87017	2.73865	-4.00727
C	4.58359	-0.62849	0.44195
H	4.58352	0.45414	0.48097
C	-0.43038	4.56399	0.10432
C	4.67503	-3.40979	0.3635
H	4.69152	-4.49622	0.34158
C	0.79856	-0.11483	4.28625
H	0.57419	-0.07579	5.34852
C	3.45451	3.5884	-2.11087
H	3.8647	4.49558	-2.54578
C	5.81941	-2.69642	0.72376
H	6.73797	-3.21906	0.97476
C	5.76731	-1.30159	0.75909
H	6.64937	-0.72912	1.03339
C	0.75769	1.0471	3.51251
H	0.49822	2.00052	3.96451
C	2.96381	2.22602	-0.16663
H	3.00116	2.10364	0.90872
C	-6.48635	1.30861	0.06463
H	-6.41932	2.18746	-0.58411
H	-7.21352	1.48566	0.85659
H	-6.80343	0.46817	-0.56002
C	0.17586	5.56805	-0.84238
H	0.82952	5.04347	-1.54671
H	0.7645	6.28975	-0.27598
H	-0.60171	6.07501	-1.41924
C	3.48417	3.39505	-0.72798
H	3.91198	4.15273	-0.07779
C	-4.94563	-3.66631	0.28361
H	-5.72575	-4.17674	-0.28067
H	-5.37116	-3.18607	1.16965
H	-4.20319	-4.39082	0.63259
O	1.71113	-1.36644	-1.80386
H	2.56641	-1.79089	-1.96613

Int-1-b

Zero-point correction = 0.625238 (Hartree/Particle)

Thermal correction to Energy = 0.666267

Thermal correction to Enthalpy = 0.667211

Thermal correction to Gibbs Free Energy = 0.552520

Sum of electronic and zero-point Energies = -2333.222387

Sum of electronic and thermal Energies = -2333.181358

Sum of electronic and thermal Enthalpies = -2333.180414

Sum of electronic and thermal Free Energies = -2333.295105

O	0.70673	-0.08023	-1.30655
C	-0.12998	0.80746	-0.58242
C	2.72491	0.29542	0.09702
C	1.86335	-0.68313	-0.7154
C	1.50592	-1.96047	0.06617
C	0.66804	1.82484	0.24672
C	1.89967	1.24735	0.96922
O	3.51755	1.12676	-0.78845
O	5.17607	-0.42041	-0.78502
C	4.72843	0.64166	-1.16639
C	5.41739	1.58767	-2.11668
O	1.13689	2.81133	-0.72555
O	1.20301	4.41872	0.87027
C	1.33905	4.07356	-0.28897
C	1.73422	4.98418	-1.4241
O	2.7126	-2.6065	0.52494
O	2.90994	-3.70977	-1.44593
C	3.3441	-3.43989	-0.34514
C	4.63968	-3.94266	0.23436
H	3.40193	-0.27585	0.73378
H	2.45008	-0.99875	-1.5817
H	0.93767	-1.75182	0.97005
H	0.95354	-2.62959	-0.59251
H	0.02312	2.32276	0.96227
H	4.85804	1.63038	-3.05687
H	5.44247	2.59877	-1.70085
H	6.42961	1.23323	-2.30952
H	2.06071	5.94385	-1.02375
H	2.52625	4.5267	-2.02244
H	0.87313	5.13855	-2.08284
H	4.9744	-4.81514	-0.32716
H	5.38625	-3.14615	0.14307
H	4.5293	-4.18285	1.29445
H	-0.60365	1.40284	-1.37062
P	-1.80579	0.01298	0.12905
C	-2.19802	-1.4422	1.24981
C	-3.3164	-2.25555	1.00104
C	-1.42381	-1.70155	2.39342
C	-3.65927	-3.28908	1.87538
H	-3.92879	-2.09529	0.1224
C	-1.74783	-2.75956	3.24786
H	-0.57909	-1.07113	2.64194

C	-2.86927	-3.55223	2.99733
H	-4.53958	-3.89196	1.67116
H	-1.12486	-2.94424	4.11805
H	-3.12919	-4.36401	3.67077
C	-3.29852	1.07639	-0.26525
C	-4.45288	0.54463	-0.86294
C	-3.29086	2.44542	0.05384
C	-5.57001	1.34538	-1.10997
H	-4.485	-0.49674	-1.15858
C	-4.39327	3.25645	-0.22891
H	-2.42559	2.87465	0.54379
C	-5.54195	2.70738	-0.80186
H	-6.4564	0.90341	-1.55593
H	-4.35524	4.31449	0.01455
H	-6.40608	3.33304	-1.00621
C	-1.90959	-1.02145	-1.53322
C	-2.13839	-0.35995	-2.75419
C	-1.62785	-2.39434	-1.59998
C	-2.10269	-1.03392	-3.97642
H	-2.36227	0.7037	-2.76509
C	-1.58884	-3.08071	-2.81895
H	-1.43411	-2.95575	-0.69247
C	-1.82738	-2.40334	-4.01467
H	-2.28987	-0.48785	-4.89735
H	-1.36334	-4.14362	-2.82826
H	-1.79595	-2.93238	-4.96291
O	-1.47195	0.99435	1.60067
H	-2.16384	0.8919	2.26912
H	2.53869	2.07166	1.29254
O	1.48355	0.45119	2.0911
C	1.27289	0.96337	3.34368
O	0.82848	0.21082	4.18022
C	1.65506	2.39799	3.61774
H	2.74345	2.51607	3.55875
H	1.20953	3.09935	2.90764
H	1.33321	2.63827	4.63046

Int-2-b

Zero-point correction = 0.610015 (Hartree/Particle)

Thermal correction to Energy = 0.651582

Thermal correction to Enthalpy = 0.652526

Thermal correction to Gibbs Free Energy = 0.535058

Sum of electronic and zero-point Energies = -2332.774018

Sum of electronic and thermal Energies = -2332.732450

Sum of electronic and thermal Enthalpies = -2332.731506

Sum of electronic and thermal Free Energies = -2332.848974

P	1.93322	-0.59382	-0.32232
O	-0.86702	-2.40246	-1.46517
O	-4.18464	0.73066	-0.28856
O	-3.35054	-2.04353	0.00655
O	-0.5468	0.91324	0.0616
O	-1.32194	3.71685	-0.23241
O	-0.27223	-4.0396	-0.00349
O	-5.07442	0.60116	1.80154
O	-4.55673	-2.38139	-1.89465
C	-2.53325	-0.9901	-0.57206
H	-2.79402	-0.89576	-1.6295
C	1.55131	-0.77601	1.49896
C	-1.05858	-1.40106	-0.44336
H	-0.89172	-1.85195	0.53905
C	-0.08878	-0.22941	-0.70208
H	-0.14367	0.02305	-1.77196
C	-0.31935	-3.59348	-1.13345
C	3.89046	-0.78845	0.03627
O	-1.79931	5.22777	1.38285
C	-2.86028	0.3132	0.14603
H	-2.8685	0.17697	1.22987
C	-1.83762	1.38617	-0.26218
H	-1.92357	1.56636	-1.34739
C	2.26894	1.13912	-0.96126
C	4.69535	-1.45524	-0.90555
H	4.2052	-1.90154	-1.76508
C	-5.19256	0.80215	0.60989
C	0.20256	-4.2919	-2.36136
H	-0.47209	-4.15337	-3.21067
H	0.35191	-5.35245	-2.15252
H	1.15939	-3.81461	-2.598
C	-2.06468	2.69683	0.47207
H	-3.12153	2.97707	0.4822
H	-1.70545	2.62779	1.50186
C	2.92243	3.48074	-0.79275
H	3.19963	4.32969	-0.17112
C	2.2439	1.28244	-2.358
H	1.99629	0.41906	-2.97069
C	-4.2639	-2.68188	-0.75333
C	1.16267	0.24942	2.37534
H	1.03636	1.2602	2.00749
C	2.6193	2.25695	-0.18976

H	2.68026	2.17938	0.89037
C	6.08107	-1.57007	-0.7466
H	6.6697	-2.09631	-1.49662
C	1.65082	-2.08224	2.00584
H	1.93505	-2.89016	1.33825
C	-1.2536	4.93435	0.33467
C	0.90691	-0.01973	3.72437
H	0.61575	0.79235	4.38693
C	2.89035	3.61195	-2.18471
H	3.13719	4.56128	-2.65411
C	6.71116	-1.02135	0.37511
H	7.78738	-1.11261	0.50607
C	1.01057	-1.32353	4.21683
H	0.80182	-1.53317	5.2631
C	1.37489	-2.35589	3.34743
H	1.44357	-3.37834	3.71051
C	2.55158	2.50306	-2.96528
H	2.53227	2.58481	-4.04981
C	4.54902	-0.25096	1.15671
H	3.97544	0.25474	1.93042
C	-6.48472	1.17077	-0.08179
H	-6.36801	2.11863	-0.61606
H	-7.28178	1.25377	0.65718
H	-6.73517	0.40643	-0.82395
C	-0.42601	5.87369	-0.50979
H	0.53003	5.40696	-0.76219
H	-0.27048	6.80743	0.03124
H	-0.94887	6.07753	-1.45038
C	5.93373	-0.36128	1.33111
H	6.40307	0.06302	2.21725
C	-4.86994	-3.83129	0.0188
H	-5.68815	-4.26675	-0.55546
H	-5.22657	-3.48755	0.99399
H	-4.09895	-4.58686	0.20133
O	1.88578	-1.78765	-1.2912

Int-2

Zero-point correction = 0.610623 (Hartree/Particle)

Thermal correction to Energy = 0.651171

Thermal correction to Enthalpy = 0.652116

Thermal correction to Gibbs Free Energy = 0.538117

Sum of electronic and zero-point Energies = -2332.750158

Sum of electronic and thermal Energies = -2332.709609

Sum of electronic and thermal Enthalpies = -2332.708665

Sum of electronic and thermal Free Energies = -2332.822664

O	1.12398	-0.4126	-1.33724
C	0.12523	0.50959	-0.81605
C	2.9053	0.23016	0.22307
C	2.15873	-0.89881	-0.50454
C	1.66072	-2.0069	0.44288
C	0.74219	1.60112	0.04899
C	1.95999	1.21117	0.93343
O	3.68025	0.999	-0.74169
O	5.51738	-0.29466	-0.40373
C	4.95074	0.62011	-0.97219
C	5.57879	1.48132	-2.04498
O	1.26468	2.63881	-0.89209
O	1.1177	4.31877	0.62858
C	1.33791	3.91388	-0.50273
C	1.70459	4.81744	-1.66131
O	2.78174	-2.54536	1.19815
O	3.24442	-4.09656	-0.39611
C	3.49874	-3.54864	0.66103
C	4.68129	-3.88959	1.5392
H	3.59076	-0.19057	0.9581
H	2.872	-1.36191	-1.19569
H	0.95248	-1.62929	1.17952
H	1.2121	-2.7992	-0.15648
H	-0.02039	2.092	0.6474
H	5.089	1.27893	-3.00311
H	5.43334	2.54148	-1.81929
H	6.64174	1.25094	-2.12053
H	2.07777	5.76878	-1.27928
H	2.44457	4.34081	-2.30863
H	0.80614	5.0025	-2.26055
H	5.06652	-4.87318	1.26705
H	5.46241	-3.13905	1.37442
H	4.40505	-3.85859	2.596
H	-0.19705	1.01652	-1.73034
P	-1.62519	-0.26796	0.03262
C	-3.41369	-0.90087	0.65671
C	-4.50849	-1.04134	-0.21456
C	-3.63577	-1.20049	2.01091
C	-5.76524	-1.45745	0.23419
H	-4.38301	-0.82475	-1.27462
C	-4.89056	-1.61889	2.47506
H	-2.798	-1.10154	2.69426
C	-5.96331	-1.7491	1.58891

H	-6.58927	-1.55534	-0.47056
H	-5.02797	-1.84483	3.53127
H	-6.93865	-2.07362	1.94528
C	-2.47126	1.32856	-0.52598
C	-2.76394	1.69828	-1.84779
C	-2.8323	2.22047	0.49791
C	-3.40347	2.90947	-2.13569
H	-2.51118	1.03811	-2.67092
C	-3.44988	3.4404	0.21525
H	-2.64286	1.93892	1.53044
C	-3.74264	3.79094	-1.10653
H	-3.63248	3.16268	-3.16855
H	-3.7088	4.1138	1.02874
H	-4.23107	4.736	-1.33009
C	-1.58667	-1.67281	-1.22913
C	-1.38565	-1.52569	-2.60856
C	-1.7202	-2.976	-0.72288
C	-1.36377	-2.63521	-3.45993
H	-1.21509	-0.545	-3.03795
C	-1.6622	-4.09054	-1.5616
H	-1.87671	-3.1162	0.34282
C	-1.49774	-3.92407	-2.94002
H	-1.21788	-2.48943	-4.52775
H	-1.75238	-5.08799	-1.13854
H	-1.46212	-4.78864	-3.59801
O	-0.9856	-0.34868	1.43232
H	2.50727	2.13044	1.16539
O	1.68834	0.51227	2.16161
C	1.11617	1.02018	3.28648
O	1.23238	0.37808	4.30914
C	0.35152	2.31679	3.21575
H	0.80157	3.06581	2.5614
H	-0.64387	2.08013	2.8267
H	0.25099	2.707	4.22944

Int-1′

Zero-point correction = 0.624778 (Hartree/Particle)

Thermal correction to Energy = 0.666094

Thermal correction to Enthalpy = 0.667038

Thermal correction to Gibbs Free Energy = 0.551898

Sum of electronic and zero-point Energies = -2333.238624

Sum of electronic and thermal Energies = -2333.197308

Sum of electronic and thermal Enthalpies = -2333.196364

Sum of electronic and thermal Free Energies = -2333.311504

O	0.22725	-1.95073	-1.84508
O	3.40359	-0.15994	1.28725
O	2.7921	-2.04031	-0.8185
O	0.56548	1.44851	-0.39238
O	2.88211	3.06255	-0.39428
O	0.11211	-1.10525	-3.94689
O	5.33579	0.11224	0.11524
O	2.18821	-3.65931	0.6601
C	1.83346	-1.10865	-0.28357
H	1.33016	-1.57164	0.56755
C	0.85898	-0.74626	-1.40229
H	1.43791	-0.34498	-2.24078
C	-0.1358	0.37285	-0.99839
C	-0.12832	-1.99821	-3.16289
O	1.42923	4.79823	-0.60724
C	2.58441	0.15137	0.14118
H	3.22532	0.49999	-0.67186
C	1.59303	1.2384	0.58458
H	1.15902	0.92959	1.53998
C	4.76114	-0.16065	1.14506
C	-0.84257	-3.27918	-3.49308
H	-0.44121	-4.12063	-2.92371
H	-0.76782	-3.46811	-4.56445
H	-1.89654	-3.15066	-3.22524
C	2.23532	2.60092	0.8067
H	3.00743	2.52511	1.5761
C	2.86105	-3.28756	-0.27703
C	2.36298	4.14793	-1.02737
C	5.42188	-0.54466	2.44523
H	6.50428	-0.51913	2.32239
H	5.10126	-1.54752	2.74366
H	5.11824	0.14337	3.23993
C	3.1071	4.41814	-2.31052
H	4.18668	4.35854	-2.15091
H	2.84068	3.65429	-3.04885
C	3.88472	-4.11929	-1.00716
H	4.01206	-5.07179	-0.49329
H	4.83695	-3.58465	-1.06507
H	3.54783	-4.29628	-2.03367
P	-1.87036	-0.03154	-0.17598
O	-2.32845	-0.71358	-1.80421
H	2.82874	5.40039	-2.69205
C	-1.3018	0.43907	1.63786
C	-1.46913	1.69283	2.2457

C	-0.67969	-0.56552	2.40558
C	-1.02019	1.94014	3.5499
H	-1.95846	2.49928	1.71211
C	-0.22473	-0.32831	3.70436
H	-0.55737	-1.56592	1.99746
C	-0.39117	0.93325	4.28393
H	-1.16965	2.92317	3.98906
H	0.25155	-1.1301	4.26204
H	-0.04538	1.12396	5.29602
C	-2.72514	-1.56062	0.48457
C	-2.45178	-2.84743	0.00271
C	-3.67347	-1.40807	1.50799
C	-3.10934	-3.95802	0.53807
H	-1.7168	-2.98175	-0.77834
C	-4.36094	-2.51485	2.01159
H	-3.87675	-0.42687	1.92501
C	-4.07495	-3.79599	1.53396
H	-2.86732	-4.95092	0.16946
H	-5.10618	-2.37357	2.78926
H	-4.59463	-4.65958	1.93897
C	-2.96035	1.43353	-0.47714
C	-4.35175	1.26272	-0.5626
C	-2.41477	2.70632	-0.71376
C	-5.18151	2.34866	-0.84762
H	-4.78803	0.27936	-0.42343
C	-3.24983	3.78685	-1.01761
H	-1.34298	2.86778	-0.65971
C	-4.63368	3.61436	-1.07687
H	-6.25628	2.20057	-0.90314
H	-2.80917	4.76239	-1.2015
H	-5.28041	4.45626	-1.30643
H	-0.49922	0.80448	-1.93252
H	1.4757	3.3211	1.11333
H	-2.43265	-0.03616	-2.48619

Int-1′-b

Zero-point correction = 0.624140 (Hartree/Particle)
Thermal correction to Energy = 0.665523
Thermal correction to Enthalpy = 0.666467
Thermal correction to Gibbs Free Energy = 0.550456
Sum of electronic and zero-point Energies = -2333.232136
Sum of electronic and thermal Energies = -2333.190753
Sum of electronic and thermal Enthalpies = -2333.189809

Sum of electronic and thermal Free Energies = -2333.305820

O	0.53135	0.51033	0.70468
C	-0.02699	-0.13204	-0.45344
C	2.72365	0.00933	-0.29104
C	1.8749	0.9889	0.55318
P	-2.09796	0.06172	-0.42515
C	1.82892	2.42014	0.0037
C	0.71089	-1.46029	-0.69063
C	2.14255	-1.39858	-0.14413
O	4.06135	0.01486	0.2456
O	5.00362	0.25591	-1.81196
C	5.11283	0.13303	-0.61169
C	6.414	0.0864	0.1503
O	2.94739	-2.32861	-0.89736
O	3.61829	-3.43953	0.97219
C	3.63441	-3.29826	-0.23104
C	4.39939	-4.15883	-1.20549
O	0.13196	-2.63672	-0.04282
O	-0.97872	-3.20752	-1.92371
C	-0.60473	-3.47403	-0.78917
C	-0.90285	-4.75839	-0.06624
O	3.15355	2.97162	-0.17532
O	3.17623	3.69726	1.97275
C	3.70656	3.61325	0.88412
C	5.03342	4.22811	0.51347
H	0.19868	0.47895	-1.3402
H	2.77808	0.29629	-1.34461
H	2.29575	1.00561	1.56323
H	1.37774	2.44639	-0.98965
H	1.25459	3.04873	0.68401
H	0.75047	-1.66982	-1.76269
H	2.15319	-1.69494	0.90692
H	6.43386	0.87048	0.91297
H	6.50529	-0.87179	0.67118
H	7.24507	0.21507	-0.54276
H	5.04238	-3.5366	-1.83472
H	4.99562	-4.88695	-0.65605
H	3.70067	-4.67736	-1.8697
H	-1.57209	-5.37078	-0.66995
H	0.03029	-5.29819	0.12347
H	-1.35837	-4.53847	0.90288
H	5.62051	4.39783	1.4166
H	5.57813	3.60078	-0.19531
H	4.85033	5.19365	0.02888

C	-1.81722	1.90121	-0.31402
C	-1.72134	2.59289	-1.53091
C	-1.7684	2.62192	0.88587
C	-1.57284	3.98213	-1.54595
H	-1.78068	2.05152	-2.47176
C	-1.63286	4.0128	0.86746
H	-1.83413	2.11079	1.84005
C	-1.53112	4.69738	-0.34628
H	-1.50118	4.50344	-2.49641
H	-1.59914	4.5587	1.80611
H	-1.4236	5.77804	-0.35684
C	-4.00855	0.37148	-0.65621
C	-4.7223	0.95294	0.40738
C	-4.72002	0.10166	-1.83654
C	-6.0819	1.24993	0.30571
H	-4.2176	1.17419	1.3443
C	-6.08415	0.40155	-1.94822
H	-4.20802	-0.35219	-2.6758
C	-6.77209	0.97616	-0.87946
H	-6.60148	1.69352	1.15105
H	-6.60579	0.17974	-2.87589
H	-7.83037	1.20693	-0.96494
C	-2.33525	-0.92183	1.13553
C	-3.28436	-1.95752	1.09398
C	-1.61021	-0.71599	2.31994
C	-3.49848	-2.77347	2.20625
H	-3.87073	-2.1186	0.19476
C	-1.85087	-1.51449	3.44205
H	-0.84766	0.04976	2.36524
C	-2.78895	-2.54738	3.38913
H	-4.23395	-3.57132	2.15233
H	-1.29343	-1.33017	4.356
H	-2.96795	-3.16902	4.26178
O	-2.09334	-0.66914	-1.91085
H	-1.48489	-1.42845	-2.02864

Int-2′-b

Zero-point correction = 0.610773 (Hartree/Particle)

Thermal correction to Energy = 0.652180

Thermal correction to Enthalpy = 0.653124

Thermal correction to Gibbs Free Energy = 0.536251

Sum of electronic and zero-point Energies = -2332.765082

Sum of electronic and thermal Energies = -2332.723676

Sum of electronic and thermal Enthalpies = -2332.722732
Sum of electronic and thermal Free Energies = -2332.839604

O	0.1104	-2.41059	-1.5412
O	3.29765	-0.15633	1.18037
O	2.82066	-2.23646	-0.81249
O	0.63305	1.21233	-0.92815
O	3.02861	2.80473	-0.52248
O	-0.18347	-2.17245	-3.77994
O	5.2701	0.27104	0.12759
O	2.47935	-3.72203	0.8793
C	1.81039	-1.30329	-0.33153
H	1.36508	-1.7157	0.57347
C	0.77201	-1.1328	-1.4427
H	1.31358	-0.96573	-2.3817
C	-0.21602	0.04947	-1.22176
C	-0.42638	-2.74697	-2.73581
O	4.27597	4.00716	0.94067
C	2.50616	0.01407	-0.02811
H	3.16786	0.30182	-0.84592
C	1.45187	1.10769	0.21392
H	0.86365	0.83483	1.10334
C	4.63808	-0.00824	1.12849
C	-1.3498	-3.92771	-2.59316
H	-0.95033	-4.66057	-1.88656
H	-1.52295	-4.38359	-3.56933
H	-2.28695	-3.53299	-2.18665
C	2.0667	2.46874	0.50512
H	2.56588	2.47617	1.47567
C	3.02307	-3.39713	-0.15789
C	4.09975	3.53997	-0.17057
C	5.2445	-0.21446	2.49712
H	6.3273	-0.30301	2.40477
H	4.82211	-1.10217	2.97485
H	5.00612	0.64865	3.1279
C	5.06189	3.67263	-1.32517
H	5.61712	2.73239	-1.41403
H	4.5257	3.83629	-2.26311
C	4.0284	-4.24771	-0.90247
H	4.29694	-5.11157	-0.29376
H	4.9175	-3.66034	-1.14848
H	3.58674	-4.58539	-1.84582
P	-2.16454	-0.118	-0.31743
O	-2.44645	-1.26049	-1.30426
H	5.7589	4.48826	-1.12922

C	-2.3862	1.66215	-0.8395
C	-3.14134	1.8372	-2.01091
C	-1.86706	2.80097	-0.20932
C	-3.3752	3.11108	-2.53248
H	-3.54563	0.96022	-2.51019
C	-2.11244	4.07982	-0.72038
H	-1.25838	2.70436	0.68291
C	-2.86647	4.2417	-1.88517
H	-3.95863	3.22053	-3.44368
H	-1.70472	4.94945	-0.20975
H	-3.05122	5.23527	-2.28587
C	-1.48112	-0.4242	1.39317
C	-0.98521	-1.71417	1.64178
C	-1.51232	0.49004	2.45959
C	-0.50495	-2.07092	2.90486
H	-0.97351	-2.43947	0.83332
C	-1.01584	0.1416	3.71882
H	-1.94455	1.47569	2.32451
C	-0.5066	-1.1404	3.94779
H	-0.11552	-3.07284	3.0648
H	-1.04101	0.87083	4.52553
H	-0.12345	-1.4123	4.92798
C	-4.004	-0.157	0.46209
C	-4.71519	-1.3712	0.44433
C	-4.63957	0.93266	1.08333
C	-5.98237	-1.49709	1.02312
H	-4.26007	-2.22073	-0.05505
C	-5.90993	0.82295	1.66282
H	-4.15058	1.90384	1.10675
C	-6.5895	-0.39765	1.63935
H	-6.50095	-2.45382	0.98628
H	-6.36947	1.69418	2.12678
H	-7.57721	-0.48869	2.0862
H	-0.59101	0.29127	-2.21887
H	1.2811	3.22871	0.49664

Int-2′

Zero-point correction = 0.610798 (Hartree/Particle)
Thermal correction to Energy = 0.652155
Thermal correction to Enthalpy = 0.653099
Thermal correction to Gibbs Free Energy = 0.534831
Sum of electronic and zero-point Energies = -2332.760611
Sum of electronic and thermal Energies = -2332.719254
Sum of electronic and thermal Enthalpies = -2332.718310

Sum of electronic and thermal Free Energies = -2332.836578

O	-0.69402	0.98971	-0.30875
C	0.01601	0.37988	0.81345
C	-2.81555	-0.18397	-0.01254
C	-2.09403	1.1785	-0.16162
P	2.06822	0.21686	0.38956
C	-2.38059	2.17171	0.98139
C	-0.79138	-0.84452	1.32319
C	-1.81602	-1.29937	0.28296
O	-3.44673	-0.56059	-1.2747
O	-5.48525	0.21047	-0.63786
C	-4.76356	-0.33909	-1.44794
C	-5.22782	-0.88402	-2.77903
O	-2.59189	-2.41156	0.78285
O	-3.09924	-4.55465	0.90393
C	-2.39631	-3.69947	0.40337
C	-1.33957	-4.00706	-0.63437
O	0.04829	-1.98772	1.60265
O	-0.04791	-1.5836	3.83984
C	0.4435	-2.16047	2.89108
C	1.56039	-3.16524	2.97926
O	-3.7944	2.34794	1.233
O	-3.99302	3.78207	-0.5103
C	-4.48942	3.16933	0.41585
C	-5.94633	3.20614	0.8027
H	0.0601	1.10969	1.62864
H	-3.59179	-0.15179	0.75465
H	-2.42955	1.63536	-1.09708
H	-1.98613	1.81264	1.93187
H	-1.92352	3.13137	0.73212
H	-1.31713	-0.61781	2.25686
H	-1.30698	-1.5664	-0.64327
H	-4.52351	-0.62065	-3.57223
H	-5.27242	-1.97698	-2.72214
H	-6.22114	-0.49488	-3.00392
H	-0.35739	-3.64375	-0.324
H	-1.31184	-5.08816	-0.77006
H	-1.5767	-3.53199	-1.59189
H	1.6214	-3.56106	3.99416
H	1.42956	-3.97429	2.25583
H	2.47745	-2.61987	2.73218
H	-6.4379	4.03207	0.28745
H	-6.40243	2.25751	0.50275
H	-6.05754	3.30718	1.8856

C	2.01947	2.0588	0.05253
C	2.18794	2.86706	1.18871
C	1.87649	2.68641	-1.1931
C	2.20757	4.2606	1.08417
H	2.31319	2.39077	2.15818
C	1.89654	4.07965	-1.30009
H	1.74948	2.09639	-2.09429
C	2.0598	4.87543	-0.16233
H	2.34091	4.8651	1.97854
H	1.78012	4.54377	-2.27683
H	2.07235	5.95913	-0.24724
C	4.01485	0.22865	-0.06464
C	4.50419	0.44395	-1.36569
C	4.97295	0.06839	0.95127
C	5.87315	0.49937	-1.64643
H	3.80644	0.56415	-2.19233
C	6.34739	0.12283	0.68559
H	4.61371	-0.10623	1.9605
C	6.80662	0.33902	-0.61676
H	6.21118	0.6649	-2.668
H	7.0606	-0.00639	1.49821
H	7.87299	0.38032	-0.82818
C	1.74085	-0.91733	-1.07144
C	2.33949	-2.1879	-1.00795
C	0.9677	-0.61	-2.20224
C	2.17686	-3.11894	-2.03454
H	2.95207	-2.43855	-0.14659
C	0.82193	-1.53413	-3.24567
H	0.45867	0.34325	-2.26478
C	1.42353	-2.79192	-3.16792
H	2.65032	-4.09495	-1.95696
H	0.2312	-1.26515	-4.11862
H	1.30852	-3.50842	-3.97755
O	2.29197	-0.2862	1.8253

TS

Zero-point correction = 0.610175 (Hartree/Particle)
Thermal correction to Energy = 0.650786
Thermal correction to Enthalpy = 0.651730
Thermal correction to Gibbs Free Energy = 0.537762
Sum of electronic and zero-point Energies = -2332.746881
Sum of electronic and thermal Energies = -2332.706270
Sum of electronic and thermal Enthalpies = -2332.705326
Sum of electronic and thermal Free Energies = -2332.819294

O	1.19948	-0.11562	-1.42749
C	0.1699	0.67424	-0.7402
C	2.91677	0.23461	0.31161
C	2.23645	-0.7204	-0.68725
C	1.78221	-2.03268	-0.01638
C	0.77318	1.63307	0.24186
C	1.91454	1.07304	1.1198
O	3.78179	1.1646	-0.40376
O	5.56532	-0.22935	-0.20589
C	5.06088	0.80465	-0.60552
C	5.79237	1.85727	-1.40833
O	1.40526	2.77931	-0.52837
O	1.20834	4.24247	1.19677
C	1.48535	3.98235	0.03284
C	1.93855	5.02837	-0.96745
O	2.92357	-2.68379	0.6084
O	3.49334	-3.7463	-1.31654
C	3.71217	-3.46547	-0.1522
C	4.92141	-3.92488	0.62864
H	3.53502	-0.33763	1.00314
H	2.99065	-0.99544	-1.43346
H	1.07892	-1.86022	0.79428
H	1.35485	-2.69086	-0.77126
H	0.00177	2.08089	0.86121
H	5.38054	1.88974	-2.42231
H	5.64807	2.84602	-0.96378
H	6.85355	1.61066	-1.45073
H	2.30336	5.90985	-0.43754
H	2.70954	4.62596	-1.62907
H	1.08343	5.31514	-1.58983
H	5.38177	-4.7751	0.12353
H	5.6365	-3.09562	0.66752
H	4.65006	-4.18308	1.6553
H	-0.21879	1.27382	-1.56572
P	-1.93056	-0.11622	0.13348
C	-3.72679	-0.68108	0.64292
C	-4.65072	-1.2087	-0.27556
C	-4.15233	-0.53585	1.97264
C	-5.94475	-1.57114	0.10817
H	-4.35882	-1.35199	-1.31373
C	-5.44587	-0.89802	2.36963
H	-3.44147	-0.14052	2.69111
C	-6.34996	-1.41567	1.43852
H	-6.63487	-1.9781	-0.62828

H	-5.74496	-0.77796	3.40914
H	-7.35516	-1.69817	1.74332
C	-2.59362	1.23019	-0.96442
C	-2.98069	1.09308	-2.30706
C	-2.73595	2.48833	-0.3569
C	-3.48782	2.18222	-3.02026
H	-2.89677	0.13399	-2.80708
C	-3.23953	3.57912	-1.07085
H	-2.45759	2.60346	0.68684
C	-3.61719	3.43291	-2.40808
H	-3.78431	2.05062	-4.05836
H	-3.33833	4.54302	-0.57731
H	-4.00974	4.2795	-2.9655
C	-1.56683	-1.72195	-0.72515
C	-1.14624	-1.83686	-2.05818
C	-1.69391	-2.89529	0.03825
C	-0.90709	-3.09163	-2.62605
H	-0.96821	-0.94871	-2.65148
C	-1.41771	-4.14658	-0.51612
H	-2.01307	-2.82597	1.07425
C	-1.03791	-4.25174	-1.85782
H	-0.58603	-3.15744	-3.66245
H	-1.50742	-5.03894	0.0983
H	-0.82708	-5.22436	-2.29441
O	-1.4581	0.28071	1.53352
H	2.44443	1.90911	1.58638
O	1.49278	0.14528	2.13822
C	0.9152	0.48278	3.32693
O	0.85675	-0.37834	4.17956
C	0.36251	1.87129	3.5196
H	0.91896	2.65802	3.00719
H	-0.65177	1.85171	3.10655
H	0.3136	2.07275	4.59089

TS′

Zero-point correction = 0.608841 (Hartree/Particle)

Thermal correction to Energy = 0.649903

Thermal correction to Enthalpy = 0.650847

Thermal correction to Gibbs Free Energy = 0.533963

Sum of electronic and zero-point Energies = -2332.751156

Sum of electronic and thermal Energies = -2332.710094

Sum of electronic and thermal Enthalpies = -2332.709150

Sum of electronic and thermal Free Energies = -2332.826034

O	0.61572	0.37792	0.87873

C	0.10772	-0.08315	-0.4146
C	2.87629	-0.06873	0.02888
C	1.96514	0.82342	0.9082
P	-2.47621	-0.15093	-0.29784
C	2.01735	2.30861	0.54878
C	0.90206	-1.28325	-0.89234
C	2.21414	-1.43729	-0.12594
O	4.14086	-0.26738	0.71338
O	5.35489	0.73837	-0.92996
C	5.28702	0.12539	0.11567
C	6.48284	-0.32307	0.92419
O	3.1467	-2.29413	-0.82699
O	4.38023	-4.11796	-0.99648
C	3.50993	-3.52085	-0.39306
C	2.82078	-4.10619	0.82564
O	0.20182	-2.57983	-0.70871
O	0.01338	-2.89423	-2.9562
C	-0.28736	-3.18523	-1.81464
C	-1.25823	-4.28188	-1.45108
O	3.39357	2.74917	0.68173
O	2.86165	4.79546	-0.12594
C	3.68888	3.99665	0.26968
C	5.17495	4.25426	0.35704
H	0.24401	0.72107	-1.14844
H	3.08516	0.3687	-0.94944
H	2.30751	0.70267	1.94557
H	1.67584	2.49152	-0.47287
H	1.38683	2.88005	1.23459
H	1.10764	-1.21307	-1.96235
H	2.0092	-1.83581	0.86873
H	6.65635	-1.38745	0.73104
H	7.36372	0.24083	0.61512
H	6.30143	-0.20008	1.99491
H	1.73343	-4.06653	0.72378
H	3.15407	-5.1388	0.9289
H	3.09566	-3.55287	1.73039
H	-1.33701	-4.99364	-2.27475
H	-0.96703	-4.79167	-0.52831
H	-2.22406	-3.79192	-1.28877
H	5.53218	4.07084	1.37519
H	5.69931	3.55935	-0.30663
H	5.38399	5.28414	0.06691
C	-2.15677	1.53913	-0.95117
C	-2.42153	1.72704	-2.31773

C	-1.68155	2.62481	-0.19926
C	-2.21444	2.97132	-2.91768
H	-2.77456	0.88687	-2.90936
C	-1.48073	3.87007	-0.80059
H	-1.43277	2.49087	0.84795
C	-1.74781	4.05091	-2.1617
H	-2.41469	3.09499	-3.97923
H	-1.09748	4.69667	-0.2078
H	-1.58051	5.01809	-2.628
C	-4.37491	0.04588	-0.10716
C	-5.16676	-1.11094	-0.20821
C	-5.03186	1.26047	0.14816
C	-6.55361	-1.06185	-0.04447
H	-4.67641	-2.0512	-0.44026
C	-6.42113	1.31959	0.31
H	-4.46194	2.18407	0.20331
C	-7.18903	0.15632	0.21874
H	-7.14056	-1.97383	-0.13112
H	-6.90166	2.2771	0.49959
H	-8.26875	0.19927	0.34122
C	-2.147	-0.38022	1.50698
C	-1.57032	-1.5911	1.91392
C	-2.54298	0.53951	2.48875
C	-1.38745	-1.87201	3.26881
H	-1.24727	-2.29526	1.15499
C	-2.33242	0.27208	3.84513
H	-3.03673	1.46415	2.2081
C	-1.75782	-0.93752	4.24183
H	-0.94457	-2.82001	3.56572
H	-2.63326	1.00483	4.59022
H	-1.60435	-1.15193	5.29664
O	-2.42637	-1.34778	-1.23201

3a

Zero-point correction = 0.279308 (Hartree/Particle)

Thermal correction to Energy = 0.298159

Thermal correction to Enthalpy = 0.299103

Thermal correction to Gibbs Free Energy = 0.232218

Sum of electronic and zero-point Energies = -993.120582

Sum of electronic and thermal Energies = -993.101731

Sum of electronic and thermal Enthalpies = -993.100787

Sum of electronic and thermal Free Energies = -993.167672

O	-0.20141	0.528	2.36924
C	1.13628	0.68497	2.51202

C	0.13845	0.6576	-0.08332
C	-0.70158	0.11792	1.07961
C	-0.84197	-1.41372	1.08234
C	2.04886	0.65381	1.52937
C	1.64946	0.45211	0.1031
O	-0.0483	2.09059	-0.19116
O	-1.83891	1.77666	-1.54602
C	-1.09058	2.52415	-0.95215
C	-1.18158	4.02797	-0.93559
O	1.91664	-0.91128	-0.34909
O	3.27721	-2.47348	-1.11517
C	3.11677	-1.30156	-0.86427
C	4.1745	-0.2469	-1.12587
O	-1.50942	-1.84465	-0.1192
O	-3.52857	-1.50242	0.85316
C	-2.87094	-1.82858	-0.11285
C	-3.42038	-2.24194	-1.45244
H	1.39202	0.87122	3.55014
H	-0.19495	0.18036	-1.00603
H	-1.70258	0.54988	1.01968
H	0.12659	-1.91226	1.07165
H	-1.42132	-1.70779	1.95915
H	3.09153	0.80344	1.78562
H	2.16123	1.1551	-0.55903
H	-1.48454	4.36188	0.06228
H	-0.20743	4.47558	-1.14984
H	-1.92015	4.35353	-1.66769
H	4.41596	0.32721	-0.22677
H	5.07077	-0.75592	-1.4785
H	3.84198	0.45763	-1.89595
H	-4.48144	-2.47098	-1.35364
H	-3.29557	-1.41062	-2.15492
H	-2.87394	-3.10046	-1.85009
