# Supplementary material for: P(v) intermediate-mediated E1cB elimination for the synthesis of glycals
Source: Chem Sci. 2022 Apr 22;13(19):5588–96. doi: 10.1039/d2sc01423h (PMC9116453; doi:10.1039/d2sc01423h)
Supplement: SC-013-D2SC01423H-s002 [file SC-013-D2SC01423H-s002.pdf]

Electronic Supplementary Material (ESI) for Chemical Science.
This journal is © The Royal Society of Chemistry 2022

This version of the ESI published 12/05/2022 replaces the previous version published 22/04/2022. The authors regret that there were errors in the section "5. General Procedure for the Synthesis of Glycals 3" and in the NMR-data of compound 3ag which are now corrected.

Supporting Information

P(V) Intermediates-mediated E1cb Elimination for the Synthesis of Glycals

Fen Liu, Haiyang Huang,* Longgen Sun, Zeen Yan, Xiao Tan, Jing Li, Xinyue Luo, Haixin Ding, Qiang Xiao*

Contents

P(V) Intermediates-mediated E1cb Elimination for the Synthesis of Glycals S1
1. **Represent synthesis of glycals.** S2
2. **Table S1. Optimization of reaction conditions.** S3
3. **Conventional Preparation Route for 1*D*-glycals and Our Synthetic Method.** S3
4. **Mechanistic Investigation** S5
5. **General Procedure for the Synthesis of Glycals 3.** S12
6. **The Analytical and Spectral Characterization Data of Compounds 3.** S12
7. **General Procedure for the Synthesis of Deuterated Glycals 1*D*-3.** S28
8. **The Analytical and Spectral Characterization Data of Deuterated Compounds 1*D*-3.** S28
9. **The Larger-scale (200 g) Reactions.** S34
10. **References** S35
11. **X-ray Crystal Structures of Compounds 2a′.** S36
12. **Copies of ^{31}P NMR, ^{1}H NMR, ^{13}C NMR Spectra of Compounds 2.** S39
13. **Copies of ^{1}H NMR, ^{13}C NMR Spectra of Compounds 3.** S53
14. **Copies of ^{1}H NMR, ^{13}C NMR Spectra of Compounds 1*D*-3.** S95

1. Represent synthesis of glycals.

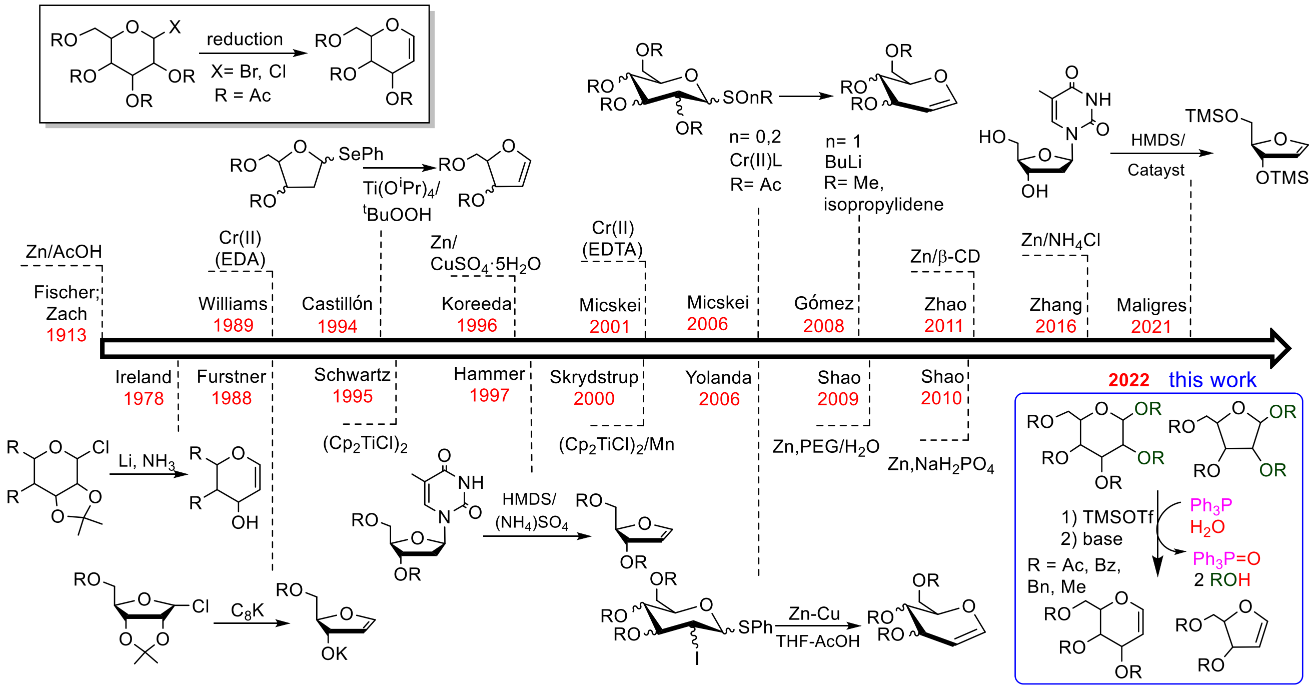

Fig. S1 Represent synthesis of glycals from its first discovery in 1913 up to the present.

Protocols for the preparation of glycals were limited as shown in Fig. S1. Actually, since the Fischer-Zach method was first developed in 1913, which treated the air-sensitive peracetylated glycosyl bromide with much excess zinc in acetic acid to afford the corresponding glycals, it is still the most widely adopted approach for the preparation of glycals to date.[1] To obviate the requirement of excess zinc and acetic acid, numerous synthetic methods have been explored, such as Li/NH_3,[2] Cr(II)/EDA,[3] $(CP_2TiCl)_2$,[4] Zn/$CuSO_4$,[5] Zn/PEG-H_2O,[6] and etc. However, no substantial improvement was achieved. Therefore, nearly all disadvantages of the Fischer-Zach method remained, including expensive and excess metallic reagents, complicated operations. In addition, it is worth mentioning that the Fischer-Zach method was not suitable for furanoid glycals, which will further eliminate to give furans. Although some other methodologies for the synthesis of glycals have also been developed, which applied thiophenyl glycoside,[7] glycosyl sulfones,[8] and glycosyl sulfoxides,[9] as starting materials. These protocols seemingly showed some more serious drawbacks, such as the multistep preparation of the appropriate precursor and poor generality. In an attempt to compensate the deficiencies, an electrochemical strategy was also developed recently.[10] However, it requires using the toxic mercury cathode, complex divided electrochemical cell, and

strongly acidic conditions. Therefore, it is highly worthwhile to conceive an innovative and green methodology to synthesize glycals with improved flexibility, efficiency, generality, and practicality.

2. Table S1. Optimization of reaction conditions[a]

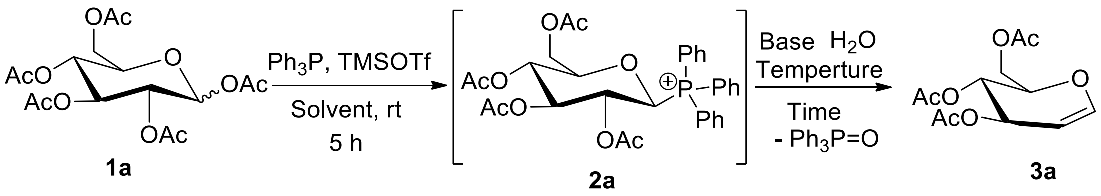

Entry	Solvent	Base	Time	Temperture	**3a** (yield, %)
1	THF	K_2CO_3	2 h	80 °C	23
2	Toluene	K_2CO_3	2 h	80 °C	18
3	CH_3CN	K_2CO_3	2 h	80 °C	30
4	DCM	K_2CO_3	2 h	80 °C	69
5	DCM	K_2CO_3	4 h	80 °C	75
6	DCM	K_2CO_3	4 h	60 °C	76
7	DCM	NaOH (3 M)	1 h	60 °C	68
8	DCM	NaOH (3 M)	0.5 h	25 °C	76
9	DCM	NaOH (2 M)	1 h	25 °C	88
10	DCM	NaOH (1 M)	2 h	25 °C	94
11[b]	DCM	NaOH (1 M)	2 h	25 °C	60

[a]Reaction conditions: **1a** (1 mmol), Ph_3P (1.2 mmol) and TMSOTf (1.1 mmol) in solvent (3.0 ml) at rt for 0.5-4 h, and then base and H_2O (1.0 ml) were added at rt. Total isolated yield for two steps.
[b]TMSOTf was replaced with BF_3.

3. Conventional Preparation Route for 1*D*-glycals and Our Synthetic Method.

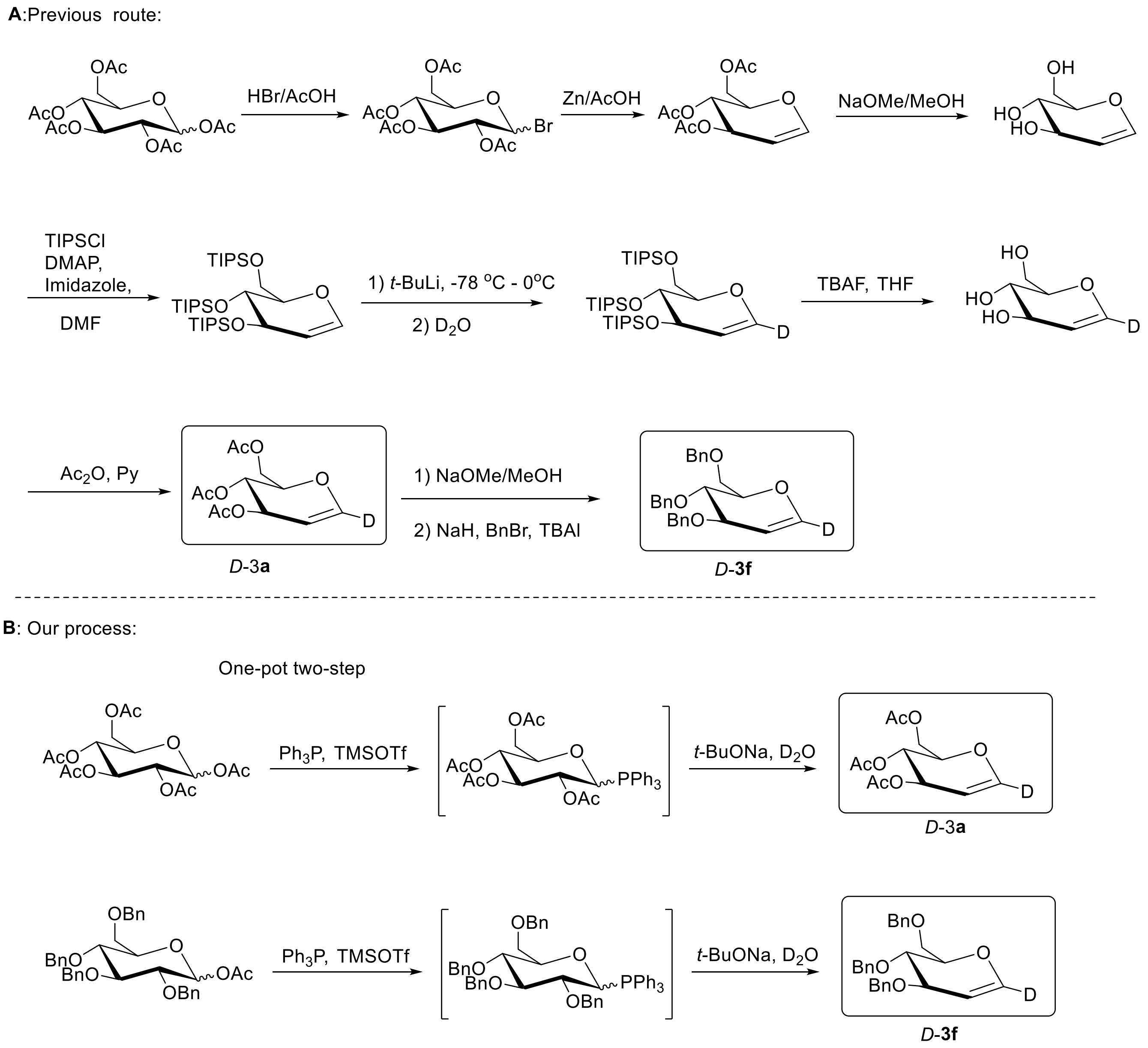

Scheme S1. Previous reported preparation method and our direct one-pot two-step strategy for 1*D*-glucose derivatives.

Multistep reactions (at least 7 steps for *D*-**3f**, 8 steps for *D*-**3f**) were required for the synthesis of triacetyl or tribenzyl protected 1-*D*-glucose in previous report (as shown in Scheme S1A).[12] Several protections/deprotections as additional processes are necessary due to the incompatibility of protecting group to halogen/lithium-exchange or reductive elimination reaction. However, only two steps are required even in one-pot system from the commercial substrates to 1-deuterium-glycals by our developed P(V) intermediates-mediated elimination as shown in Scheme S1B.

4. Mechanistic Investigation

4.1 The Intermediate 2a

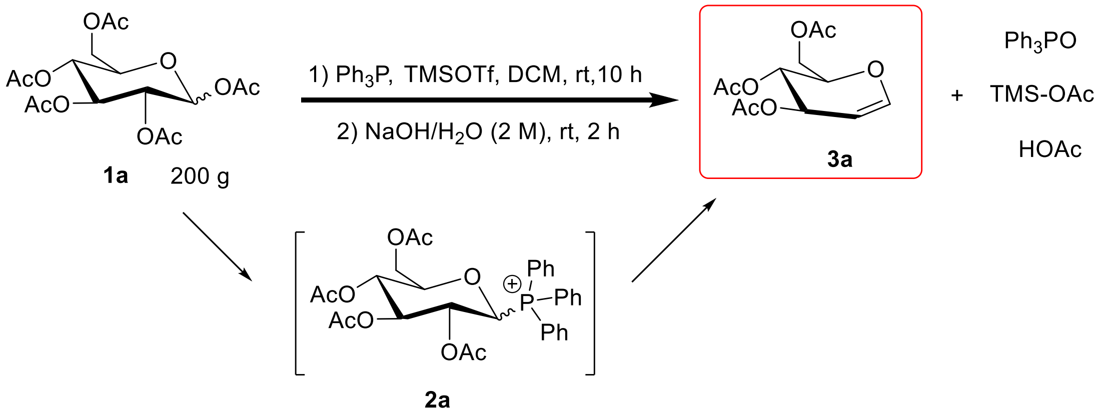

Scheme S2. Synthesis of glycal **3a** via the intermediate **2a**.

2a: White solid; yield (93%); ^{31}P NMR (162 MHz, Chloroform-*d*) δ 22.14; ^{1}H NMR (400 MHz, Chloroform-*d*) δ 7.87 – 7.76 (m, 9H), 7.66 (dt, *J* = 10.8, 5.4 Hz, 6H), 6.60 (dd, *J* = 10.4, 2.0 Hz, 1H), 5.47 (t, *J* = 9.2 Hz, 1H), 5.16 (dd, *J* = 19.6, 10.2 Hz, 1H), 4.85 (t, *J* = 9.8 Hz, 1H), 4.59 (dd, *J* = 9.6, 4.2 Hz, 1H), 4.14 (d, *J* = 12.2 Hz, 1H), 3.97 (dd, *J* = 12.8, 5.2 Hz, 1H), 1.96 (s, 3H), 1.86 (s, 3H), 1.85 (s, 3H), 1.37 (s, 3H); ^{13}C NMR (101 MHz, Chloroform-*d*) δ 170.13 (s, C), 169.71 (s, C), 169.23 (s, C), 169.11 (s, C), 135.50 (d, J_{C-P} = 3.0 Hz, 3CH), 134.55 (d, J_{C-P} = 10.0 Hz, 6CH), 130.38 (d, J_{C-P} = 13.0 Hz, 6CH), 120.83 (d, J_{C-P} = 320.8 Hz C), 116.01 (s, C), 115.16 (s, C), 74.13 (d, J_{C-P} =142.4 Hz, CH), 70.30 (s, CH), 69.59 (s, CH), 67.78 (s, CH), 67.28 (s, CH_2), 61.38 (s, CH), 20.57 (s, CH_3), 20.54 (s, CH_3), 20.40 (s, CH_3), 19.88 (s, CH_3). HRMS Calcd. For $C_{32}H_{34}O_9P^+$ [M−OTf$^-$]$^+$, 593.1940. Found: 593.1935.

4.2 The Isotope-labeling Experiments

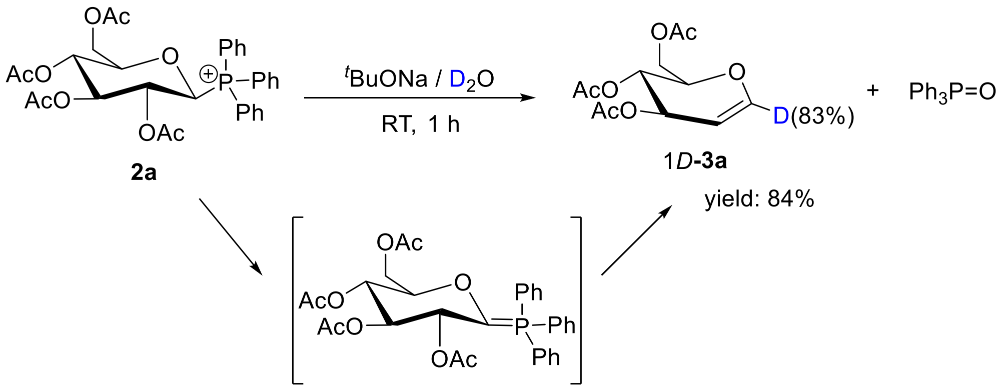

Scheme S3. The hydroylsis-elimination reaction of glycosylphosphonium **2a** by D_2O/base. The major product 1*D*-**3a** (83%) was obtained, implying that the ylide species is the key intermediate.

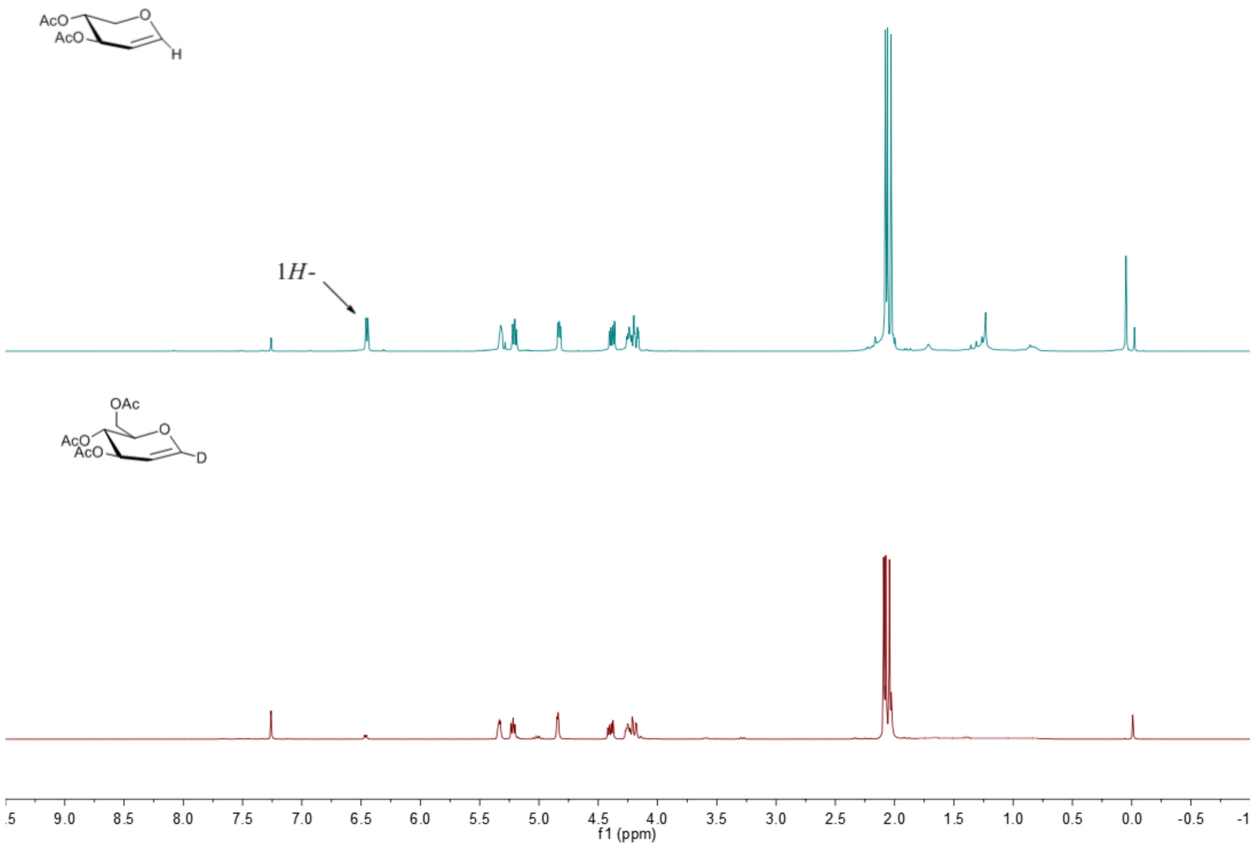

Fig S2. ^{1}H NMR ($CDCl_3$-*d*) of **3a** and 1-*D*-**3a**

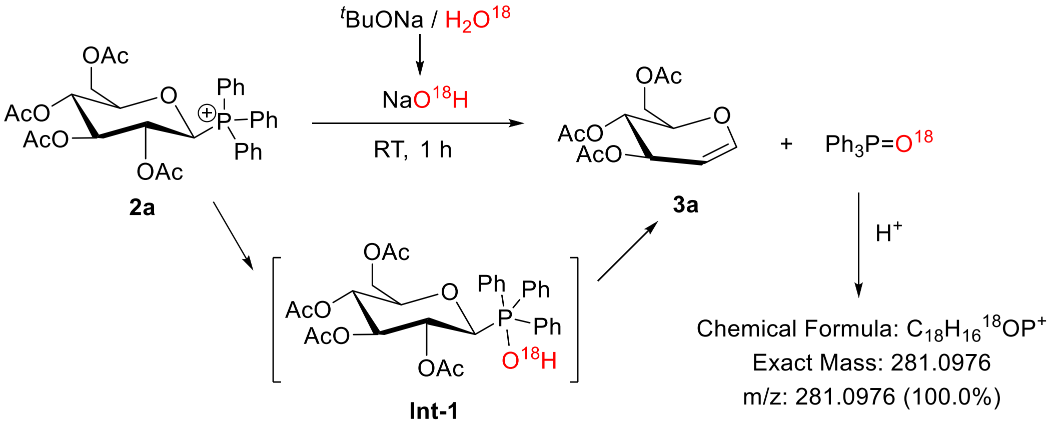

Scheme S4. ^{18}O-labeling reaction.

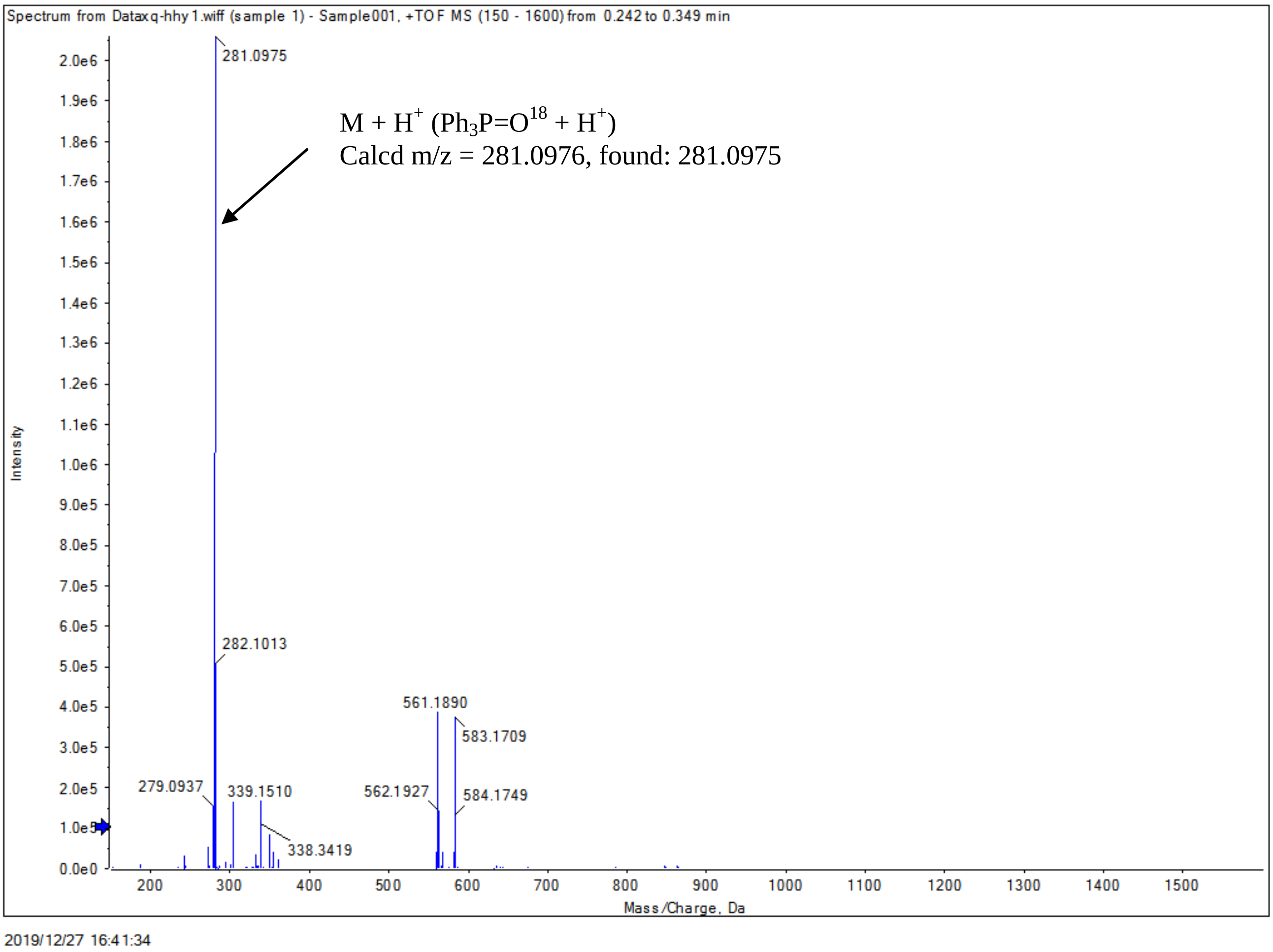

Fig S3. The HRMS of $O^{18}PPh_3$

4.3 The Track Experiments (^{31}P-NMR)

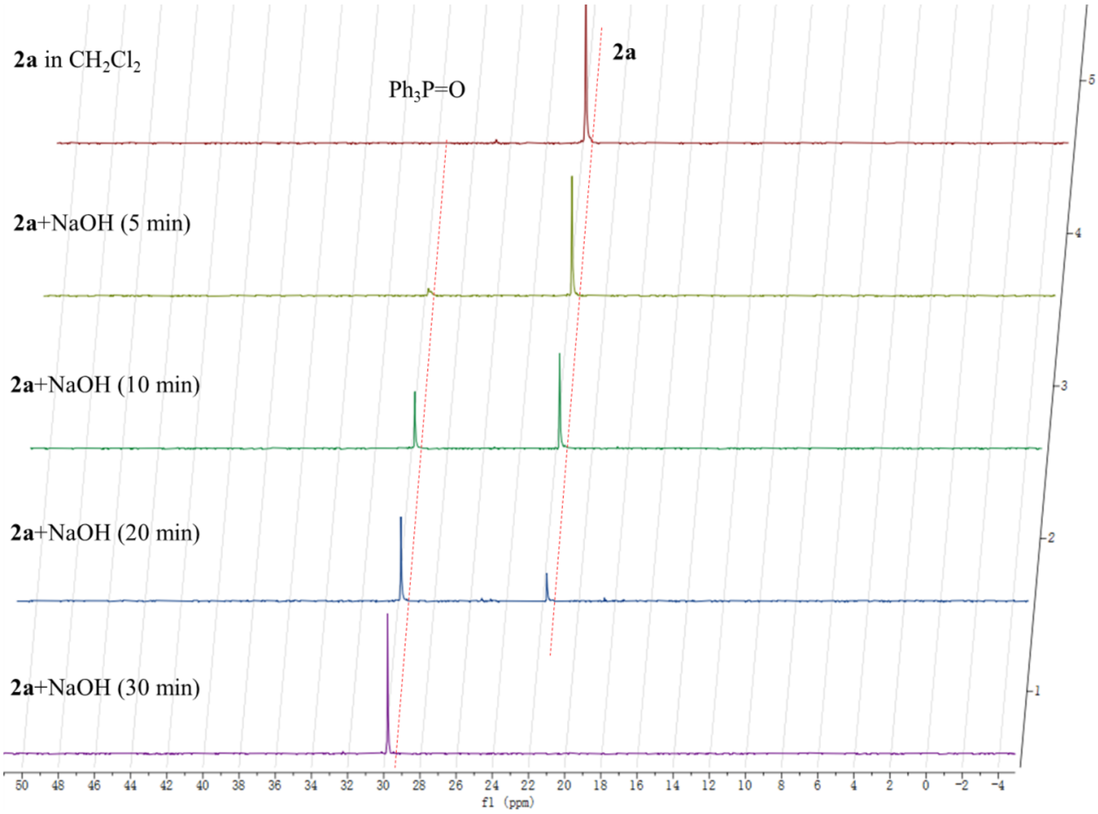

Fig. S4 The ^{31}P NMR tracing of hydrolysis reaction of **2a** with NaOH at room temperature in CH_2Cl_2. We don't observe any an intermediate in this reaction condition, which imply that the formation and conversion of the intermediates are the fast processes.

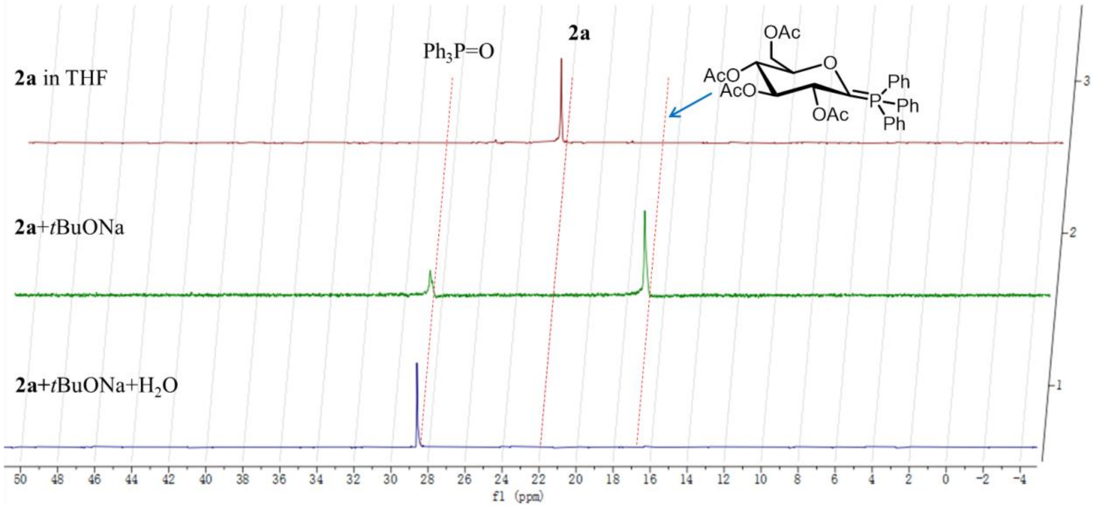

Fig. S5 The ^{31}P NMR tracing of treating **2a** with ^{t}BuNa and then water in hydropenic THF. We can observe the glycosyl ylide species, which indicate that glycosyl ylide could be one of key intermediates and both its formation and conversion are very fast.

4.4 Computation of Hydrolysis-E1cb Reaction.

Computational Methods

Density functional theory (DFT) investigations were performed to delineate the detailed mechanism of the hydrolysis-elimination reaction of glucosylphosphonium salt **2a**. All density functional theory calculations were carried out with the Gaussian 16 programs. The geometry optimizations and frequency calculations of the reactants, transition states, and products were performed with the B3LYP method at the 6-31+G(d, p), and energy and frequency calculations at M06-2X/6-311+G(d, p)/IEF-PCM$_{DCM}$ level. The Localized orbital locator (LOL) analysis and highest occupied molecular orbital (HOMO) distribution of transition state TS along its intrinsic reaction coordinate (IRC) are performed at B3LYP/6-31+G(d, p) level. The energies given in this work are M06-2X calculated Gibbs free energies in DCM solvent.

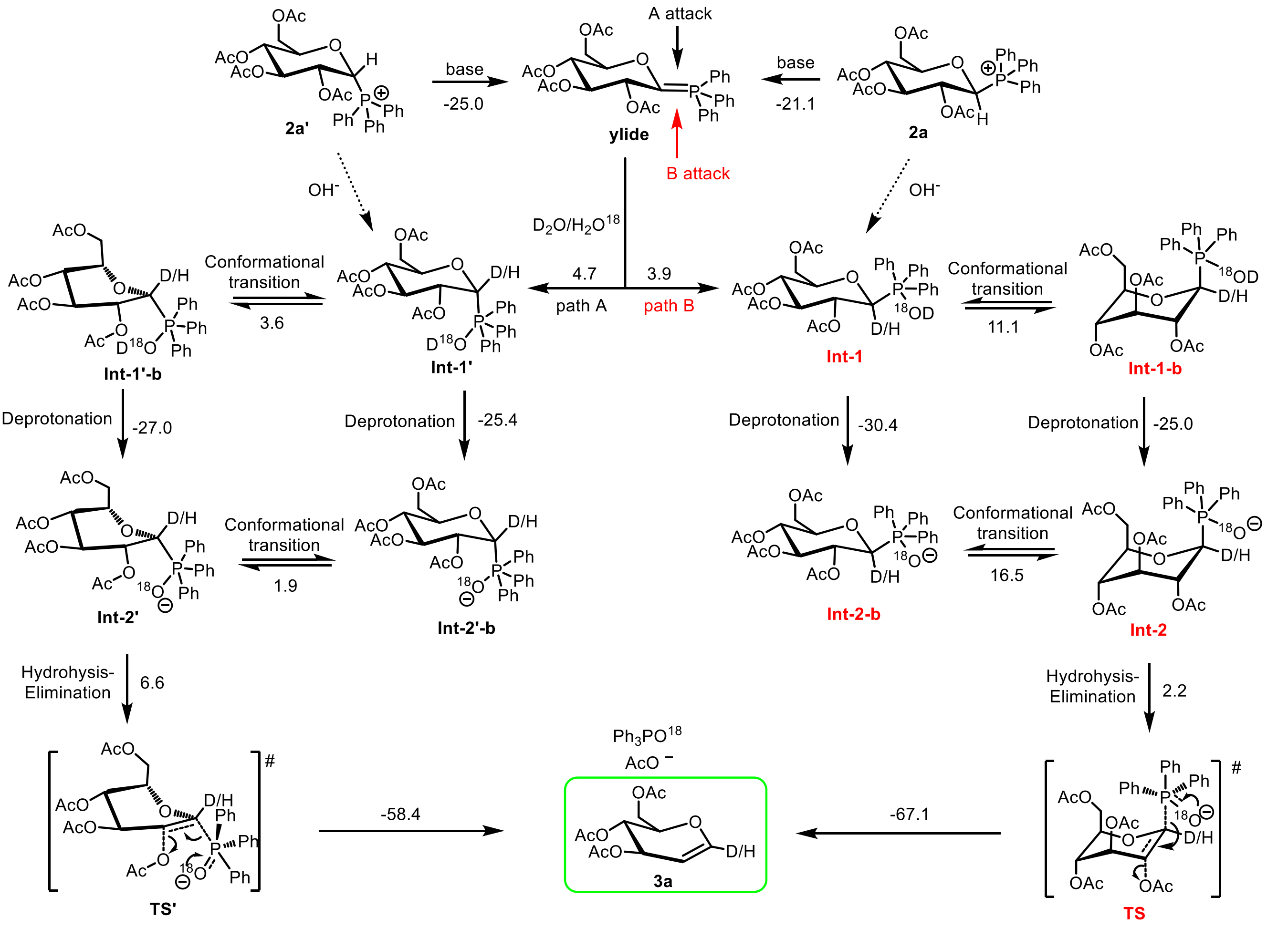

Scheme S5. The calculated reaction pathway by DFT at the m06-2x/6-311+G(d, p)/IEF-PCMDCM//b3lyp/6-31+G(d, p) level using 2a (glucose-triphenylphosphonium) as computational model substrate. A fast intermolecular addition reaction between the generated phosphorus-ylide and $H_2O/D_2O/H_2O^{18}$ molecule from two

different orientations (path a and path b) proceeded to give α/β-glucose-hydroxylphosphorane (**Int-1** and **Int-1′**). Subsequently, the intermediates undergoes conformational conversion and deprotonation to respectively deliver **INT-2** or **INT-2′**, where the several equilibrium reactions and intermediates (**Int-1-b**, **Int-2-b**, **Int-1′-b**, and **Int-2′-b**) were involved. Finally, the elimination reaction undergoes to give the final glycal through a transition state **TS** (the barrier of 2.2 kcal/mol) or **TS′** (the barrier of 6.6 kcal/mol).

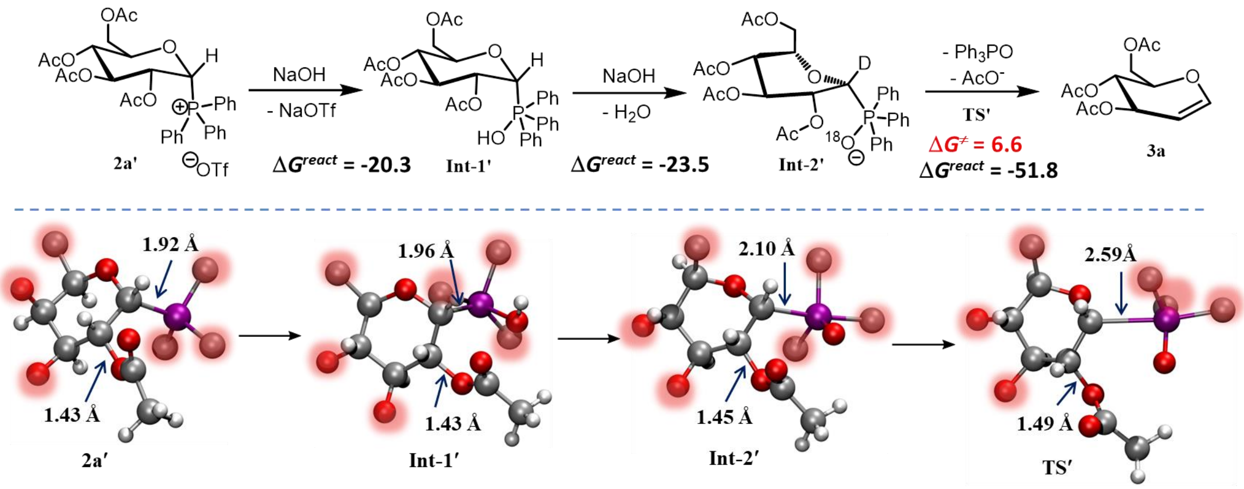

Fig. S6 The proposed reaction process and optimized structures for **2a′**, **Int-1′**, **Int-2′**, and **TS′**. The energy are shown in kcal mol^{-1}.

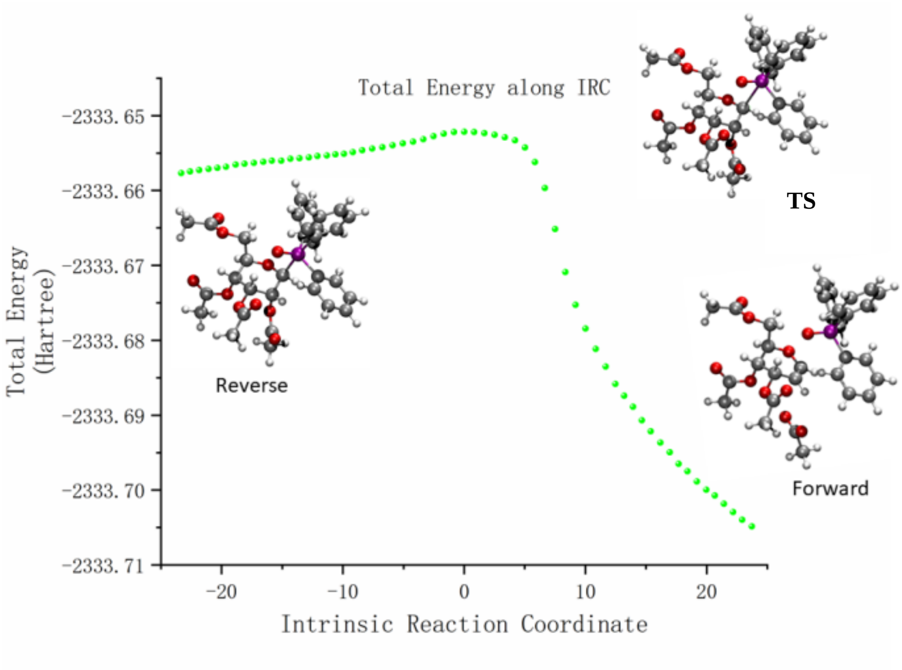

Fig. S7. The IRC plots of **TS** calculated at the b3lyp/6-31+G(d, p) level.

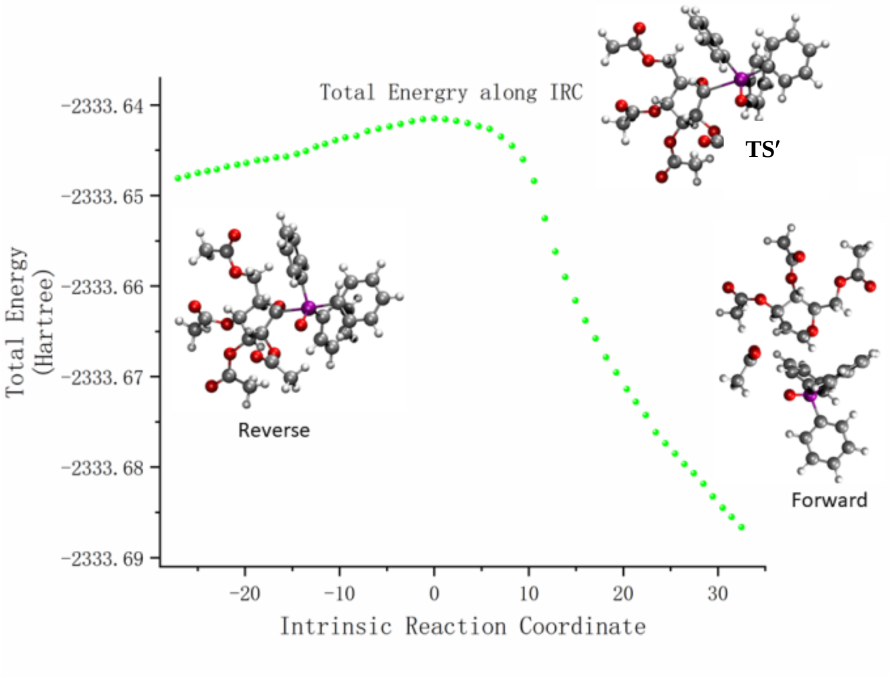

Fig. S8. The IRC plots of **TS′** calculated at the b3lyp/6-31+G(d, p) level.

Captions to Movies S1 and S2.

Animation of Reaction Coordinate (computational analysis) Move S1.

LOL analysis of TS. Localized orbital locator (LOL) analysis of transition state TS along its intrinsic reaction coordinate (IRC). This video show an axial P-C(glycosyl) bond cleavage and C=C bond formation with a nucleofugality leaving. The calculated methods see supporting computational details. The video was created using Multiwfn, VMD, and Windows Movie Maker.

Animation of Frontier Molecular Orbital for P-C Bond Cleavage and C=C Bond Forming Event (computational analysis) Move S2.

Orbital analysis of TS. Change in the HOMO involved in transition state TS along its intrinsic reaction coordinate (IRC). This video show the δ-electron heterolytic cleavage and π-electron formation with a nucleofugality leaving. The calculated methods see supporting computational details. The video was created using Multiwfn, VMD, and Windows Movie Maker.

5. General Procedure for the Synthesis of Glycals 3.

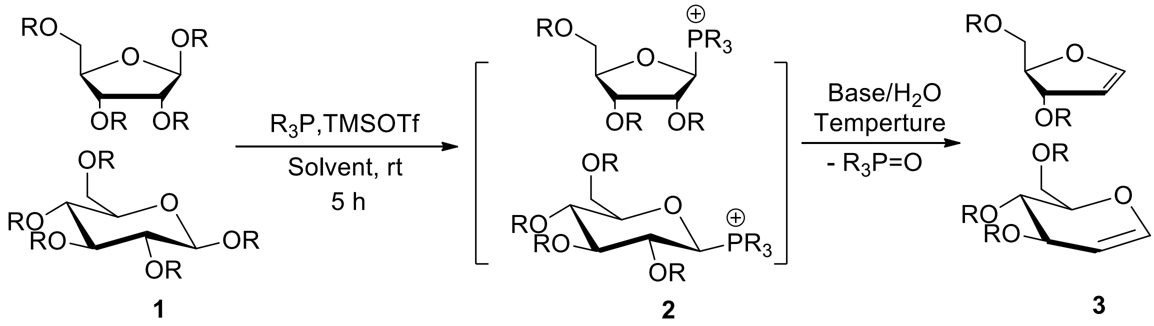

Compounds **1** (1 mmol) and triphenylphosphine (1.2 mmol) were dissolved in DCM (5 mL) in a Schlenk bottle under argon gas atmosphere, TMSOTf (1.1 mmol) was added under 0 °C. The mixture was stirred at room temperature for 5-6 h, and then corresponding phosphonium salts **2** were obtained. Without crude products not purified further, aqueous NaOH (1 M) was added directly at room temperature. After the reaction was completed, the water (10 mL) was added to the resulting mixture. The organic layer was separated, and then the aqueous layer was extracted with CH_2Cl_2 (10 mL × 2). All combined organic solutions were dried with anhydrous Na_2SO_4, and the solvent was removed under reduced pressure. The residue was column chromatography to afford the corresponding products **3**.

6. The Analytical and Spectral Characterization Data of Compounds 3.

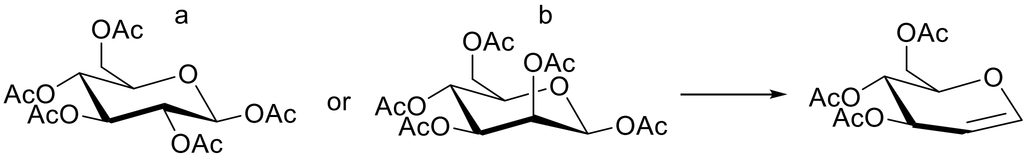

3a[1]**:** Pale yellow liquid; yield (85%[a], 90%[b]); ^{1}H NMR (400 MHz, Chloroform-*d*) δ 6.45 (d, *J* = 6.2 Hz, 1H), 5.33 – 5.31 (m, 1H), 5.22 – 5.19 (m, 1H), 4.83 (dd, *J* = 6.2, 3.2 Hz, 1H), 4.38 (dd, *J* = 12.0, 5.8 Hz, 1H), 4.26 – 4.22 (m, 1H), 4.18 (dd, *J* = 12.0, 3.0 Hz, 1H), 2.08 (s, 3H), 2.06 (s, 3H), 2.03 (s, 3H); ^{13}C NMR (100 MHz, Chloroform-*d*) δ 170.7 (s, C), 170.5 (s, C), 169.7 (s, C), 145.8 (s, CH), 99.1 (s, CH), 74.1 (s, CH), 67.6 (s, CH), 67.31(s, CH), 61.5 (s, CH_2), 21.1 (s, CH_3), 20.9 (s, CH_3), 20.8 (s, CH_3). HRMS Calcd. For $C_{12}H_{17}O_7$ $[M + H^+]^+$, 273.0969. Found: 273.0966.

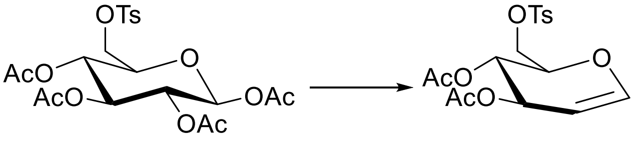

3b[1b]**:** Pale yellow liquid; yield (90%); ^{1}H NMR (400 MHz, Chloroform-*d*) δ 7.78 (d, *J* = 8.0 Hz, 2H), 7.34 (d, *J* = 8.0 Hz, 2H), 6.34 (d, *J* = 4.0 Hz, 1H), 5.25 (t, *J* = 4.0 Hz, 1H), 5.11 (t, *J* = 6.0 Hz, 1H), 4.80 (dd, *J* = 6.0, 3.4 Hz, 1H), 4.25 – 4.19 (m, 3H), 2.44 (s, 3H), 2.02 (s, 3H), 2.01 (s, 3H); ^{13}C NMR (100 MHz, Chloroform-*d*) δ 170.3 (s, C), 169.5 (s, C), 145.40 (s, CH), 145.2 (s, C), 132.6 (s, C) 123.0 (s, 2CH), 128.1 (s, 2CH), 99.0 (s, CH), 73.3 (s, CH), 67.1 (s, CH_2), 66.7 (s, CH), 66.5 (s, CH_2), 21.8 (s, CH_3), 21.0 (s, CH_3), 20.8 (s, CH_3). HRMS Calcd. For $C_{17}H_{21}O_8S$ $[M + H^+]^+$, 385.0952. Found: 385.0956.

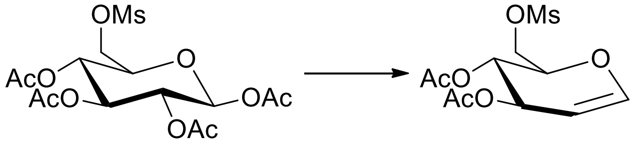

3c[6a]**:** Pale yellow liquid; yield (87%); ^{1}H NMR (400 MHz, Chloroform-*d*) δ 6.48 (d, *J* = 6.2 Hz, 1H), 5.35 (s, 1H), 5.23 – 5.20 (m, 1H), 4.89 (dd, *J* = 6.2, 3.4 Hz, 1H), 4.48 (dd, *J* = 11.4, 6.0 Hz, 1H), 4.37 – 4.33 (m, 2H), 3.07 (s, 3H), 2.10 (s, 3H), 2.06 (s, 3H); ^{13}C NMR (100 MHz, Chloroform-*d*) δ 169.8 (s, C), 169.1 (s, C), 144.8 (s, CH), 98.9 (s, CH), 73.1 (s, CH), 66.5 (s, CH_2), 66.4 (s, CH), 65.1 (s, CH), 37.4 (s, CH_3), 20.4 (s, CH_3), 20.3 (s, CH_3). HRMS Calcd. For $C_{11}H_{17}O_8S$ $[M + H^+]^+$, 309.0639. Found: 309.0640.

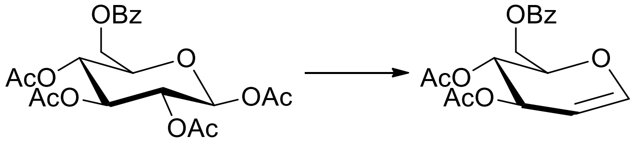

3d[4b]**:** Pale yellow liquid; yield (86%); ^{1}H NMR (400 MHz, Chloroform-*d*) δ 8.01 (d, *J* = 7.2 Hz, 2H), 7.59 (t, *J* = 7.4 Hz, 1H), 7.45 (t, *J* = 7.8 Hz, 2H), 6.52 (d, *J* = 6.0 Hz, 1H), 5.53 – 5.44 (m, 2H), 4.93 (dd, *J* = 5.0, 1.2 Hz, 1H), 4.44 – 4.36 (m, 2H), 4.29 (d, *J* = 8.8 Hz, 1H), 2.07 (s, 3H), 2.03 (s, 3H); ^{13}C NMR (100 MHz, Chloroform-*d*) δ 170.7 (s, C), 170.5 (s, C), 165.3 (s, C), 145.8 (s, CH), 133.7 (s, CH), 130.0 (s, 2CH), 129.3 (s, C), 128.7 (s, 2CH), 99.1 (s, CH), 74.1 (s, CH), 68.0 (s, CH), 67.3 (s, CH), 61.9 (s, CH_2), 21.1 (s, CH_3), 20.8 (s, CH_3). HRMS Calcd. For $C_{17}H_{19}O_7$ $[M + H^+]^+$, 335.1125. Found: 335.1123.

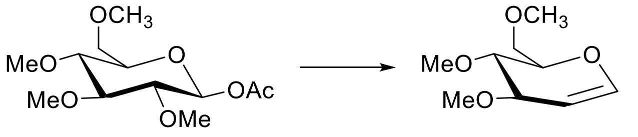

3e[4c]**:** Pale yellow liquid; yield (80%); ^{1}H NMR (400 MHz, Chloroform-*d*) δ 6.38 (d, *J* = 5.4 Hz, 1H), 4.82 (dd, *J* = 6.2, 2.8 Hz, 1H), 3.98 – 3.84 (m, 1H), 3.87 (d, *J* = 3.2 Hz, 1H), 3.69 – 3.61 (m, 2H), 3.53 (s, 3H), 3.45 (dd, *J* = 8.4, 6.2 Hz, 1H), 3.41 (s, 3H), 3.40 (s, 3H); ^{13}C NMR (100 MHz, Chloroform-*d*) δ 144.7 (s, CH), 99.7 (s, CH), 76.8 (s, CH), 76.4 (s, CH), 76.0 (s, CH), 71.0 (s, CH_2), 59.4 (s, CH_3), 59.4 (s, CH_3), 55.9 (s, CH_3). HRMS Calcd. For $C_9H_{17}O_4$ $[M + H^+]^+$, 189.1121. Found: 189.1125.

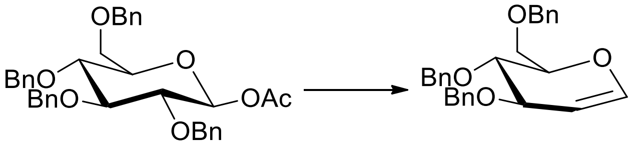

3f[1b]**:** Pale yellow liquid; yield (91%); ^{1}H NMR (400 MHz, Chloroform-*d*) δ 7.33 (d, *J* = 4.2 Hz, 9H), 7.26 (dd, *J* = 17.0, 6.0 Hz, 6H), 6.42 (d, *J* = 6.0 Hz, 1H), 4.88 (dd, *J* = 6.0, 2.4 Hz, 1H), 4.83 (d, *J* = 11.4 Hz, 1H), 4.65 (s, 1H), 4.62 (s, 1H), 4.56 (t, *J* = 8.8 Hz, 3H), 4.21 (d, *J* = 5.0 Hz, 1H), 4.09 – 4.03 (m, 1H), 3.89 – 3.75 (m, 3H); ^{13}C NMR (100 MHz, Chloroform-*d*) δ 144.9 (s, CH), 138.5 (s, C), 138.3 (s, C), 138.1 (s, C), 128.6 (s, 2CH), 128.5 (s, 2CH), 128.5 (s, 2CH), 128.1 (s, 2CH), 127.9 (s, 2CH), 127.9 (s, 3CH), 127.8 (s, 2CH), 100.1 (s, CH), 76.9 (s, CH), 75.9 (s, CH), 74.5 (s, CH), 73.9 (s, CH_2), 73.6 (s, CH_2), 70.6 (s, CH_2), 68.7 (s, CH_2). HRMS Calcd. For $C_{27}H_{29}O_4$ $[M + H^+]^+$, 417.2060. Found: 417.2063.

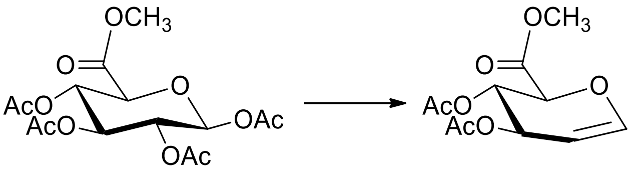

3g: Pale yellow liquid; yield (87%); ^{1}H NMR (400 MHz, Chloroform-*d*) δ 6.68 (d, *J* = 6.0 Hz, 1H), 5.41 (d, *J* = 1.4 Hz, 1H), 5.02 – 4.98 (m, 2H), 4.83 (s, 1H), 3.79 (s, 3H), 2.12 (s, 3H), 1.99 (s, 3H); ^{13}C NMR (100 MHz, Chloroform-*d*) δ 169.6 (s, C), 169.4 (s, C), 167.3 (s, C), 146.4 (s, CH), 97.3 (s, CH), 72.3 (s, CH), 67.4 (s, CH), 62.6 (s, CH), 52.4 (s, CH_3), 21.0 (s, CH_3), 20.9 (s, CH_3). HRMS Calcd. For $C_{11}H_{14}NaO_7$ $[M + Na^+]^+$, 281.0632. Found: 281.0635.

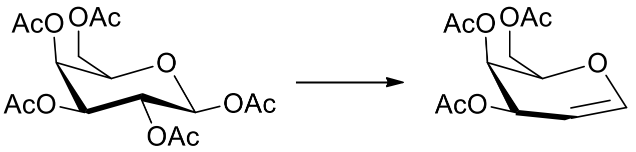

3h[1b-1d]**:** Pale yellow liquid; yield (84%); ^{1}H NMR (400 MHz, Chloroform-*d*) δ 6.46 (d, *J* = 6.4 Hz, 1H), 5.55 (s, 1H), 5.43 (d, *J* = 4.4 Hz, 1H), 4.73 (d, *J* = 5.4 Hz, 1H), 4.34 – 4.29 (m, 1H), 4.26 – 4.19 (m, 2H), 2.13 (s, 3H), 2.09 (s, 3H), 2.03 (s, 3H); ^{13}C NMR (100 MHz, Chloroform-*d*) δ 170.7 (s, C), 170.5 (s, C), 170.3 (s, C), 145.6 (s, CH), 99.0 (s, CH), 73.0 (s, CH), 64.0 (s, CH), 63.9 (s, CH), 62.1 (s, CH_2), 21.0 (s, CH_3), 20.9 (s, CH_3), 20.8 (s, CH_3). HRMS Calcd. For $C_{12}H_{17}O_7$ $[M + H^+]^+$, 273.0969. Found: 273.0970.

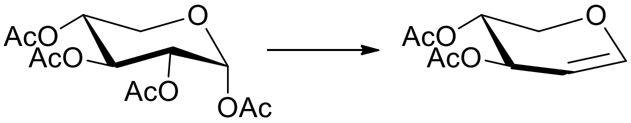

3i[1b]**:** Pale yellow liquid; yield (90%); ^{1}H NMR (400 MHz, Chloroform-*d*) δ 6.59 (d, *J* = 5.4 Hz, 1H), 4.98 – 4.93 (m, 3H), 4.19 (d, *J* = 12.2 Hz, 1H), 3.97 (d, *J* = 12.0 Hz, 1H), 2.09 (s, 3H), 2.06 (s, 3H); ^{13}C NMR (100 MHz, Chloroform-*d*) δ 170.1 (s, C), 169.9 (s, C), 148.2 (s, CH), 97.5 (s, CH), 67.3 (s, CH), 63.7 (s, CH_2), 63.5 (s, CH), 21.3 (s, CH_3), 21.1 (s, CH_3). HRMS Calcd. For $C_9H_{13}O_5$ $[M + H^+]^+$, 201.0757. Found: 201.0755.

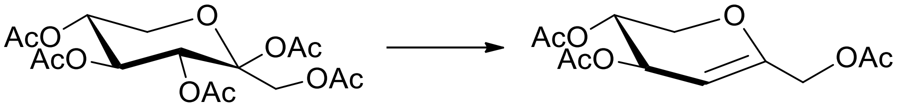

3j: Pale yellow liquid; yield (90%); ^{1}H NMR (400 MHz, Chloroform-*d*) δ 5.63 – 5.59 (m, 1H), 5.07 (t, *J* = 3.0 Hz, 1H), 4.49 (s, 1H), 4.38 – 4.33 (m, 1H), 4.27 (dd, *J* = 11.8, 4.8 Hz, 1H), 4.21 (d, *J* = 1.0 Hz, 1H), 4.15 (dd, *J* = 11.8, 6.6 Hz, 1H), 2.06 (s, 3H), 2.05 (s, 3H), 2.04 (s, 3H); ^{13}C NMR (100 MHz, Chloroform-*d*) δ 170.5 (s, C), 169.9 (s, C), 169.6 (s, C), 158.2 (s, C), 87.3 (s, CH_2), 81.9 (s, CH), 76.2 (s, CH), 74.8 (s, CH), 62.9 (s, CH_2), 20.9 (s, CH_3), 20.7 (s, 2CH_3). HRMS Calcd. For $C_{12}H_{17}O_7$ $[M + H^+]^+$, 273.0969. Found: 273.0965.

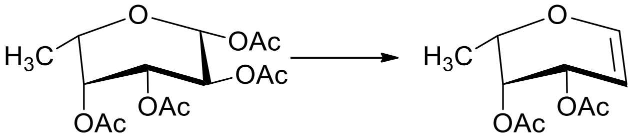

3k[4e]**:** Pale yellow liquid; yield (94%); ^{1}H NMR (400 MHz, Chloroform-*d*) δ 6.46 (d, *J* = 6.4 Hz, 1H), 5.59 – 5.54 (m, 1H), 5.28 (d, *J* = 4.4 Hz, 1H), 4.63 (d, *J* = 6.4 Hz, 1H), 4.20 (q, *J* = 6.6 Hz, 1H), 2.15 (s, 3H), 2.01 (s, 3H), 1.27 (s, 1H), 1.26 (s, 1H), 1.24 (s, 1H); ^{13}C NMR (100 MHz, Chloroform-*d*) δ 170.8 (s, C), 170.5 (s, C), 146.3 (s, CH), 98.4 (s,

CH), 71.7 (s, CH), 66.5 (s, CH), 65.2 (s, CH), 21.0 (s, CH_3), 20.8 (s, CH_3), 16.6 (s, CH_3). HRMS Calcd. For $C_{10}H_{15}O_5$ $[M+H^+]^+$, 215.0914. Found: 215.0915.

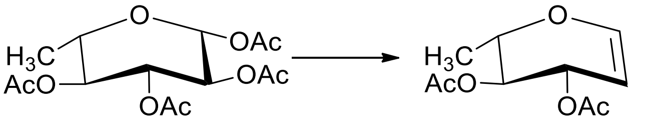

3l[1b]**:** Pale yellow liquid; yield (92%); ^{1}H NMR (400 MHz, Chloroform-*d*) δ 6.41 (d, *J* = 5.4 Hz, 1H), 5.34 – 5.29 (m, 1H), 5.01 (dd, *J* = 8.0, 6.2 Hz, 1H), 4.76 (dd, *J* = 6.2, 3.0 Hz, 1H), 4.13 – 4.04 (m, 1H), 2.07 (s, 3H), 2.03 (s, 3H), 1.29 (d, *J* = 6.6 Hz, 3H); ^{13}C NMR (100 MHz, Chloroform-*d*) δ 170.8 (s, C), 170.0 (s, C), 146.1 (s, CH), 98.9 (s, CH), 72.6 (s, CH), 72.0 (s, CH), 68.4 (s, CH), 21.2 (s, CH_3), 21.0 (s, CH_3), 16.7 (s, CH_3). HRMS Calcd. For $C_{10}H_{15}O_5$ $[M+H^+]^+$, 215.0914. Found: 215.0920.

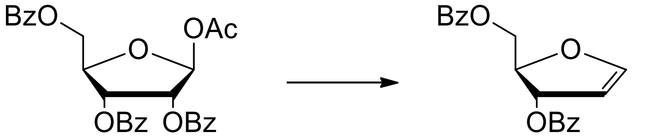

3m: Pale yellow liquid; yield (85%); ^{1}H NMR (400 MHz, Chloroform-*d*) δ 8.08 – 8.03 (m, 4H), 7.57 (dd, *J* = 8.2, 6.8 Hz, 2H), 7.44 (t, *J* = 7.8 Hz, 4H), 6.74 (d, *J* = 2.2 Hz, 1H), 5.99 (s, 1H), 5.34 (t, *J* = 2.6 Hz, 1H), 4.94 – 4.89 (m, 1H), 4.62 (dd, *J* = 11.8, 4.2 Hz, 1H), 4.56 (dd, *J* = 11.8, 6.2 Hz, 1H); ^{13}C NMR (100 MHz, Chloroform-*d*) δ 166.5 (s, C), 166.3 (s, C), 152.2 (s, CH), 133.3 (d, *J* = 5.0 Hz, 2C), 129.8 (d, *J* = 9.7 Hz, 5CH), 128.4(s, 5CH), 99.6 (s, CH), 83.8 (s, CH), 79.3 (s, CH), 64.1 (s, CH_2). HRMS Calcd. For $C_{19}H_{17}O_5$ $[M+H^+]^+$, 325.1071. Found: 325.1069.

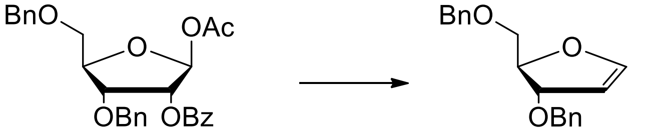

3n[11]**:** Pale yellow liquid; yield (90%); ^{1}H NMR (400 MHz, Chloroform-*d*) δ 7.35 (dd, *J* = 9.2, 5.8 Hz, 10H), 6.61 (d, *J* = 2.4 Hz, 1H), 5.19 (s, 1H), 4.66 (t, *J* = 4.6 Hz, 2H), 4.59 (d, *J* = 7.4 Hz, 2H), 4.53 (s, 2H), 3.56 (dd, *J* = 9.8, 6.4 Hz, 1H), 3.43 (dd, *J* = 10.2, 5.2 Hz, 1H); ^{13}C NMR (100 MHz, Chloroform-*d*) δ 150.5 (s, CH), 138.3 (s, C), 137.9 (s, C), 128.5 (s, 4CH), 128.0 (s, 2CH), 127.8 (t, J = 5.8 Hz, 4CH), 100.7 (s, CH), 84.9 (s, CH), 82.7 (s, CH), 73.5 (s, CH_2), 69.9 (s, CH_2), 69.7 (s, CH_2). HRMS Calcd. For $C_{19}H_{21}O_3$ $[M+H^+]^+$, 297.1485. Found: 297.1480.

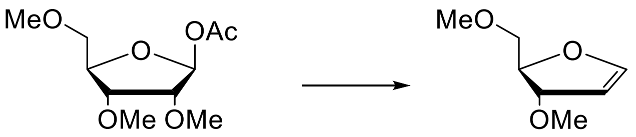

3o: Pale yellow liquid; yield (90%); ^{1}H NMR (400 MHz, Chloroform-*d*) δ 6.55 (d, *J* = 1.8 Hz, 1H), 5.15 (s, 1H), 4.49 (s, 1H), 4.41 (s, 1H), 3.46 (dd, *J* = 10.2, 6.8 Hz, 1H), 3.39 (s, 3H), 3.36 (d, *J* = 5.4 Hz, 1H), 3.27 (s, 3H). ^{13}C NMR (100 MHz, Chloroform-*d*) δ 149.9 (s, CH), 99.6 (s, CH), 83.7 (s, CH), 83.7 (s, CH), 72.3 (s, CH_2), 58.8 (s, CH_3), 54.1 (s, CH_3). HRMS Calcd. For $C_7H_{13}O_3$ $[M+H^+]^+$, 145.0859. Found: 145.0860.

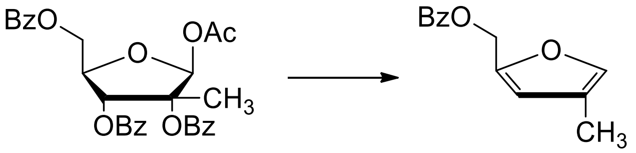

4p: Pale yellow liquid; yield (85%); ^{1}H NMR (400 MHz, Chloroform-*d*) δ 7.94 – 7.91 (m, 2H), 7.40 (t, *J* = 7.4 Hz, 1H), 7.29 (d, *J* = 7.8 Hz, 2H), 7.07 (s, 1H), 6.22 (s, 1H), 5.13 (s, 2H), 1.89 (d, *J* = 0.7 Hz, 3H); ^{13}C NMR (100 MHz, Chloroform-*d*) δ 166.3 (s, C), 149.6 (s, C), 140.0 (s, CH), 133.1 (s, CH), 130.1 (s, C), 129.8 (s, 2CH), 128.4 (s, 2CH), 121.0 (s, C), 113.5 (s, CH), 58.8 (s, CH_2), 9.7 (s, CH_3). HRMS Calcd. For $C_{13}H_{13}O_3$ $[M+H^+]^+$, 217.0859. Found: 217.0858.

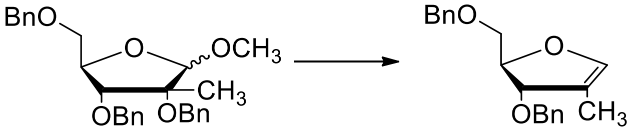

3q: Pale yellow liquid; yield (89%); ^{1}H NMR (400 MHz, Chloroform-*d*) δ 7.38 – 7.32 (m, 10H), 6.28 (s, 1H), 4.64 – 4.56 (m, 4H), 4.49 (d, *J* = 12.4 Hz, 2H), 3.56 (dd, *J* = 10.0, 6.4 Hz, 1H), 3.40 (dd, *J* = 10.0, 6.4 Hz, 1H), 1.72 (s, 3H); ^{13}C NMR (100 MHz, Chloroform-*d*) δ 144.3 (s, CH), 138.4 (d, *J* = 53.8 Hz, C), 133.8 (d, *J* = 19.4 Hz, C), 128.5 (d, *J* = 5.2 Hz, 4CH), 127.9 (s, 2CH), 127.8 (s, 2CH), 127.7 (s, 2CH), 109.9 (s, CH), 85.9 (s, CH), 84.5 (s, CH), 73.6 (s, CH_2), 70.3 (s, CH_2), 69.7 (s, CH_2), 9.0 (s, CH_3). HRMS Calcd. For $C_{20}H_{23}O_3$ $[M+H^+]^+$, 311.1642. Found: 311.1645.

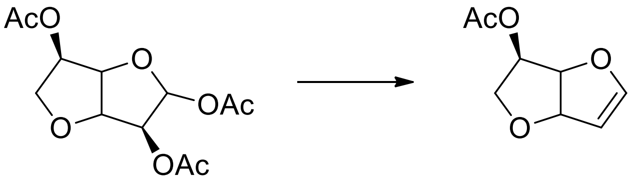

3r: Pale yellow liquid; yield (81%); ^{1}H NMR (400 MHz, Chloroform-*d*) δ 6.28 (d, *J* = 5.4 Hz, 1H), 5.43 (s, 1H), 5.03 (t, *J* = 5.8 Hz, 1H), 4.53 (s, 1H), 4.32 (t, *J* = 8.6 Hz, 2H), 4.17 (dd, *J* = 10.6, 3.8 Hz, 1H), 2.11 (s, 3H); ^{13}C NMR (100 MHz, Chloroform-*d*) 170.3 (s, C), 145.3 (s, CH), 102.2 (s, CH), 77.4 (s, CH), 74.3 (s, CH_2), 74.2 (s, CH), 72.1 (s, CH), 21.1 (s, CH_3). HRMS Calcd. For $C_8H_{11}O_4$ $[M + H^+]^+$, 171.0652. Found: 171.0650.

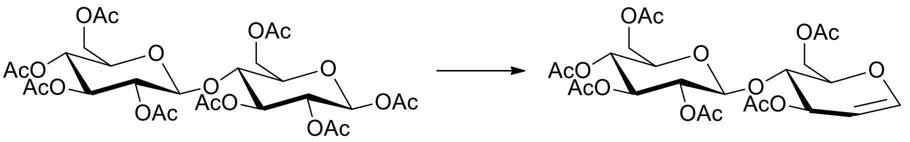

3s[6a]**:** Pale yellow liquid; yield (87%); ^{1}H NMR (400 MHz, Chloroform-*d*) δ 6.40 (d, *J* = 6.0 Hz, 1H), 5.41 (s, 1H), 5.18 (t, *J* = 9.4 Hz, 1H), 5.08 (t, *J* = 9.6 Hz, 1H), 4.97 (t, *J* = 8.8 Hz, 1H), 4.82 (dd, *J* = 6.0, 3.2 Hz, 1H), 4.68 (d, *J* = 8.0 Hz, 1H), 4.44 (d, *J* = 11.4 Hz, 1H), 4.31 (dd, *J* = 12.4, 4.4 Hz, 1H), 4.21 – 4.11 (m, 2H), 4.05 (d, *J* = 12.4 Hz, 1H), 3.98 (t, 1H), 3.67 (d, 1H), 2.12 (s, 3H), 2.09 (s, 3H), 2.04 (s, 6H), 2.01 (s, 3H), 1.99 (s, 3H); ^{13}C NMR (100 MHz, Chloroform-*d*) δ 170.8 (s, C), 170.6 (s, C), 170.4 (s, C), 170.1 (s, C), 169.4 (s, C), 169.3 (s, C), 145.6 (s, CH), 100.7 (s, CH), 99.2 (s, CH), 74.8 (s, CH), 74.5 (s, CH), 72.9 (s, CH), 72.1 (s, CH), 71.5 (s, CH), 68.7 (s, CH), 68.2 (s, CH), 61.9 (s, CH_2), 61.9 (s, CH_2), 21.1 (s, CH_3), 21.0 (s, CH_3), 20.8 (s, CH_3), 20.7 (s, 2CH_3) , 20.7 (s, CH_3). HRMS Calcd. For $C_{24}H_{32}NaO_{15}$ $[M + Na^+]^+$, 583.1633. Found: 583.1636.

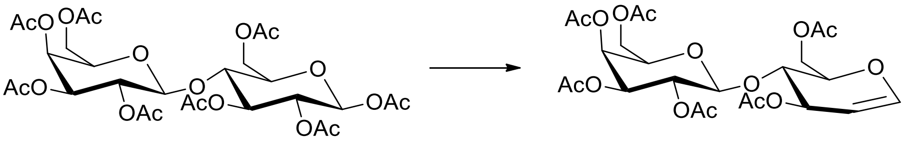

3t[6a]**:** Pale yellow liquid; yield (89%); ^{1}H NMR (400 MHz, Chloroform-*d*) δ 6.40 (d, *J* = 6.0 Hz, 1H), 5.42 – 5.38 (m, 1H), 5.36 (d, *J* = 2.8 Hz, 1H), 5.18 (dd, *J* = 10.4, 8.0 Hz, 1H), 4.99 (dd, *J* = 10.6, 3.4 Hz, 1H), 4.83 (dd, *J* = 6.0, 3.4 Hz, 1H), 4.65 (d, *J* = 8.0 Hz, 1H), 4.43 (dd, *J* = 11.4, 2.0 Hz, 1H), 4.22 – 4.11 (m, 3H), 4.07 (dd, *J* = 11.2, 7.4 Hz, 1H), 3.99 (dd, *J* = 7.0, 5.8 Hz, 1H), 3.90 (t, *J* = 6.8 Hz, 1H), 2.15 (s, 3H), 2.11 (s, 3H), 2.08 (s, 3H), 2.05 (d, *J* = 4.0 Hz, 6H), 1.97 (s, 3H); ^{13}C NMR (100 MHz, Chloroform-*d*) δ 170.6 (s, C), 170.5 (s, C), 170.3 (s, C), 170.2 (s, C), 170.1 (s, C), 169.4 (s, C), 145.6 (s, CH), 101.2 (s, CH), 99.1 (s, CH), 74.8 (s, CH), 74.3 (s, CH), 71.0 (s, CH), 70.9 (s, CH), 69.0 (s, CH), 68.9 (s, CH), 66.9 (s, CH), 62.0 (s, CH), 61.1 (s, CH), 21.2 (s, CH_3), 21.0

(s, CH_3), 20.8 (s, CH_3), 20.7 (s, CH_3), 20.7 (s, CH_3), 20.6 (s, CH_3). HRMS Calcd. For $C_{24}H_{33}O_{15}$ $[M + H^{+}]^{+}$, 561.1814. Found: 561.1815.

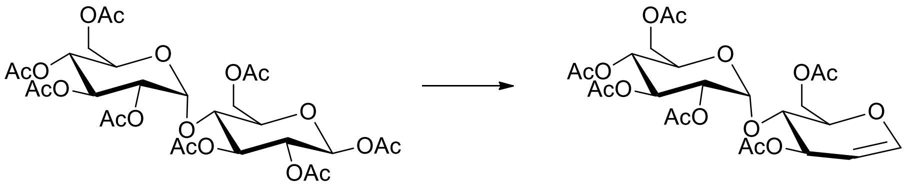

3u[4e]**:** Pale yellow liquid; yield (90%); ^{1}H NMR (400 MHz, Chloroform-*d*) δ 6.43 (d, *J* = 6.2 Hz, 1H), 5.49 (d, *J* = 3.8 Hz, 1H), 5.40 (t, *J* = 10.0 Hz, 1H), 5.16 (t, *J* = 3.8 Hz, 1H), 5.04 (t, *J* = 10.0 Hz, 1H), 4.82 (dd, *J* = 10.2, 4.2 Hz, 2H), 4.35 (t, *J* = 4.6 Hz, 2H), 4.31 – 4.27 (m, 1H), 4.23 (dd, *J* = 12.4, 4.2 Hz, 1H), 4.11 – 3.99 (m, 3H), 2.11 (s, 3H), 2.09 (s, 3H), 2.04 (d, *J* = 2.2 Hz, 6H), 2.02 (s, 3H), 2.00 (s, 3H); ^{13}C NMR (100 MHz, Chloroform-*d*) δ 170.7 (s, C), 170.6 (s, C), 170.5 (s, C), 170.4 (s, C), 170.1 (s, C), 169.7 (s, C), 145.7 (s, CH), 98.7 (s, CH), 96.0 (s, CH), 74.3 (s, CH), 72.7 (s, CH), 70.6 (s, CH), 69.8 (s, CH), 69.6 (s, CH), 68.4 (s, CH), 68.4 (s, CH), 62.0 (s, CH), 61.8 (s, CH), 21.2 (s, CH_3), 20.9 (s, CH_3), 20.8 (d, *J* = 1.6 Hz, $2CH_3$), 20.7 (s, CH_3), 20.7 (s, CH_3). HRMS Calcd. For $C_{24}H_{33}O_{15}$ $[M + H^{+}]^{+}$, 561.1814. Found: 561.1812.

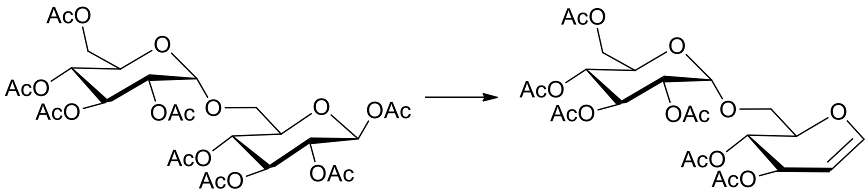

3v: Pale yellow liquid; yield (80%);^{1}H NMR (400 MHz, Chloroform-*d*) δ 6.40 (d, *J* = 6.0 Hz, 1H), 5.38 (s, 1H), 5.29 – 5.24 (m, 1H), 5.18 (s, 1H), 5.11 (dd, *J* = 14.6, 4.4 Hz, 2H), 5.02 (dd, *J* = 10.8, 3.4 Hz, 1H), 4.82 – 4.75 (m, 1H), 4.19 (s, 2H), 4.01 (d, *J* = 6.4 Hz, 2H), 3.78 (dd, *J* = 11.0, 6.2 Hz, 1H), 3.61 (dd, *J* = 11.2, 4.0 Hz, 1H), 2.06 (s, 3H), 2.02 (d, *J* = 2.8 Hz, 6H), 1.96 (s, 6H), 1.90 (s, 3H); ^{13}C NMR (100 MHz, Chloroform-*d*) δ 170.5 (s, C), 170.3 (s, C), 170.2 (s, C), 170.1 (s, C), 169.9 (s, C), 169.5 (s, C), 145.6 (s, CH), 98.4 (s, CH), 96.3 (s, CH), 74.5 (s, CH), 68.0 (s, 2CH), 67.4 (s, 2CH), 66.6 (s, CH), 66.4 (s, CH), 65.5 (s, CH_2), 61.7 (s, CH_2), 20.9 (s, CH_3), 20.8 (s, CH_3), 20.7 (d, *J* = 3.8 Hz, $2CH_3$), 20.6 (d, *J* = 2.3 Hz, $2CH_3$). HRMS Calcd. For $C_{24}H_{33}O_{15}$ $[M + H^{+}]^{+}$, 561.1814. Found: 561.1817.

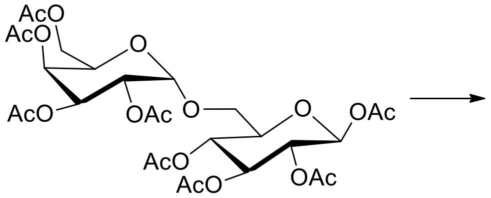

3w: White solid, yield (85%); ^{1}H NMR (400 MHz, Chloroform-*d*) δ 6.42 (d, *J* = 6.2 Hz, 1H), 5.40 (d, *J* = 2.6 Hz, 1H), 5.29 (dd, *J* = 10.8, 3.2 Hz, 1H), 5.19 (d, *J* = 3.8 Hz, 1H), 5.16 – 5.12 (m, 1H), 5.11 (d, *J* = 3.6 Hz, 1H), 5.05 (dd, *J* = 10.8, 3.6 Hz, 1H), 4.79 (dd, *J* = 6.0, 3.8 Hz, 1H), 4.20 (d, *J* = 6.4 Hz, 2H), 4.03 (d, *J* = 6.6 Hz, 2H), 3.81 (dd, *J* = 11.2, 6.1 Hz, 1H), 3.63 (dd, *J* = 11.2, 4.2 Hz, 1H), 2.08 (s, 3H), 2.05 (s, 3H), 2.04 (s, 3H), 1.98 (s, 6H), 1.92 (s, 3H); ^{13}C NMR (100 MHz, Chloroform-*d*) δ 170.6 (s, C), 170.3 (s, C), 170.2 (s, C), 170.2 (s, C), 169.9 (s, C), 169.5 (s, C), 145.7 (s, CH), 98.5 (s, CH), 96.3 (s, CH), 74.5 (s, CH), 68.0 (s, 2CH), 67.5 (d, *J* = 2.2 Hz, 2CH), 66.6 (s, CH), 66.4 (s, CH), 65.6 (s, CH_2), 61.7 (s, CH_2), 21.0 (s, CH_3), 20.8 (s, CH_3), 20.7 (s, CH_3), 20.7 (s, CH_3), 20.6 (s, CH_3), 20.6 (s, CH_3). HRMS Calcd. For $C_{24}H_{33}O_{15}$ $[M + H^+]^+$, 561.1814. Found: 561.1817.

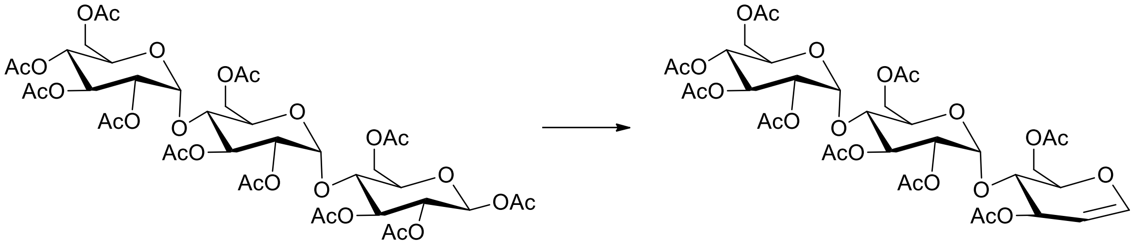

3x[5]: Pale yellow liquid; yield (85%); ^{1}H NMR (400 MHz, Chloroform-*d*) δ 6.43 (d, *J* = 6.2 Hz, 1H), 5.45 – 5.40 (m, 1H), 5.39 – 5.31 (m, 3H), 5.20 – 5.16 (m, 1H), 5.05 (t, *J* = 10.0 Hz, 1H), 4.84 (dd, *J* = 10.6, 4.0 Hz, 1H), 4.79 (dd, *J* = 6.2, 3.4 Hz, 1H), 4.68 (dd, *J* = 10.4, 4.0 Hz, 1H), 4.48 (dd, *J* = 12.4, 2.2 Hz, 1H), 4.37 (d, *J* = 4.6 Hz, 2H), 4.30 – 4.25 (m, 1H), 4.23 (dd, *J* = 12.6, 3.4 Hz, 1H), 4.17 (dd, *J* = 12.4, 3.4 Hz, 1H), 4.06 – 4.02 (m, 1H), 4.01 (d, *J* = 2.0 Hz, 1H), 3.99 (d, *J* = 9.8 Hz, 1H), 3.96 – 3.90 (m, 2H), 2.13 (s, 3H), 2.13 (s, 3H), 2.07 (s, 3H), 2.04 (s, 3H), 2.01 (s, 3H), 2.00 (s, 3H), 1.99 (s, 3H), 1.99 (s, 3H), 1.98 (s, 3H); ^{13}C NMR (100 MHz, Chloroform-*d*) δ 170.7 (s, C), 170.6 (s, 2C), 170.6 (s, C), 170.5 (s, 2C), 170.0 (s, C), 169.9 (s, C), 169.6 (s, C), 145.8 (s, CH), 98.7 (s, CH), 95.8 (s, CH), 95.8 (s, CH), 74.2 (s, CH), 72.8 (s, CH), 72.6 (s, CH), 72.0 (s, CH), 71.0 (s, CH), 70.1 (s, CH), 69.9 (s, CH), 69.4 (s, CH), 68.8 (s, CH), 68.6 (s, CH), 68.0 (s,

CH), 62.5 (s, CH_2), 62.1 (s, CH_2), 61.5 (s, CH_2), 21.2 (s, CH_3), 21.0 (s, CH_3), 20.9 (s, $2CH_3$), 20.8 (s, CH_3), 20.7 (s, $3CH_3$), 20.6 (s, CH_3). HRMS Calcd. For $C_{36}H_{49}O_{23}$ $[M+H^+]^+$, 848.2659. Found: 842.2660.

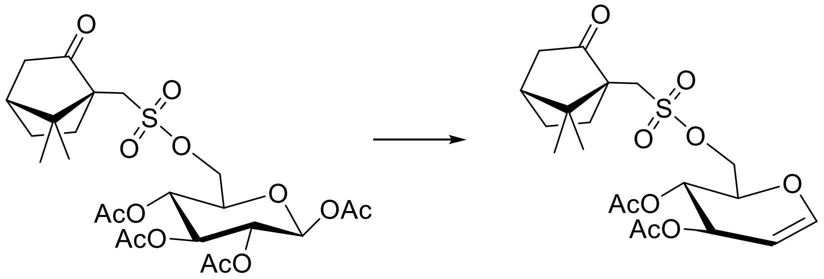

3y: White solid; yield (86%); ^{1}H NMR (400 MHz, Chloroform-*d*) δ 6.44 (dd, *J* = 6.2, 1.2 Hz, 1H), 5.51 – 5.47 (m, 1H), 5.05 (dd, *J* = 8.4, 6.4 Hz, 1H), 4.83 (dd, *J* = 6.2, 3.0 Hz, 1H), 4.49 (dd, *J* = 12.4, 5.0 Hz, 1H), 4.28 (ddd, *J* = 13.6, 7.0, 3.0 Hz, 2H), 3.69 (d, *J* = 14.8 Hz, 1H), 3.04 (d, *J* = 14.8 Hz, 1H), 2.47 – 2.33 (m, 2H), 2.12 (s, 3H), 2.10 (s, 3H), 2.08 (s, 1H), 2.08 – 2.00 (m, 1H), 1.93 (d, *J* = 18.6 Hz, 1H), 1.64 (ddd, *J* = 14.0, 9.4, 4.6 Hz, 1H), 1.43 (ddd, *J* = 13.0, 9.4, 3.8 Hz, 1H), 1.09 (s, 3H), 0.86 (s, 3H); ^{13}C NMR (100 MHz, Chloroform-*d*) δ 214.2 (s, C), 170.7 (s, C), 170.6 (s, C), 145.8 (s, CH), 98.9 (s, CH), 74.1 (s, CH), 73.2 (s, CH), 68.1 (s, CH), 61.3 (s, CH_2), 58.0 (s, C), 48.6 (s, CH_2), 48.1 (s, CH), 42.8 (s, CH), 42.5 (s, CH_2), 27.0 (s, CH_2), 24.9 (s, CH_2), 21.1 (s, CH_3), 20.9 (s, CH_3), 19.8 (s, CH_3), 19.7 (s, CH_3). HRMS Calcd. For $C_{20}H_{29}O_9S$ $[M+H^+]^+$, 445.1527. Found: 445.1530.

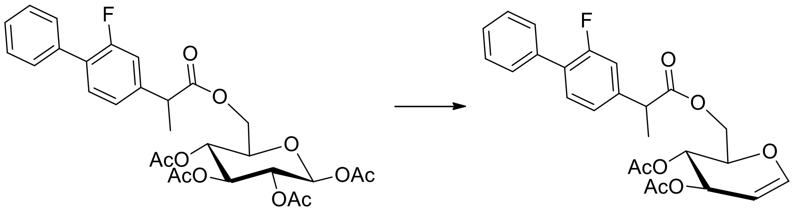

3z: Pale yellow liquid; yield (80%); ^{1}H NMR (400 MHz, Chloroform-*d*) δ 7.55 – 7.51 (m, 2H), 7.42 (t, *J* = 7.6 Hz, 2H), 7.39 – 7.30 (m, 2H), 7.10 (dd, *J* = 12.0, 6.2 Hz, 2H), 6.45 – 6.40 (m, 1H), 5.40 (t, *J* = 18.8 Hz, 1H), 5.30 – 5.25 (m, 1H), 4.83 – 4.75 (m, 1H), 4.41 – 4.19 (m, 1H), 4.19 – 4.12 (m, 1H), 4.09 – 3.97 (m, 1H), 3.76 (q, *J* = 7.2 Hz, 1H), 2.10 – 2.03 (m, 2H), 2.00 (d, *J* = 6.0 Hz, 3H), 1.85 (s, 1H), 1.53 (dd, *J* = 7.2, 2.6 Hz, 3H); ^{13}C NMR (100 MHz, Chloroform-*d*) δ 172.5 (s, C), 170.4 (s, C), 170.3 (s, C), 160.7 (d, *J* = 248.8 Hz, C), 145.7 (s, CH), 141.0 (s, C), 135.3 (s, C), 131.0 (s, CH), 129.0 (s, 2CH), 128.5 (s, 2CH), 128.2 (s, CH), 127.8 (s, CH), 123.4 (s, CH), 115.0 (s, CH), 99.2 (s, CH),

73.9 (s, CH), 68.1 (s, CH), 67.7 (s, CH), 61.4 (s, CH_2), 44.9 (s, CH), 20.9 (s, CH_3), 20.6 (s, CH_3), 17.9 (s, CH_3). HRMS Calcd. For $C_{25}H_{26}FO_7$ $[M + H^+]^+$, 457.1657. Found: 457.1655.

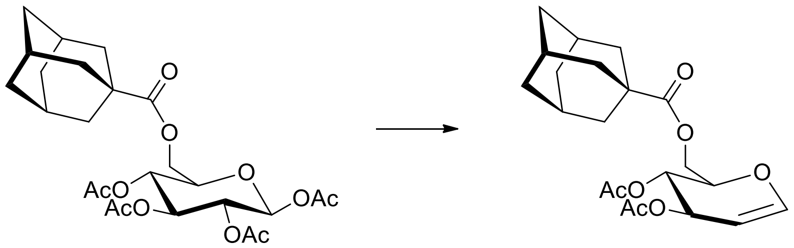

3aa: White solid; yield (86%); ^{1}H NMR (400 MHz, Chloroform-*d*) δ 6.44 (d, *J* = 6.2 Hz, 1H), 5.44 – 5.35 (m, 1H), 5.22 (dd, *J* = 8.2, 6.4 Hz, 1H), 4.80 (dd, *J* = 6.2, 3.0 Hz, 1H), 4.31 (dd, *J* = 12.0, 5.6 Hz, 1H), 4.24 – 4.12 (m, 2H), 2.07 (s, 3H), 2.01 (s, 3H), 1.98 (s, 3H), 1.83 (s, 6H), 1.69 (s, 6H); ^{13}C NMR (100 MHz, Chloroform-*d*) δ 176.1 (s, C), 170.7 (s, C), 170.5 (s, C), 145.7 (s, CH), 99.4 (s, CH), 74.3 (s, CH), 67.9 (s, CH), 66.6 (s, CH), 61.6 (s, CH_2), 40.8 (s, C), 38.6 (s, CH_2), 36.4 (s, CH_2), 27.8 (s, CH), 21.1 (s, CH_3), 20.8 (s, CH_3). HRMS Calcd. For $C_{21}H_{29}O_7$ $[M + H^+]^+$, 393.1908. Found: 393.1906.

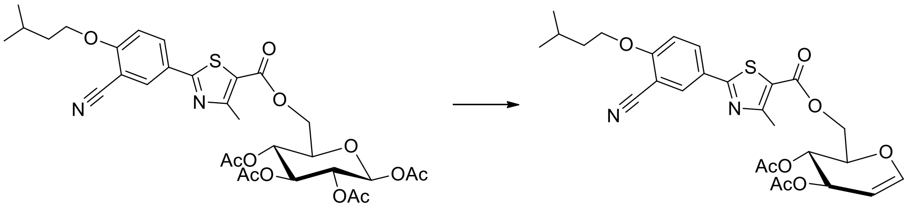

3ab: White solid; yield (82 %); ^{1}H NMR (400 MHz, Chloroform-*d*) δ 8.13 (t, *J* = 3.6 Hz, 1H), 8.03 (dd, *J* = 8.8, 2.2 Hz, 1H), 6.98 (d, *J* = 9.0 Hz, 1H), 6.49 (d, *J* = 6.2 Hz, 1H), 5.39 (dt, *J* = 11.8, 5.2 Hz, 2H), 4.88 (dd, *J* = 6.2, 3.4 Hz, 1H), 4.52 – 4.34 (m, 2H), 4.29 – 4.17 (m, 1H), 3.86 (d, *J* = 6.6 Hz, 2H), 2.69 (s, 3H), 2.16 (dt, *J* = 20.0, 6.6 Hz, 1H), 2.06 (d, *J* = 6.0 Hz, 3H), 2.05 – 2.00 (m, 3H), 1.04 (d, *J* = 6.8 Hz, 6H); ^{13}C NMR (100 MHz, Chloroform-*d*) δ 170.5 (s, C), 170.3 (s, C), 168.1 (s, C), 162.6 (s, C), 162.5 (s, C), 160.5 (s, C), 145.7 (s, CH), 132.7 (s, CH), 132.1 (s, CH), 125.7 (s, C), 120.4 (s, C), 115.3 (s, C), 112.7 (s, CH), 103.0 (s, C), 98.8 (s, CH), 75.7 (s, CH_2), 73.8 (s, CH), 68.1 (s, CH), 66.8 (s, CH), 61.4 (s, CH_2), 28.1 (s, CH_3), 21.0 (s, CH_3), 20.7 (s, CH_3), 19.0 (s, CH_3), 17.6 (s, CH_3). HRMS Calcd. For $C_{27}H_{31}N_2O_8$ $[M + H^+]^+$, 543.1796. Found: 543.1795.

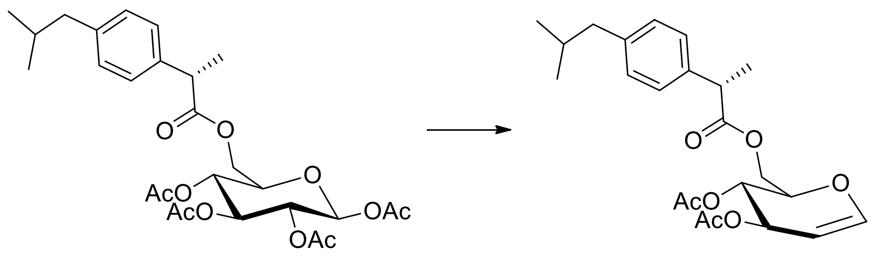

3ac: White solid; yield (81 %); ^{1}H NMR (400 MHz, Chloroform-*d*) δ 7.14 (d, *J* = 8.2 Hz, 2H), 7.06 (d, *J* = 8.0 Hz, 2H), 6.42 – 6.37 (m, 1H), 5.42 – 5.35 (m, 1H), 5.23 (dd, *J* = 8.8, 6.6 Hz, 1H), 4.77 (dd, *J* = 6.2, 3.0 Hz, 1H), 4.12 – 4.05 (m, 1H), 4.03 (dd, *J* = 12.4, 2.6 Hz, 1H), 3.88 (dd, *J* = 12.4, 5.4 Hz, 1H), 3.68 (q, *J* = 7.2 Hz, 1H), 2.42 (d, *J* = 7.2 Hz, 2H), 2.00 (s, 3H), 1.97 (s, 3H), 1.86 – 1.77 (m, 1H), 1.46 (d, *J* = 7.2 Hz, 3H), 0.86 (d, *J* = 6.6 Hz, 6H); ^{13}C NMR (100 MHz, Chloroform-*d*) δ 173.2 (s, C), 170.6 (s, C), 170.3 (s, C), 145.7 (s, CH), 140.9 (s, CH), 137.0 (s, CH), 129.5 (s, 2CH), 127.1 (s, 2CH), 99.3 (s, CH), 74.0 (s, CH), 68.3 (s, CH), 67.3 (s, CH), 61.4 (s, CH_2), 45.1 (s, CH), 45.0 (s, CH_2), 30.2 (s, CH), 22.4 (s, 2CH), 21.0 (s, CH_3), 20.7 (s, CH_3), 18.0 (s, CH_3). HRMS Calcd. For $C_{23}H_{31}O_7$ $[M + H^+]^+$, 419.2064. Found: 419.2060.

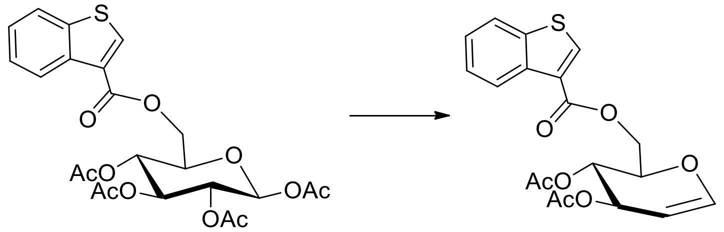

3ad: White solid; yield (93%); ^{1}H NMR (400 MHz, Chloroform-*d*) δ 8.58 (d, *J* = 8.2 Hz, 1H), 8.47 (s, 1H), 7.87 (d, *J* = 8.0 Hz, 1H), 7.49 (t, *J* = 7.6 Hz, 1H), 7.41 (t, *J* = 7.6 Hz, 1H), 6.51 (d, *J* = 6.2 Hz, 1H), 5.38 (d, *J* = 4.0 Hz, 2H), 4.89 (d, *J* = 3.8 Hz, 1H), 4.63 (dd, *J* = 12.2, 3.2 Hz, 1H), 4.55 (dd, *J* = 12.2, 5.8 Hz, 1H), 4.45 (s, 1H), 2.11 (s, 3H), 2.05 (s, 3H); ^{13}C NMR (100 MHz, Chloroform-*d*) δ 170.5 (s, C), 169.8 (s, C), 162.1 (s, C), 145.9 (s, CH), 140.1 (s, C), 137.6 (s, CH), 136.8 (s, C), 126.4 (s, C), 125.7 (s, CH), 125.2 (s, CH), 124.7 (s, CH), 122.6 (s, CH), 99.1 (s, CH), 74.1 (s, CH), 67.5 (s, CH), 67.4 (s, CH), 61.5 (s, CH_2), 21.1 (s, CH_3), 21.0 (s, CH_3). HRMS Calcd. For $C_{19}H_{19}O_7$ $[M + H^+]^+$, 391.0846. Found: 391.0850.

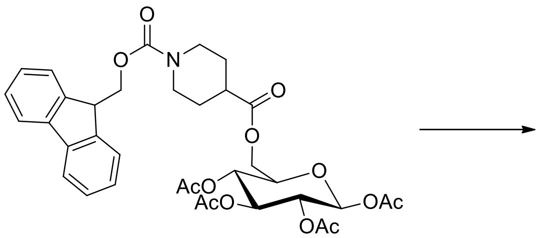

3ae: White solid; yield (90%); ^{1}H NMR (400 MHz, Chloroform-*d*) δ 7.77 (d, *J* = 7.6 Hz, 2H), 7.57 (d, *J* = 7.4 Hz, 2H), 7.40 (t, *J* = 7.4 Hz, 2H), 7.32 (t, *J* = 7.4 Hz, 2H), 6.48 (d, *J* = 6.0 Hz, 1H), 5.39 (s, 1H), 5.28 – 5.22 (m, 1H), 4.84 (dt, *J* = 7.8, 4.0 Hz, 1H), 4.41 (t, *J* = 6.6 Hz, 3H), 4.27 – 4.16 (m, 3H), 4.05 (d, *J* = 28.4 Hz, 2H), 2.91 (s, 2H), 2.55 – 2.42 (m, 1H), 2.10 (s, 3H), 2.02 (s, 3H), 1.87 (d, *J* = 8.2 Hz, 2H), 1.59 (s, 2H); ^{13}C NMR (100 MHz, Chloroform-*d*) δ 173.1 (s, C), 170.7 (s, C), 170.5 (s, C), 155.2 (s, C), 145.8 (s, CH), 144.1 (s, 2C), 141.5 (s, 2C), 127.8 (s, 2CH), 127.2 (s, 2CH), 125.1 (s, 2CH), 120.1 (s, 2CH), 99.2 (s, CH), 74.1 (s, CH), 67.6 (s, CH), 67.4 (s, CH_2), 67.4 (s, CH), 61.4 (s, CH_2), 47.5 (s, CH), 43.2 (s, CH_2), 43.2 (s, CH_2), 40.9 (s, CH), 27.8 (s, CH_2), 27.7 (s, CH_2), 21.1 (s, CH_3), 20.9 (s, CH_3). HRMS Calcd. For $C_{31}H_{34}NO_9$ [M + H$^+$]$^+$, 564.2228. Found: 564.2230.

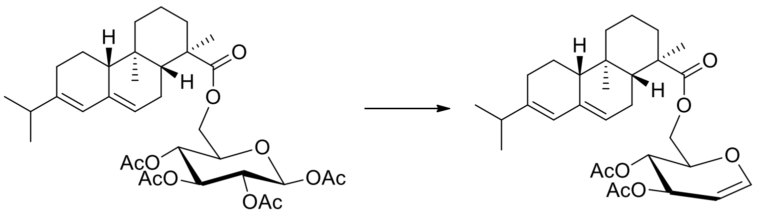

3af: White solid; yield (64%); ^{1}H NMR (400 MHz, Chloroform-*d*) δ 6.45 (d, *J* = 6.0 Hz, 1H), 5.37 – 5.13 (m, 3H), 4.85 (dd, *J* = 6.0, 3.6 Hz, 1H), 4.33 – 4.18 (m, 3H), 2.20 (dd, *J* = 13.4, 7.2 Hz, 1H), 2.05 (d, *J* = 11.4 Hz, 9H), 1.87 (ddd, *J* = 22.4, 15.4, 6.8 Hz, 6H), 1.59 (d, *J* = 10.8 Hz, 3H), 1.26 (d, *J* = 8.4 Hz, 3H), 1.21 (s, 2H), 1.11 – 0.91 (m, 6H), 0.91 – 0.84 (m, 2H), 0.81 (s, 2H); ^{13}C NMR (100 MHz, Chloroform-*d*) δ 178.1 (s, C), 170.3 (s, C), 169.5 (s, C), 145.7 (s, CH), 145.3 (s, C), 135.6 (s, C), 122.4 (s, CH), 120.5 (s, CH), 98.5 (s, CH), 73.8 (s, CH), 67.4 (s, CH), 66.7 (s, CH), 61.4 (s, CH_2), 50.8 (s, CH), 46.7 (s, C), 45.2 (s, CH), 38.2 (s, CH_2), 37.0 (s, CH_2), 34.9 (s, CH), 34.6 (s, C), 27.4 (s, CH_2), 25.6 (s, CH_2), 22.5 (s, CH_2), 21.4 (s, CH_3), 21.1 (s, CH_3), 20.9 (s, $2CH_3$), 18.1 (s, CH_2), 17.0 (s, CH_3), 14.1 (s, CH_3). HRMS Calcd. For $C_{30}H_{43}O_7$ [M + H$^+$]$^+$, 515.3003. Found: 515.3000.

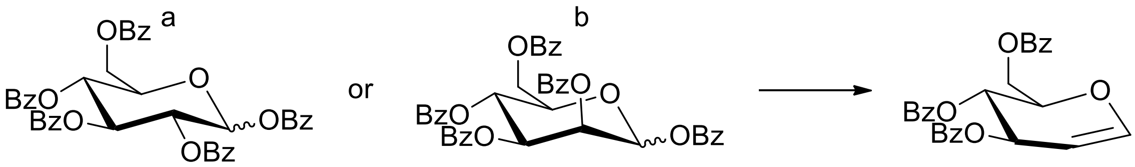

3ag[10c]**:** Pale yellow liquid; yield (90%[a], 88%[b]); ^{1}H NMR (400 MHz, Chloroform-*d*) δ 8.08 – 8.00 (m, 6H), 7.58 – 7.51 (m, 3H), 7.45 – 7.38 (m, 6H), 6.61 (d, J = 6.2 Hz, 1H), 5.82 (t, J = 5.5 Hz, 1H), 5.73 (t, J = 3.9 Hz, 1H), 5.13 (dd, J = 6.1, 3.5 Hz, 1H), 4.71 (s, 3H); ^{13}C NMR (100 MHz, Chloroform-*d*) δ 166.2 (s, C), 165.9 (s, C), 165.2 (s, C), 146.0 (s, CH), 133.5 (s, CH), 133.3 (s, CH), 133.2 (s, CH), 129.9 (s, 2CH), 129.8 (s, 2CH), 129.7 (s, 2CH), 129.6 (s, C), 129.5 (s, C), 129.2 (s, C), 128.6 (s, 2CH), 128.5 (s, 2CH), 128.4 (s, 2CH), 98.8 (s, CH), 74.0 (s, CH), 68.0 (s, CH), 67.5 (s, CH), 62.2 (s, CH_2). HRMS Calcd. For $C_{27}H_{23}O_7$ $[M + H^{+}]^{+}$, 459.1438. Found: 459.1436.

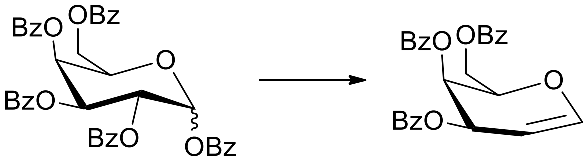

3ah[6a]**:** Pale yellow liquid; yield (88%); ^{1}H NMR (400 MHz, Chloroform-*d*) δ 8.04 (t, *J* = 8.6 Hz, 4H), 7.90 (d, *J* = 8.0 Hz, 2H), 7.59 – 7.48 (m, 3H), 7.42 (t, *J* = 7.8 Hz, 4H), 7.34 (t, *J* = 7.8 Hz, 2H), 6.64 (d, *J* = 6.2 Hz, 1H), 5.94 (d, *J* = 9.2 Hz, 2H), 5.04 – 4.98 (m, 1H), 4.81 (dd, *J* = 11.4, 7.6 Hz, 1H), 4.75 – 4.69 (m, 1H), 4.58 (dd, *J* = 11.4, 4.6 Hz, 1H); ^{13}C NMR (100 MHz, Chloroform-*d*) δ 165.9 (s, C), 165.5 (s, C), 165.2 (s, C), 145.4 (s, CH), 133.1 (s, CH), 132.9 (s, CH), 132.8 (s, CH), 129.6 (s, 3C), 129.4 (s, 2CH), 129.3 (s, 2CH), 128.2 (s, 2CH), 128.1 (s, 3CH), 128.0 (s, 3CH), 98.8 (s, CH), 72.8 (s, CH), 64.7 (s, CH), 64.4 (s, CH), 62.2 (s, CH_2). HRMS Calcd. For $C_{27}H_{23}O_7$ $[M + H^{+}]^{+}$, 459.1438. Found: 459.1436.

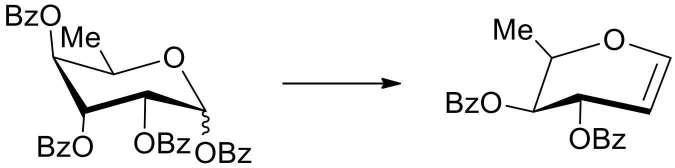

3ai[6a]**:** Pale yellow liquid; yield (92%); ^{1}H NMR (400 MHz, Chloroform-*d*) δ 8.02 (dd, *J* = 13.4, 7.8 Hz, 4H), 7.55 (dd, *J* = 16.2, 7.8 Hz, 2H), 7.42 (q, *J* = 7.4 Hz, 4H), 6.54 (d, *J* = 6.2 Hz, 1H), 5.71 (s, 1H), 5.54 – 5.49 (m, 1H), 5.01 (dd, J = 6.0, 3.0 Hz, 1H), 4.36 (d, *J* = 6.8 Hz, 1H), 1.45 (d, *J* = 6.6 Hz, 3H); ^{13}C NMR (100 MHz, Chloroform-*d*) δ 166.2 (s, C),

165.6 (s, C), 146.3 (s, CH), 133.5 (s, CH), 133.3 (s, CH), 130.0 (s, C), 129.8 (s, C), 128.6 (s, 4CH), 128.5 (s, 4CH), 98.9 (s, CH), 72.8 (s, CH), 72.1 (s, CH), 68.9 (s, CH), 16.8 (s, CH_3). HRMS Calcd. For $C_{20}H_{19}O_5$ $[M + H^+]^+$, 339.1227. Found: 339.1230.

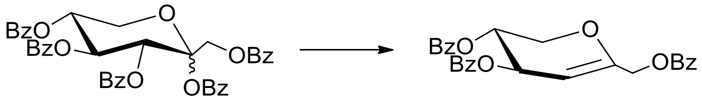

3aj: Pale yellow liquid; yield (83%); ^{1}H NMR (400 MHz, Chloroform-*d*) δ 8.13 – 8.04 (m, 6H), 7.62 – 7.53 (m, 3H), 7.49 – 7.39 (m, 6H), 6.21 – 6.17 (m, 1H), 5.68 (t, *J* = 2.8 Hz, 1H), 4.83 – 4.78 (m, 1H), 4.75 – 4.70 (m, 2H), 4.69 (s, 1H), 4.46 (d, *J* = 1.2 Hz, 1H); ^{13}C NMR (100 MHz, Chloroform-*d*) δ 166.3 (s, C), 165.7 (s, C), 165.4 (s, C), 158.5 (s, C), 133.8 (s, CH), 133.7 (s, CH), 133.3 (s, CH), 130.1 (s, 2CH), 130.0 (s, 2CH), 129.9 (s, 2CH), 129.7 (s, C), 129.2 (s, C), 128.9 (s, C), 128.7 (s, 2CH), 128.6 (s, 2CH), 128.5 (s, 2CH), 87.9 (s, CH), 82.5 (s, CH), 77.5 (s, CH_2), 75.7 (s, CH_2), 64.0 (s, CH). HRMS Calcd. For $C_{27}H_{23}O_7$ $[M + H^+]^+$, 459.1438. Found: 459.1435.

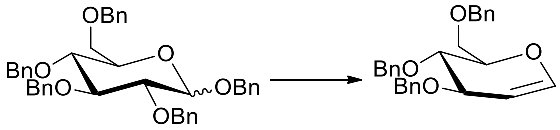

3f[1b]**:** Pale yellow liquid; yield (91%); ^{1}H NMR (400 MHz, Chloroform-*d*) δ 7.33 (d, *J* = 4.2 Hz, 9H), 7.26 (dd, *J* = 17.0, 6.0 Hz, 6H), 6.42 (d, *J* = 6.0 Hz, 1H), 4.88 (dd, *J* = 6.0, 2.4 Hz, 1H), 4.83 (d, *J* = 11.4 Hz, 1H), 4.65 (s, 1H), 4.62 (s, 1H), 4.56 (t, *J* = 8.8 Hz, 3H), 4.21 (d, *J* = 5.0 Hz, 1H), 4.09 – 4.03 (m, 1H), 3.89 – 3.75 (m, 3H); ^{13}C NMR (100 MHz, Chloroform-*d*) δ 144.9 (s, CH), 138.5 (s, C), 138.3 (s, C), 138.1 (s, C), 128.6 (s, 2CH), 128.5 (s, 2CH), 128.5 (s, 2CH), 128.1 (s, 2CH), 127.9 (s, 2CH), 127.8 (s, 3CH), 127.7 (s, 2CH), 100.1 (s, CH), 76.9 (s, CH), 75.9 (s, CH), 74.5 (s, CH), 73.9 (s, CH_2), 73.6 (s, CH_2), 70.6 (s, CH_2), 68.7 (s, CH_2). HRMS Calcd. For $C_{27}H_{29}O_4$ $[M + H^+]^+$, 417.2060. Found: 417.2063.

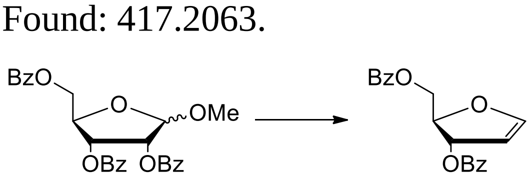

3m: Pale yellow liquid; yield (85%); ^{1}H NMR (400 MHz, Chloroform-*d*) δ 8.08 – 8.03 (m, 4H), 7.57 (dd, *J* = 8.2, 6.8 Hz, 2H), 7.44 (t, *J* = 7.8 Hz, 4H), 6.74 (d, *J* = 2.2 Hz, 1H), 5.99 (s, 1H), 5.34 (t, *J* = 2.6 Hz, 1H), 4.94 – 4.89 (m, 1H), 4.62 (dd, *J* = 11.8, 4.2 Hz, 1H), 4.56 (dd, *J* = 11.8, 6.2 Hz, 1H); ^{13}C NMR (100 MHz, Chloroform-*d*) δ 166.5 (s, C),

166.3 (s, C), 152.2 (s, CH), 133.3 (d, *J* = 5.0 Hz, 2C), 129.8 (d, *J* = 9.7 Hz, 5CH), 128.4(s, 5CH), 99.6 (s, CH), 83.8 (s, CH), 79.3 (s, CH), 64.1 (s, CH_2). HRMS Calcd. For $C_{19}H_{17}O_5$ $[M + H^+]^+$, 325.1071. Found: 325.1069.

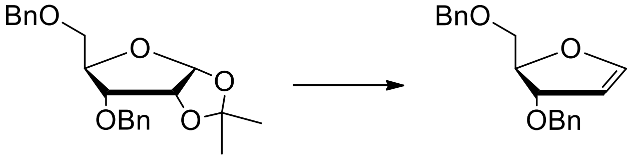

3n[11]**:** Pale yellow liquid; yield (86%); ^{1}H NMR (400 MHz, Chloroform-*d*) δ 7.35 (dd, *J* = 9.2, 5.8 Hz, 10H), 6.61 (d, *J* = 2.4 Hz, 1H), 5.19 (s, 1H), 4.66 (t, *J* = 4.6 Hz, 2H), 4.59 (d, *J* = 7.4 Hz, 2H), 4.53 (s, 2H), 3.56 (dd, *J* = 9.8, 6.4 Hz, 1H), 3.43 (dd, *J* = 10.2, 5.2 Hz, 1H); ^{13}C NMR (100 MHz, Chloroform-*d*) δ 150.5 (s, CH), 138.3 (s, C), 137.9 (s, C), 128.5 (s, 4CH), 128.0 (s, 2CH), 127.8 (t, J = 5.7 Hz, 4CH), 100.7 (s, CH), 84.9 (s, CH), 82.7 (s, CH), 73.5 (s, CH_2), 69.9 (s, CH_2), 69.7 (s, CH_2). HRMS Calcd. For $C_{19}H_{21}O_3$ $[M + H^+]^+$, 297.1485. Found: 297.1480.

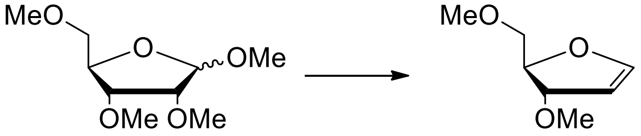

3o: Yellow liquid, yield (80%); ^{1}H NMR (400 MHz, Chloroform-*d*) δ 6.55 (d, *J* = 1.8 Hz, 1H), 5.15 (s, 1H), 4.49 (s, 1H), 4.41 (s, 1H), 3.46 (dd, *J* = 10.2, 6.8 Hz, 1H), 3.39 (s, 3H), 3.36 (d, *J* = 5.4 Hz, 1H), 3.27 (s, 3H); ^{13}C NMR (100 MHz, Chloroform-*d*) δ 149.9 (s, CH), 99.6 (s, CH), 83.7 (s, CH), 83.7 (s, CH), 72.3 (s, CH_2), 58.8 (s, CH), 54.1 (s, CH). HRMS Calcd. For $C_7H_{13}O_3$ $[M + H^+]^+$, 145.0859. Found: 145.0860.

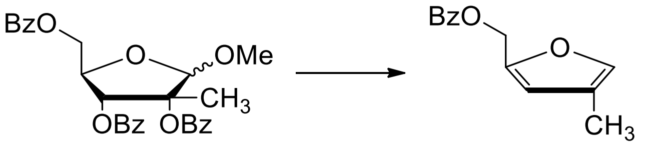

4p: Pale yellow liquid; yield (85%); ^{1}H NMR (400 MHz, Chloroform-*d*) δ 7.94 – 7.91 (m, 2H), 7.40 (t, *J* = 7.4 Hz, 1H), 7.29 (d, *J* = 7.8 Hz, 2H), 7.07 (s, 1H), 6.22 (s, 1H), 5.13 (s, 2H), 1.89 (d, *J* = 0.8 Hz, 3H); ^{13}C NMR (100 MHz, Chloroform-*d*) δ 166.3 (s, C), 149.6 (s, C), 140.0 (s, CH), 133.1 (s, CH), 130.1 (s, C), 129.8 (s, 2CH), 128.4 (s, 2CH), 121.0 (s, C), 113.5 (s, CH), 58.8 (s, CH_2), 9.7 (s, CH_3). HRMS Calcd. For $C_{13}H_{13}O_3$ $[M + H^+]^+$, 217.0859. Found: 217.0858.

7. General Procedure for the Synthesis of Deuterated Glycals 1*D*-3.

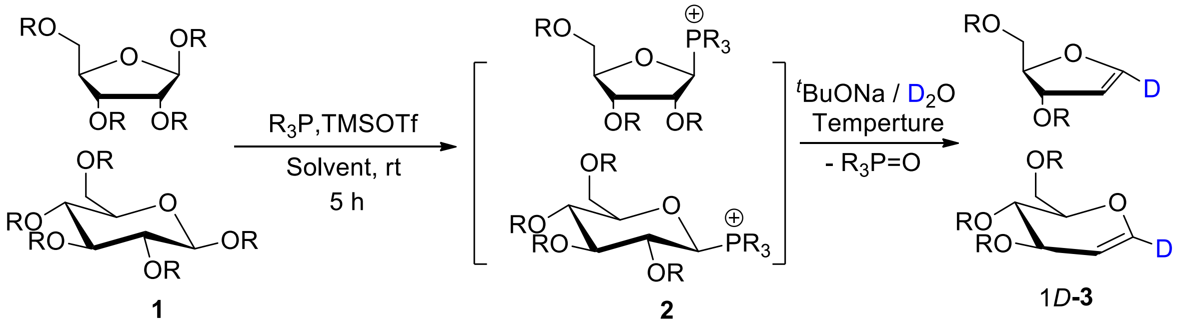

Compounds **1** (1 mmol) and triphenylphosphine (1.2 mmol) were dissolved in DCM (5 mL) in a Schlenk bottle under argon gas atmosphere, TMSOTf (1.1 mmol) was added under 0 °C and the mixture was stirred at room temperature for 5-6 h , and then corresponding phosphonium ylides **2** were obtained. Crude products not purified further and potassium t-butoxide and deuterium oxide added directly at room temperature. After the reaction was finished and then DCM was removed under reduced pressure. The residue was column chromatography to afford the corresponding products **1*D*-3**.

8. The Analytical and Spectral Characterization Data of Deuterated Compounds 1*D*-3.

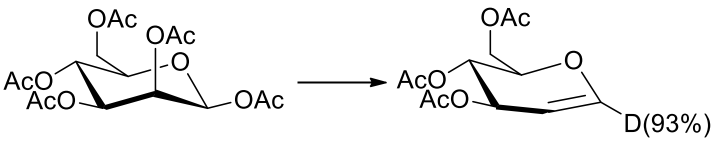

***D*-3a[12b]:** Pale yellow liquid; yield (83%); ^{1}H NMR (400 MHz, Chloroform-*d*) δ 5.34 (dd, *J* = 5.4, 3.2 Hz, 1H), 5.22 (dd, *J* = 7.4, 6.0 Hz, 1H), 4.84 (d, *J* = 3.2 Hz, 1H), 4.40 (dd, *J* = 12.0, 5.8 Hz, 1H), 4.25 (dd, *J* = 10.8, 5.4 Hz, 1H), 4.21 – 4.17 (m, 1H), 2.09 (s, 3H), 2.07 (s, 3H), 2.04 (s, 3H); ^{13}C NMR (100 MHz, Chloroform-*d*) δ 170.6 (s, C), 170.4 (s, C), 169.6 (s, C), 145.7 (s, CH), 98.8 (s, CH), 74.0 (s, CH), 67.4 (s, CH), 67.2 (s, CH), 61.4 (s, CH_2), 21.0 (s, CH_3), 20.8 (s, CH_3), 20.7 (s, CH_3). HRMS Calcd. For $C_{12}H_{16}DO_7$ $[M+H^+]^+$, 274.1032. Found: 274.1030.

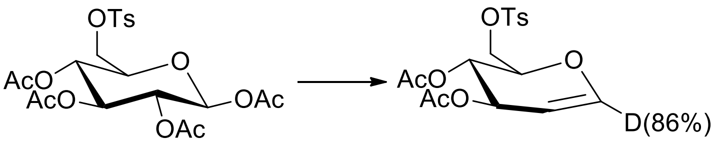

***D*-3b:** Pale yellow liquid; yield (83%); ^{1}H NMR (400 MHz, Chloroform-*d*) δ 7.78 (d, *J* = 8.4 Hz, 2H), 7.34 (d, *J* = 8.0 Hz, 2H), 5.25 (dd, *J* = 4.8, 3.8 Hz, 1H), 5.12 (t, *J* = 5.8 Hz, 1H), 4.80 (d, *J* = 3.4 Hz, 1H), 4.22 (m, *J* = 13.0, 9.6, 5.4 Hz, 3H), 2.44 (s, 3H), 2.02 (s, 3H), 2.01 (s, 3H); ^{13}C NMR (100 MHz, Chloroform-*d*) δ 170.3 (s, C), 169.5 (s, C), 145.4 (s, 2C), 145.2 (s, CH), 130.0 (s, 2CH), 128.1 (s, 2CH), 99.0 (s, CH), 73.3 (s, CH), 67.1 (s, CH_2), 66.7 (s, CH), 66.5 (s, CH), 21.8 (s, CH_3), 21.0 (s, CH_3), 20.8 (s, CH_3). HRMS Calcd. For $C_{17}H_{20}DO_8S$ $[M+H^+]^+$, 386.1014. Found: 386.1015.

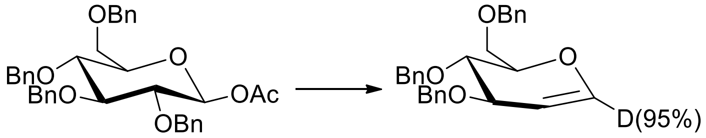

***D*-3f[12b]:** Pale yellow liquid; yield (93%); ^{1}H NMR (400 MHz, Chloroform-*d*) δ 7.41 – 7.21 (m, 16H), 4.85 (dd, *J* = 15.4, 6.8 Hz, 2H), 4.66 – 4.52 (m, 5H), 4.21 (dd, *J* = 6.0, 2.2 Hz, 1H), 4.06 (dd, *J* = 7.2, 4.0 Hz, 1H), 3.90 – 3.74 (m, 3H); ^{13}C NMR (100 MHz, Chloroform-*d*) δ 144.9 (s, CH), 138.5 (s, C), 138.3 (s, C), 138.1 (s, C), 128.5 (s, 2CH), 128.5 (s, 2CH), 128.4 (s, 2CH), 128.1 (s, 2CH), 127.9 (s, 2CH), 127.9 (s, 3CH), 127.8 (s, 2CH), 100.1 (s, CH), 76.9 (s, CH), 75.9 (s, CH), 74.5 (s, CH), 73.9 (s, CH_2), 73.6 (s, CH_2), 70.6 (s, CH_2), 68.7 (s, CH_2). HRMS Calcd. For $C_{27}H_{28}DO_4$ $[M+H^+]^+$, 418.2123. Found: 418.2125.

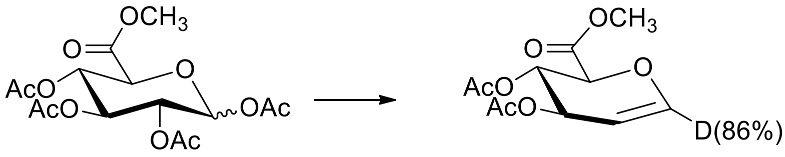

***D*-3g:** White solid; yield (82%); ^{1}H NMR (400 MHz, Chloroform-*d*) δ 5.40 (dd, *J* = 4.2, 2.6 Hz, 1H), 5.03 – 4.95 (m, 2H), 4.82 (d, *J* = 1.2 Hz, 1H), 3.78 (s, 3H), 2.10 (s, 3H), 1.97 (s, 3H); ^{13}C NMR (100 MHz, Chloroform-*d*) δ 169.7 (s, C), 169.4 (s, C), 167.4 (s, C), 146.5 (s, CH), 97.4 (s, CH), 72.4 (s, CH), 67.5 (s, CH), 62.6 (s, CH), 52.5 (s, CH), 21.1 (s, CH_3), 21.0 (s, CH_3). HRMS Calcd. For $C_{11}H_{14}DO_7$ $[M+H^+]^+$, 260.0875. Found: 260.0873.

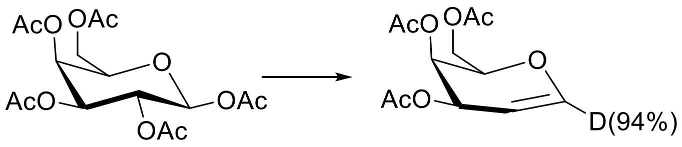

***D*-3h:** Pale yellow liquid; yield (85%); ^{1}H NMR (400 MHz, Chloroform-*d*) δ 5.55 (d, *J* = 2.8 Hz, 1H), 5.45 – 5.40 (m, 1H), 4.73 (s, 1H), 4.32 (dd, *J* = 11.4, 5.6 Hz, 1H), 4.28 – 4.16 (m, 2H), 2.13 (s, 3H), 2.09 (s, 3H), 2.03 (s, 3H); ^{13}C NMR (100 MHz, Chloroform-*d*) δ 170.7 (s, C), 170.5 (s, C), 169.7 (s, C), 145.8 (s, CH), 99.1 (s, CH), 74.1 (s, CH), 67.6 (s, CH), 67.3 (s, CH), 61.5 (s, CH_2), 21.1 (s, CH_3), 20.9 (s, CH_3), 20.8 (s, CH_3). HRMS Calcd. For $C_{12}H_{16}DO_7$ [M + H$^+$]$^+$, 274.1032. Found: 274.1030.

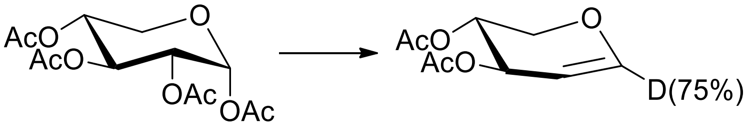

***D*-3i:** Pale yellow liquid; yield (90%); ^{1}H NMR (400 MHz, Chloroform-*d*) δ 4.97 (s, 3H), 4.20 (d, J = 12.0 Hz, 1H), 3.97 (d, J = 12.2 Hz, 1H), 2.08 (d, J = 12.6 Hz, 6H); ^{13}C NMR (100 MHz, Chloroform-*d*) δ 170.1 (s, C), 169.9 (s, C), 148.2 (s, CH), 97.5 (s, CH), 67.3 (s, CH), 63.7 (s, CH_2), 63.5 (s, CH), 21.3 (s, CH3), 21.1 (s, CH_3). HRMS Calcd. For $C_9H_{12}DO_5$ [M + H$^+$]$^+$, 202.0820. Found: 202.0825.

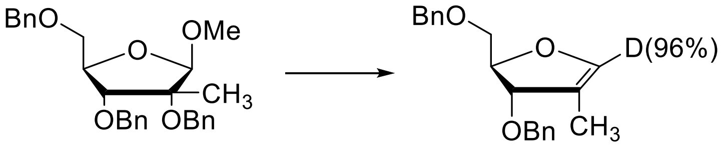

***D*-3q:** Pale yellow liquid; yield (90%); ^{1}H NMR (400 MHz, Chloroform-*d*) δ 7.39 – 7.29 (m, 10H), 4.63 – 4.55 (m, 4H), 4.49 (d, *J* = 13.0 Hz, 2H), 3.59 – 3.53 (m, 1H), 3.43 – 3.37 (m, 1H); ^{13}C NMR (100 MHz, Chloroform-*d*) δ 144.2 (s, CH), 138.5 (s, C), 138.0 (s, C), 128.4 (d, *J* = 5.0 Hz, C), 127.7 (t, *J* = 12.2 Hz, C), 109.8 (s, C), 85.8 (s, C), 84.4 (s, C), 73.5 (s, C), 70.2 (s, C), 69.6 (s, C), 9.0 (s, C). HRMS Calcd. For $C_{20}H_{22}DO_3$ [M + H$^+$]$^+$, 312.1704. Found: 312.1700.

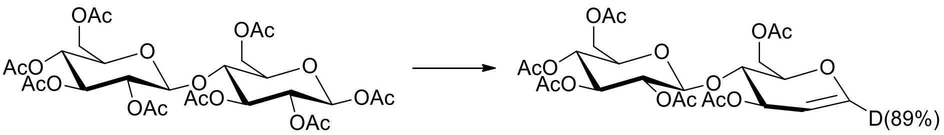

***D*-3s:** Pale yellow liquid; yield (87%); ^{1}H NMR (400 MHz, Chloroform-*d*) δ 5.38 (dd, *J* = 5.6, 3.4 Hz, 1H), 5.15 (t, *J* = 9.4 Hz, 1H), 5.04 (t, *J* = 9.6 Hz, 1H), 4.97 – 4.90 (m, 1H), 4.78 (d, *J* = 3.4 Hz, 1H), 4.66 (d, *J* = 8.0 Hz, 1H), 4.41 (dd, *J* = 11.4, 2.2 H z, 1H), 4.27 (dd, *J* = 12.4, 4.6 Hz, 1H), 4.12 (ddd, *J* = 8.8, 8.0, 4.1 Hz, 2H), 4.03 (dd, *J* = 12.4, 2.2 Hz, 1H), 3.95 (dd, *J* = 7.4, 5.8 Hz, 1H), 3.66 (ddd, *J* = 10.0, 4.4, 2.4 Hz, 1H), 2.

08 (s, 3H), 2.05 (s, 3H), 2.01 (d, *J* = 2.2 Hz, 6H), 1.98 (s, 3H), 1.96 (s, 3H); ^{13}C NMR (100 MHz, Chloroform-*d*) δ 170.8 (s, C), 170.6 (s, C), 170.4 (s, C), 170.1 (s, C), 169.4 (s, C), 169.3 (s, C), 145.6 (s, CH), 100.7 (s, CH), 99.2 (s, CH), 74.8 (s, CH), 74.5 (s, CH), 72.9 (s, CH), 72.1 (s, CH), 71.5 (s, CH), 68.7 (s, CH), 68.2 (s, CH), 61.9 (s, CH_2), 61.9 (s, CH_2), 21.1 (s, CH_3), 21.0 (s, CH_3), 20.8 (s, CH_3), 20.7 (s, 2CH_3) , 20.6 (s, CH_3). HRMS Calcd. For $C_{24}H_{32}DO_{15}$ $[M+H^+]^+$, 562.1877. Found: 562.1875.

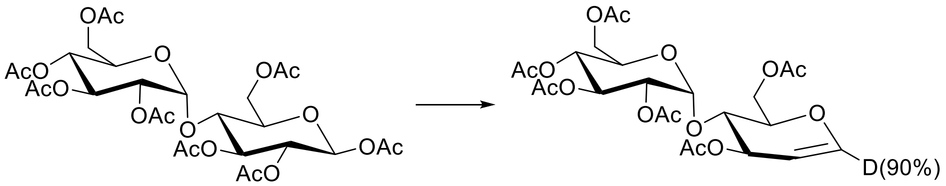

***D*-3u:** Pale yellow liquid; yield (89%); ^{1}H NMR (400 MHz, Chloroform-*d*) δ 5.48 (d, *J* = 3.8 Hz, 1H), 5.38 (t, *J* = 10.0 Hz, 1H), 5.18 – 5.12(m, 1H), 5.03 (t, *J* = 10.0 Hz, 1H), 4.83 – 4.78 (m, 2H), 4.34 (t, *J* = 4.8 Hz, 2H), 4.30 – 4.26(m, 1H), 4.22 (dd, *J* = 12.4, 4.2 Hz, 1H), 4.07 (dd, *J* = 12.4, 4.2 Hz, 1H), 4.04 – 3.97(m, 2H), 2.10 (s, 3H), 2.08 (s, 3H), 2.03 (d, *J* = 2.0 Hz, 6H), 2.01 (s, 3H), 1.99 (s, 3H); ^{13}C NMR (100 MHz, Chloroform-*d*) δ 170.6 (s, C), 170.5 (s, C), 170.4 (s, 2C), 170.0 (s, C), 169.5 (s, C), 145.6 (s, CH), 98.4 (s, CH), 95.8 (s, CH), 74.1 (s, CH), 72.5 (s, CH), 70.4 (s, CH), 69.6 (s, CH), 69.5 (s, CH), 68.3 (s, CH), 68.2 (s, CH), 61.9 (s, CH_2), 61.6 (s, CH_2), 21.1 (s, CH_3), 20.8 (s, CH_3), 20.7 (s, CH_3), 20.6 (s, CH_3), 20.5 (s, CH_3), 20.5 (s, CH_3). HRMS Calcd. For $C_{24}H_{32}DO_{15}$ $[M+H^+]^+$, 562.1877. Found: 562.1875.

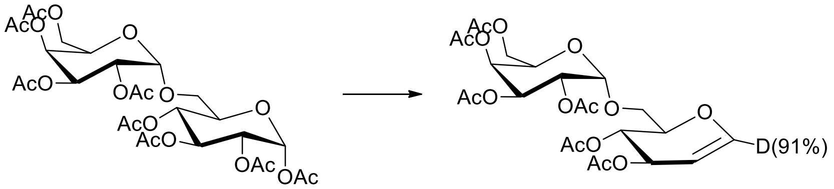

***D*-3w:** White solid, yield (85%); ^{1}H NMR (400 MHz, Chloroform-*d*) δ 5.47 (d, *J* = 2.6 Hz, 1H), 5.35 (dd, *J* = 10.8, 3.2 Hz, 1H), 5.28 – 5.24 (m, 1H), 5.23 – 5.16 (m, 2H), 5.11 (dd, *J* = 10.8, 3.2 Hz, 1H), 4.86 (d, *J* = 3.4 Hz, 1H), 4.28 (t, *J* = 5.2 Hz, 2H), 4.10 (d, *J* = 6.6 Hz, 2H), 3.87 (dd, *J* = 11.2, 6.2 Hz, 1H), 3.70 (dd, *J* = 11.2, 4.2 Hz, 1H), 2.15 (s, 3H), 2.11 (d, *J* = 3.2 Hz, 6H), 2.05 (s, 6H), 1.99 (s, 3H); ^{13}C NMR (100 MHz, Chloroform-*d*) δ 170.6 (s, C), 170.3 (s, C), 170.2 (s, C), 170.2 (s, C), 169.9 (s, C), 169.5 (s, C), 145.7 (s, CH), 98.3 (s, CH), 96.3 (s, CH), 74.4 (s, CH), 68.0 (s, 2CH), 67.5 (s, CH), 66.6 (s, CH),

66.4 (s, CH), 65.6 (s, CH_2), 61.7 (s, CH_2), 21.0 (s, CH_3), 20.8 (s, CH_3), 20.7 (s, CH_3), 20.7 (s, CH_3), 20.6 (s, CH_3), 20.5 (s, CH_3). HRMS Calcd. For $C_{24}H_{32}DO_{15}$ $[M + H^+]^+$, 562.1877. Found: 562.1873.

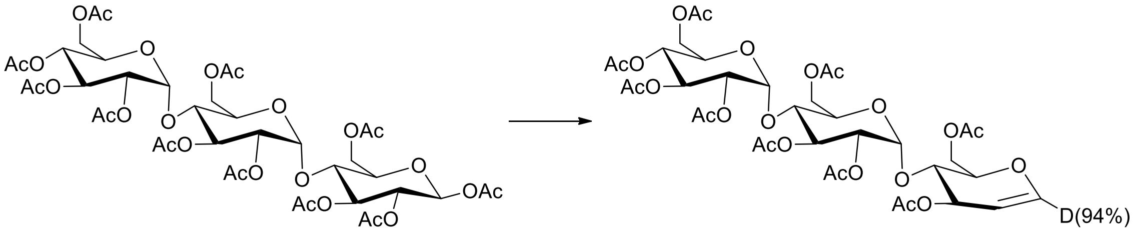

***D*-3x:** Pale yellow liquid; yield (81%); ^{1}H NMR (400 MHz, Chloroform-*d*) δ 5.46 – 5.40 (m, 1H), 5.40 – 5.31 (m, 3H), 5.21 – 5.16 (m, 1H), 5.05 (t, *J* = 10.0 Hz, 1H), 4.85 (dd, *J* = 10.6, 4.0 Hz, 1H), 4.79 (d, *J* = 3.2 Hz, 1H), 4.69 (dd, *J* = 10.4, 4.0 Hz, 1H), 4.53 – 4.45 (m, 1H), 4.38 (d, *J* = 4.6 Hz, 2H), 4.29 - 4.15 (m, 3H), 4.07 – 3.91 (m, 5H), 2.14 (d, *J* = 0.8 Hz, 6H), 2.08 (s, 3H), 2.04 (s, 3H), 2.01 (d, *J* = 1.6 Hz, 6H), 2.00 – 1.96 (m, 9H); ^{13}C NMR (100 MHz, Chloroform-*d*) δ 170.7 (s, C), 170.6 (s, 2C), 170.6 (s, C), 170.5 (s, 2C), 170.0 (s, C), 169.9 (s, C), 169.6 (s, C), 145.8 (s, CH), 98.7 (s, CH), 95.8 (s, CH), 95.7 (s, CH), 74.2 (s, CH), 72.8 (s, CH), 72.6 (s, CH), 72.0 (s, CH), 71.0 (s, CH), 70.1 (s, CH), 69.9 (s, CH), 69.4 (s, CH), 68.8 (s, CH), 68.6 (s, CH), 68.0 (s, CH), 62.5 (s, CH_2), 62.1 (s, CH_2), 61.5 (s, CH_2), 21.2 (s, CH_3), 21.0 (s, CH_3), 20.9 (s, $2CH_3$), 20.8 (s, CH_3), 20.7 (s, $3CH_3$), 20.6 (s, CH_3). HRMS Calcd. For $C_{36}H_{48}DO_{23}$ $[M + H^+]^+$, 850.2722. Found: 850.2724.

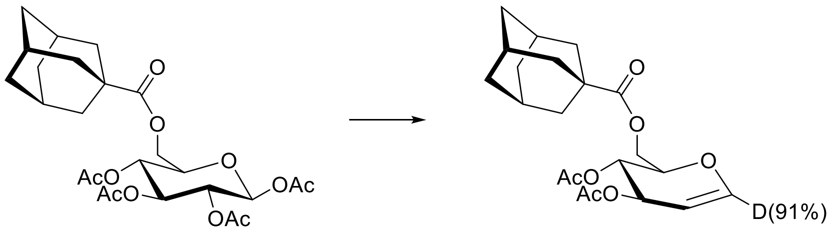

***D*-3aa:** White solid; yield (88 %); ^{1}H NMR (400 MHz, Chloroform-*d*) δ 5.42 – 5.35 (m, 1H), 5.23 (t, *J* = 7.0 Hz, 1H), 4.81 (d, *J* = 1.8 Hz, 1H), 4.32 (dd, *J* = 12.0, 5.4 Hz, 1H), 4.25 – 4.14 (m, 2H), 2.08 (s, 3H), 2.02 (s, 3H), 1.99 (s, 3H), 1.83 (s, 6H), 1.73 – 1.64 (m, 6H); ^{13}C NMR (100 MHz, Chloroform-*d*) δ 176.1 (s, C), 170.7 (s, C), 170.5 (s, C), 145.7 (s, CH), 99.4 (s, CH), 74.3 (s, CH), 67.9 (s, CH), 66.6 (s, CH), 61.6 (s, CH_2), 40.8 (s, C),

38.6 (s, CH_2), 36.4 (s, CH_2), 27.8 (s, CH), 21.1 (s, CH_3), 20.8 (s, CH_3). HRMS Calcd. For $C_{21}H_{28}DO_7$ $[M + H^+]^+$, 394.1971. Found: 394.1975.

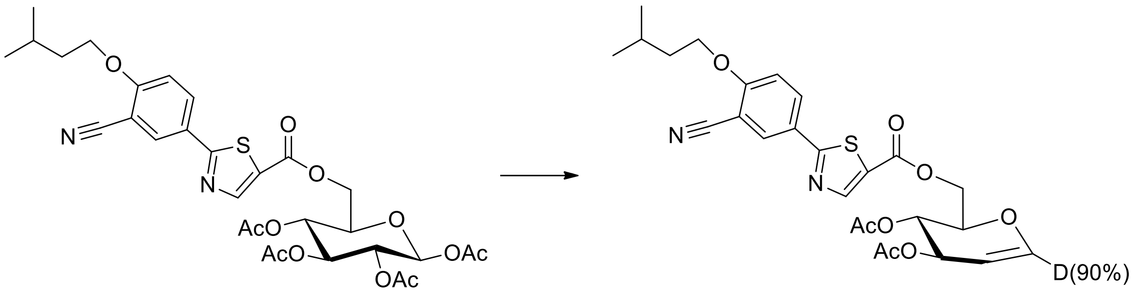

***D*-3ab:** Pale yellow liquid; yield (89%); ^{1}H NMR (400 MHz, Chloroform-*d*) δ 8.16 (dd, *J* = 5.6, 2.4 Hz, 1H), 8.06 (dd, *J* = 8.8, 2.2 Hz, 1H), 7.00 (d, *J* = 9.0 Hz, 1H), 5.53 – 5.28 (m, 2H), 4.90 (d, *J* = 3.4 Hz, 1H), 4.53 – 4.35 (m, 2H), 4.27 (d, *J* = 8.8 Hz, 1H), 3.88 (d, *J* = 6.6 Hz, 2H), 2.72 (s, 3H), 2.18 (dt, *J* = 13.4, 6.6 Hz, 1H), 2.09 (s, 3H), 2.04 (s, 3H), 1.07 (d, *J* = 6.8 Hz, 6H); ^{13}C NMR (100 MHz, Chloroform-*d*) δ 170.5 (s, C), 170.3 (s, C), 168.1 (s, C), 162.6 (s, C), 162.5 (s, C), 160.5 (s, C), 145.7 (s, CH), 132.7 (s, CH), 132.1 (s, CH), 125.7 (s, C), 120.4 (s, C), 115.3 (s, C), 112.7 (s, CH), 103.0 (s, C), 98.8 (s, CH), 75.7 (s, CH_2), 73.8 (s, CH), 68.1 (s, CH), 66.8 (s, CH), 61.4 (s, CH_2), 28.1 (s, CH_3), 21.0 (s, CH_3), 20.7 (s, CH_3), 19.0 (s, CH_3), 17.6 (s, CH_3). HRMS Calcd. For $C_{26}H_{28}DN_2O_8S$ $[M + H^+]^+$, 530.1702. Found: 530.1705.

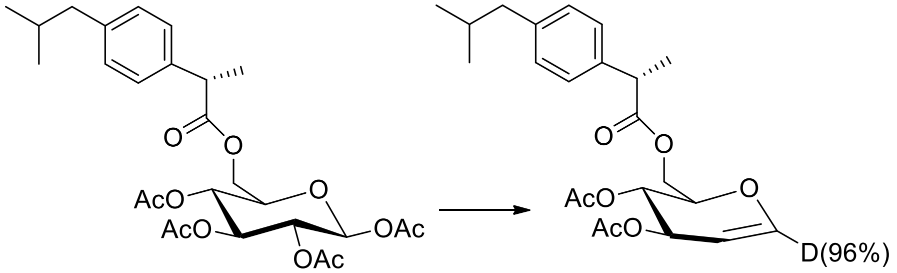

***D*-3ac:** Pale yellow liquid; yield (92%); ^{1}H NMR (400 MHz, Chloroform-*d*) δ 7.15 (d, *J* = 8.2 Hz, 2H), 7.08 (d, *J* = 8.0 Hz, 2H), 5.39 (dd, *J* = 6.4, 3.0 Hz, 1H), 5.24 (dd, *J* = 8.6, 6.4 Hz, 1H), 4.79 (d, *J* = 3.0 Hz, 1H), 4.15 – 4.00 (m, 2H), 3.90 (dd, *J* = 12.4, 5.6 Hz, 1H), 3.69 (m, 1H), 2.43 (d, *J* = 7.2 Hz, 2H), 2.02 (s, 3H), 1.99 (s, 3H), 1.90 – 1.75 (m, 1H), 1.47 (d, *J* = 7.2 Hz, 3H), 0.88 (d, *J* = 6.6 Hz, 6H); ^{13}C NMR (100 MHz, Chloroform-*d*) δ 173.2 (s, C), 170.6 (s, C), 170.3 (s, C), 145.7 (s, CH), 140.9 (s, CH), 137.0 (s, CH), 129.5 (s, 2CH), 127.1 (s, 2CH), 99.3 (s, CH), 74.0 (s, CH), 68.3 (s, CH), 67.3 (s, CH), 61.4 (s, CH_2), 45.1 (s, CH), 45.0 (s, CH_2), 30.2 (s, CH), 22.4 (s, 2CH), 21.0

(s, CH_3), 20.7 (s, CH_3), 18.0 (s, CH_3). HRMS Calcd. For $C_{23}H_{30}DO_7$ $[M+H^+]^+$, 420.2127. Found: 420.2126.

9. The Larger-scale (200 g) Reactions.

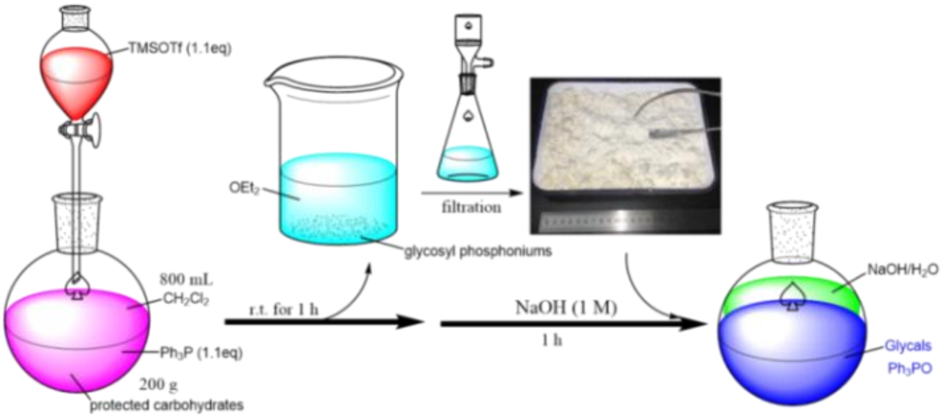

Fig. S9 The hundred gram-scale reaction process (schematic plot). The mixture of Glucose pentaacetate **1a** (200 g), Ph_3P (1.1 eq), and TMSOTf (1.1 eq) in DCM (700–800 mL) was stirred at room temperature for 100 h. The purified solid phosphonium **2a** was separated from Et_2O (500 mL) and then the gycal **3a** (370 g, 94%) was given by $NaOH/H_2O$ (2 M) hydrolysis-elimination.

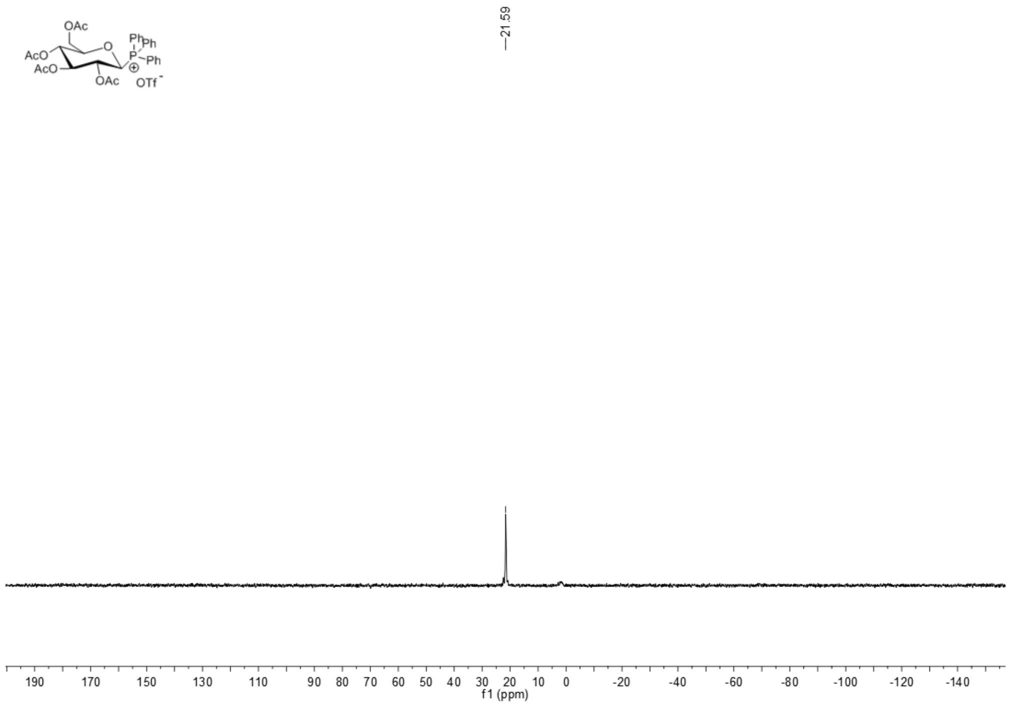

^{31}P NMR ($CDCl_3$-*d*) of intermediate **2a** separated by simple filtration

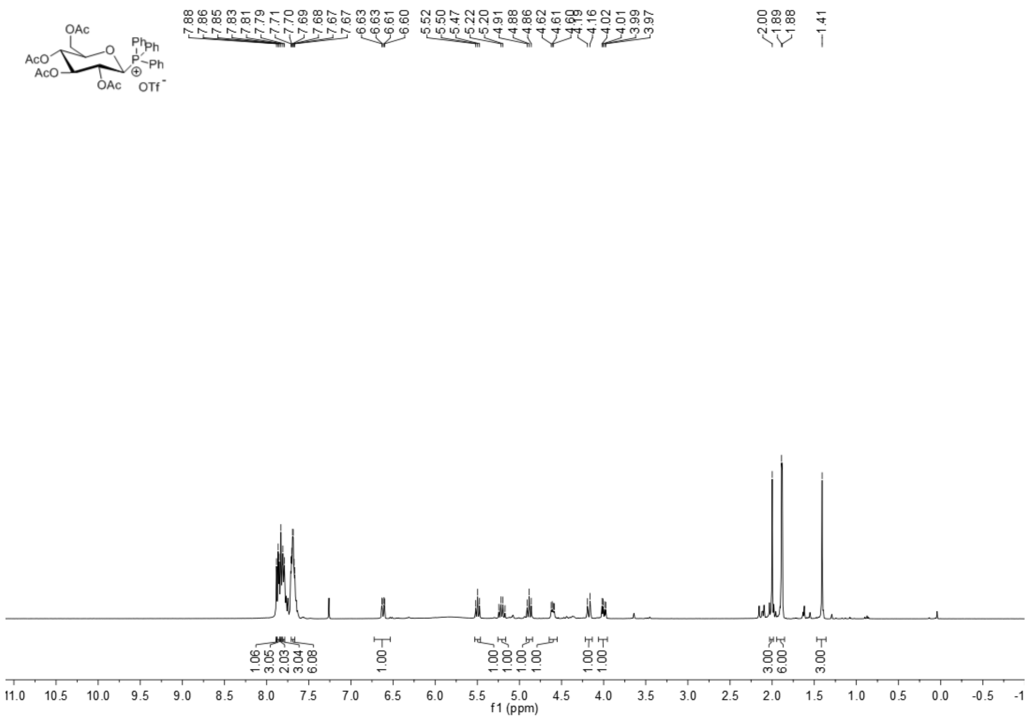

^{1}H NMR ($CDCl_3$-*d*) of intermediate **2a** separated by simple filtration.

10. References

[1] a) Csuk, R.; Fürstner, A.; Glänzer, B. I.; Weidmann, H, *J. Chem. Soc., Chem. Commun.* **1986**, *15*, 1149-1150; b) Zhao, J.; Wei, S.; Ma, X.; Shao, H. *Carbohydr. Res.* **2010**, *345*, 168-171; c) Xu, Y.; Wang, W.; Cai, Y.; Yang, X.; Wang, P. G.; Zhao, W. RSC Adv. **2014**, *4*, 46662-46665; d) Chen, H.; Xian, T.; Zhang, W.; Si, W.; Luo, X.; Zhang, B.; Zhang, J. *Carbohydr. Res.* **2016**, *431*, 42-46.

[2] Ireland, R. E.; Wilcox, C. S.; Thaisrivongs, S.; *J. Org. Chem.* **1978**, *43*, 786-787.

[3] a) POLLON, J. P.; Llewellyn, G.; Williams, J. M, *Synthesis,* **1989,** *10*, 758-759; b) Kovács, G.; Micskei, K.; Somsák, L.; *Carbohydr. Res*. **2001**, *336*, 225-228.

[4] a) Cavallaro, C. L.; Schwartz, J.; *J. Org. Chem.* **1995**, *60*, 7055-7057; b) Spencer, R. P.; & Schwartz, J.; *Tetrahedron Lett.* **1996**, *37*, 4357-4360; c) Spencer, R. P.; Cavallaro, C. L.; Schwartz, J.; *J. Org. Chem.* **1999**, 64, 3987-3995; d) Hansen, T.; Krintel, S. L.; Daasbjerg, K.; Skrydstrup, T.; *Tetrahedron Lett.* **1999**, 40, 6087-6090; e) Hansen, T.; Daasbjerg, K.; Skrydstrup, T.; *Tetrahedron Lett.* **2000**, 41, 8645-8649.

[5] Shull, B. K.; Wu, Z.; Koreeda, M.; *J. Carbohydr. Chem.* **1996**, *15*, 955-964.

[6] a) Zhao, J.; Wei, S.; Ma, X.; Shao, H.; *Green Chem.* **2009**, *11*, 1124-1127; b) Zhao, J.; Shao, H.; Wu, X.; Shi, S. *Carbohydr. Res.* **2011**, *29*, 1434-1440.

[7] Kovács, G.; Micskei, K.; Somsák, L.; *Carbohydr. Res.* **2001**, *336*, 225-228.

[8] Micskei, K.; Juhász, Z.; Ratković, Z. R.; Somsák, L.; *Tetrahedron Lett.* **2006**, *47*, 6117-6120.

[9] Gómez, A. M.; Casillas, M.; Barrio, A.; Gawel, A.; López, J. C.; *Eur. J. Org. Chem.* **2008**, 3933-3942.

[10] a) Maran, F.; Vianello, E.; Catelani, G.; D'Angeli, F.; *Electrochim. Acta*. **1989**, *34*, 587-589; b) Rondinini, S.; Mussini, P. R.; Ferzetti, V.; Monti, D.; *Electrochim. Acta*. **1991**, *36*, 1095-1098; c) Parrish, J. D.; Little, R. D.; *Tetrahedron Lett*, **2001**, *42*, 7371-7374.

[11] Bravo, F.; Kassou, M.; Castillón, S.; Tetrahedron Lett. **1999**, *40*, 1187-1190.

[12] a) Zhang, S.; Niu, Y. H.; Ye, X. S. *Org. Lett.* **2017**, *19*, 3608−3611; b) Yi, D.; Zhu, F.; Walczak, M. A. *Org. Lett.* **2018**, *20*, 4627−4631.

11. X-ray Crystal Structures of Compounds 2a′.

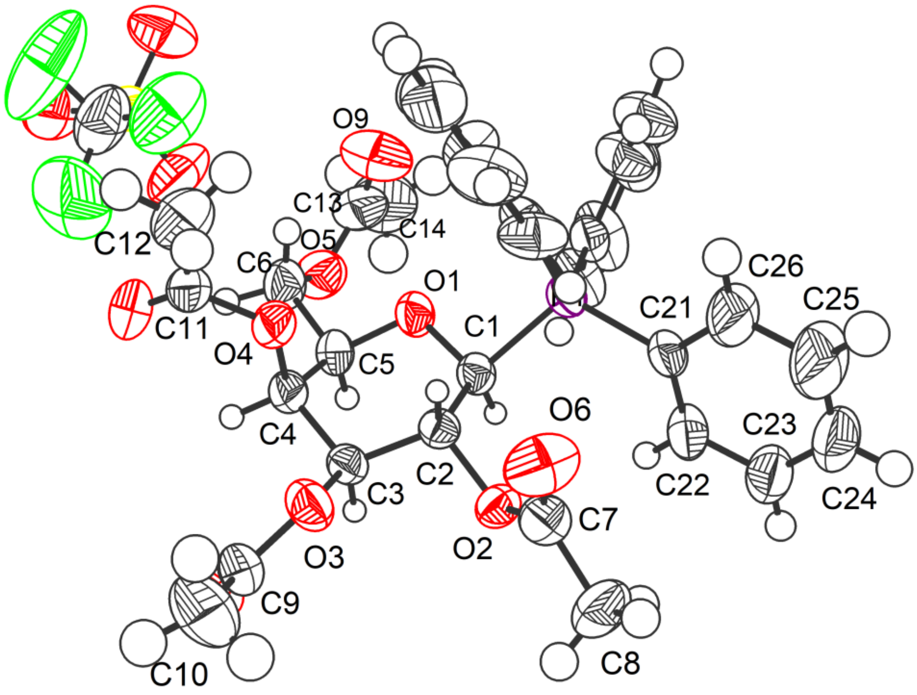

Table S2 Crystal data and structure refinement for 2a′. CCDC 2142375.

Identification code	200528HUANGHY_0m
Empirical formula	$C_{33}H_{34}F_3O_{12}PS$
Formula weight	742.63
Temperature/K	296.15
Crystal system	monoclinic
Space group	$P2_1$
a/Å	10.5045(14)
b/Å	16.820(2)
c/Å	11.0991(15)
α/°	90
β/°	112.514(2)
γ/°	90
Volume/Å^3	1811.6(4)

Z	2
ρ_{calc}g/cm^3	1.361
μ/mm^{-1}	0.208
F(000)	772.0
Crystal size/mm^3	0.03 × 0.03 × 0.03
Radiation	MoKα (λ = 0.71073)
2Θ range for data collection/ °	4.652 to 55.326
Index ranges	-13 ≤ h ≤ 13, -21 ≤ k ≤ 21, -13 ≤ l ≤ 14
Reflections collected	14659
Independent reflections	7768 [R_{int} = 0.0253, R_{sigma} = 0.0480]
Data/restraints/parameters	7768/2/455
Goodness-of-fit on F^2	1.027
Final R indexes [I>=2σ (I)]	R_1 = 0.0560, wR_2 = 0.1357
Final R indexes [all data]	R_1 = 0.0847, wR_2 = 0.1534
Largest diff. peak/hole / e Å^{-3}	0.53/-0.24
Flack parameter	0.04(4)

Table S3 Bond Lengths for 2a′.

Atom	Atom	Length/Å	Atom	Atom	Length/Å
P1	C15	1.790(5)	C2	C1	1.551(6)
P1	C1	1.849(4)	C21	C22	1.380(8)
P1	C21	1.793(5)	C21	C26	1.353(8)
P1	C27	1.786(5)	F1	C33	1.299(10)
S1	O11	1.417(6)	C5	C4	1.517(6)
S1	O12	1.415(5)	C5	C6	1.504(7)
S1	O10	1.417(5)	C11	C12	1.497(9)
S1	C33	1.798(9)	O9	C13	1.197(9)
O2	C7	1.363(6)	C27	C32	1.391(8)
O2	C2	1.436(5)	C27	C28	1.387(8)
O3	C3	1.444(6)	C22	C23	1.371(8)
O3	C9	1.364(8)	C9	C10	1.509(10)
O4	C11	1.349(6)	C32	C31	1.385(9)
O4	C4	1.449(6)	C29	C28	1.374(9)
O1	C1	1.414(5)	C29	C30	1.371(11)
O1	C5	1.436(5)	C16	C17	1.383(9)
O6	C7	1.181(7)	C20	C19	1.403(9)
O8	C11	1.188(7)	C13	C14	1.517(12)
O5	C6	1.461(7)	C17	C18	1.369(11)
O5	C13	1.293(9)	C19	C18	1.356(11)
O7	C9	1.183(8)	C24	C23	1.371(9)

Atom	Atom	Length/Å	Atom	Atom	Length/Å
C7	C8	1.483(8)	C24	C25	1.348(12)
C3	C2	1.507(6)	C30	C31	1.367(11)
C3	C4	1.509(7)	F2	C33	1.289(10)
C15	C16	1.385(8)	F3	C33	1.358(12)
C15	C20	1.380(8)	C26	C25	1.404(11)

Table S4 Bond Angles for 2a′.

Atom	Atom	Atom	Angle/°	Atom	Atom	Atom	Angle/°
C15	P1	C1	106.3(2)	O4	C11	C12	109.9(5)
C15	P1	C21	113.4(2)	O8	C11	O4	122.8(5)
C21	P1	C1	111.6(2)	O8	C11	C12	127.3(6)
C27	P1	C15	110.2(2)	C32	C27	P1	120.7(4)
C27	P1	C1	107.8(2)	C28	C27	P1	119.3(4)
C27	P1	C21	107.5(2)	C28	C27	C32	119.7(5)
O11	S1	O10	116.1(4)	O4	C4	C3	107.2(4)
O11	S1	C33	103.6(4)	O4	C4	C5	110.8(4)
O12	S1	O11	114.8(4)	C3	C4	C5	108.0(4)
O12	S1	O10	113.5(3)	C23	C22	C21	120.8(5)
O12	S1	C33	104.1(4)	O3	C9	C10	109.4(7)
O10	S1	C33	102.6(4)	O7	C9	O3	122.7(6)
C7	O2	C2	118.1(4)	O7	C9	C10	127.9(6)
C9	O3	C3	116.5(5)	C31	C32	C27	118.8(6)
C11	O4	C4	117.4(4)	C30	C29	C28	119.8(7)
C1	O1	C5	114.2(4)	O5	C6	C5	108.1(5)
C13	O5	C6	115.1(6)	C17	C16	C15	120.1(6)
O2	C7	C8	110.0(5)	C29	C28	C27	120.3(6)
O6	C7	O2	123.0(5)	C15	C20	C19	119.0(6)
O6	C7	C8	127.0(5)	O5	C13	C14	111.0(8)
O3	C3	C2	104.9(4)	O9	C13	O5	125.8(8)
O3	C3	C4	111.2(4)	O9	C13	C14	123.2(8)
C2	C3	C4	112.0(4)	C18	C17	C16	120.1(6)
C16	C15	P1	121.4(4)	C18	C19	C20	120.7(7)
C20	C15	P1	118.4(4)	C25	C24	C23	119.5(6)
C20	C15	C16	119.7(5)	C22	C23	C24	120.2(6)
O2	C2	C3	107.1(3)	C31	C30	C29	120.5(6)
O2	C2	C1	107.0(3)	C19	C18	C17	120.3(6)
C3	C2	C1	110.1(4)	C30	C31	C32	120.8(7)
O1	C1	P1	99.6(3)	C21	C26	C25	120.0(7)
O1	C1	C2	112.4(3)	F1	C33	S1	112.9(6)
C2	C1	P1	113.5(3)	F1	C33	F3	105.8(7)
C22	C21	P1	119.1(4)	F2	C33	S1	111.6(6)
C26	C21	P1	121.3(5)	F2	C33	F1	108.3(10)
C26	C21	C22	119.0(6)	F2	C33	F3	109.2(9)
O1	C5	C4	108.8(3)	F3	C33	S1	108.9(8)
O1	C5	C6	107.2(4)	C24	C25	C26	120.6(7)
C6	C5	C4	113.2(4)				

12.Copies of ^{31}P NMR, ^{1}H NMR, ^{13}C NMR Spectra of Compounds 2.

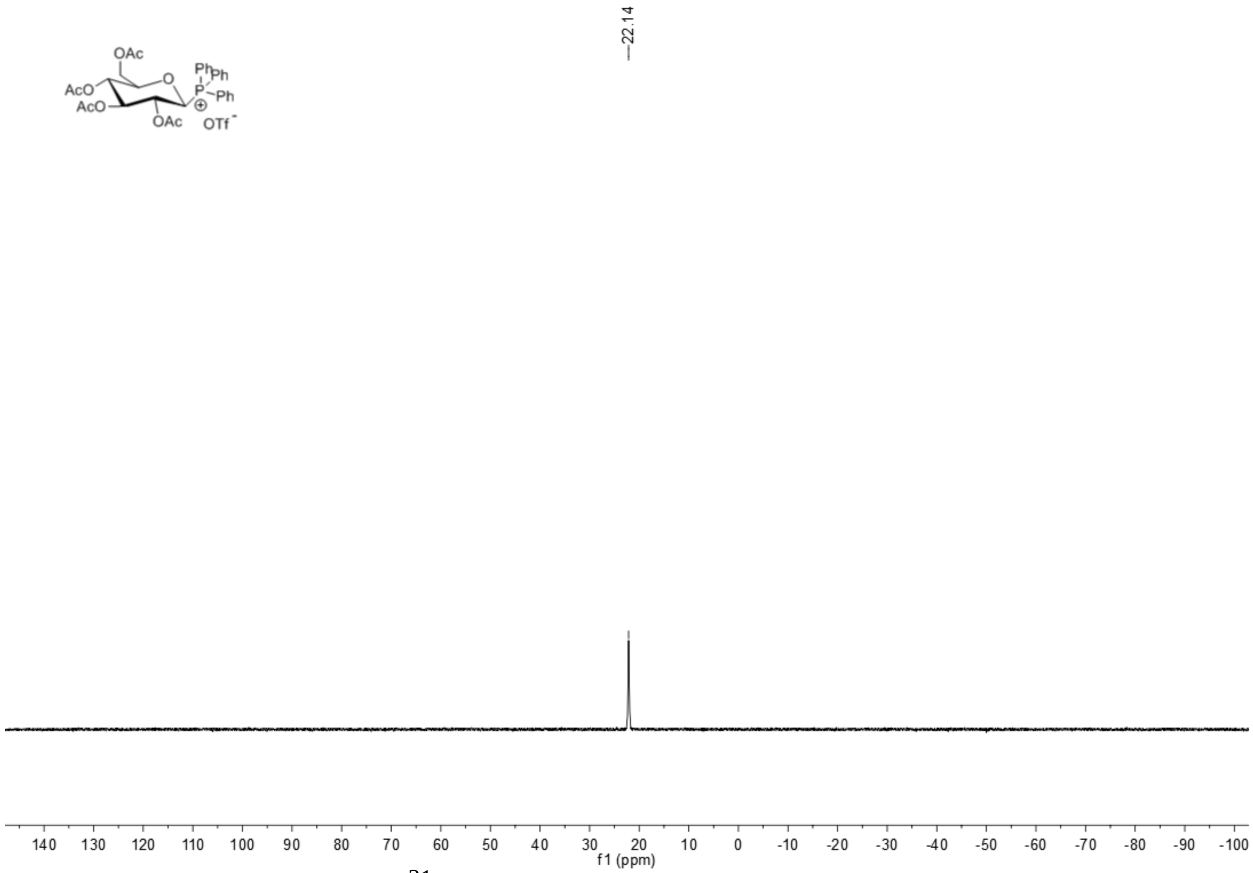

^{31}P NMR (CDCl$_3$-*d*) of **2a**

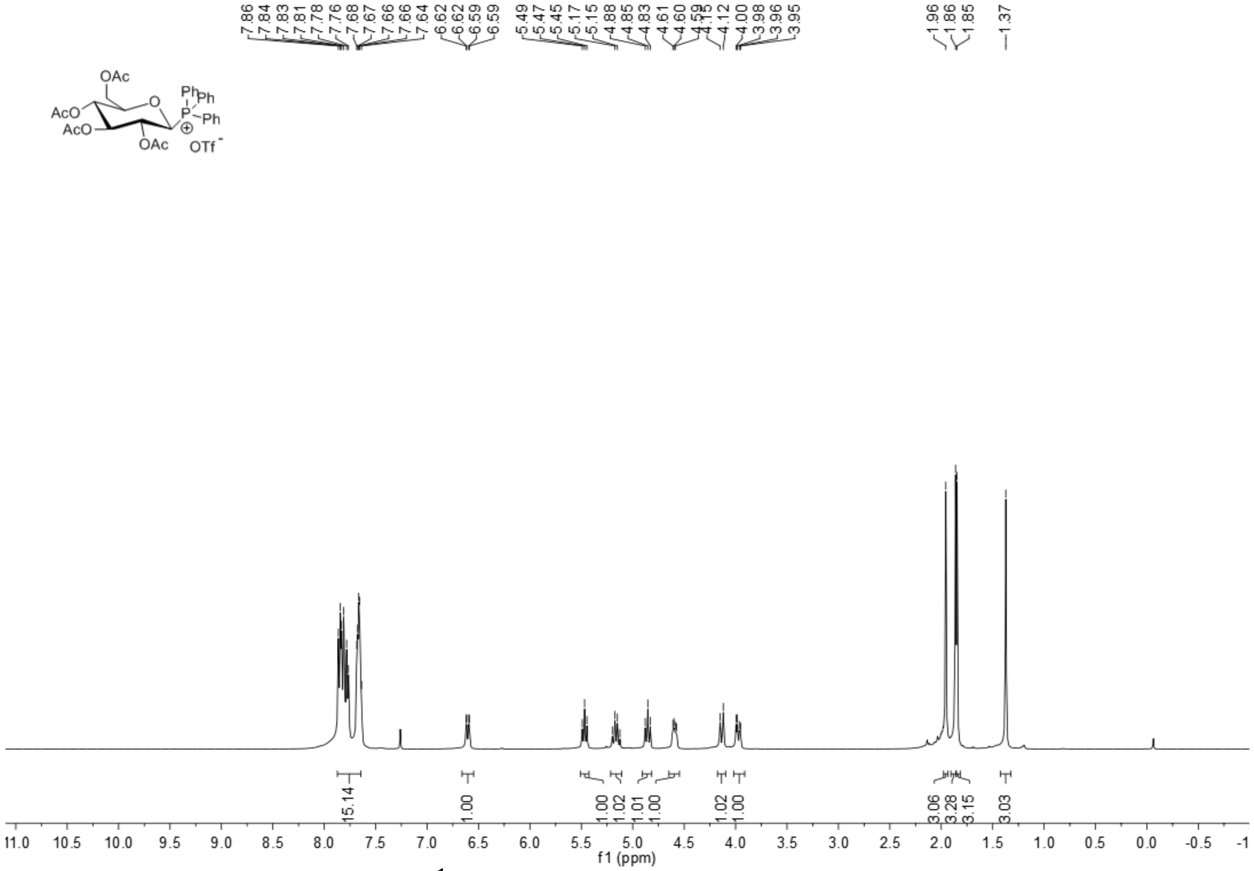

^{1}H NMR (CDCl$_3$-*d*) of **2a**

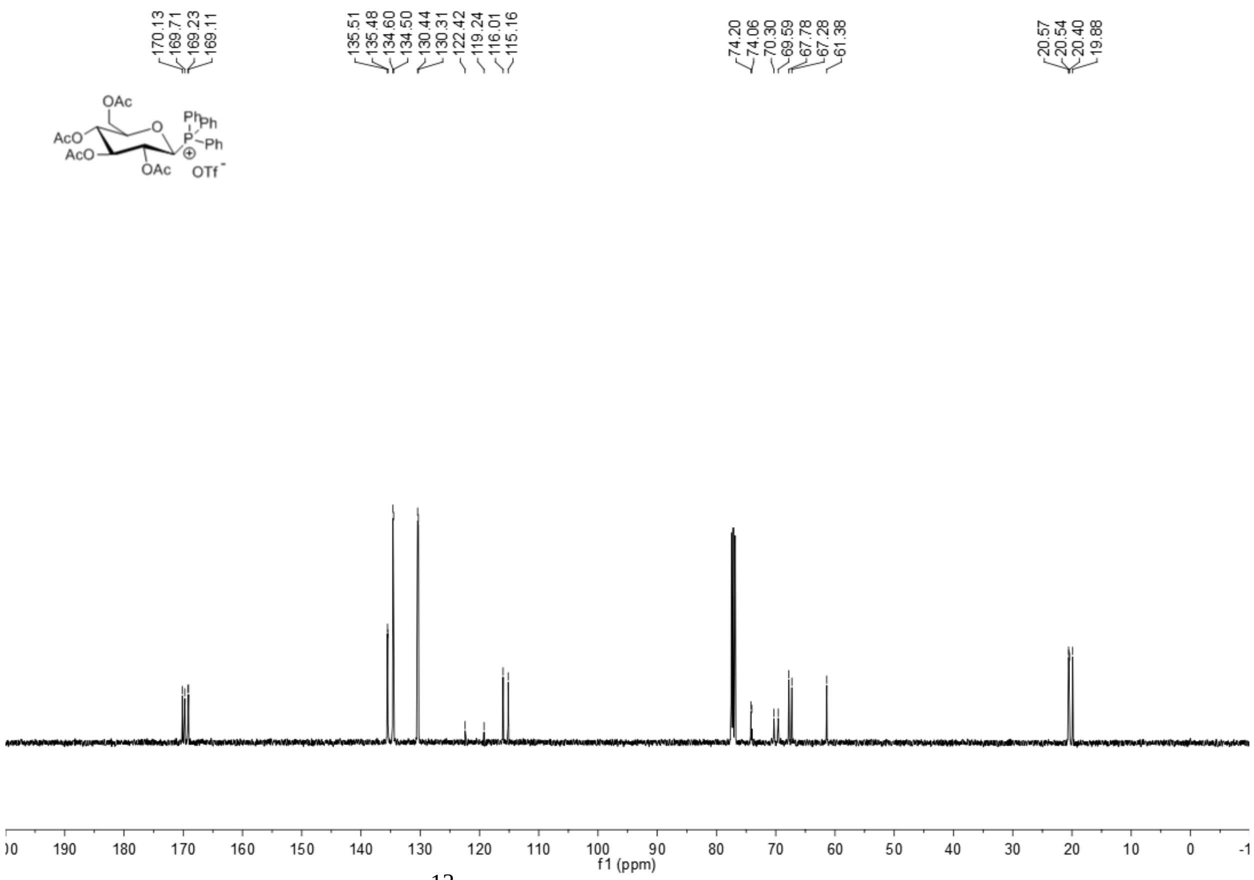

^{13}C NMR ($CDCl_3$-*d*) of **2a**

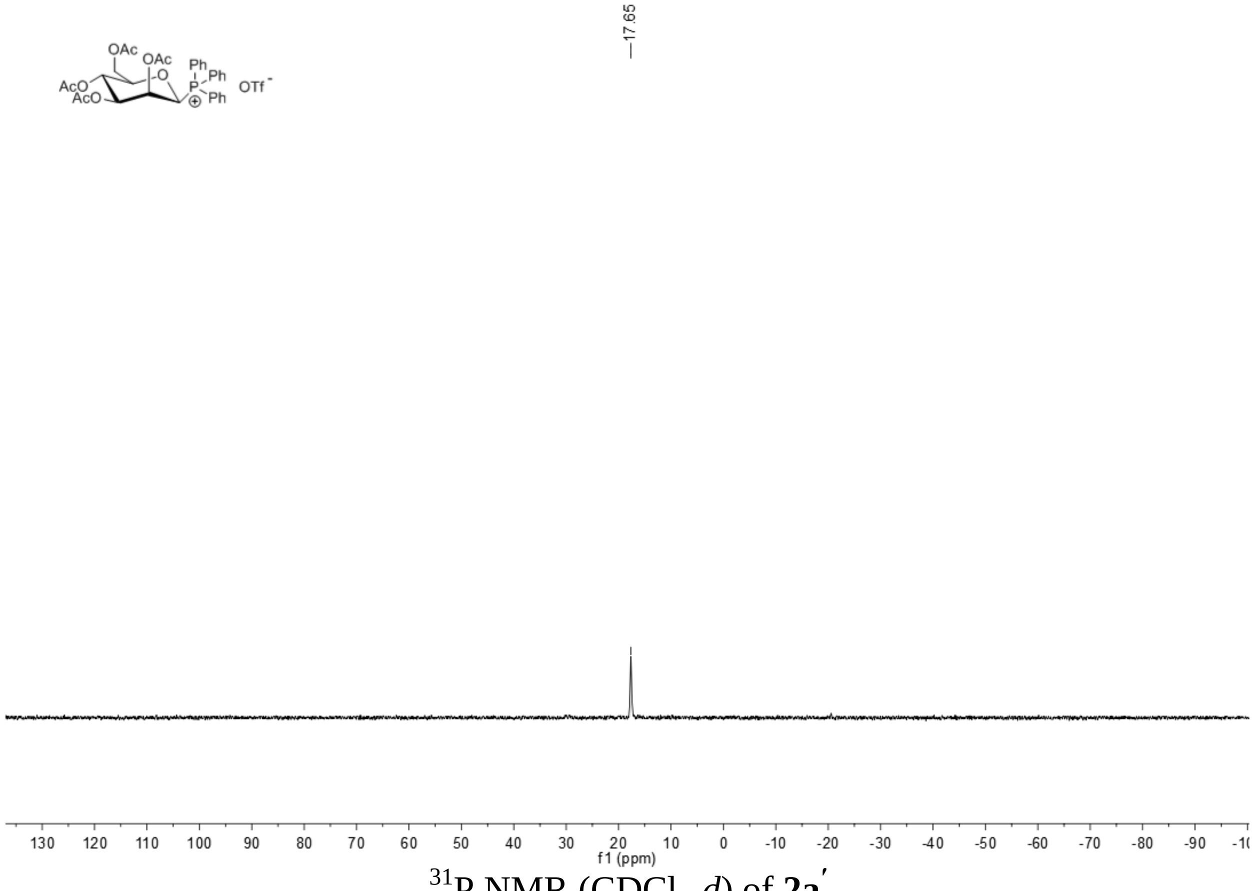

^{31}P NMR ($CDCl_3$-*d*) of **2a**′

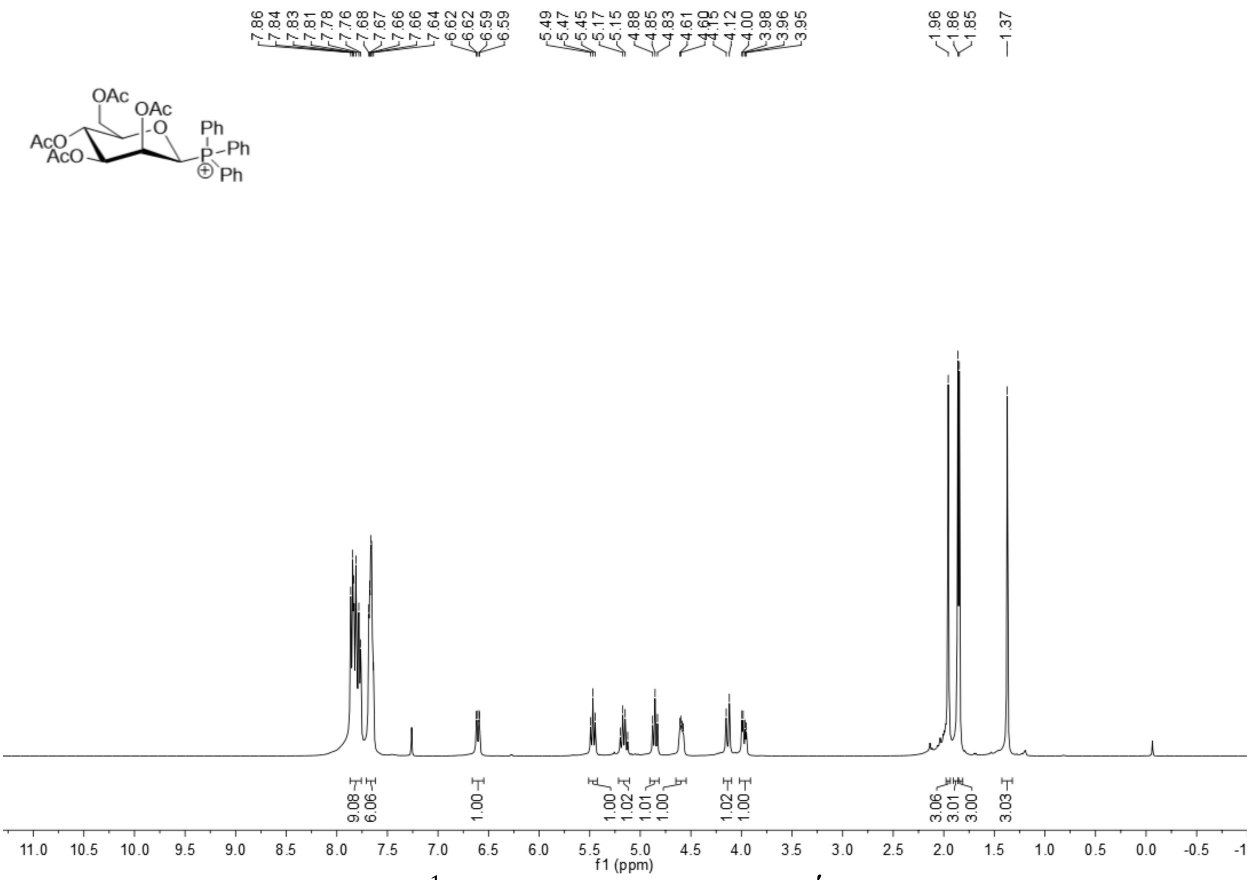

^{1}H NMR ($CDCl_3$-*d*) of **2a′**

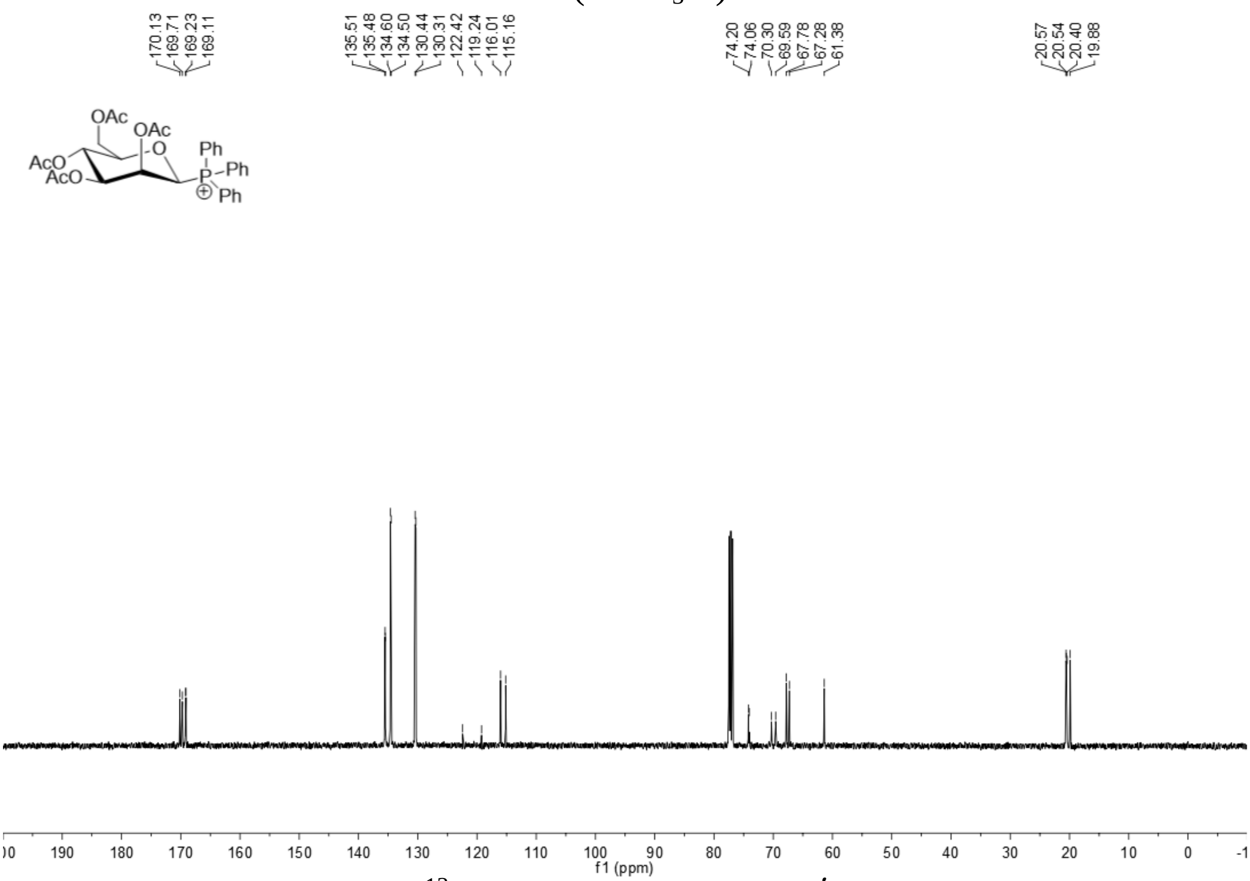

^{13}C NMR ($CDCl_3$-*d*) of **2a′**

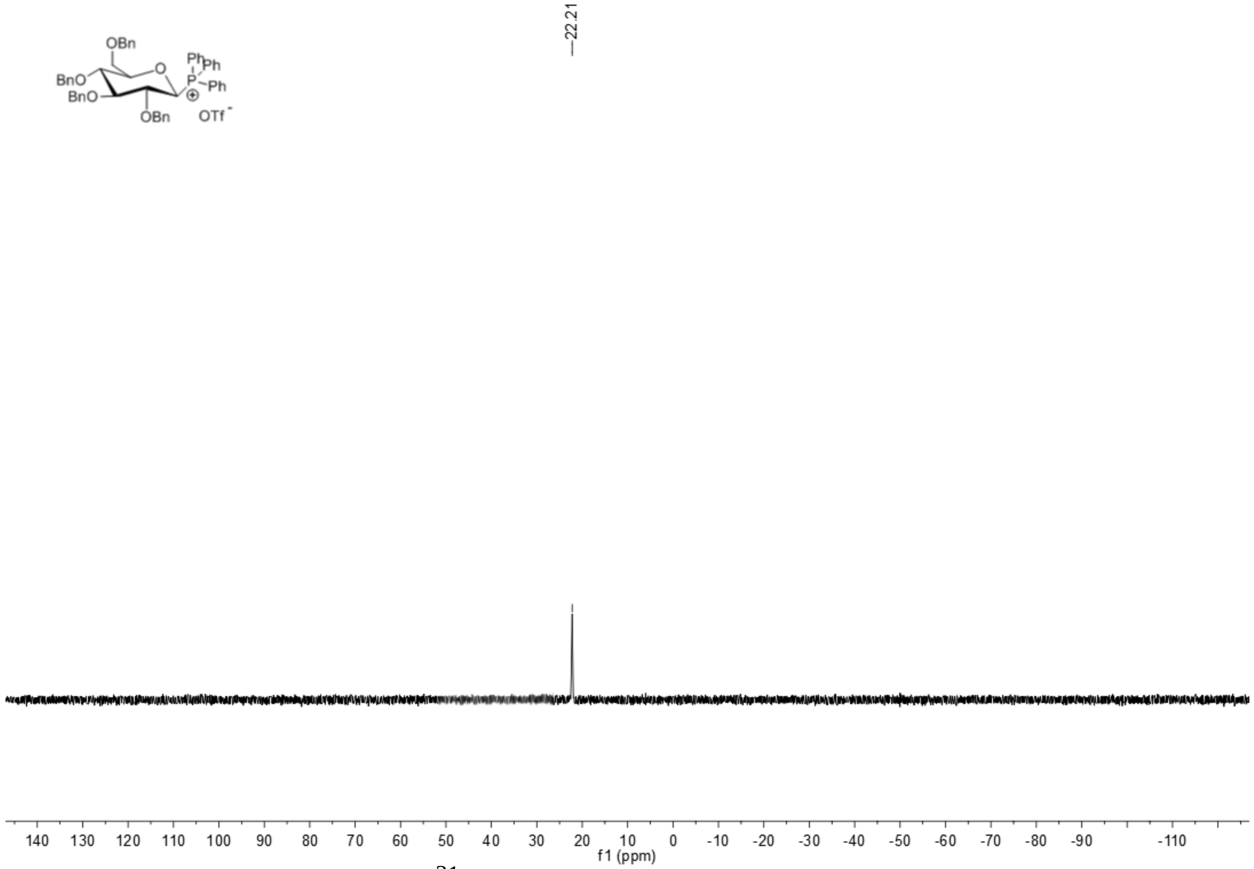

^{31}P NMR ($CDCl_3$-*d*) of **2f**

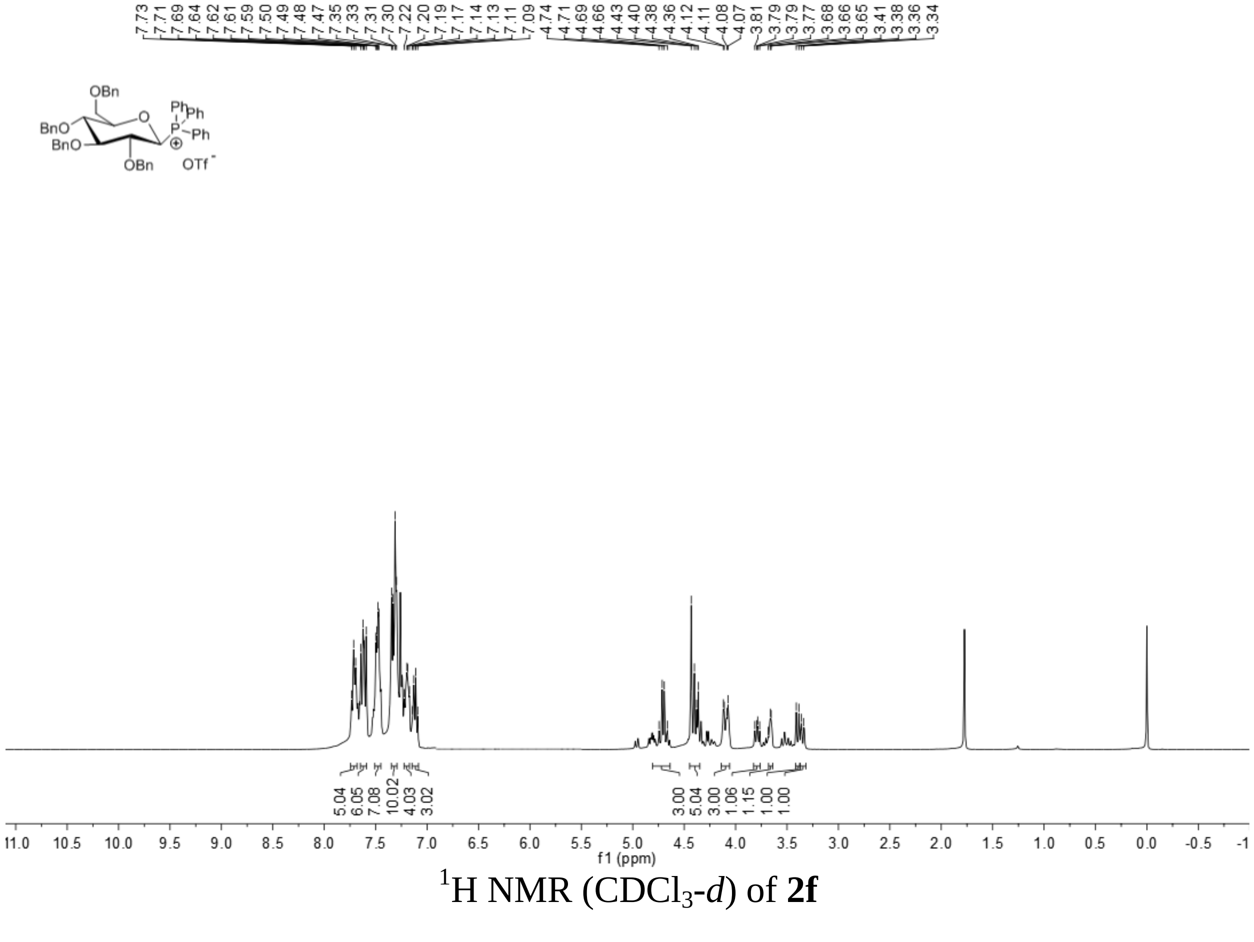

^{1}H NMR ($CDCl_3$-*d*) of **2f**

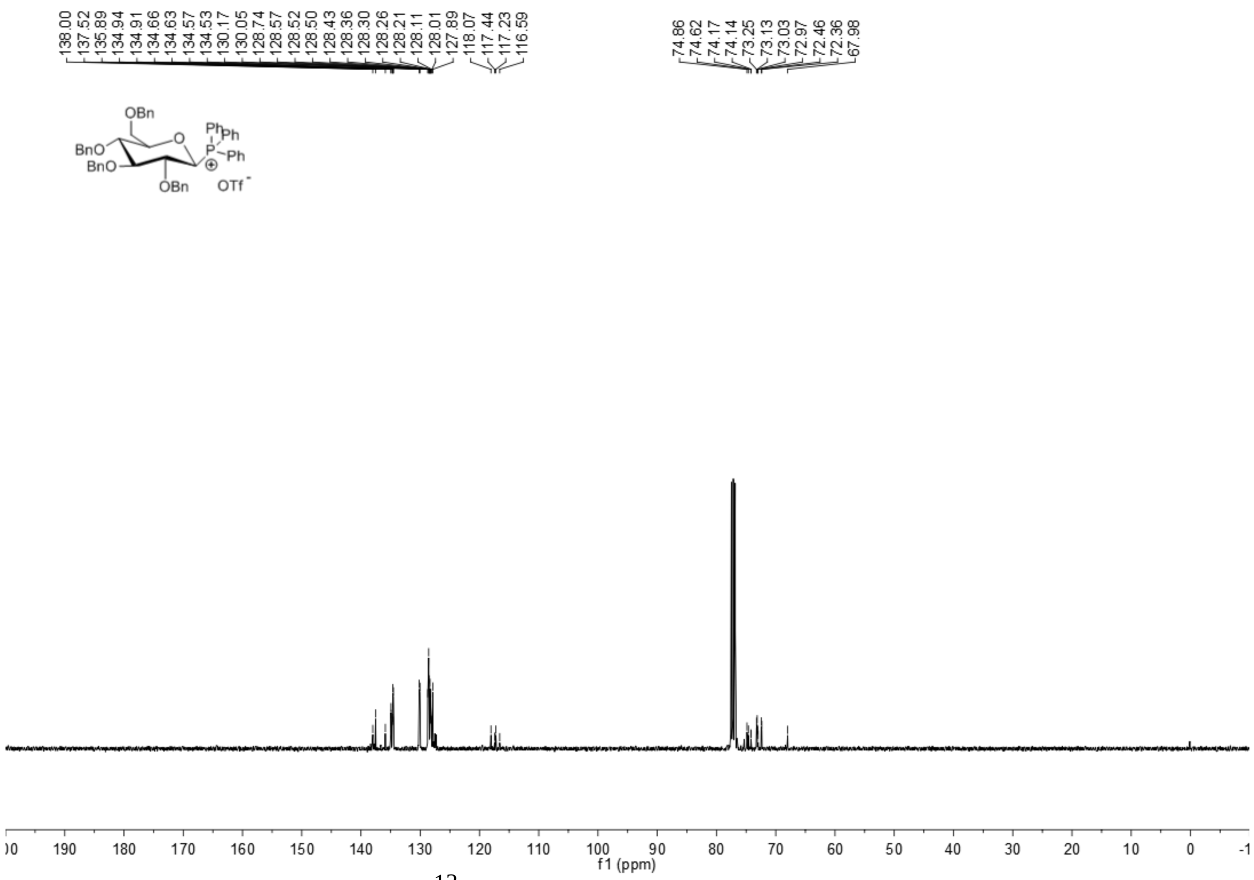

^{13}C NMR ($CDCl_3$-*d*) of **2f**

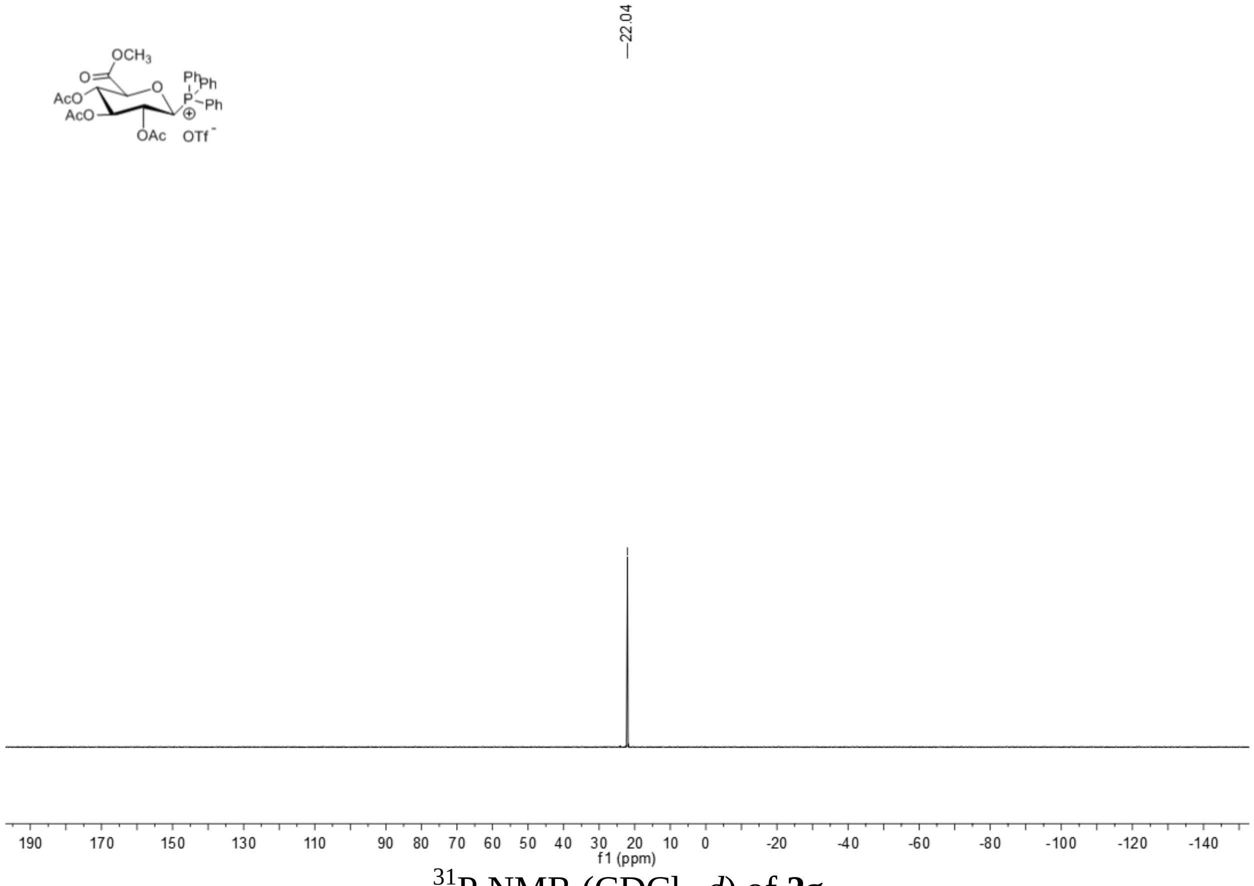

^{31}P NMR ($CDCl_3$-*d*) of **2g**

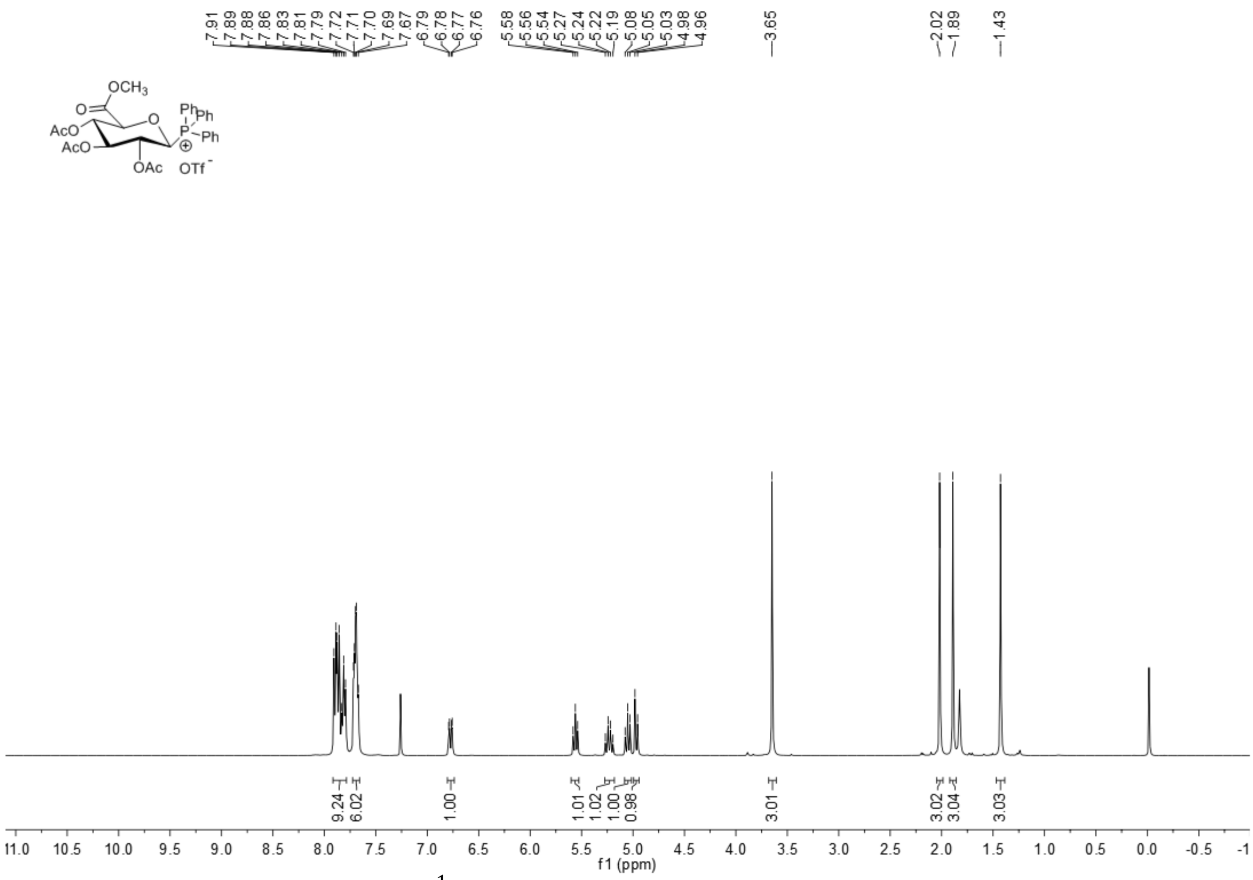

^{1}H NMR ($CDCl_3$-*d*) of **2g**

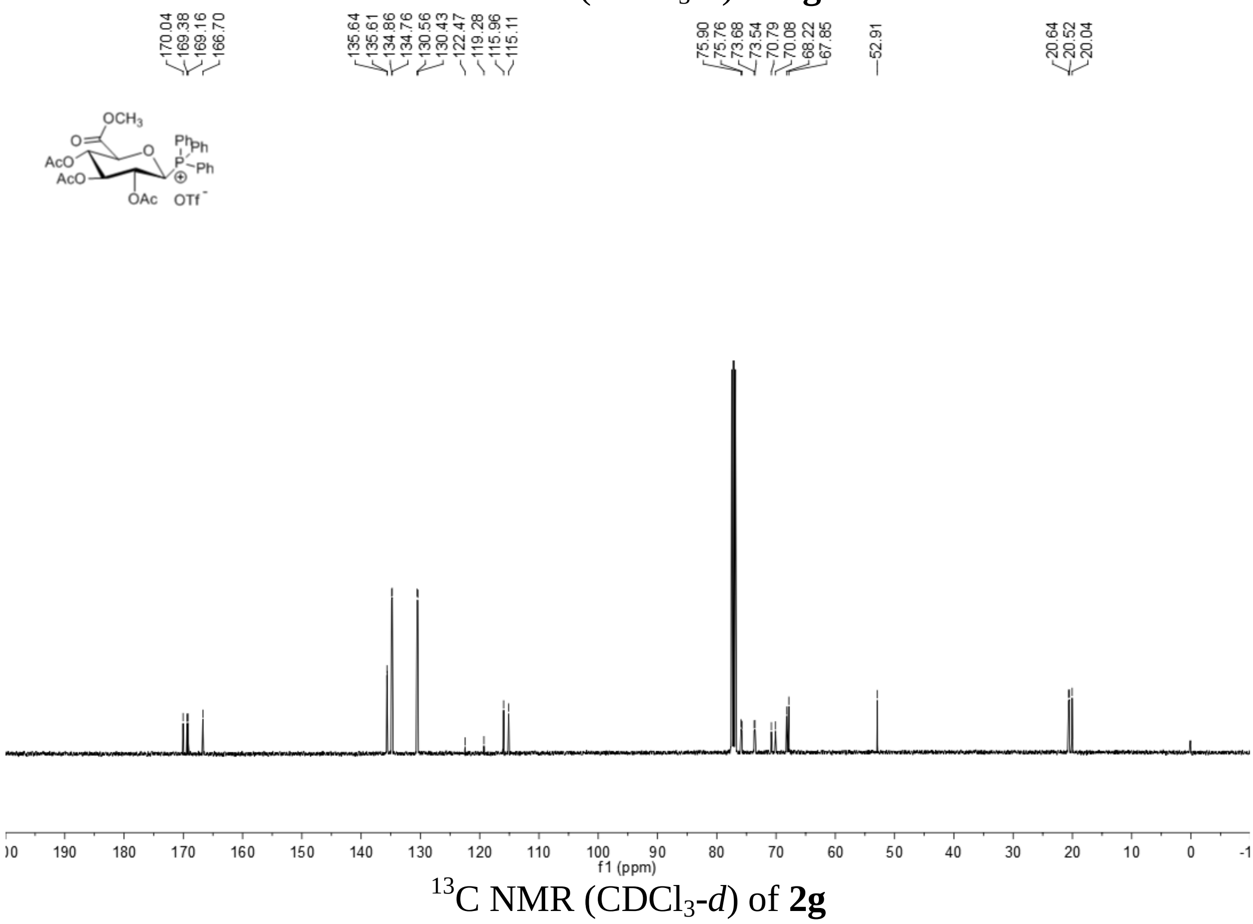

^{13}C NMR ($CDCl_3$-*d*) of **2g**

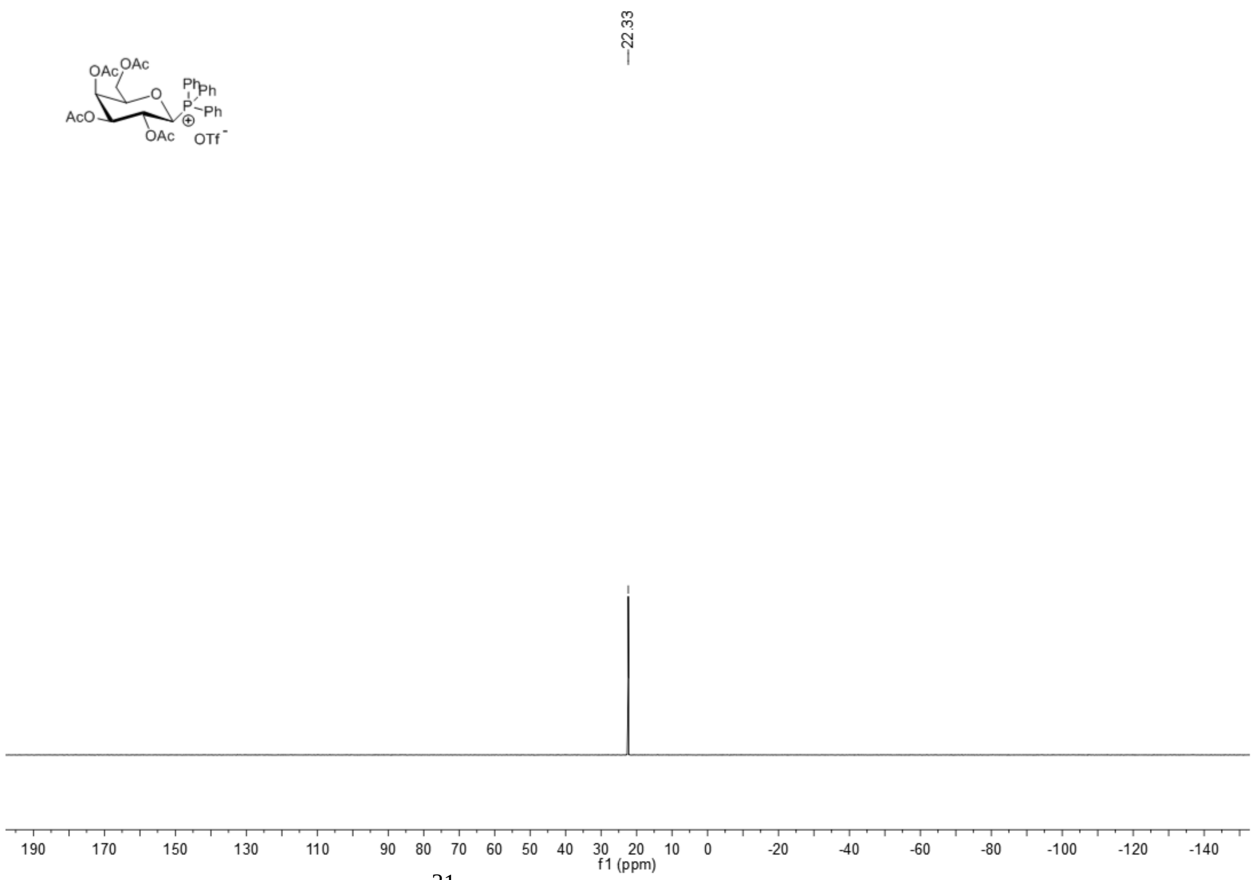

^{31}P NMR ($CDCl_3$-*d*) of **2h**

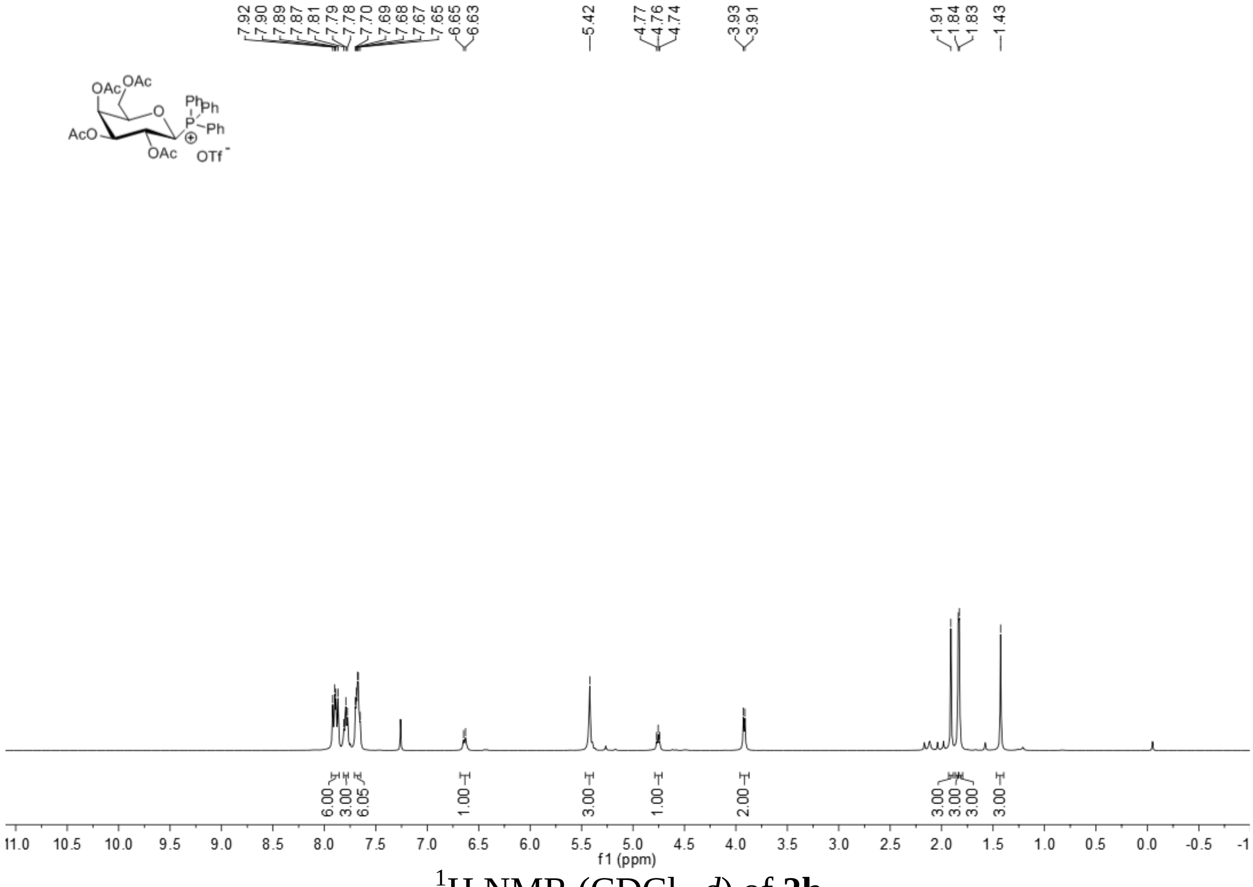

^{1}H NMR ($CDCl_3$-*d*) of **2h**

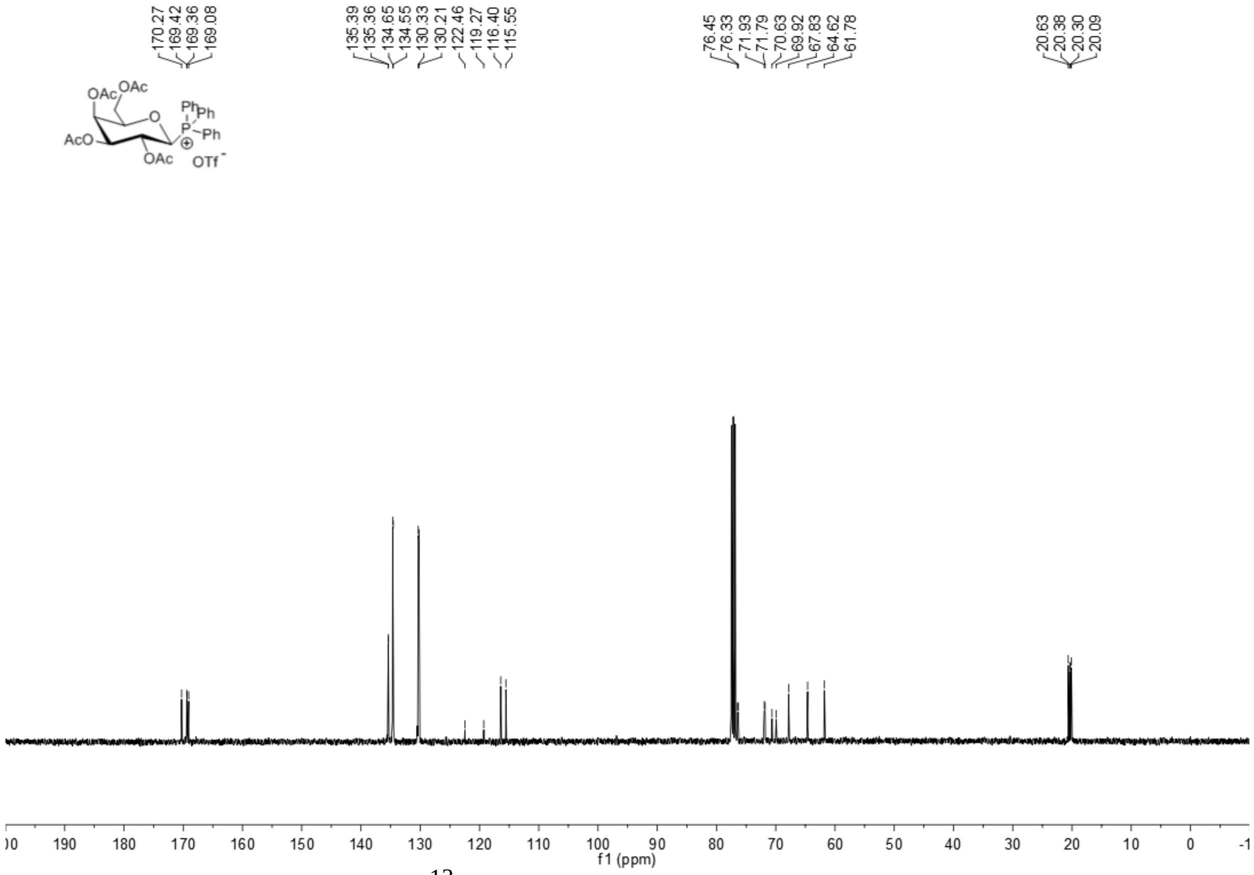

^{13}C NMR ($CDCl_3$-*d*) of **2h**

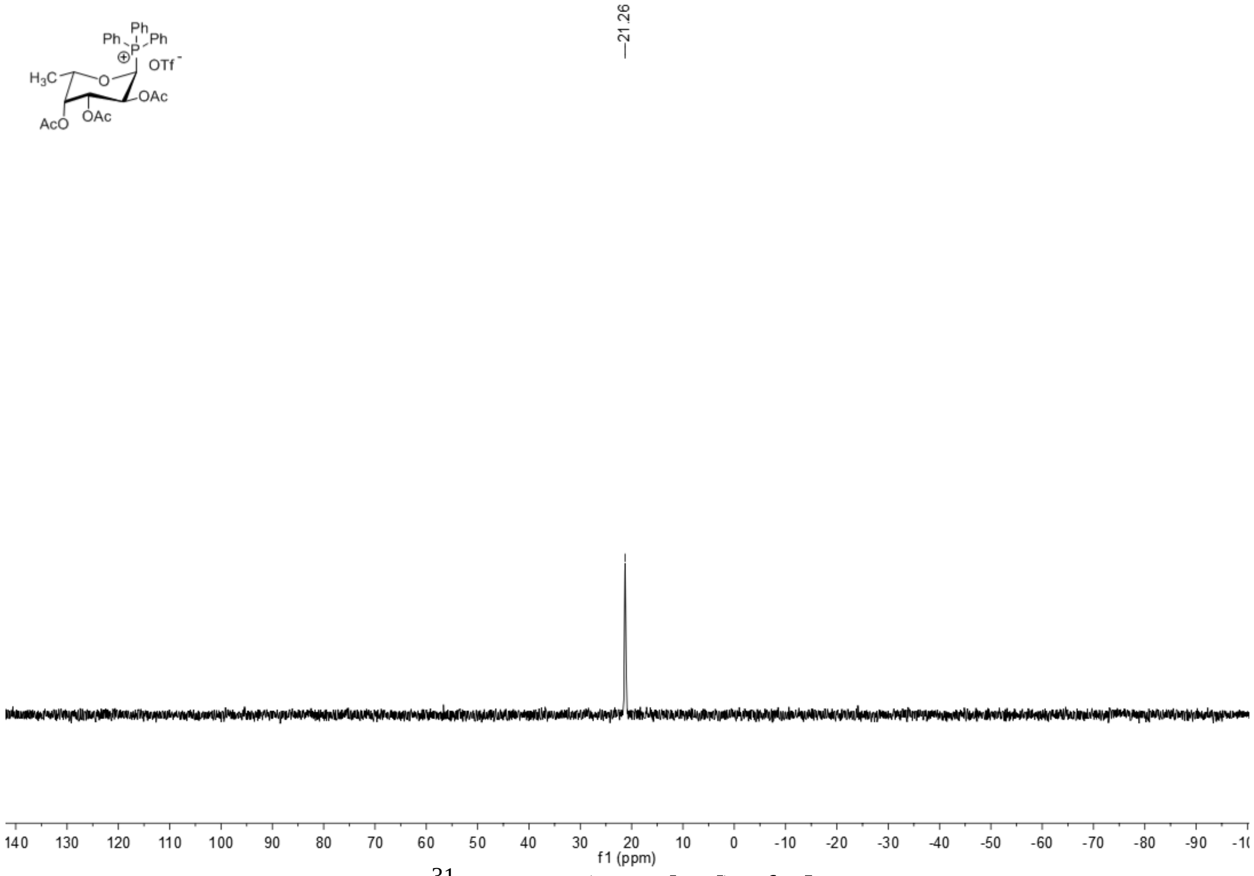

^{31}P NMR ($CDCl_3$-*d*) of **2k**

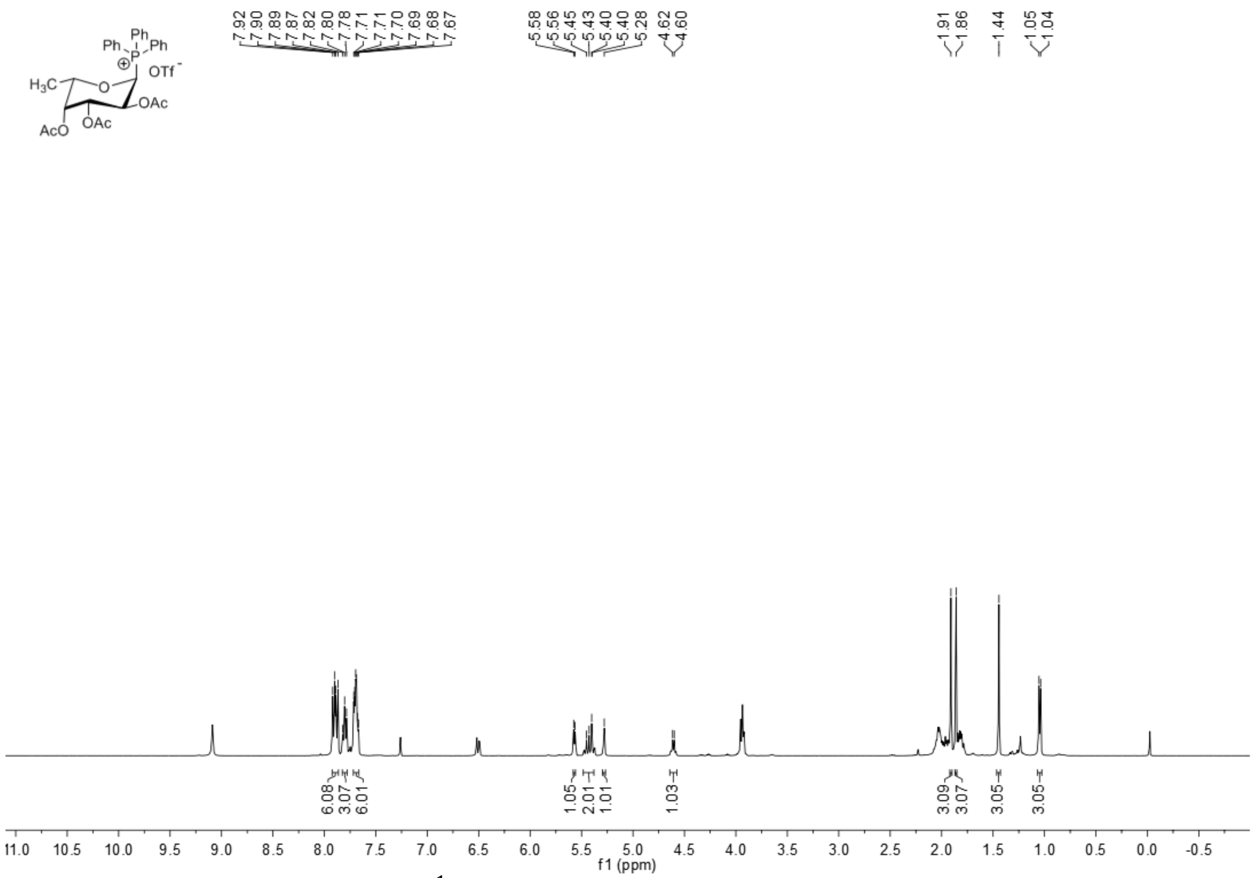

^{1}H NMR ($CDCl_3$-*d*) of **2k**

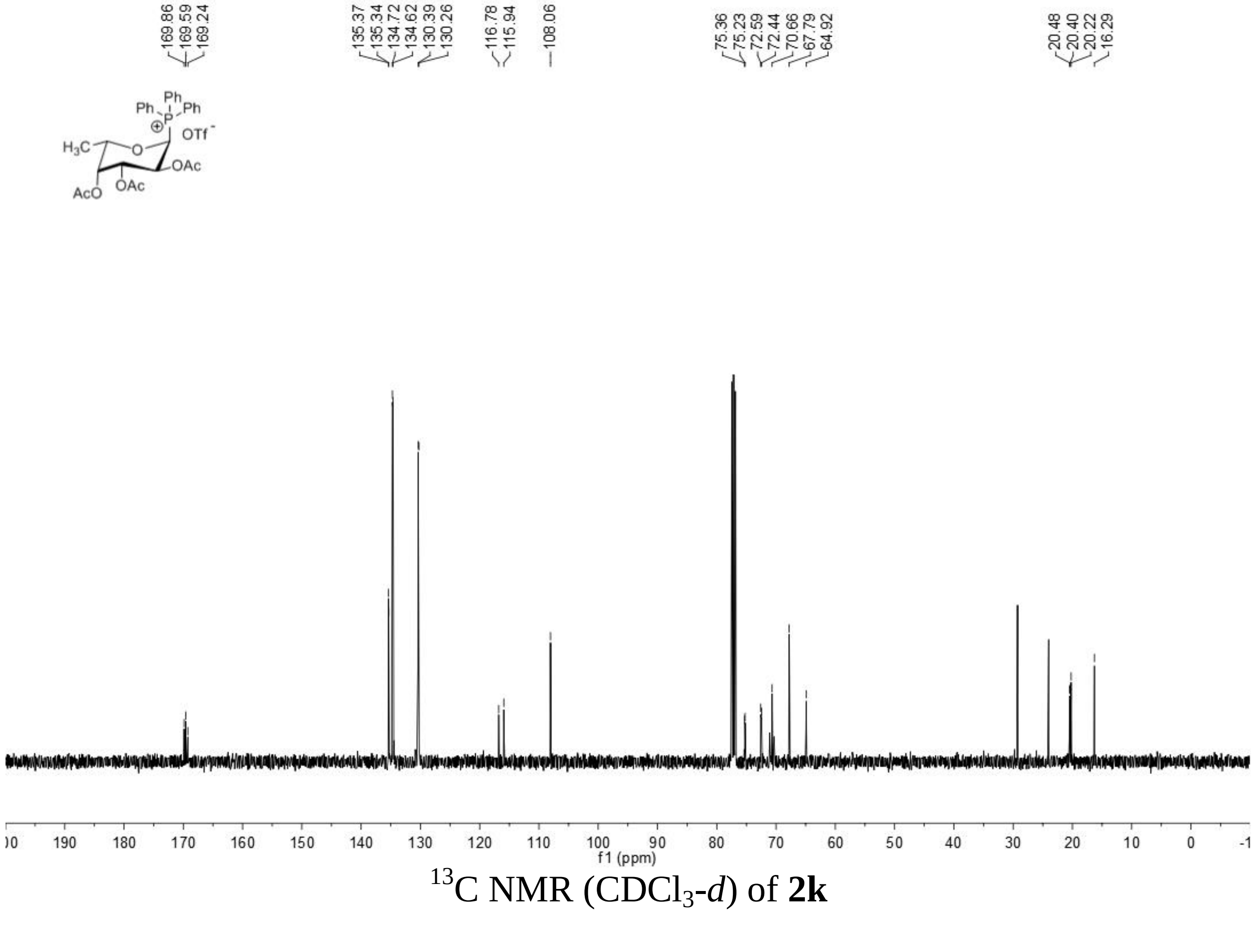

^{13}C NMR ($CDCl_3$-*d*) of **2k**

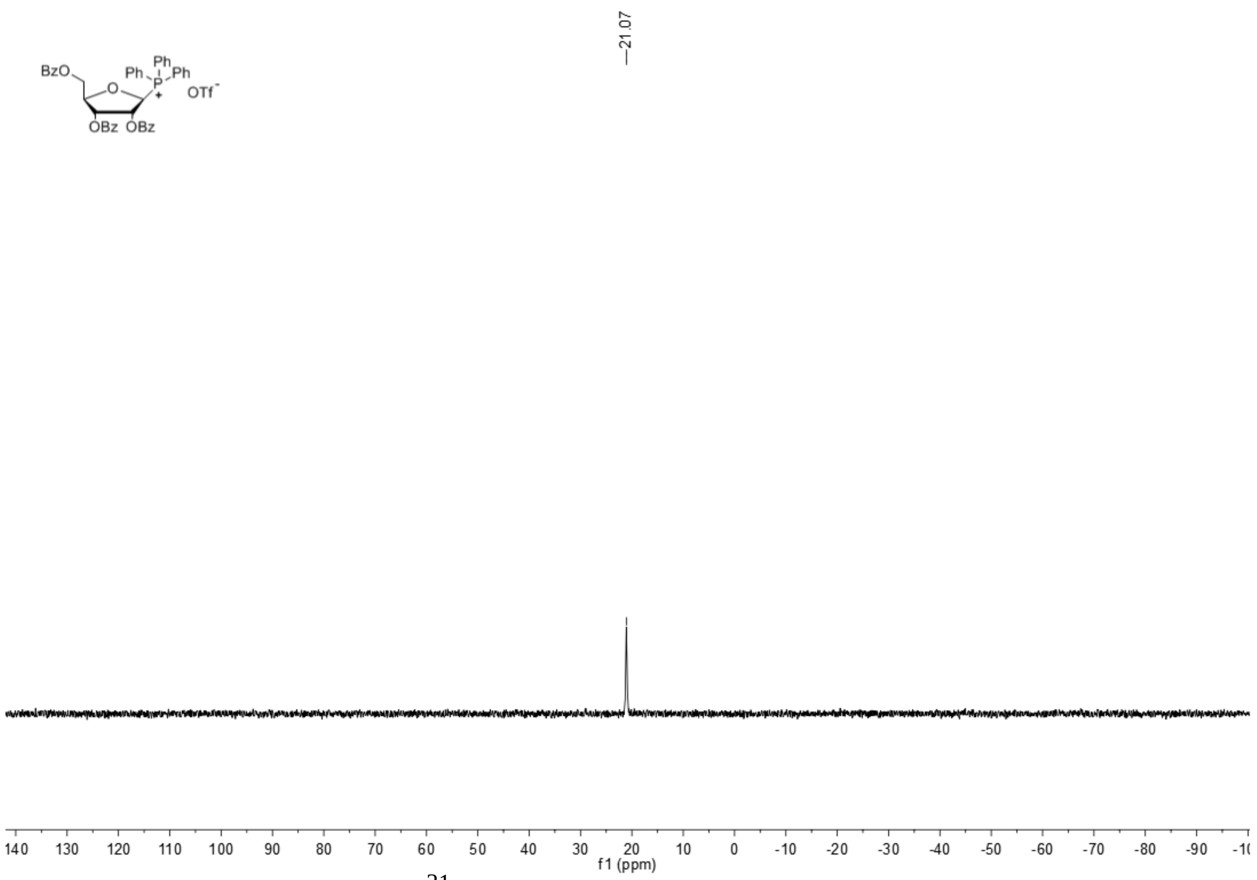

^{31}P NMR (CDCl$_3$-*d*) of **2m**

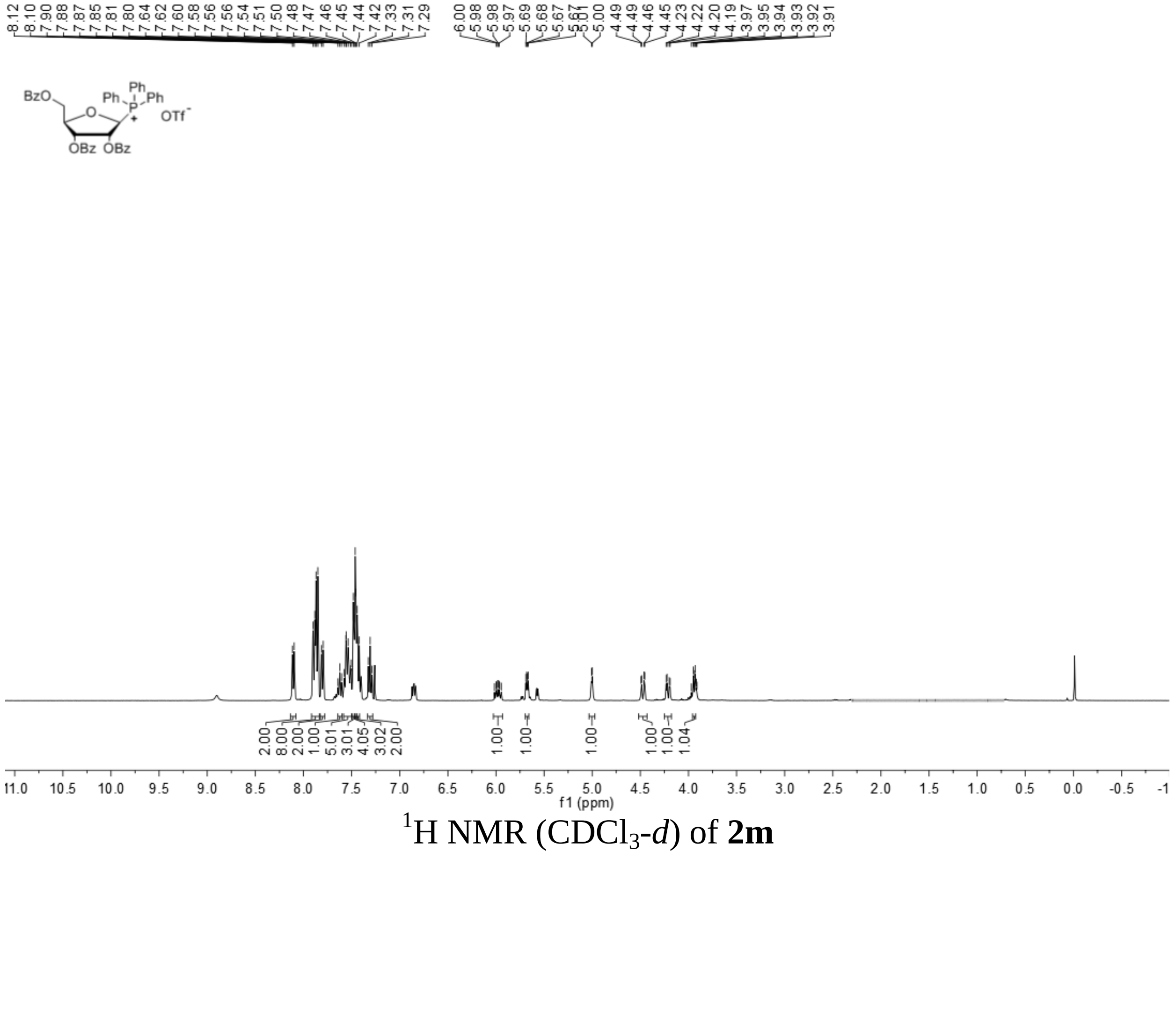

^{1}H NMR (CDCl$_3$-*d*) of **2m**

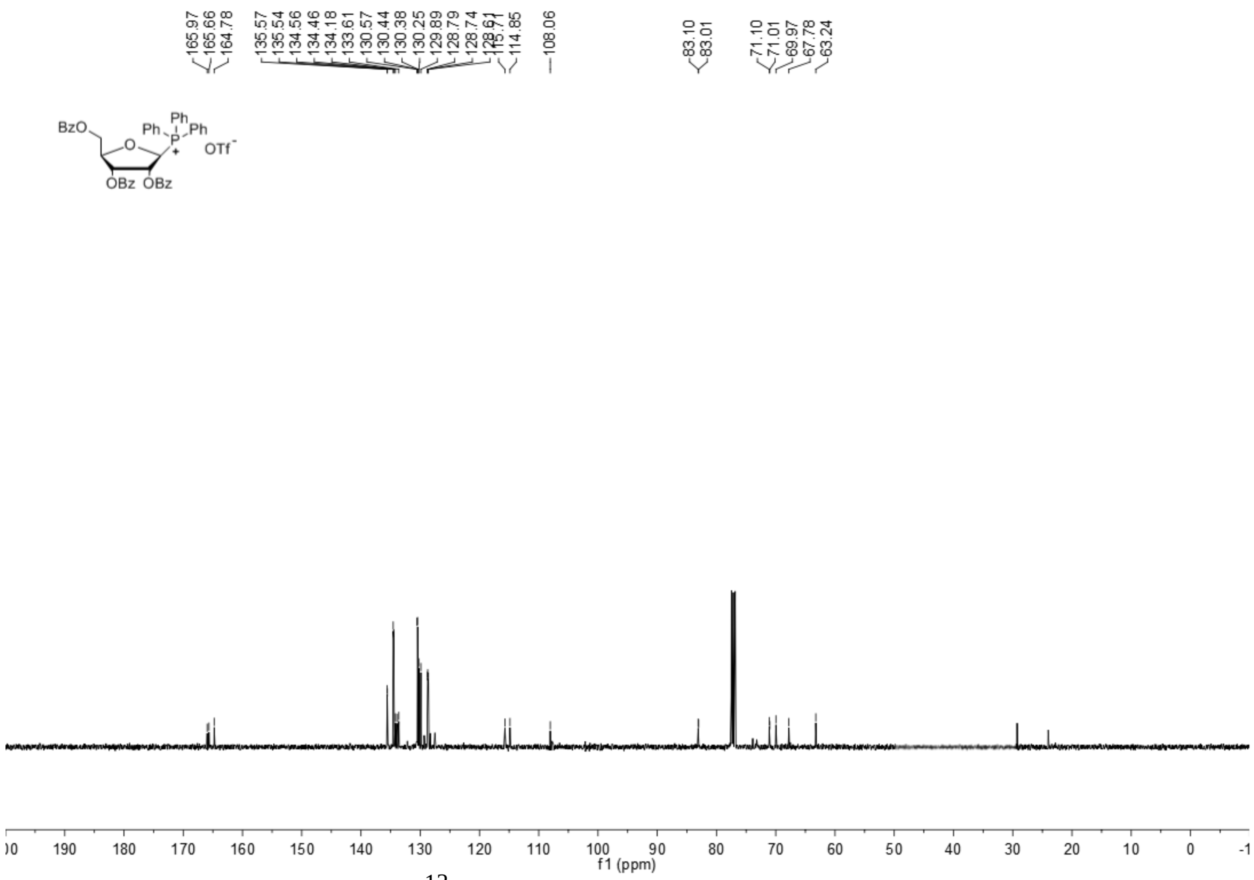

^{13}C NMR (CDCl$_3$-*d*) of **2m**

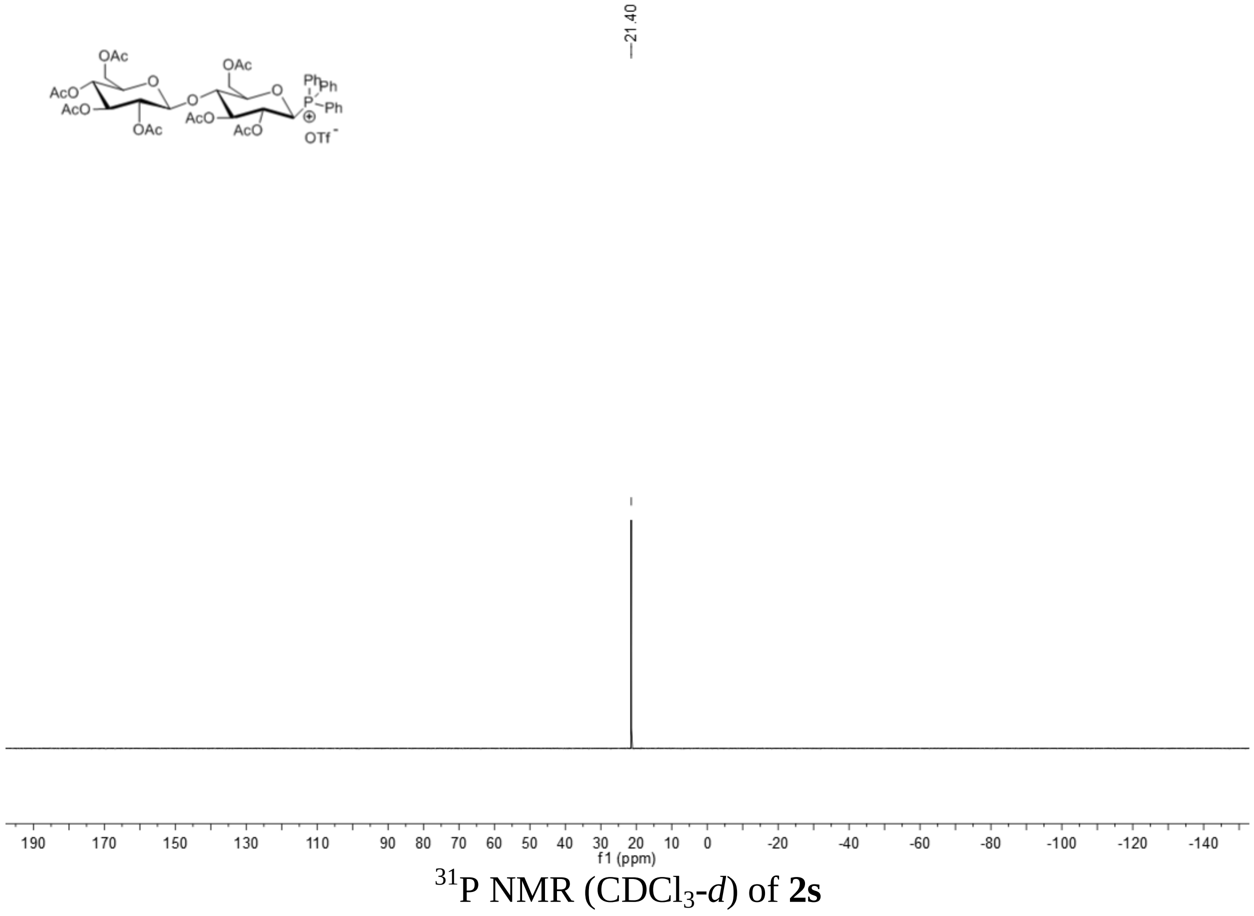

^{31}P NMR (CDCl$_3$-*d*) of **2s**

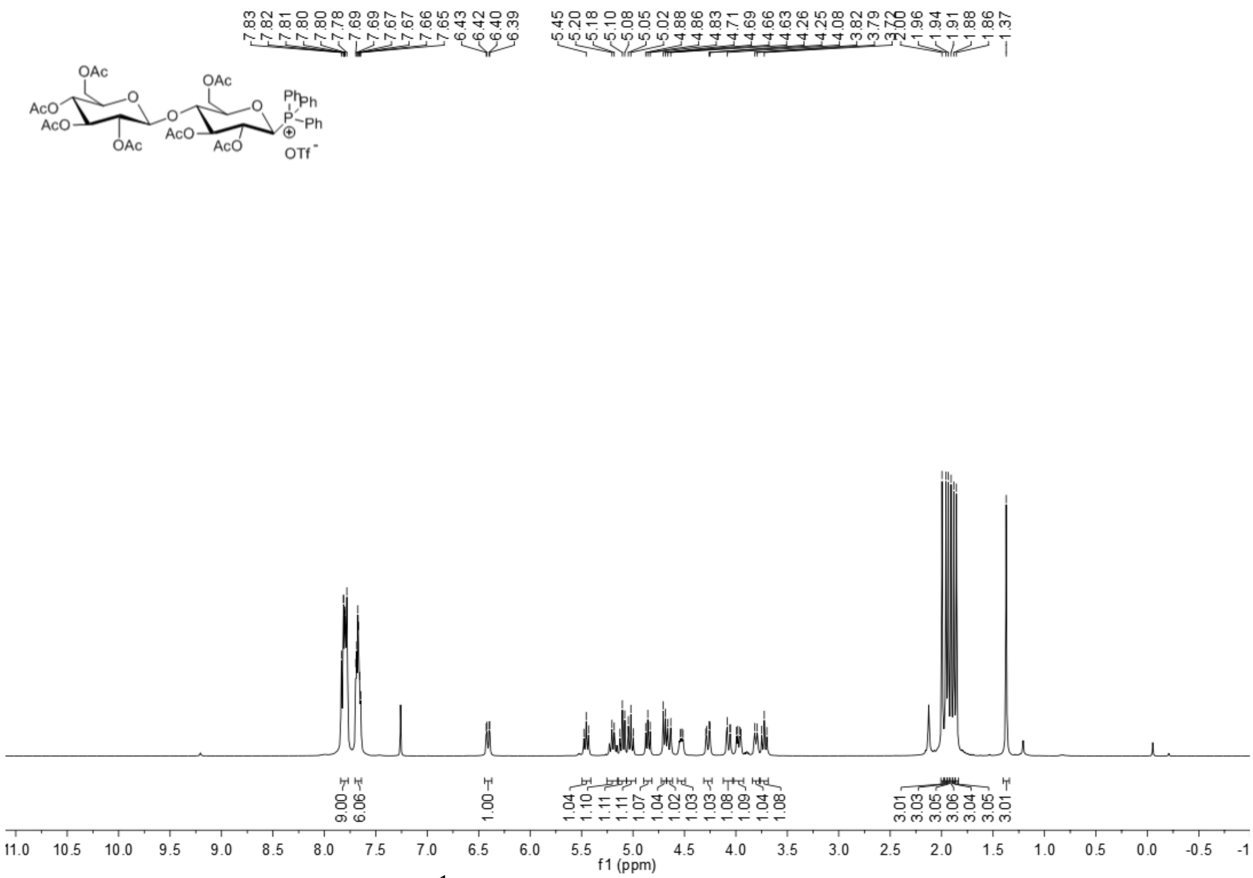

^{1}H NMR ($CDCl_3$-*d*) of **2s**

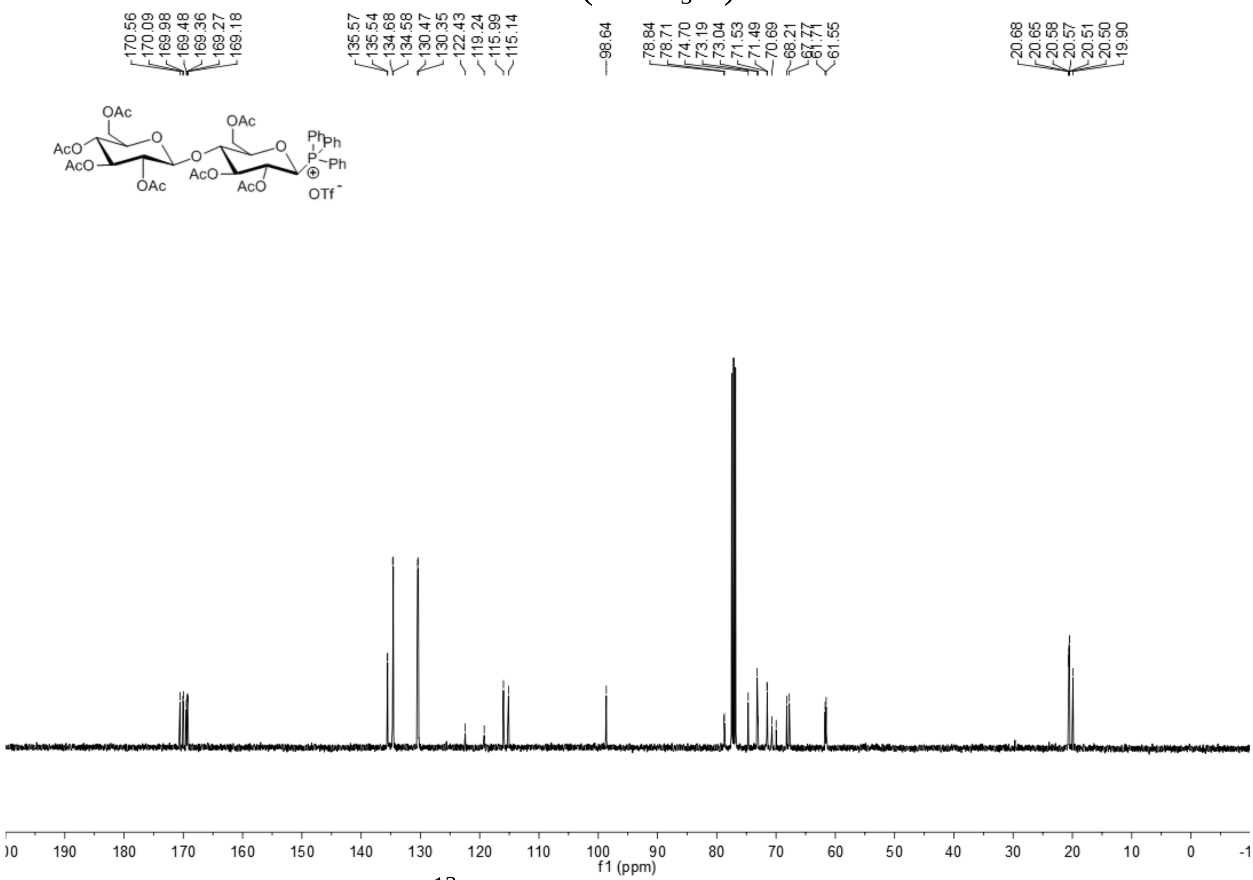

^{13}C NMR ($CDCl_3$-*d*) of **2s**

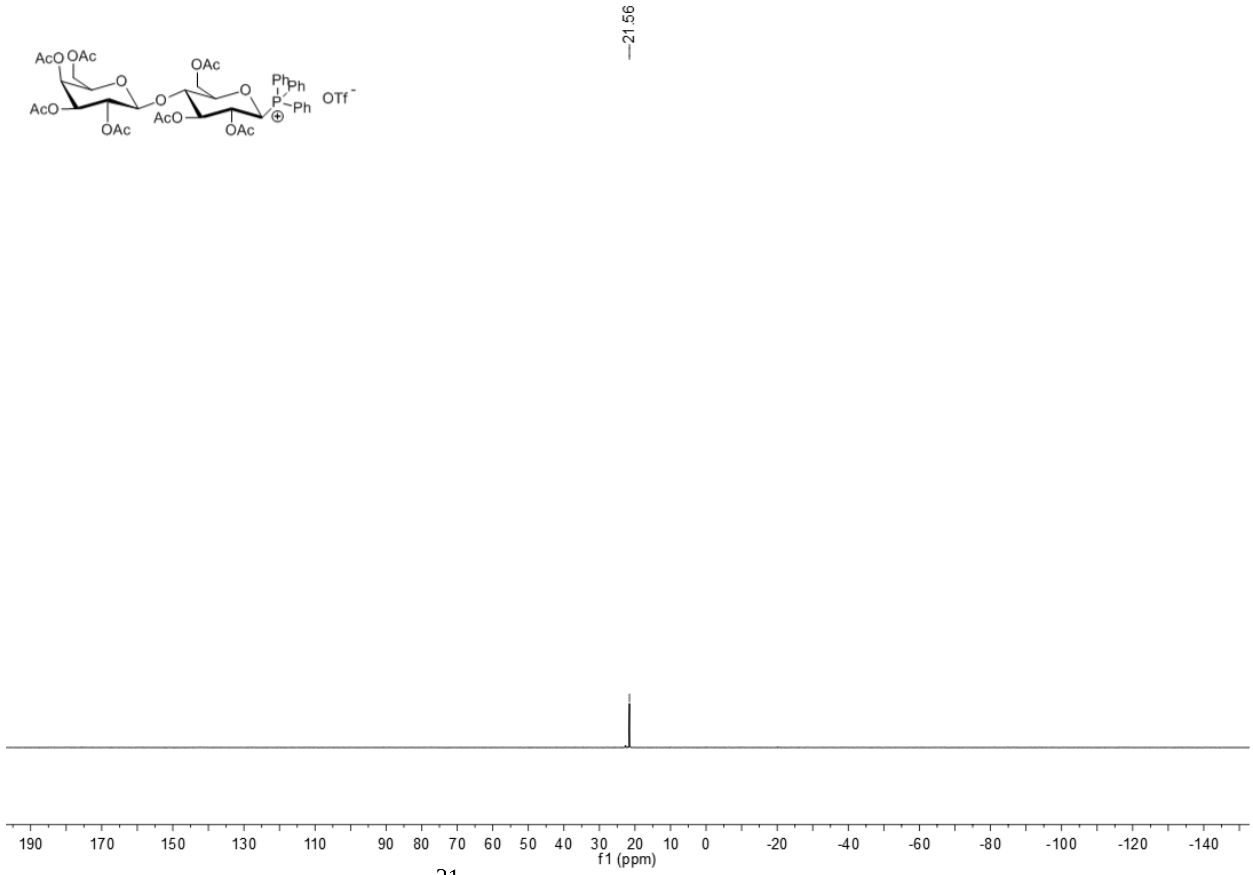

^{31}P NMR ($CDCl_3$-*d*) of **2t**

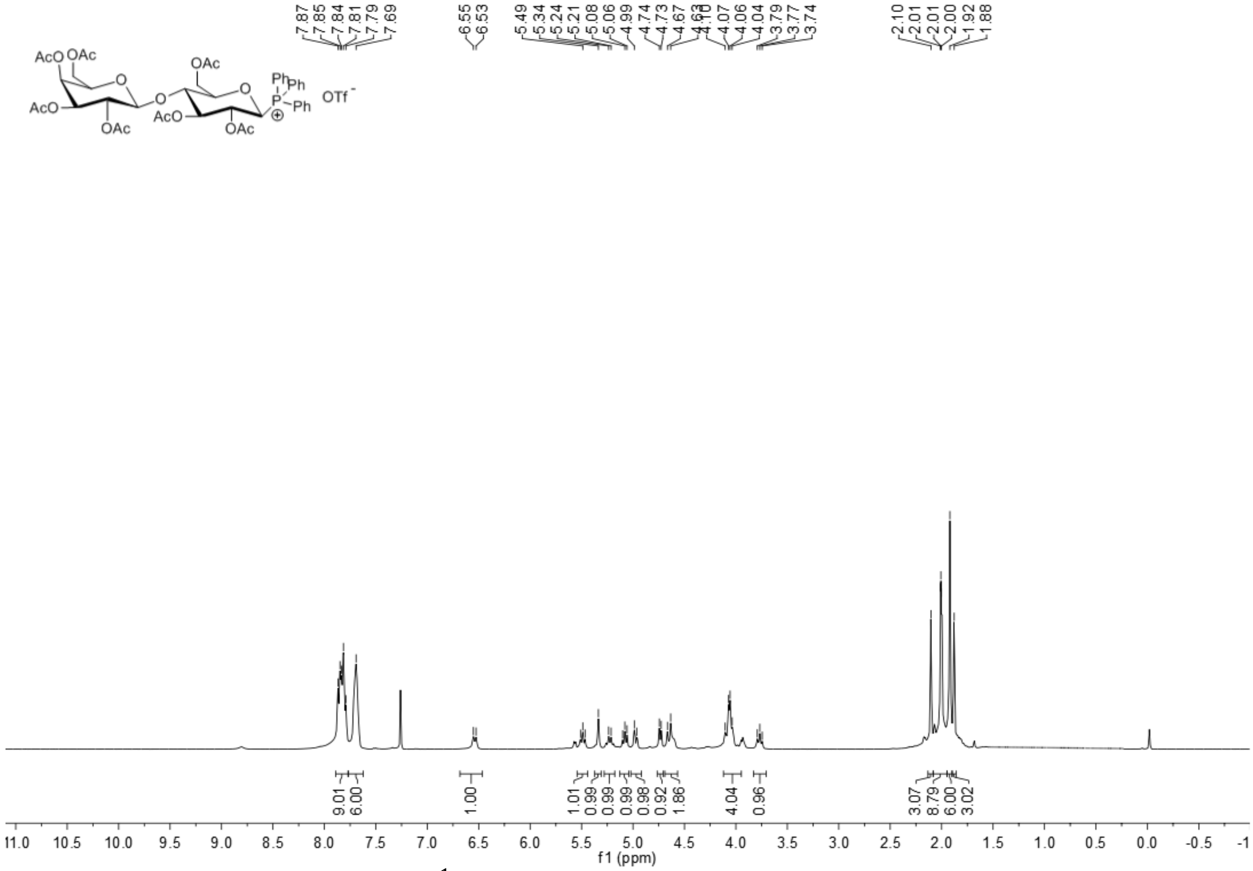

^{1}H NMR ($CDCl_3$-*d*) of **2t**

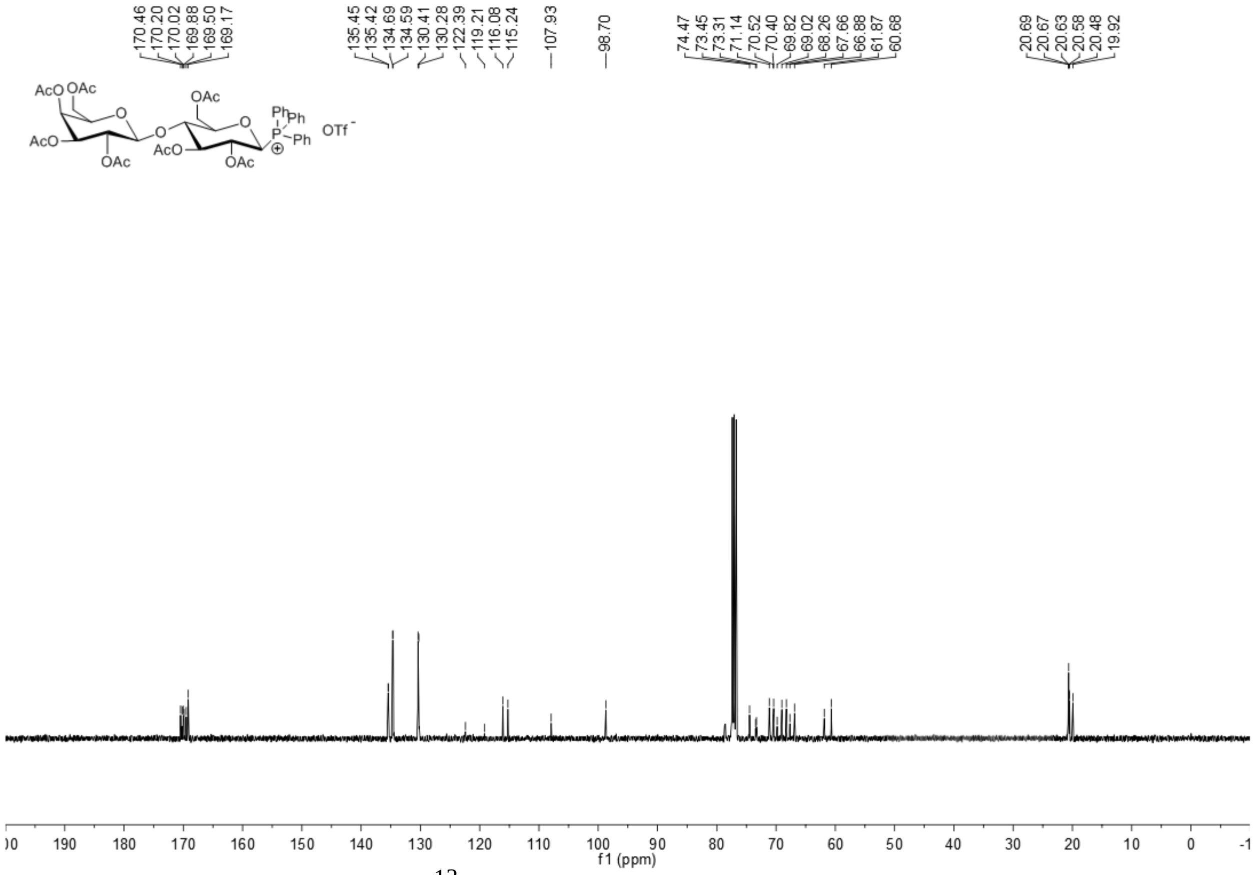

^{13}C NMR ($CDCl_3$-*d*) of **2t**

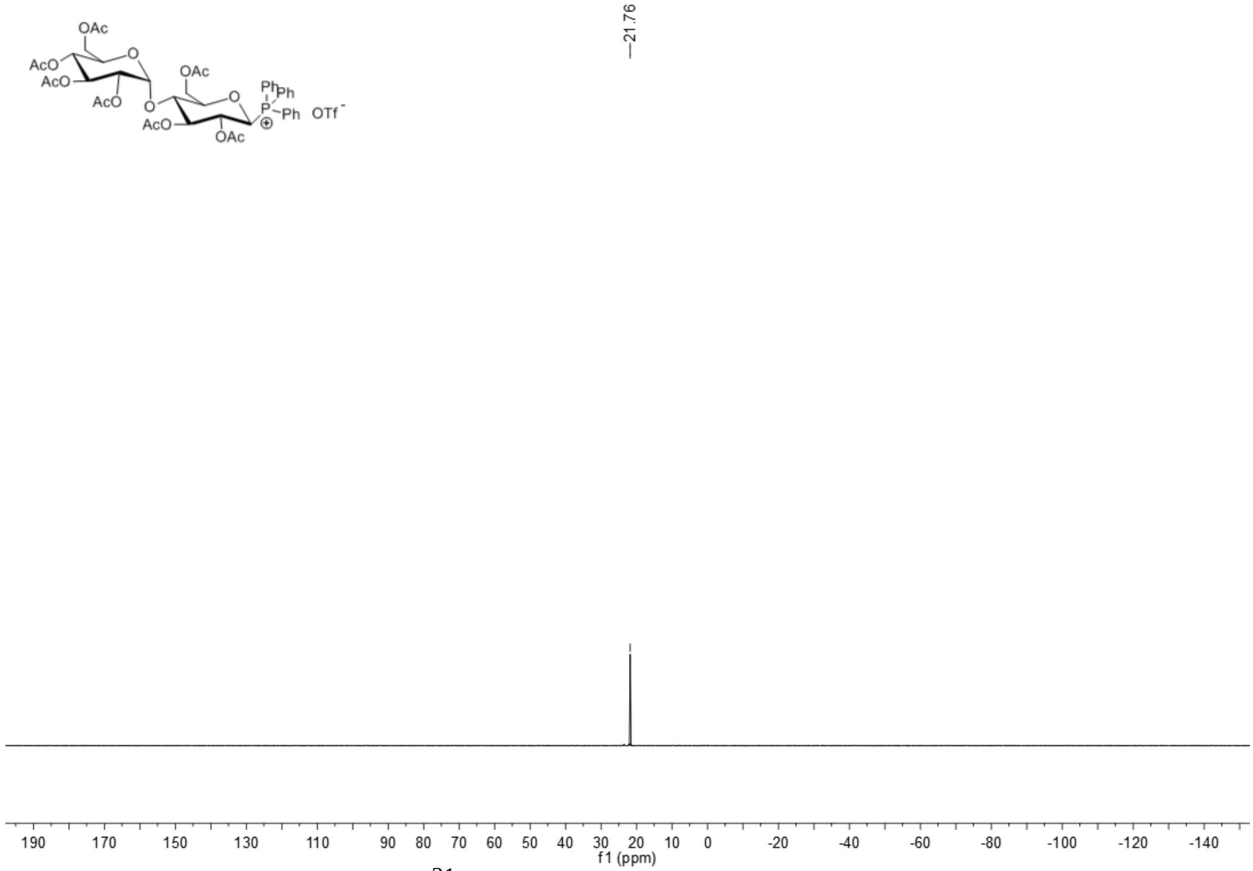

^{31}P NMR ($CDCl_3$-*d*) of **2u**

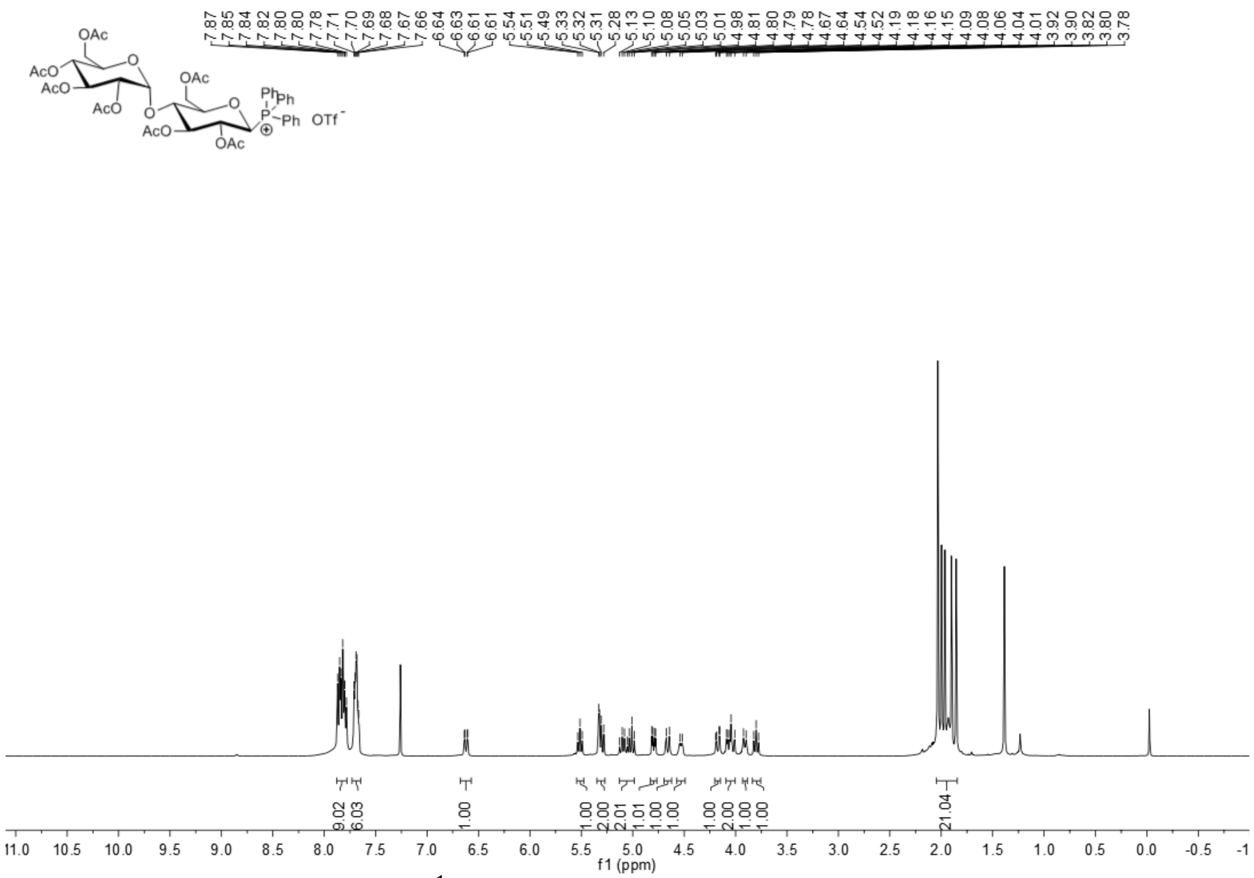

^{1}H NMR ($CDCl_3$-*d*) of **2u**

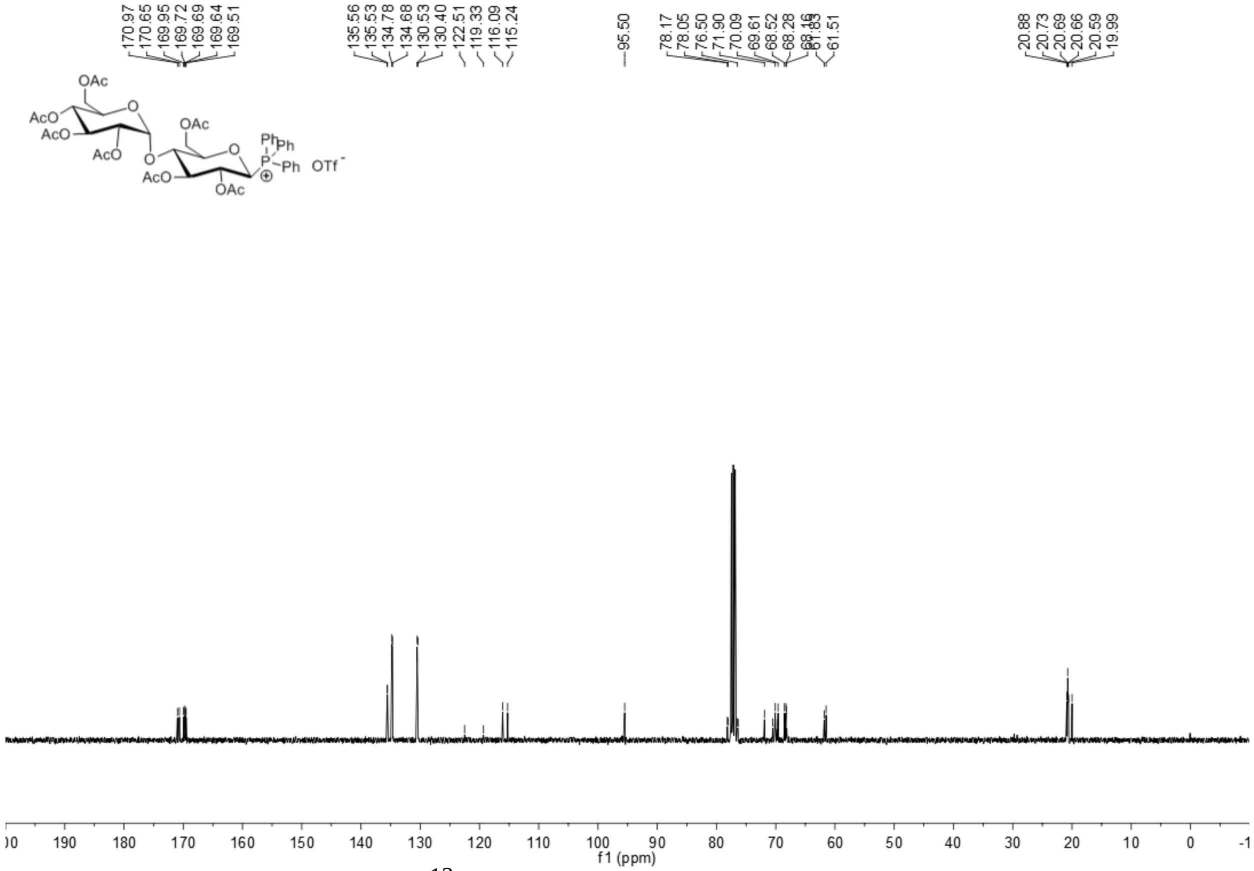

^{13}C NMR ($CDCl_3$-*d*) of **2u**

13. Copies of ^{1}H NMR, ^{13}C NMR Spectra of Compounds 3.

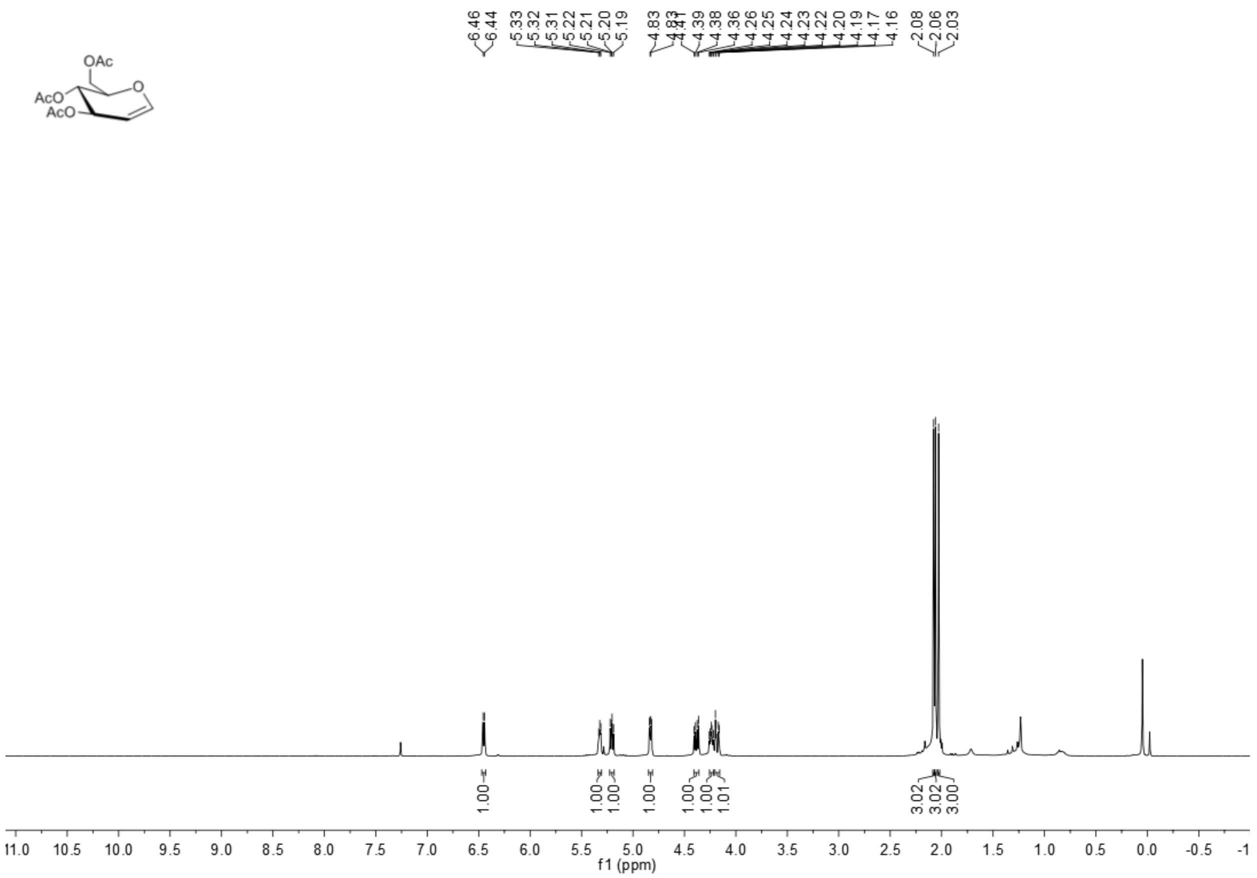

^{1}H NMR ($CDCl_3$-*d*) of **3a**[a][1]

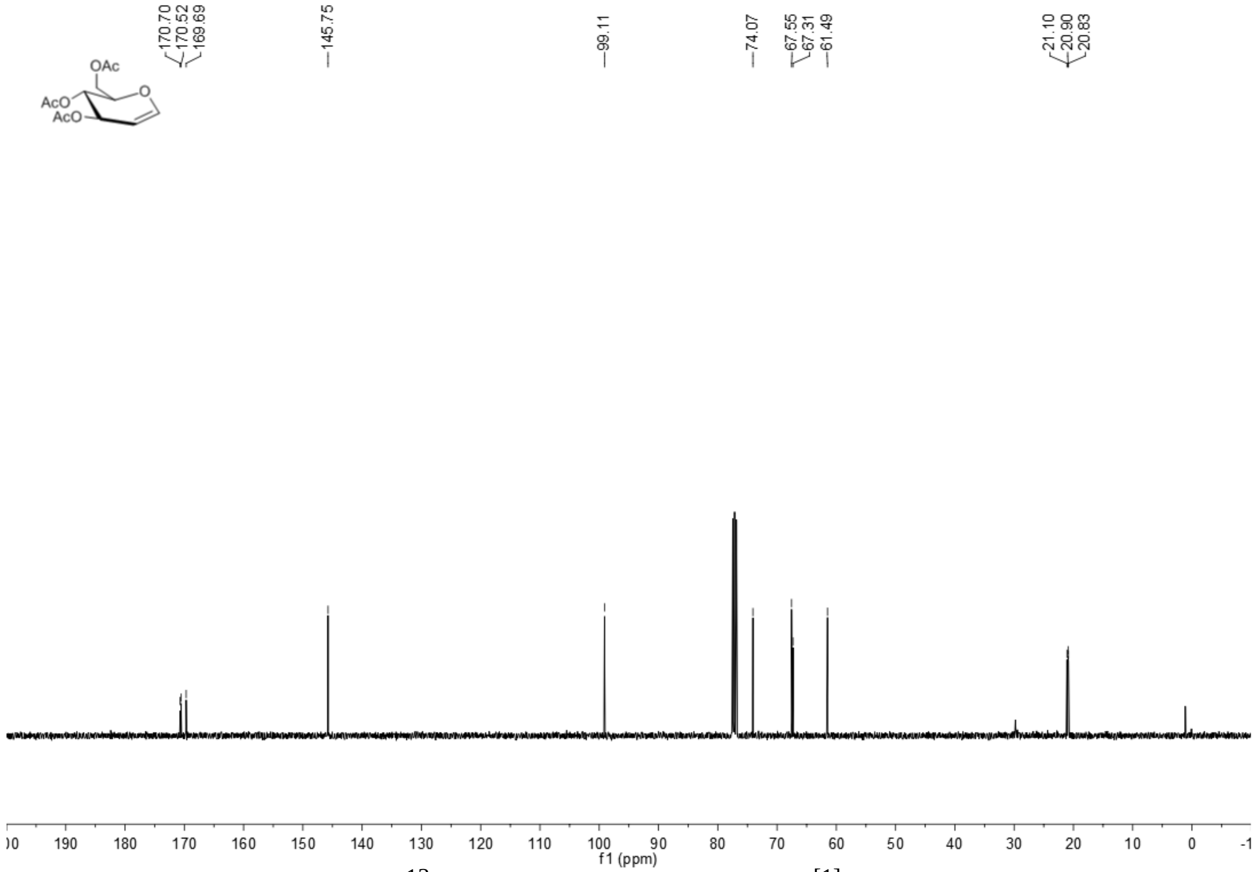

^{13}C NMR ($CDCl_3$-*d*) of **3a**[a][1]

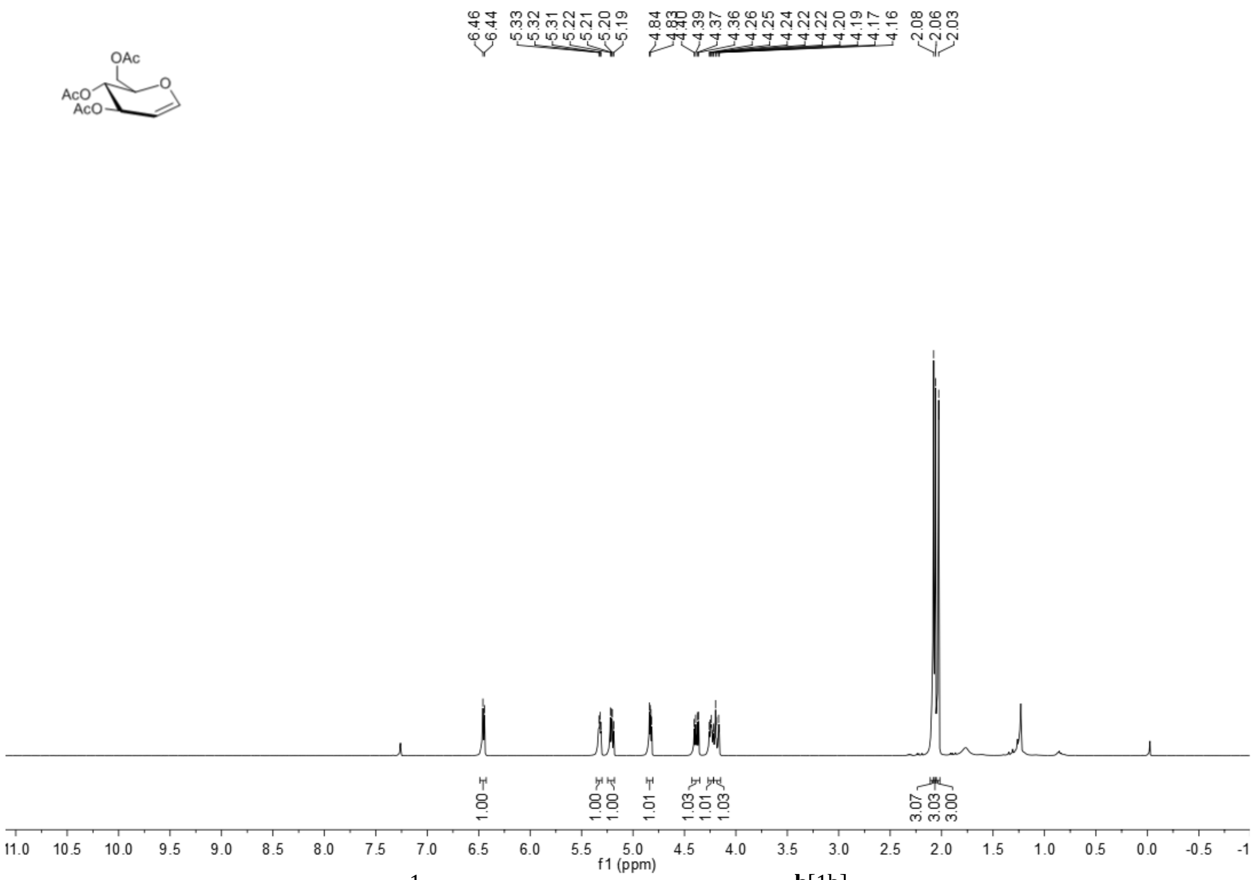

^{1}H NMR ($CDCl_3$-*d*) of **3a**[b][1b]

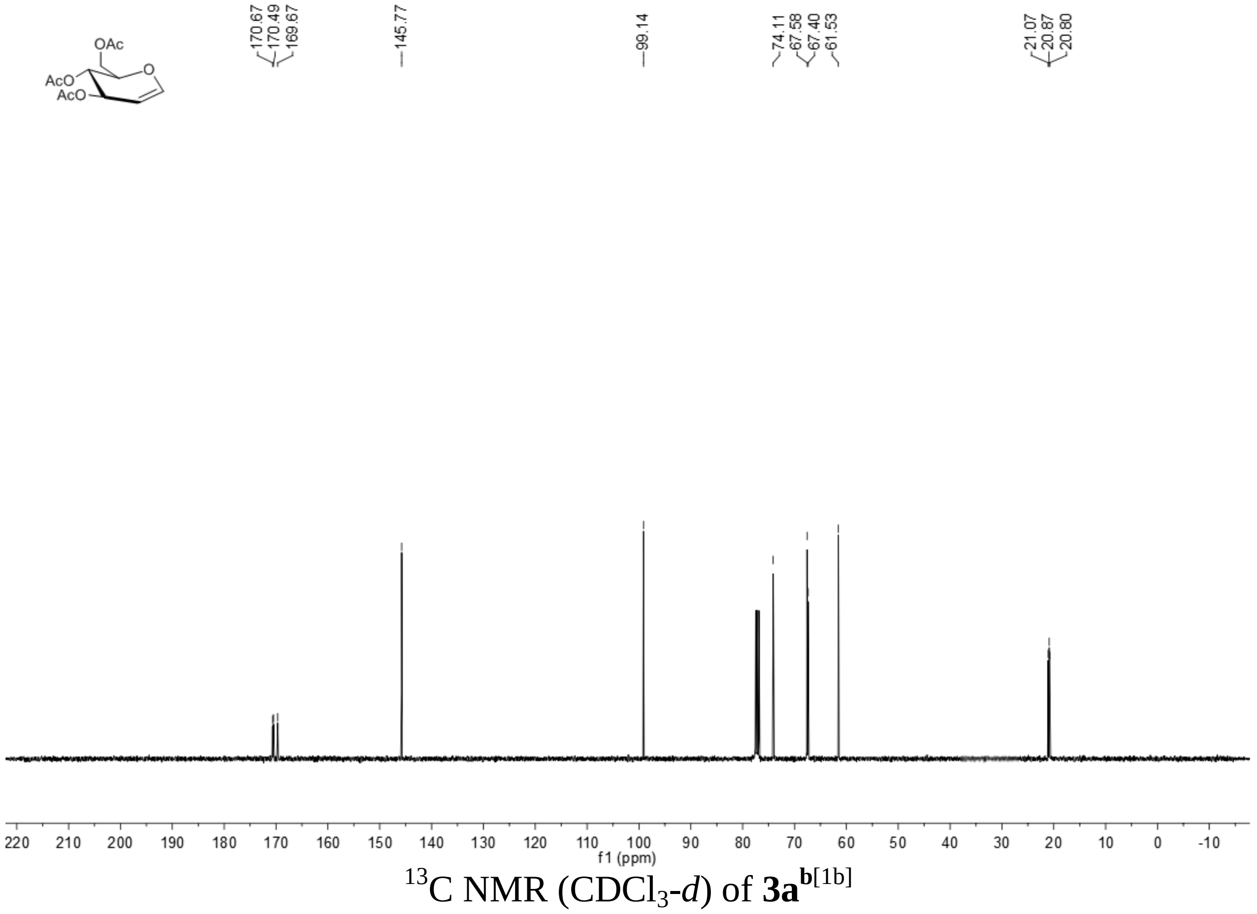

^{13}C NMR ($CDCl_3$-*d*) of **3a**[b][1b]

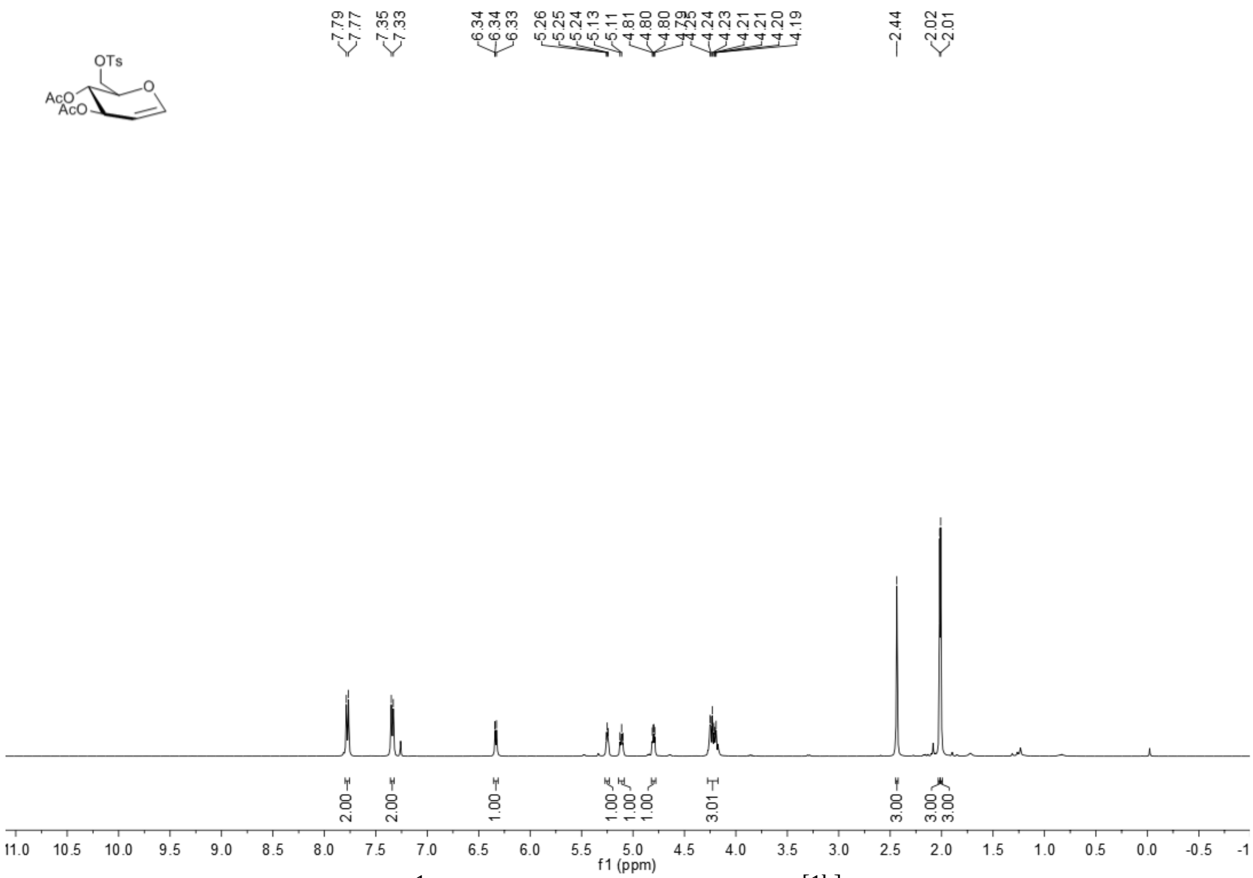

^{1}H NMR ($CDCl_3$-*d*) of **3b**[1b]

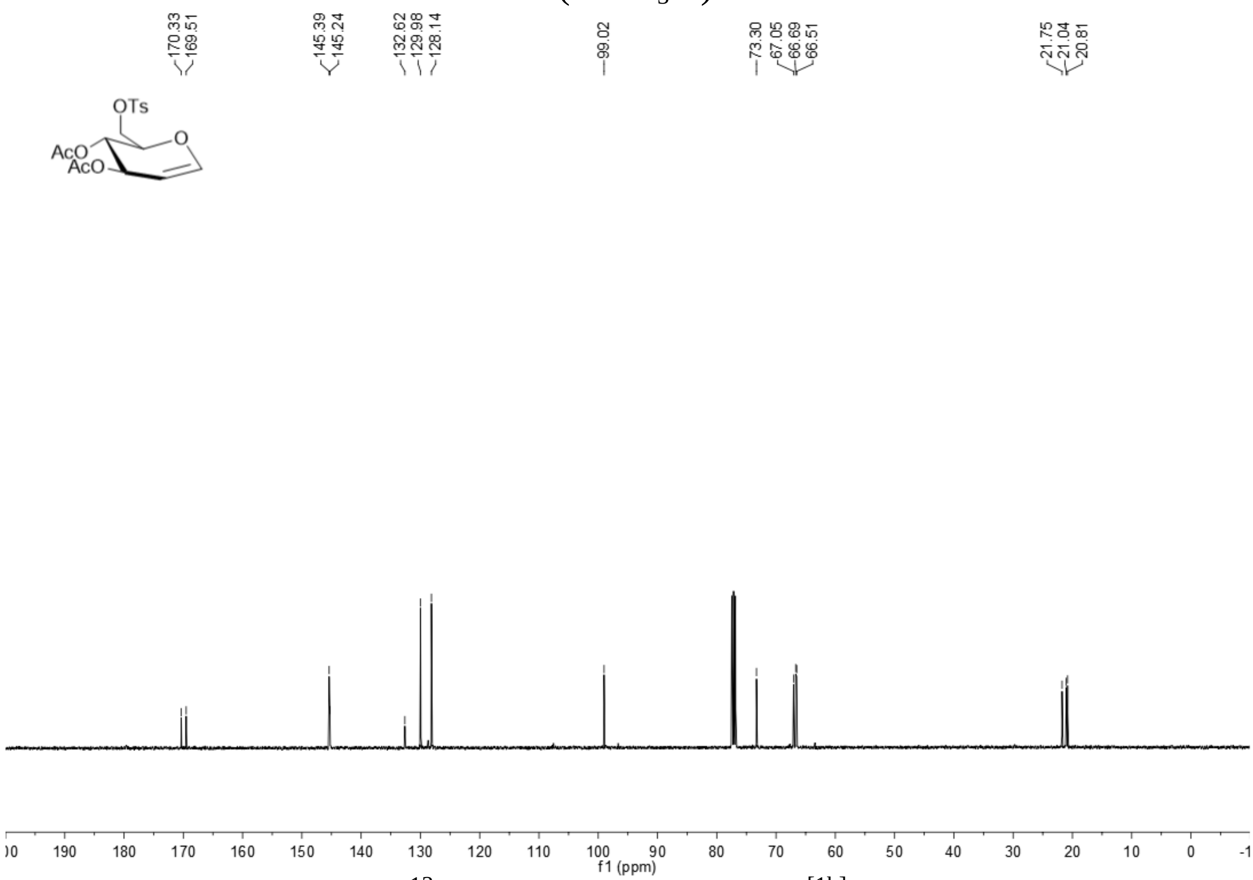

^{13}C NMR ($CDCl_3$-*d*) of **3b**[1b]

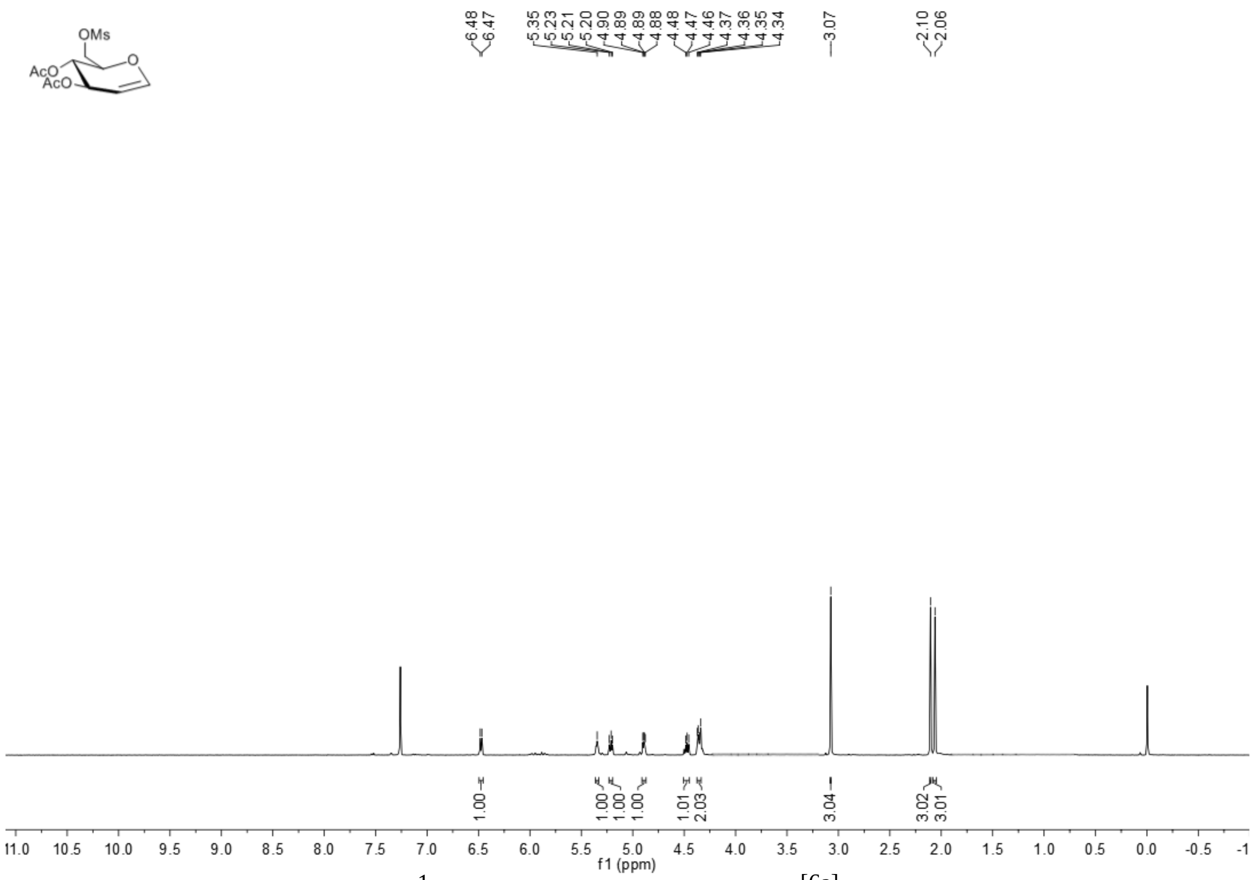

^{1}H NMR ($CDCl_3$-*d*) of **3c**[6a]

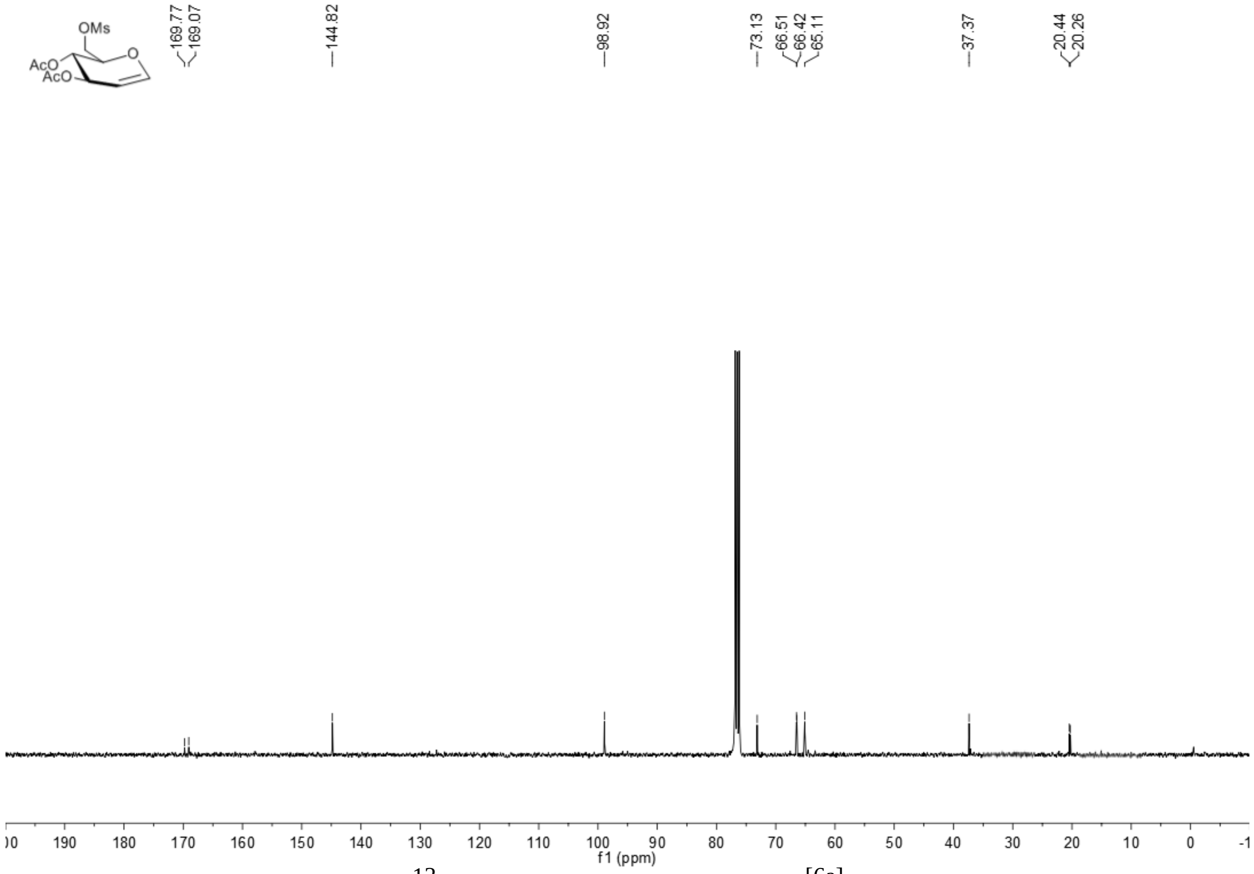

^{13}C NMR ($CDCl_3$-*d*) of **3c**[6a]

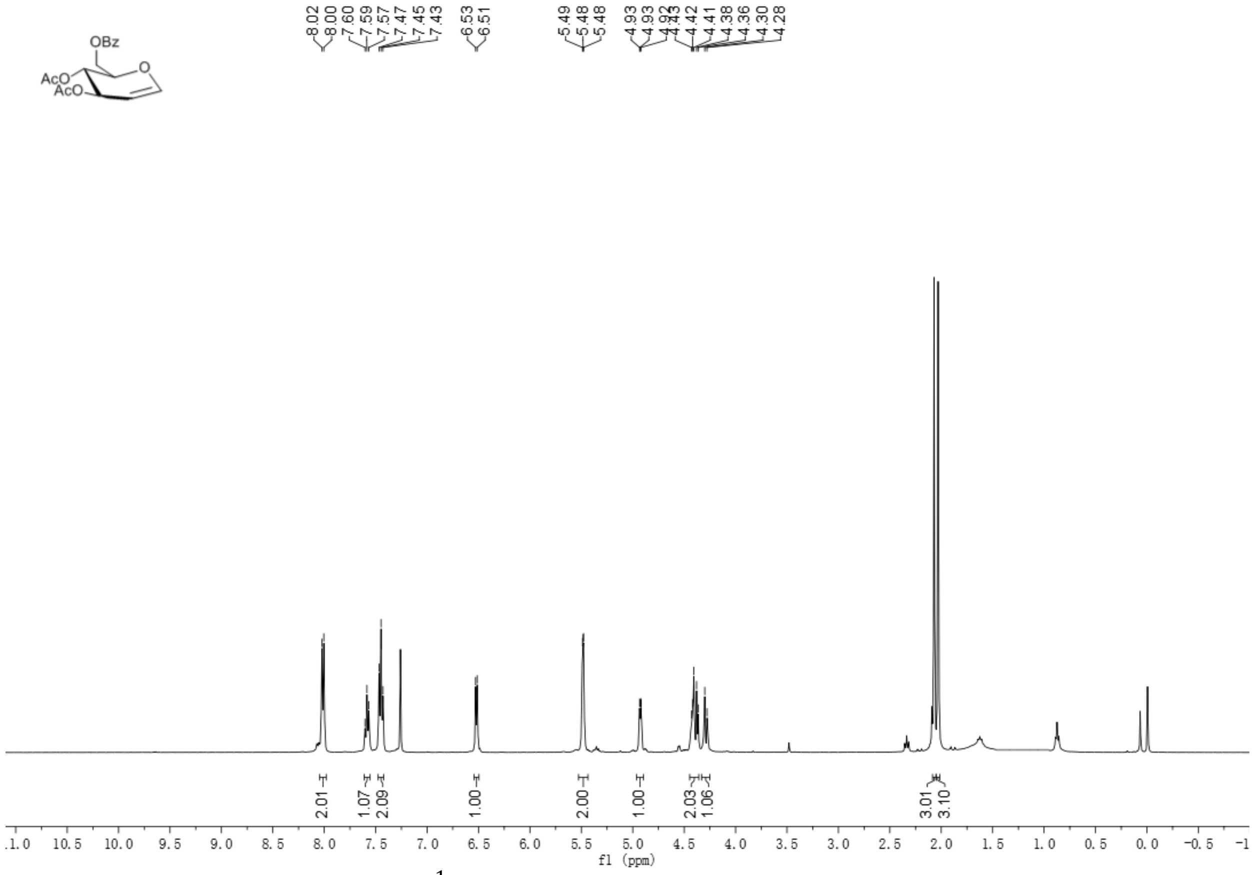

^{1}H NMR ($CDCl_3$-*d*) of **3d**

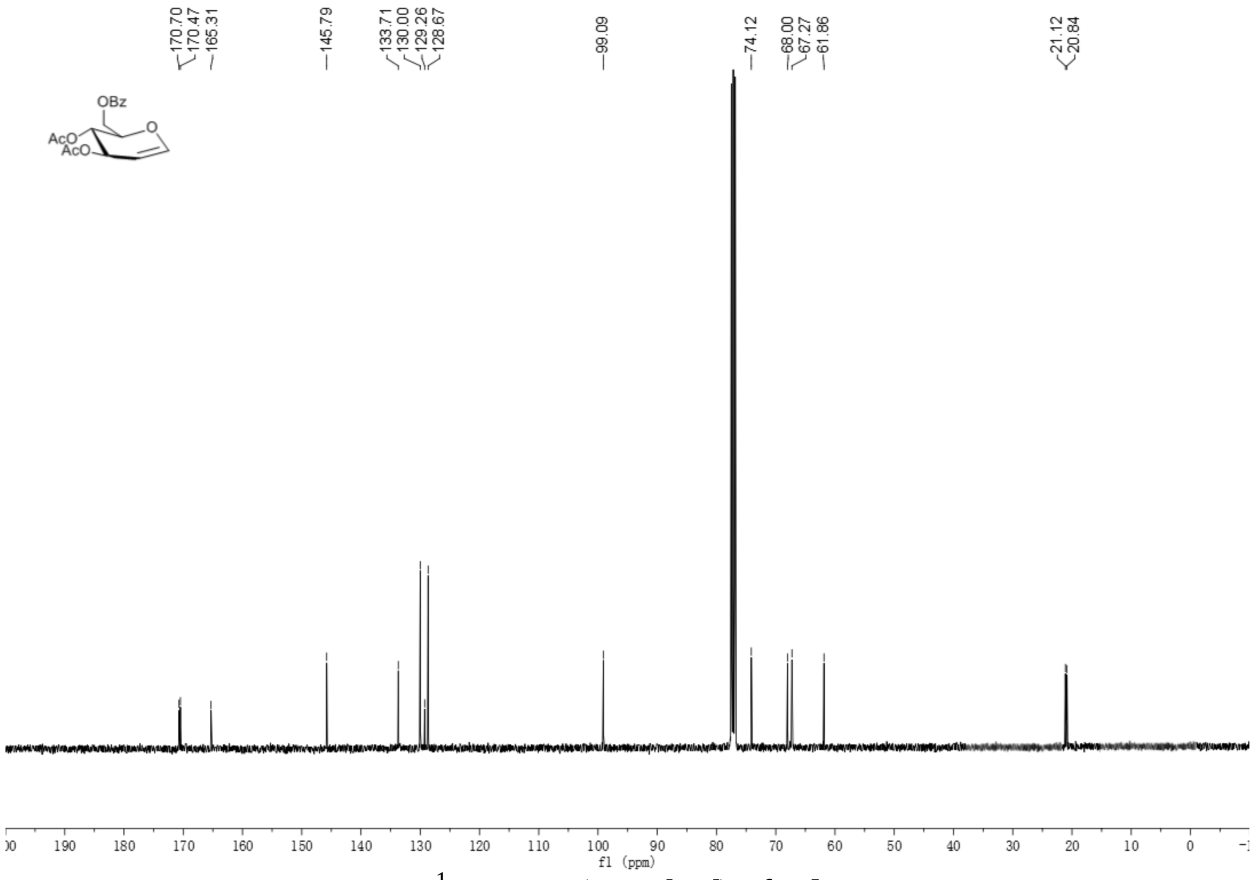

^{1}C NMR ($CDCl_3$-*d*) of **3d**

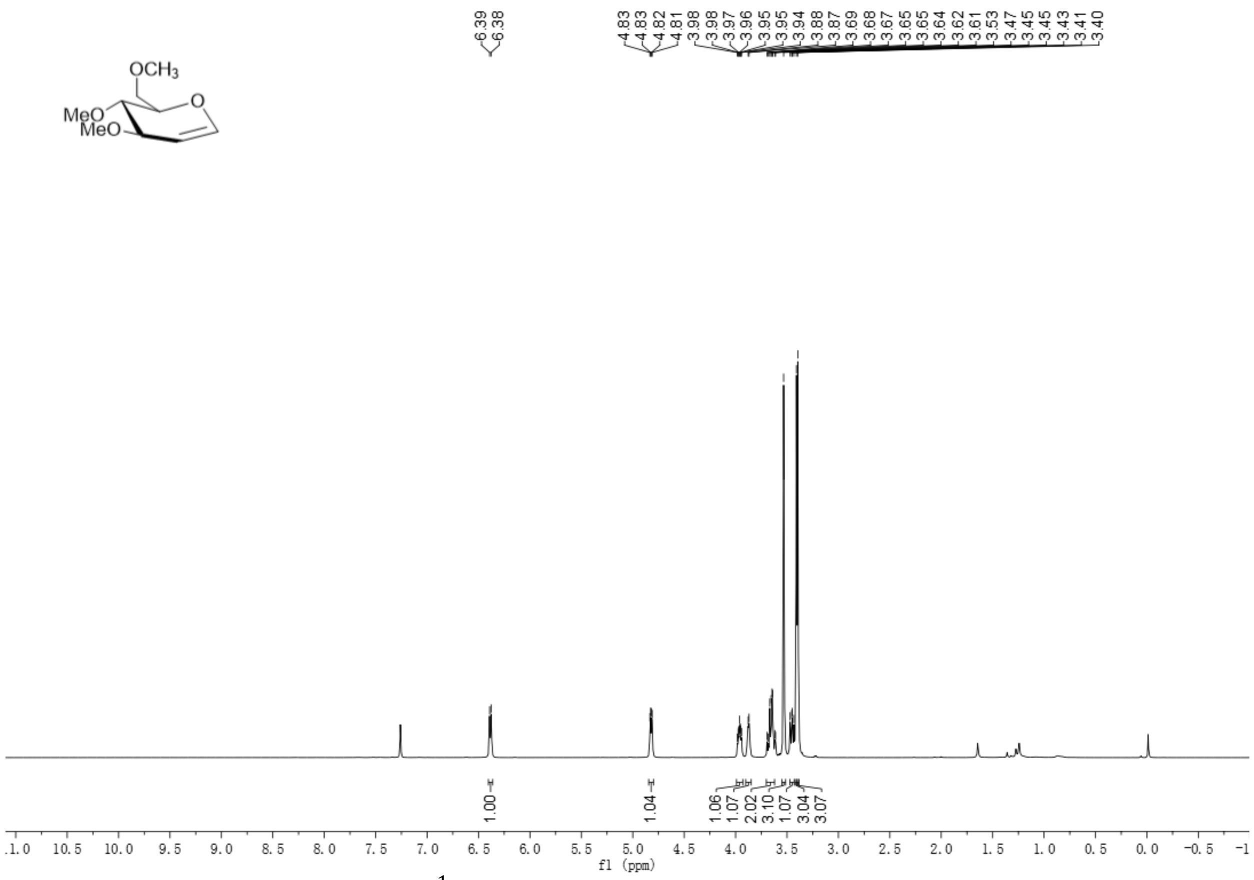

^{1}H NMR ($CDCl_3$-*d*) of **3e**

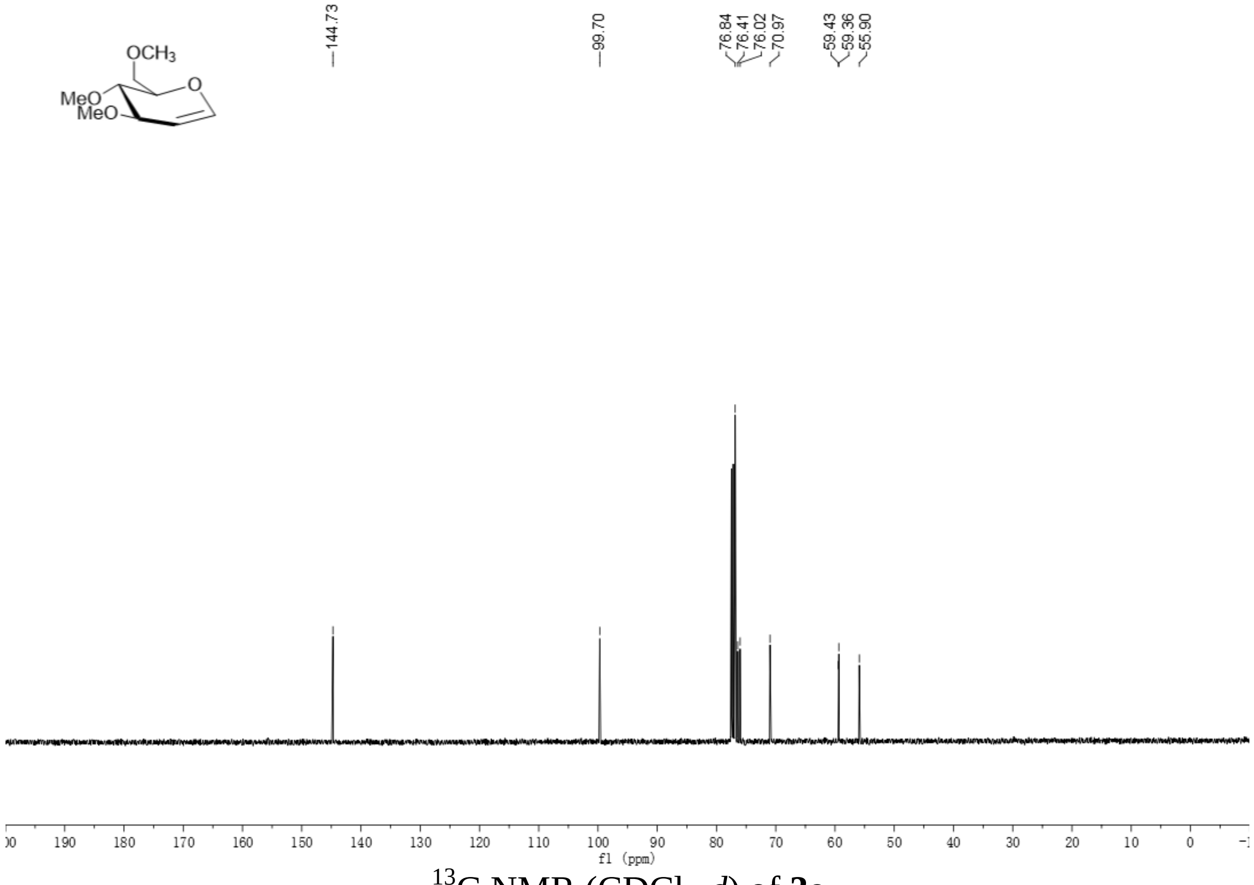

^{13}C NMR ($CDCl_3$-*d*) of **3e**

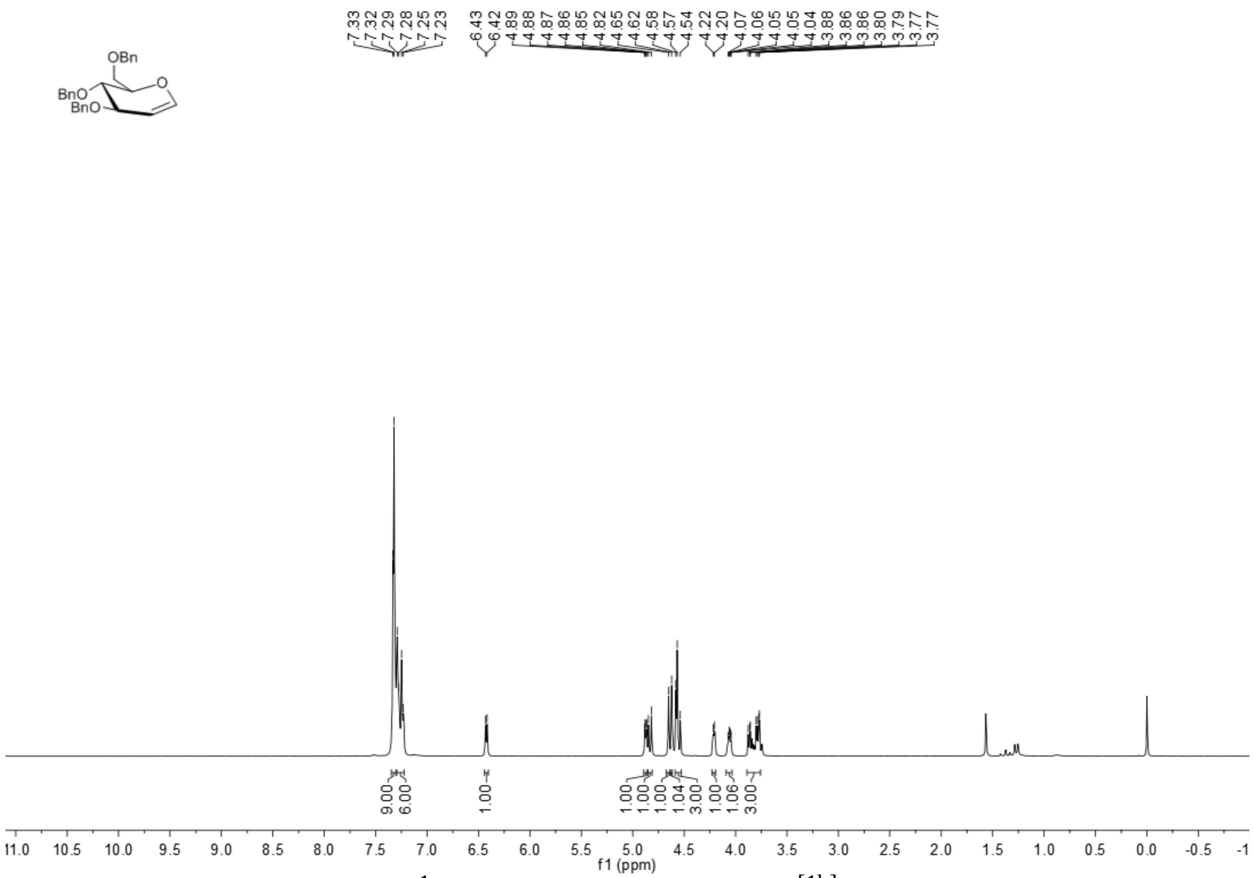

^{1}H NMR ($CDCl_3$-*d*) of **3f**[1b]

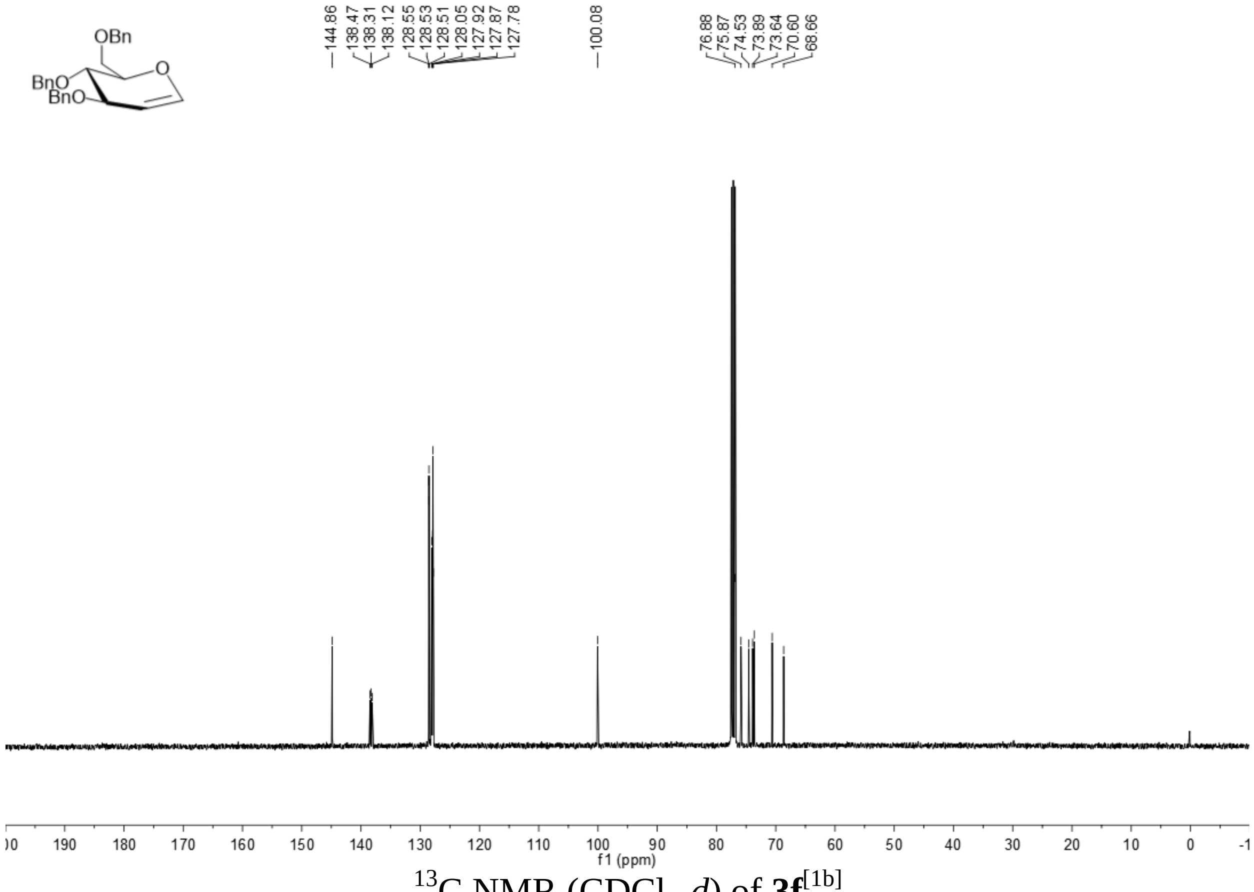

^{13}C NMR ($CDCl_3$-*d*) of **3f**[1b]

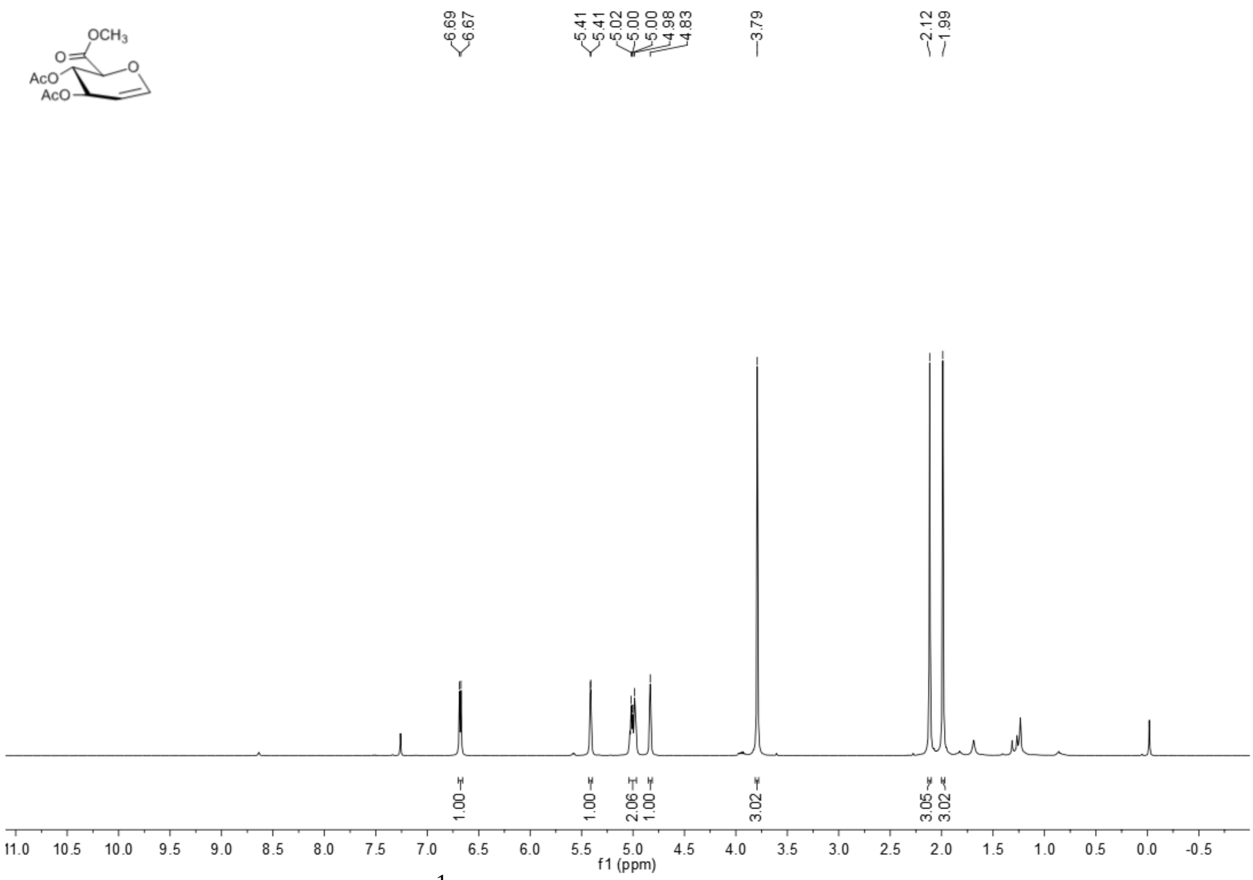

^{1}H NMR ($CDCl_3$-*d*) of **3g**

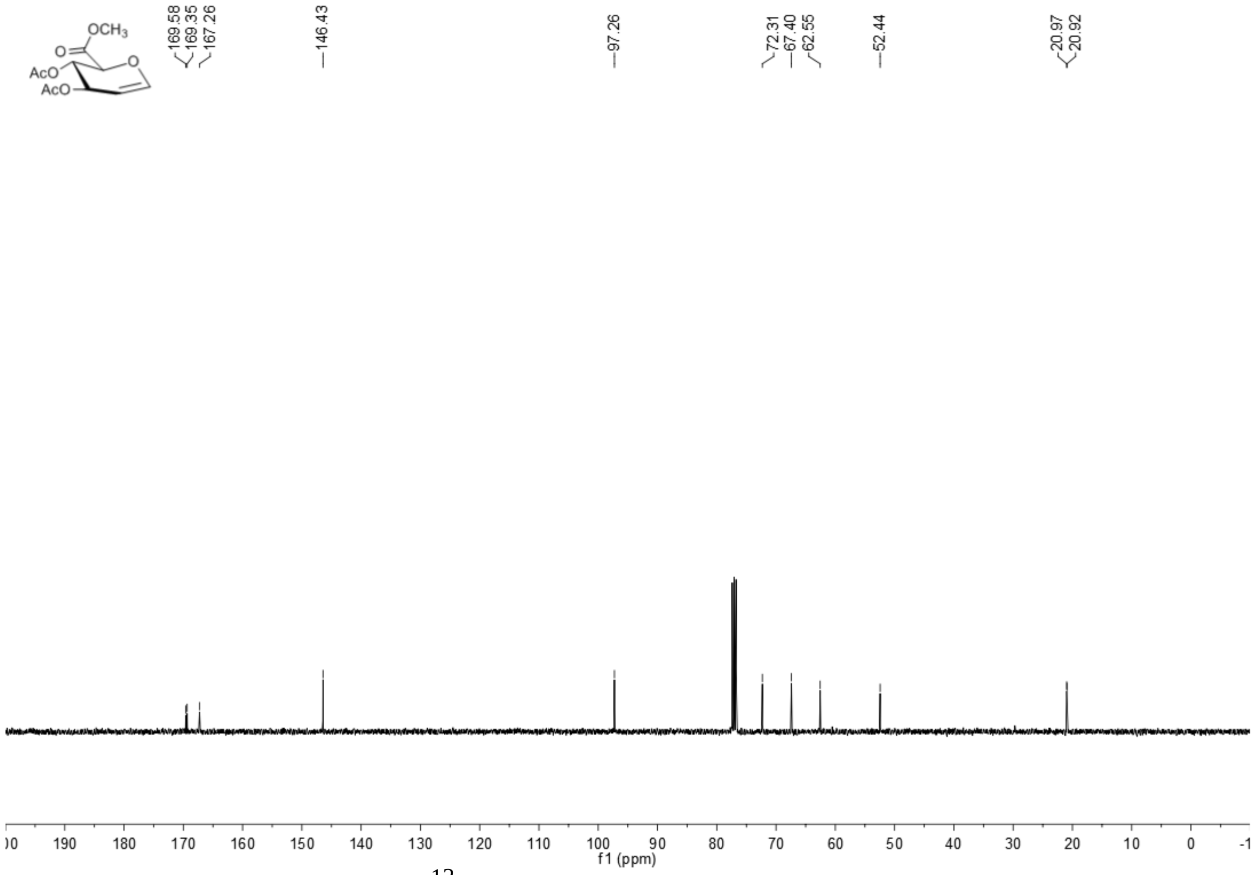

^{13}C NMR ($CDCl_3$-*d*) of **3g**

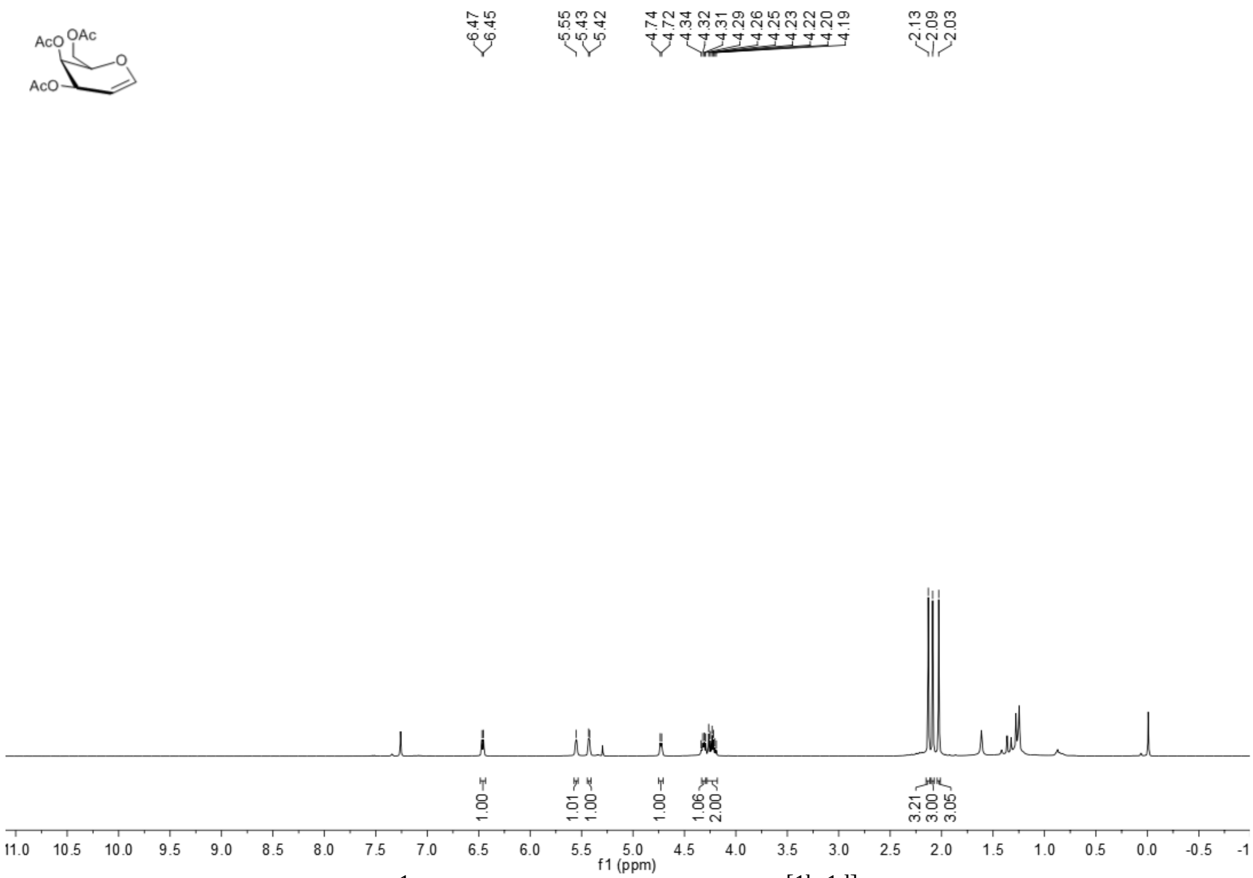

^{1}H NMR ($CDCl_3$-*d*) of **3h**[1b-1d]

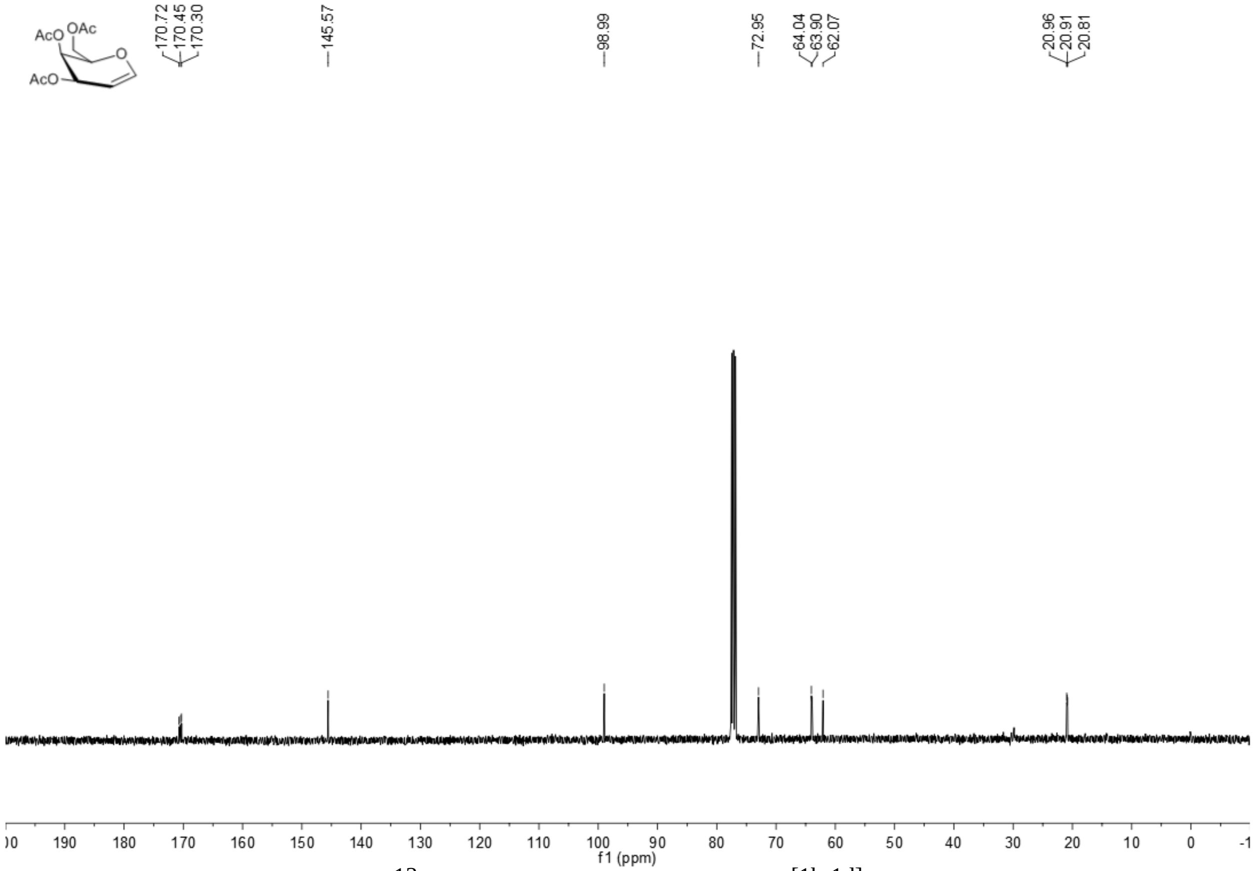

^{13}C NMR ($CDCl_3$-*d*) of **3h**[1b-1d]

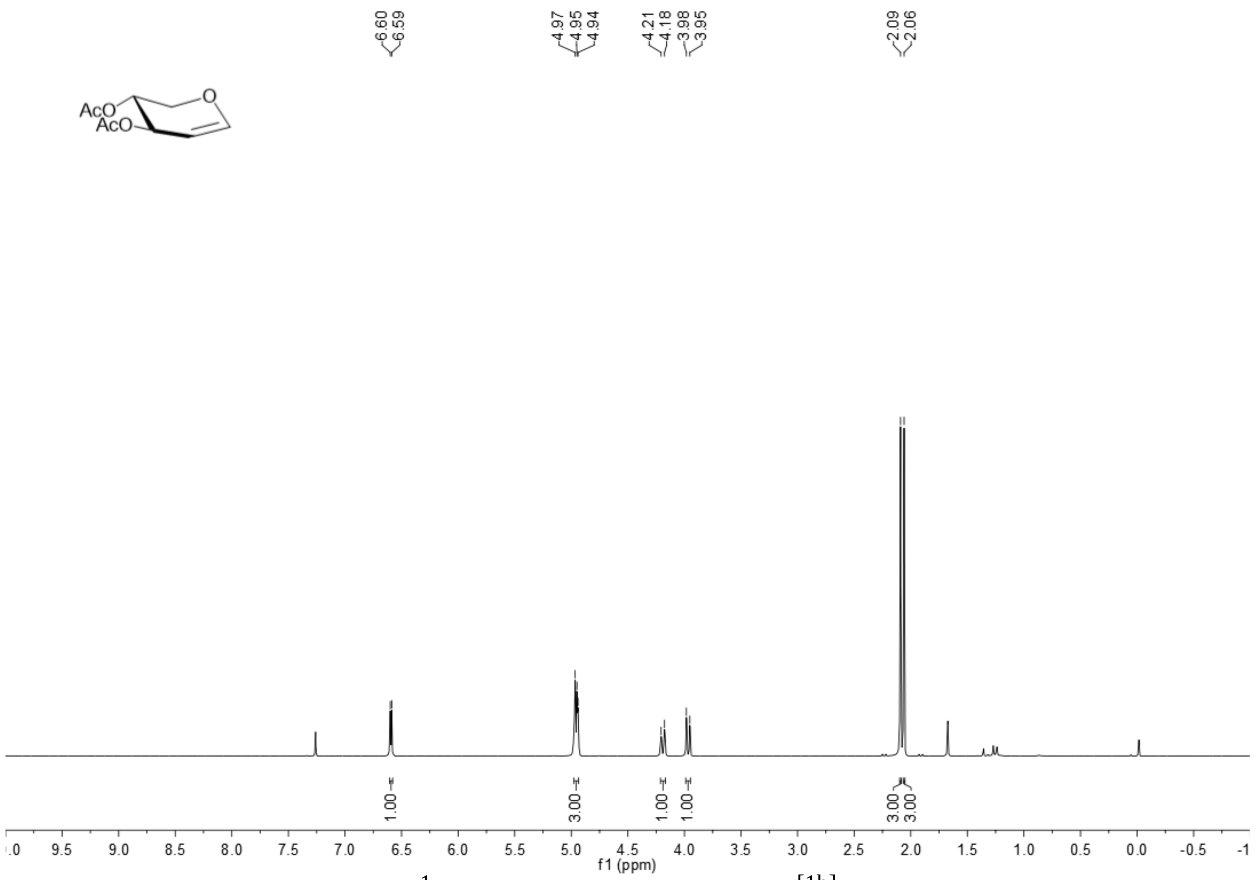

^{1}H NMR ($CDCl_3$-*d*) of **3i**[1b]

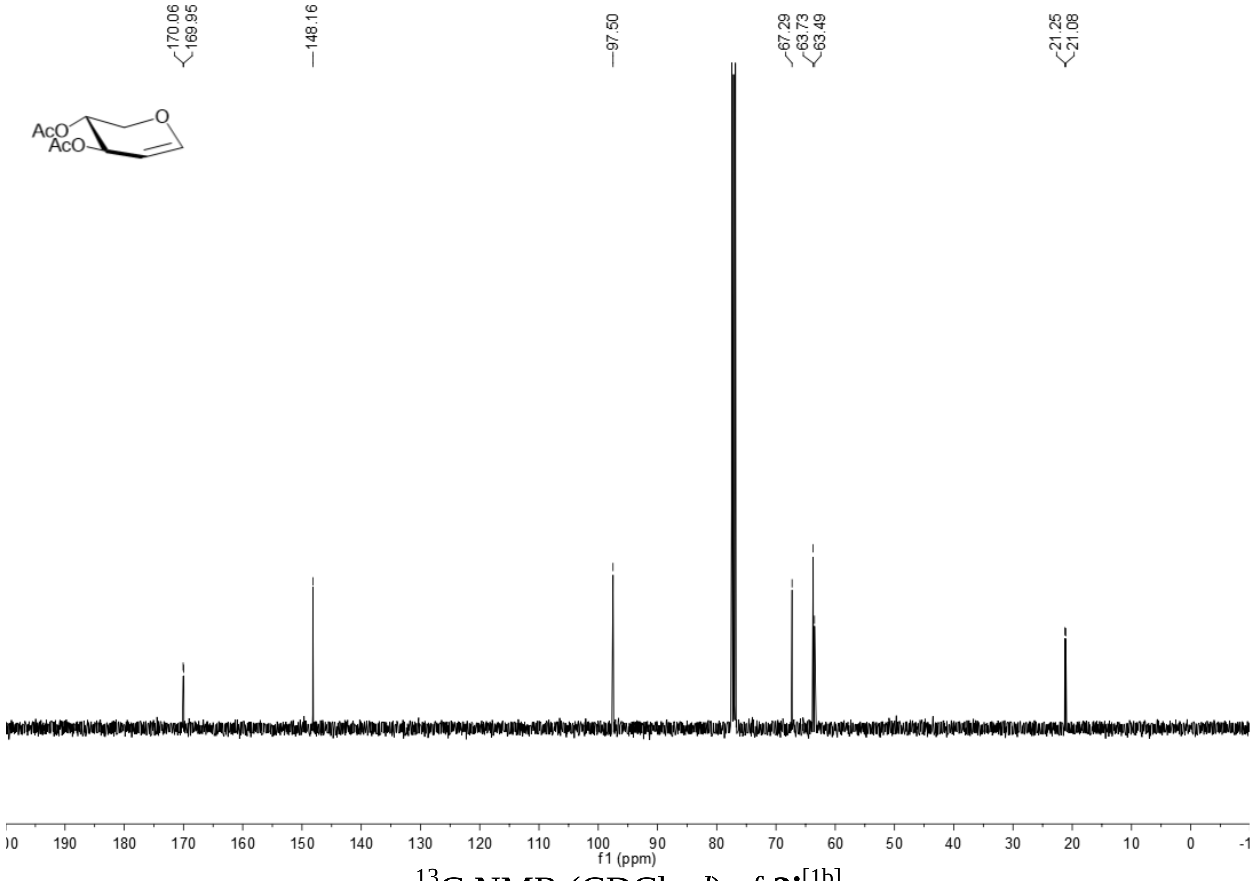

^{13}C NMR ($CDCl_3$-*d*) of **3i**[1b]

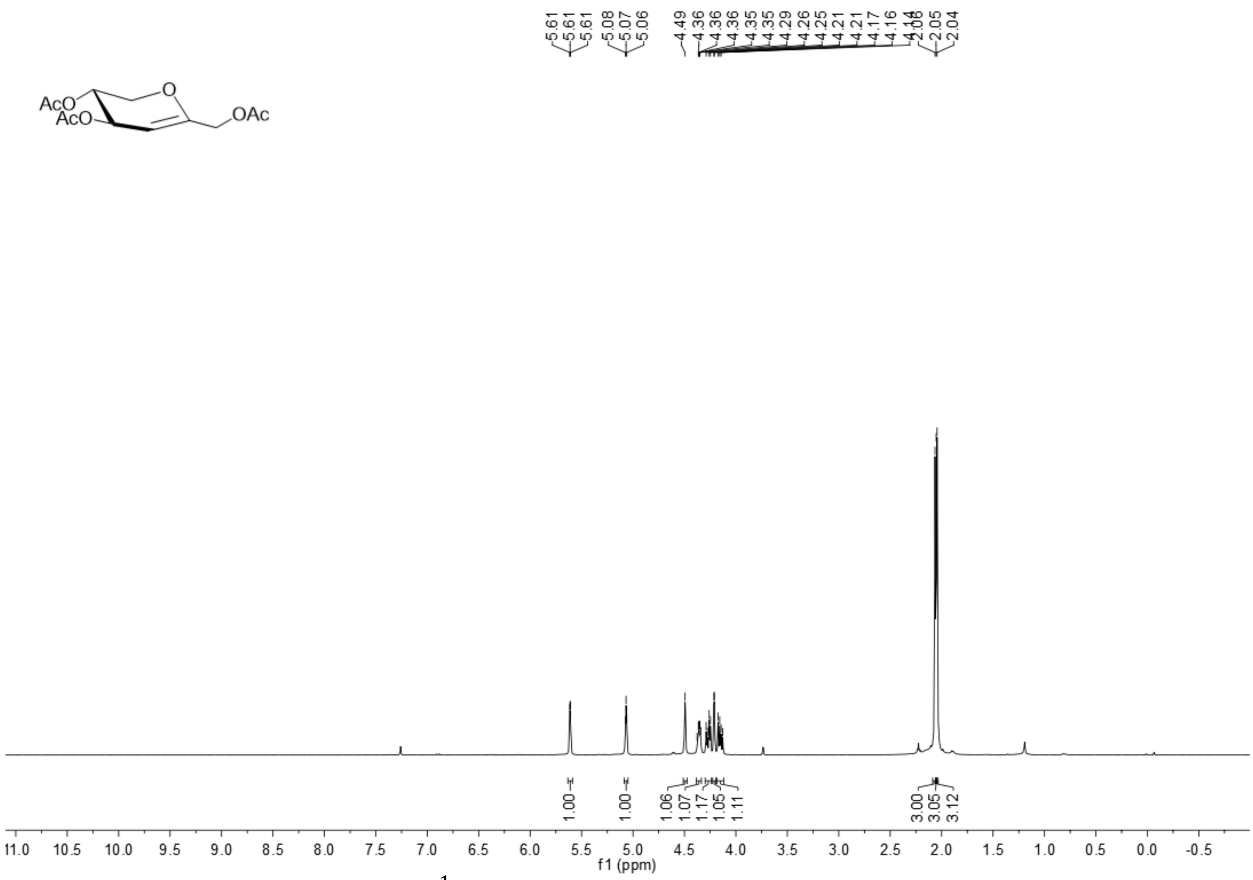

^{1}H NMR ($CDCl_3$-*d*) of **3j**

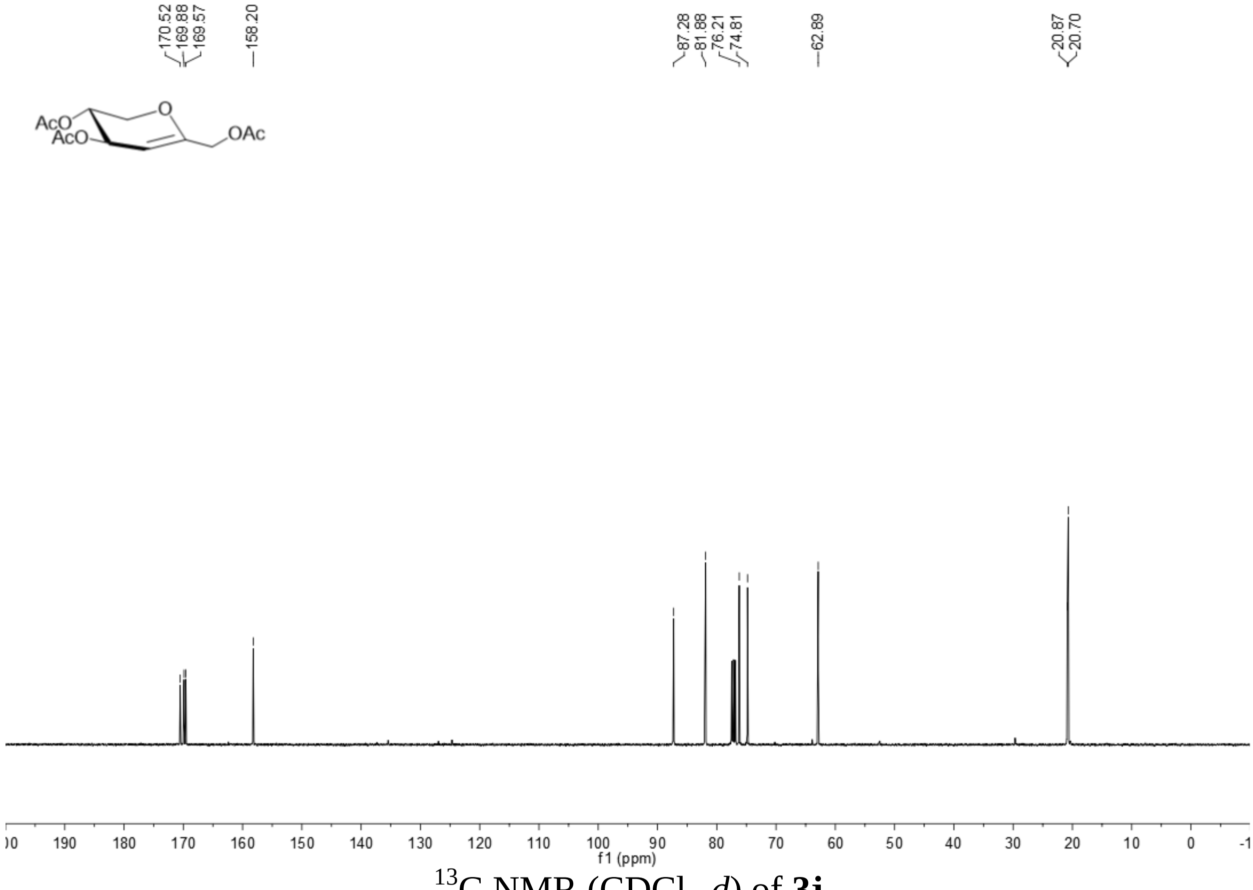

^{13}C NMR ($CDCl_3$-*d*) of **3j**

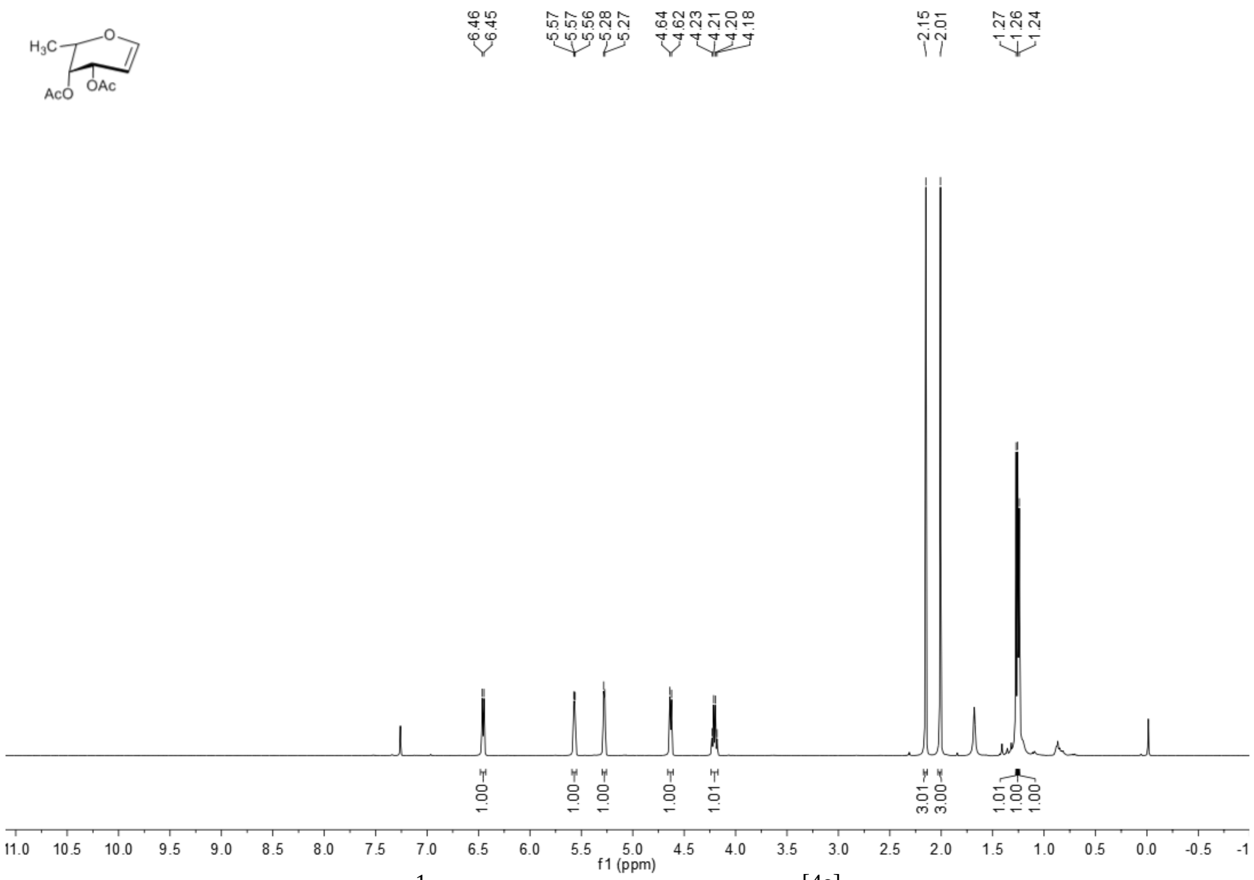

^{1}H NMR ($CDCl_3$-*d*) of **3k**[4e]

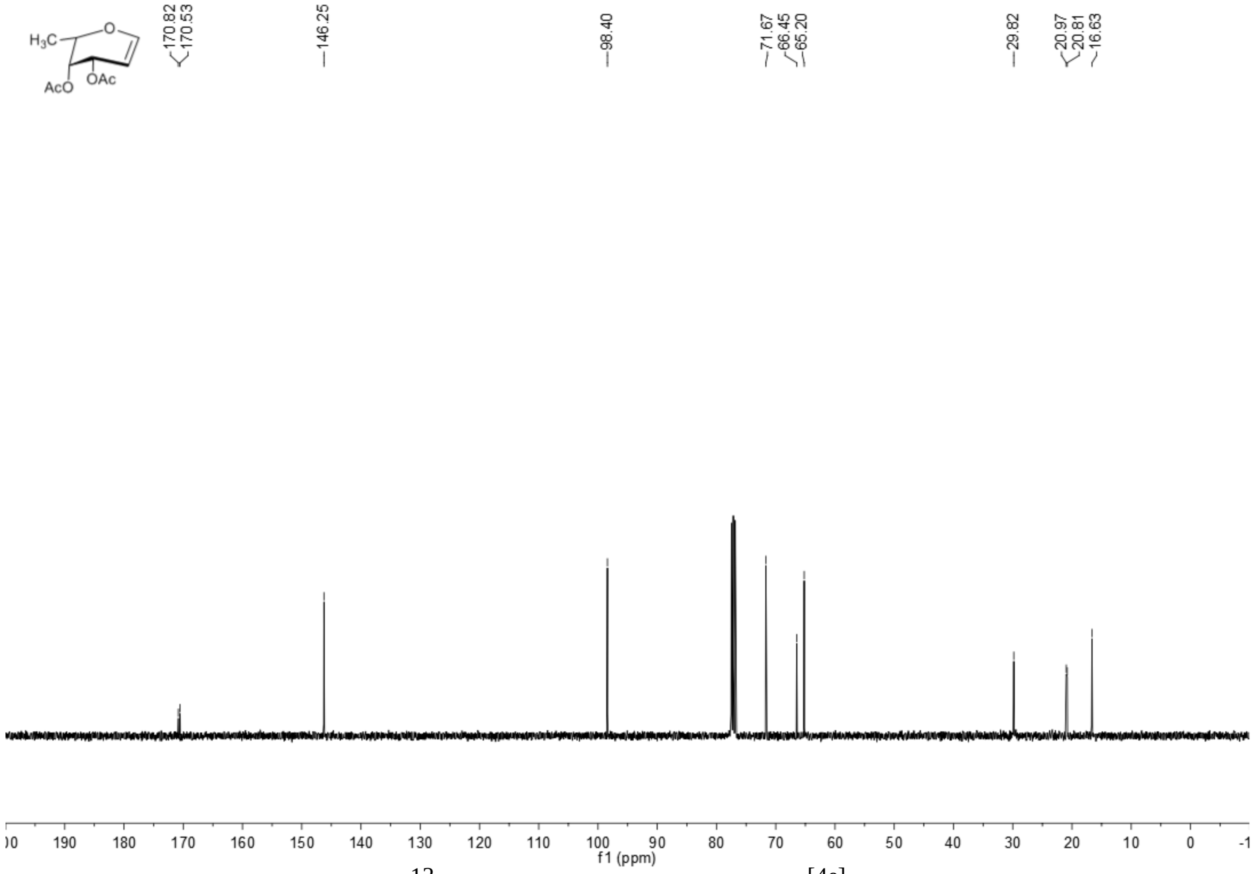

^{13}C NMR ($CDCl_3$-*d*) of **3k**[4e]

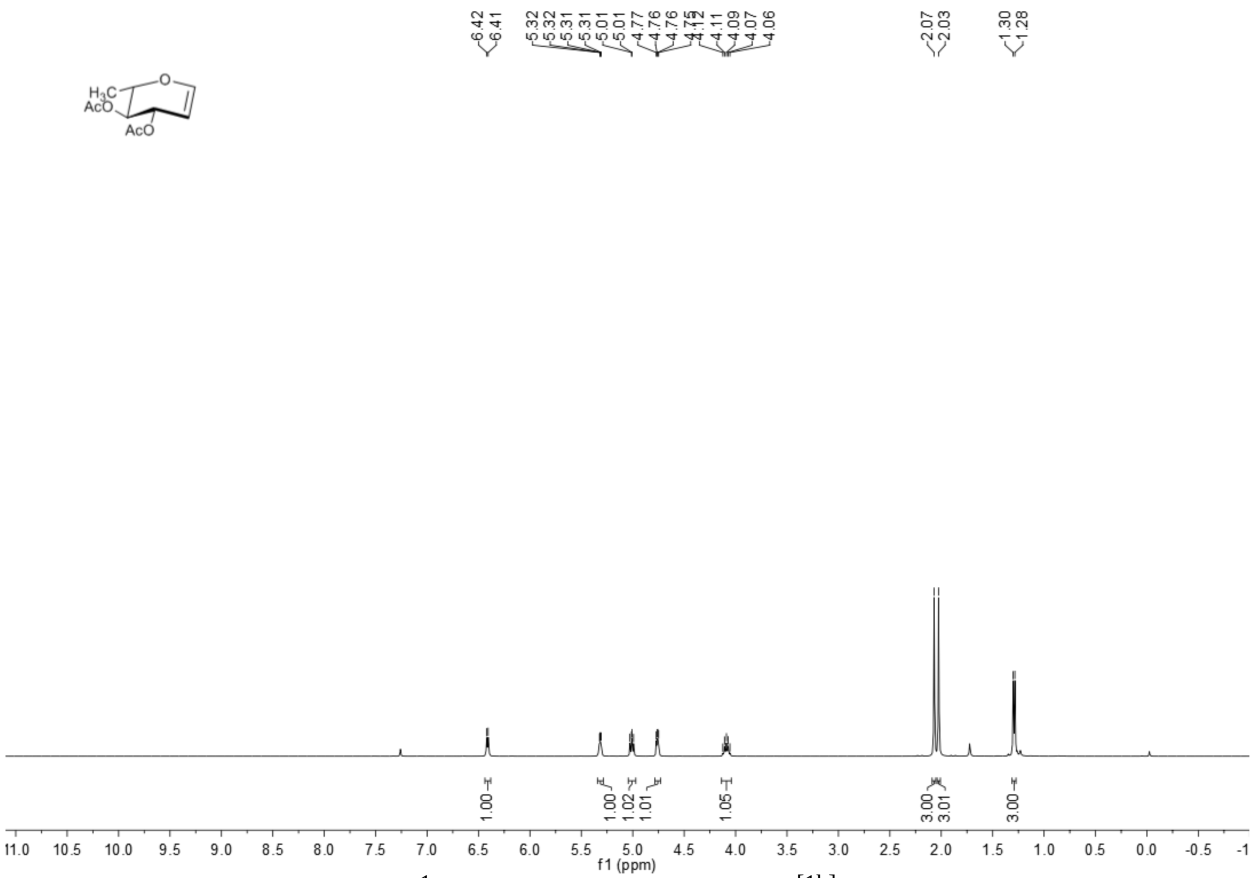

^{1}H NMR ($CDCl_3$-*d*) of **3l**[1b]

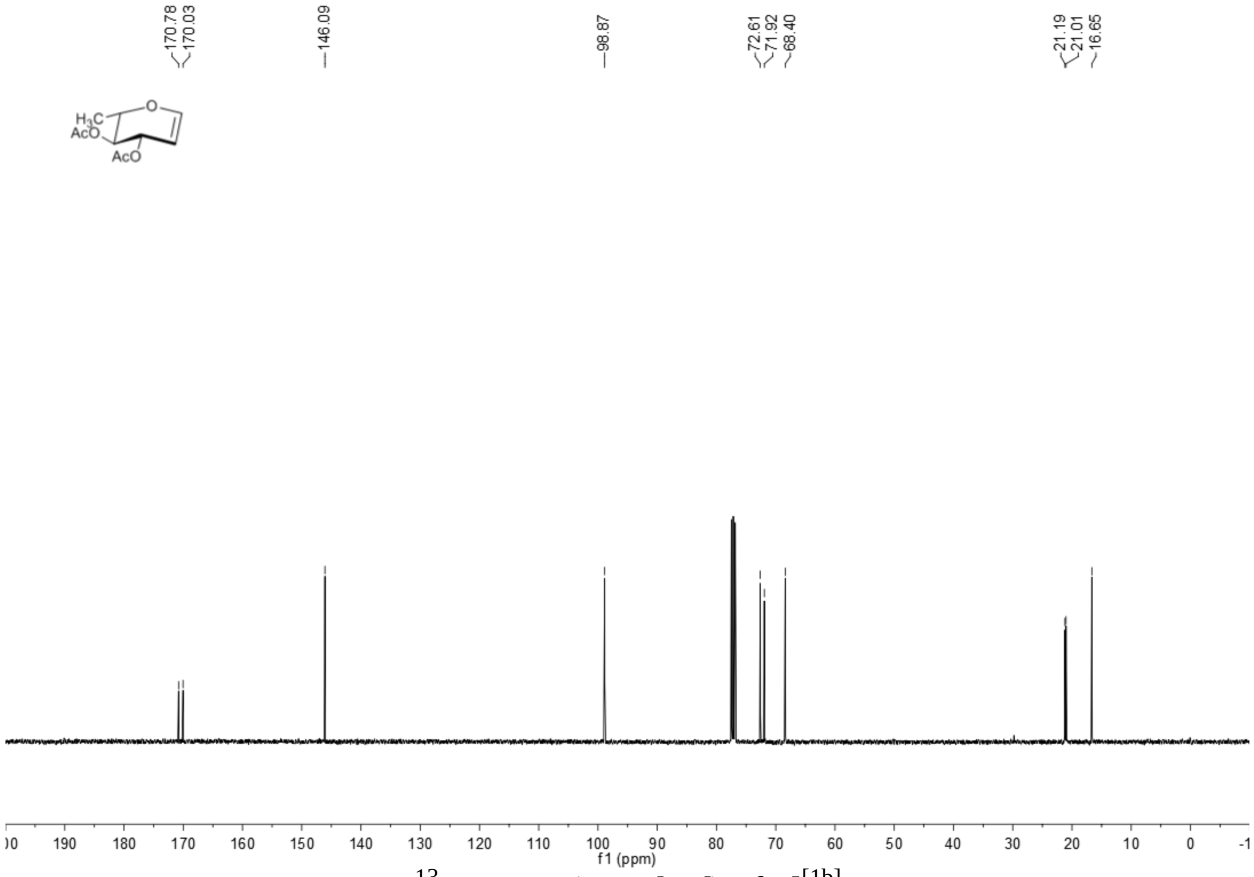

^{13}C NMR ($CDCl_3$-*d*) of **3l**[1b]

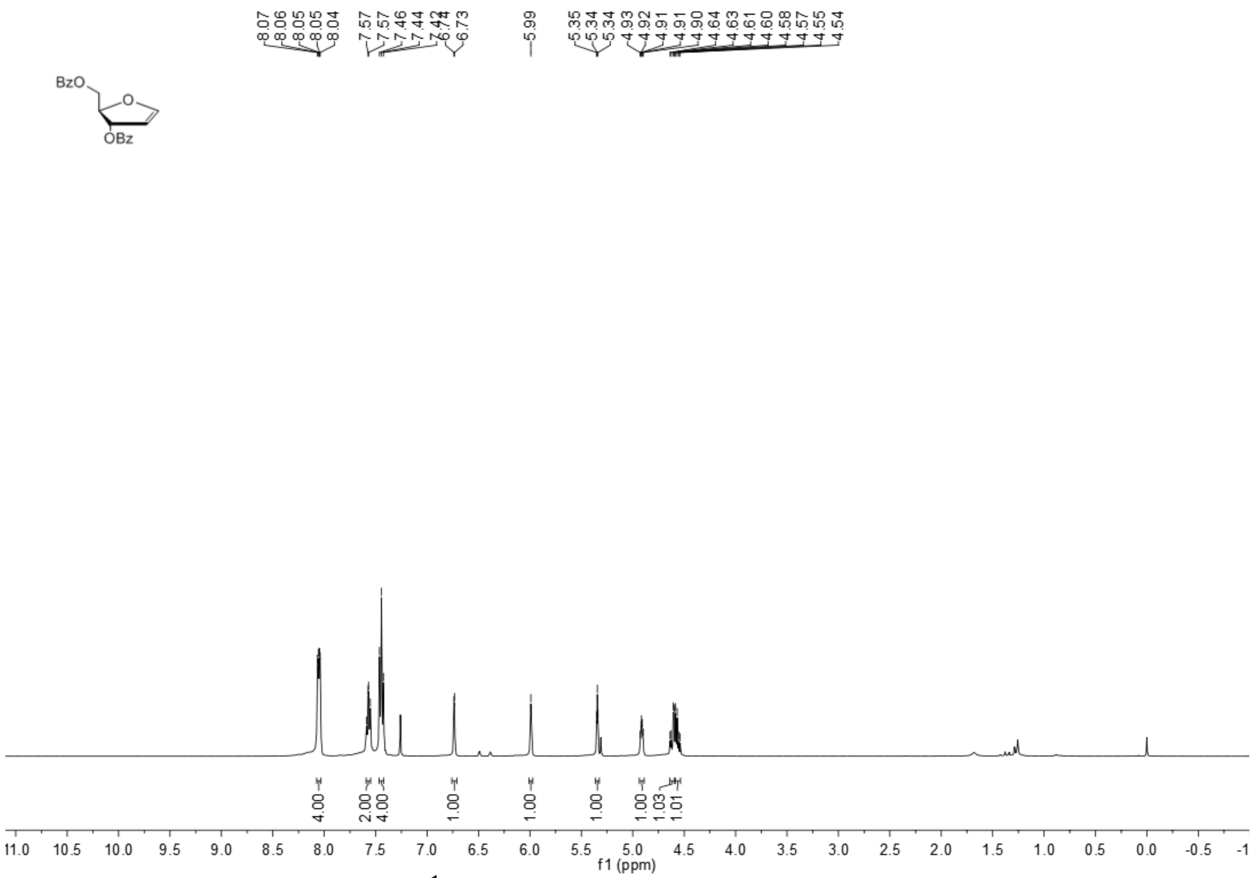

^{1}H NMR ($CDCl_3$-*d*) of **3m**

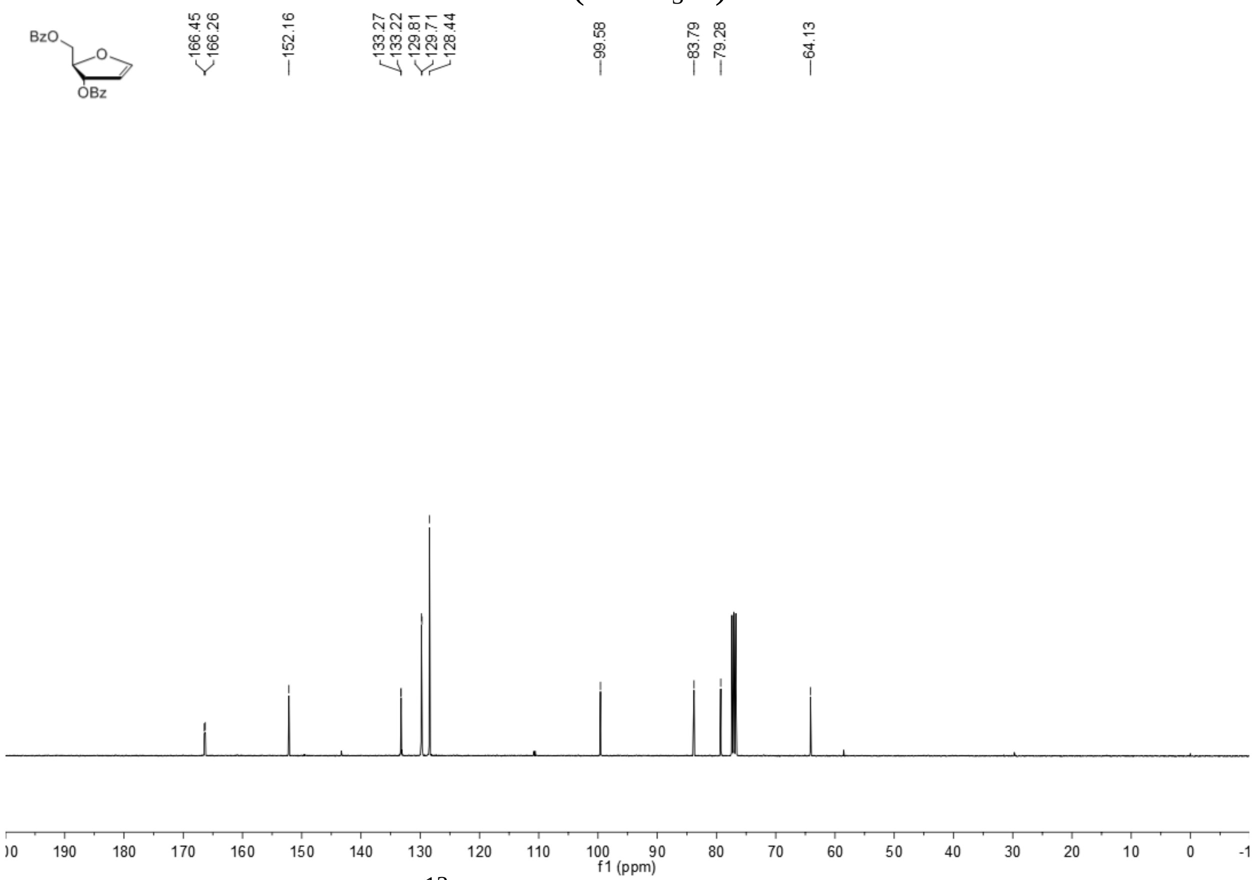

^{13}C NMR ($CDCl_3$-*d*) of **3m**

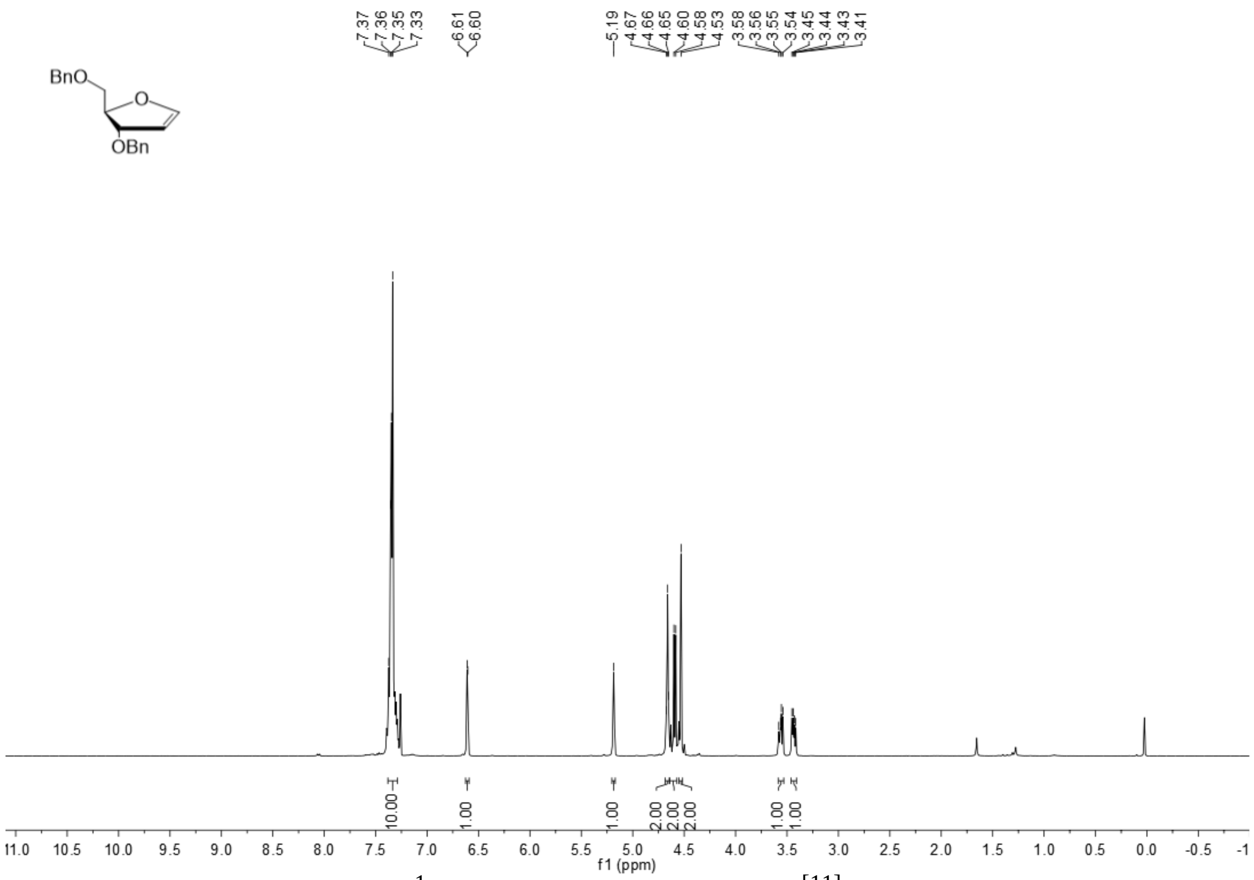

^{1}H NMR ($CDCl_3$-*d*) of **3n**[11]

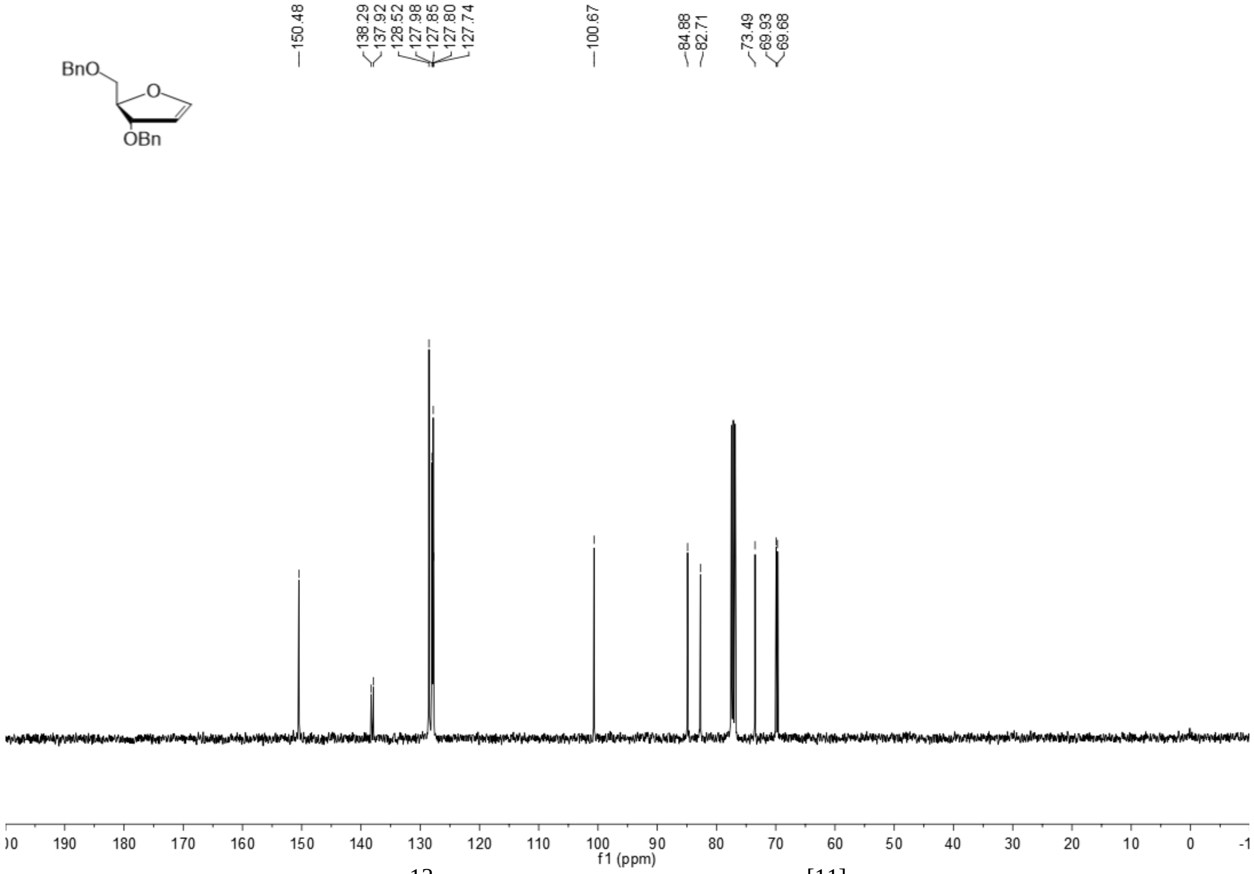

^{13}C NMR ($CDCl_3$-*d*) of **3n**[11]

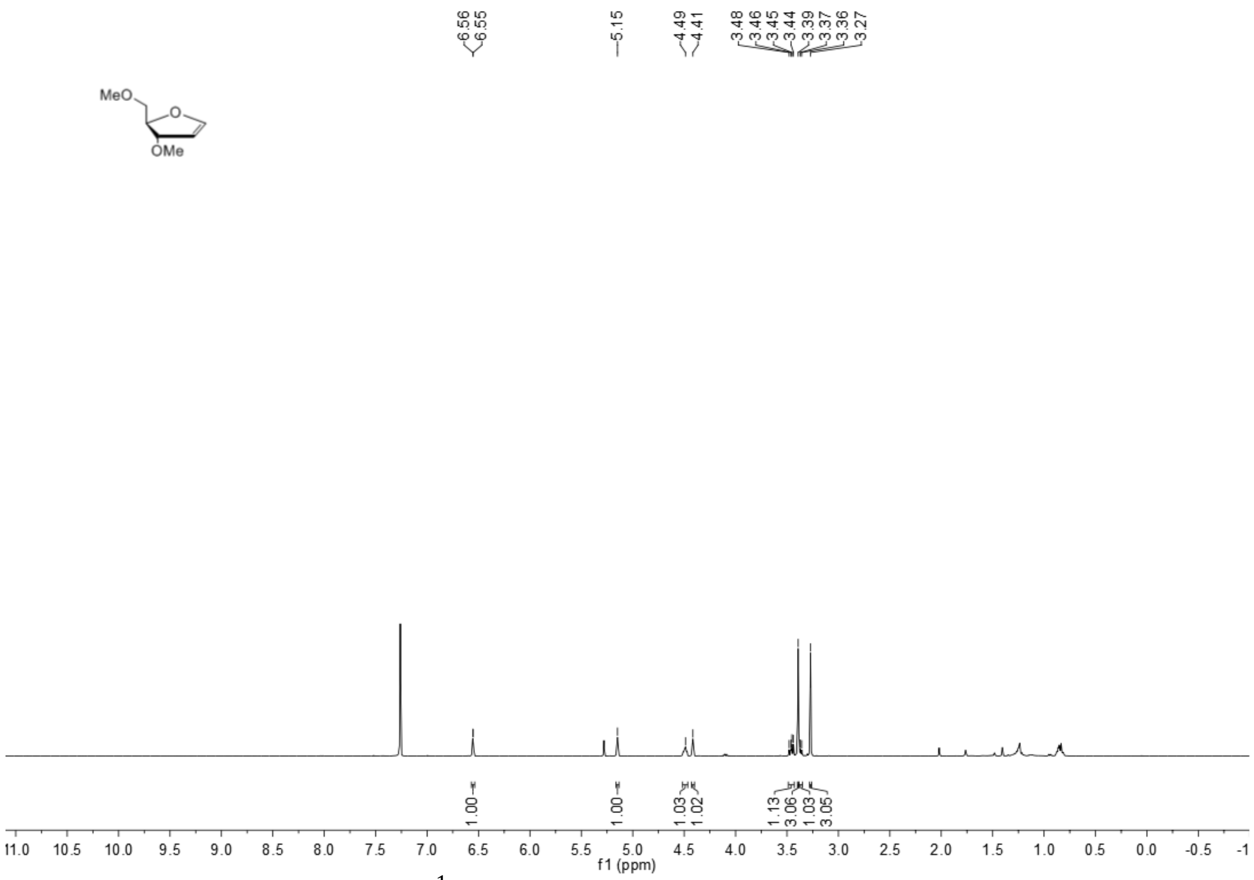

^{1}H NMR (CDCl$_3$-*d*) of **3o**

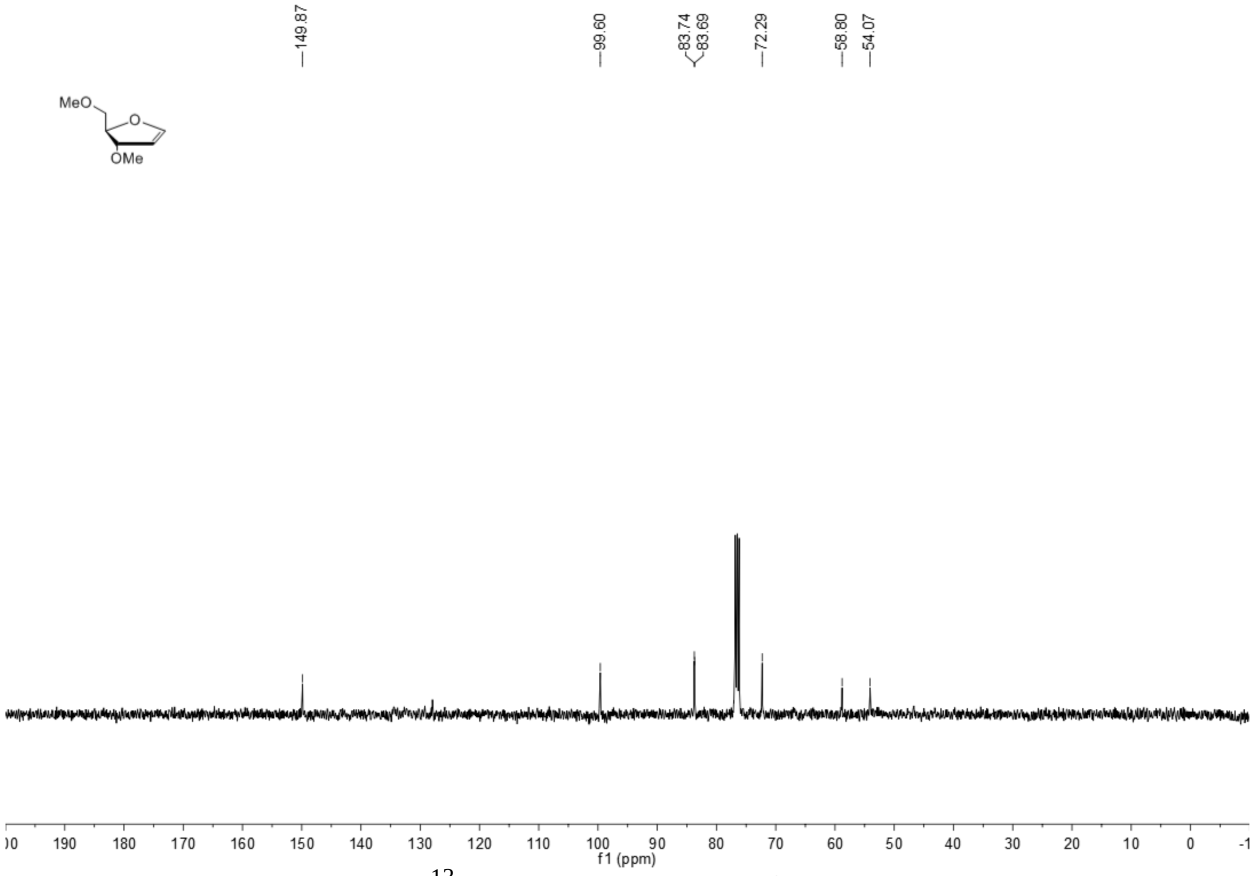

^{13}C NMR (CDCl$_3$-*d*) of **3o**

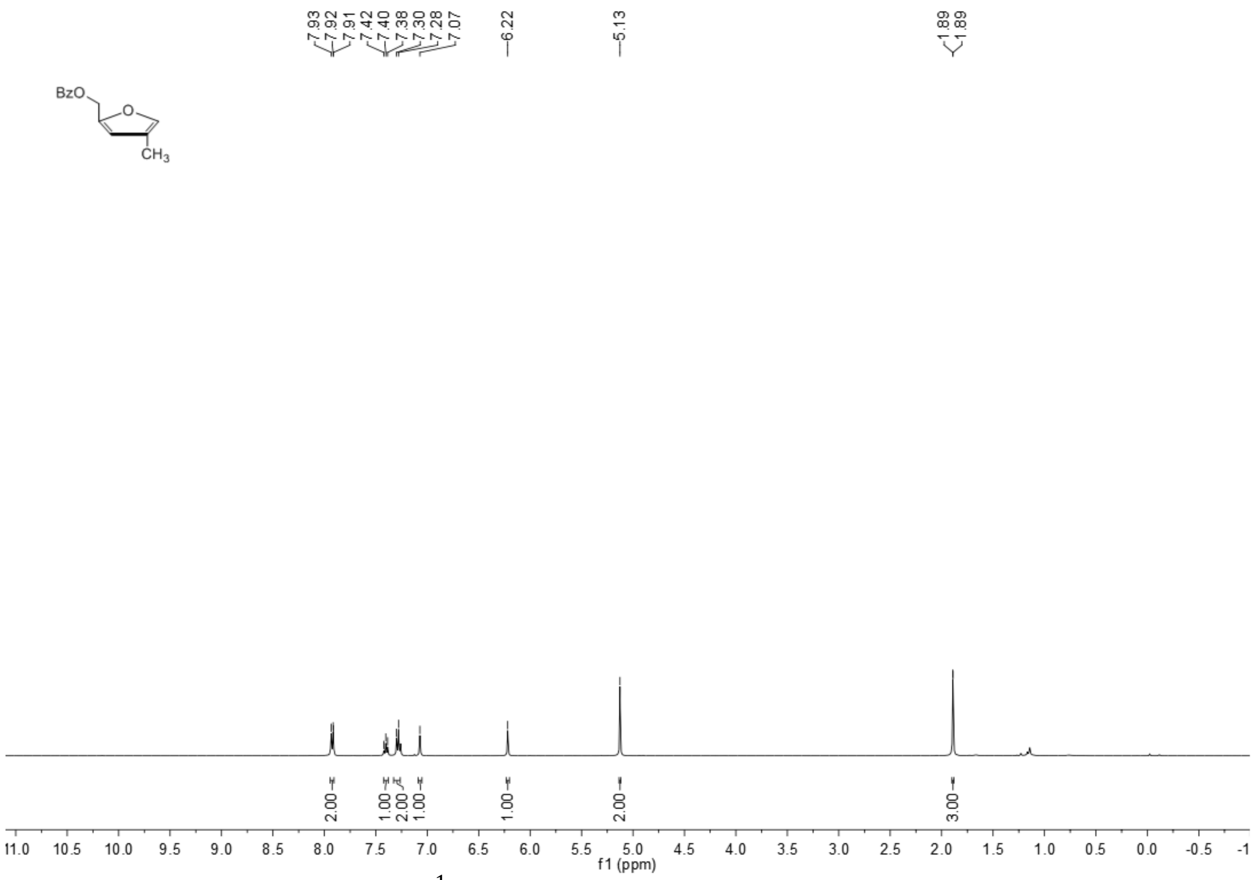

^{1}H NMR ($CDCl_3$-*d*) of **4p**

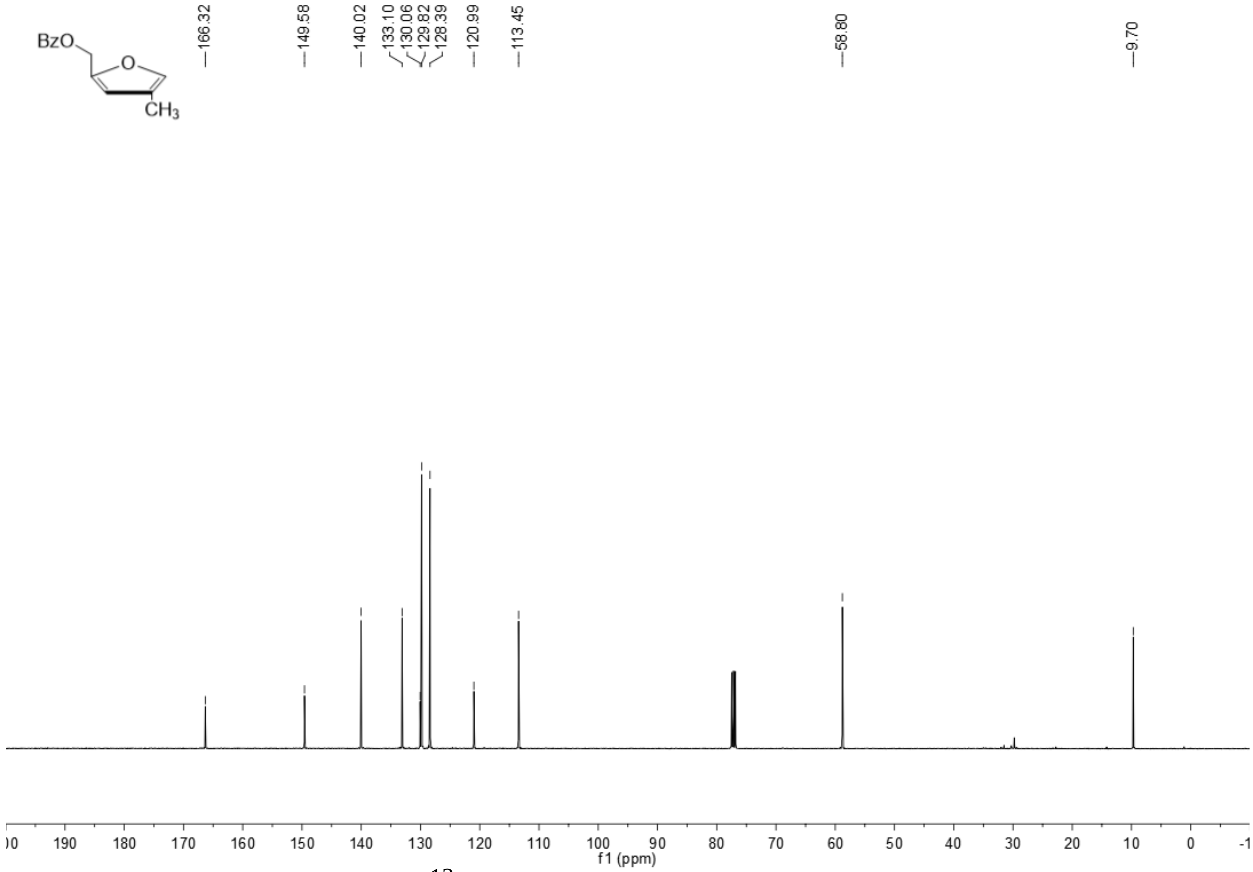

^{13}C NMR ($CDCl_3$-*d*) of **4p**

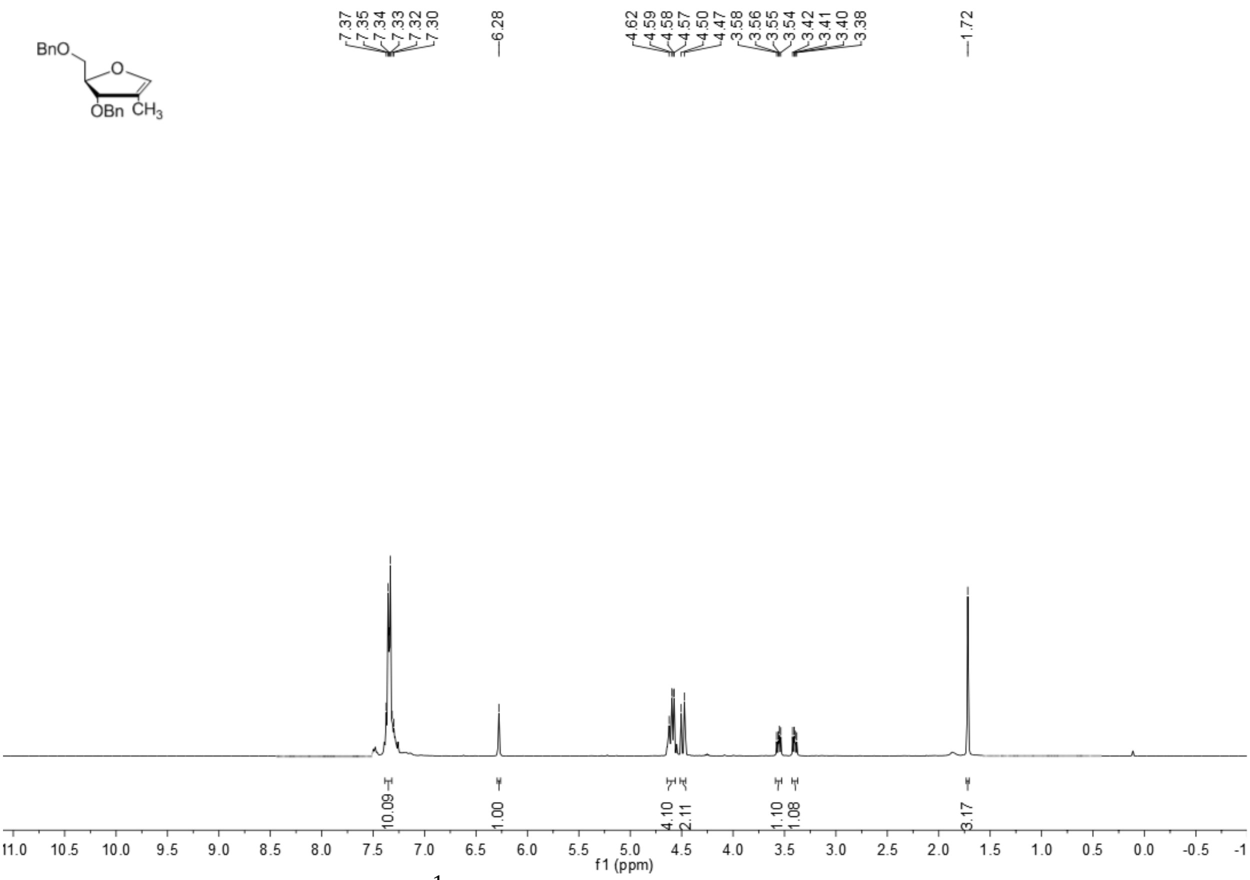

^{1}H NMR (CDCl$_3$-*d*) of **3q**

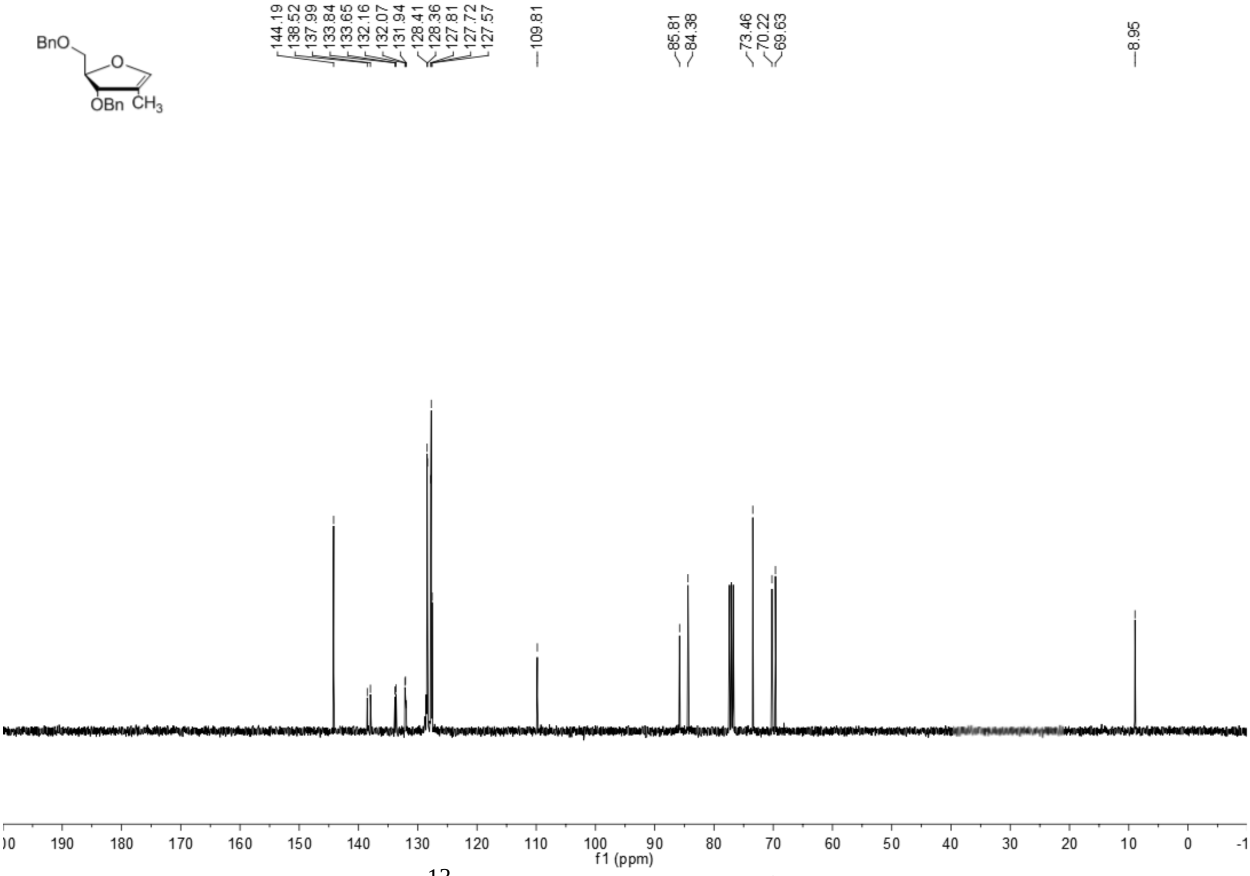

^{13}C NMR (CDCl$_3$-*d*) of **3q**

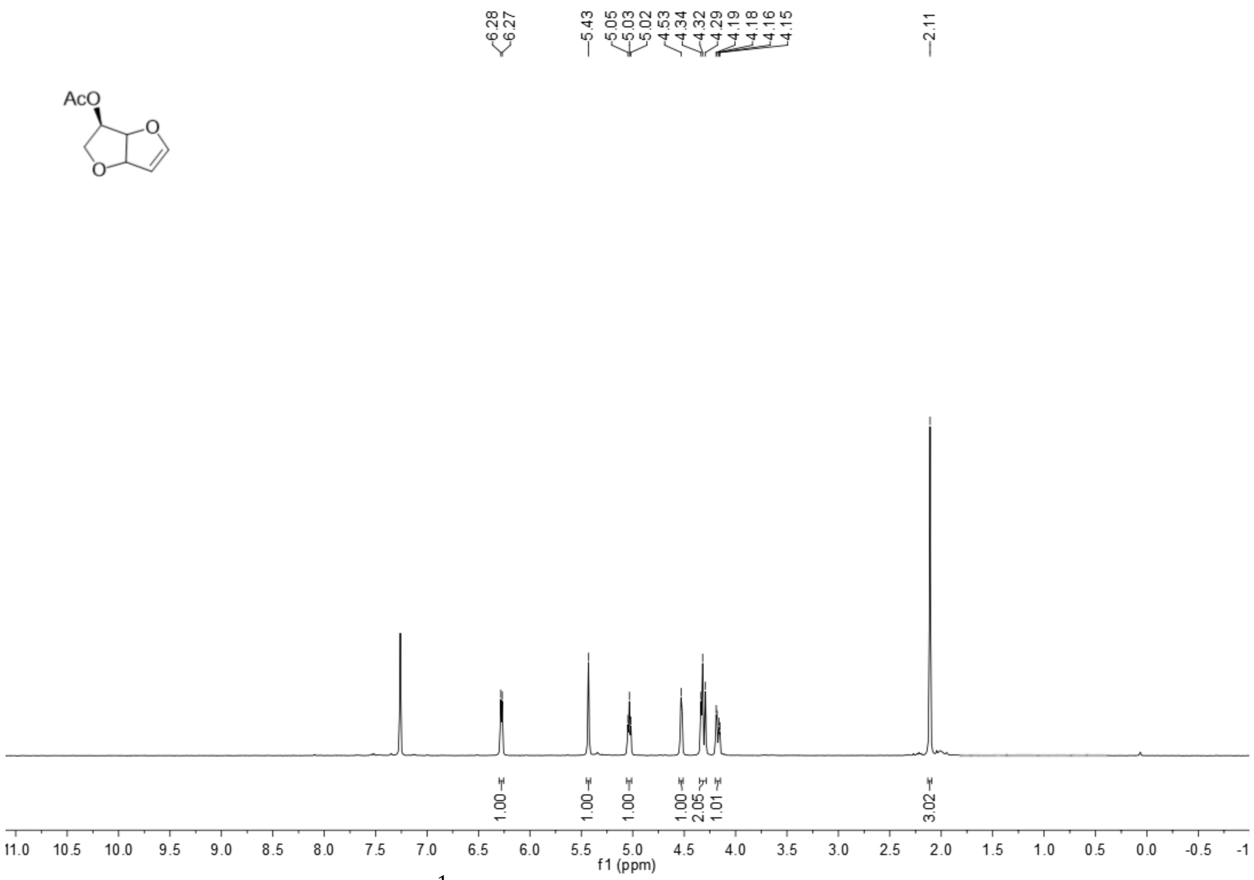

^{1}H NMR ($CDCl_3$-*d*) of **3r**

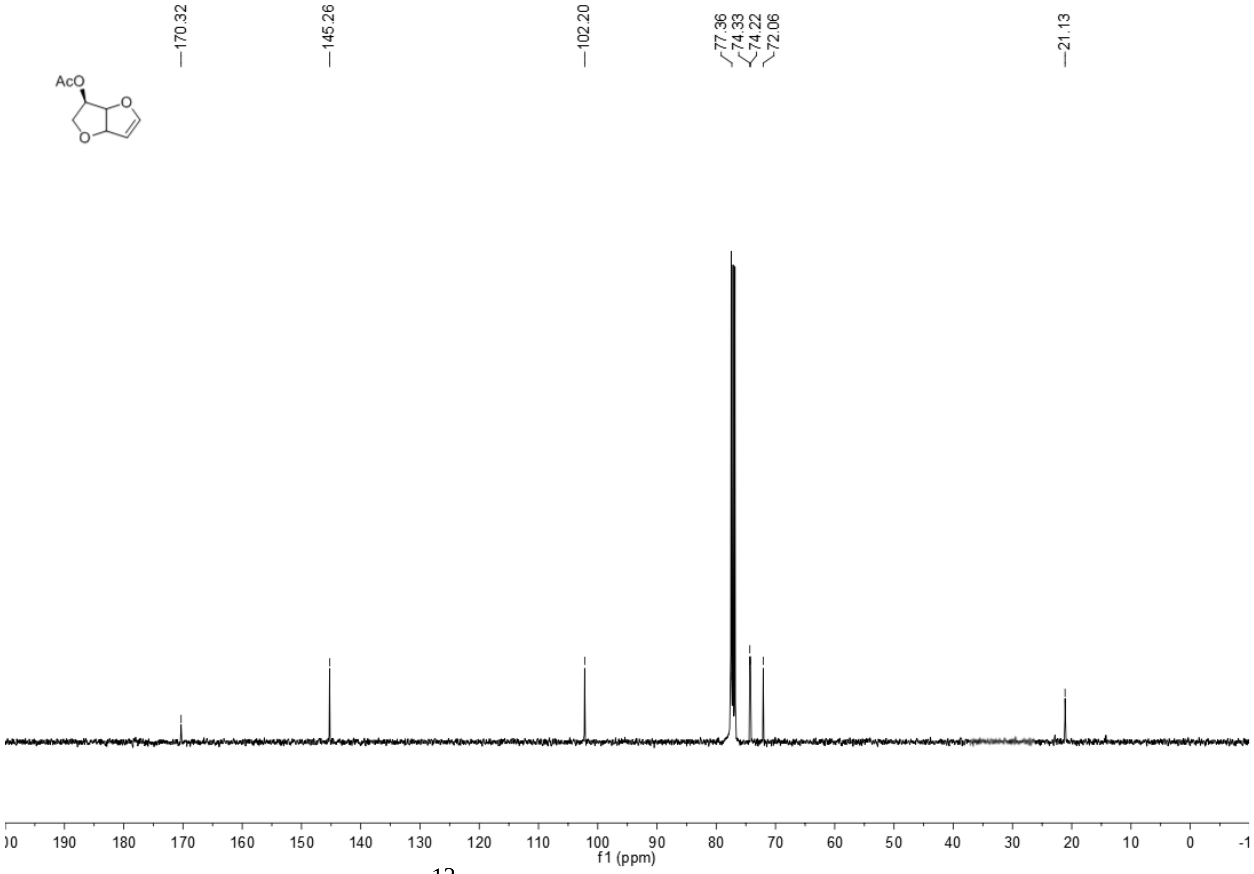

^{13}C NMR ($CDCl_3$-*d*) of **3r**

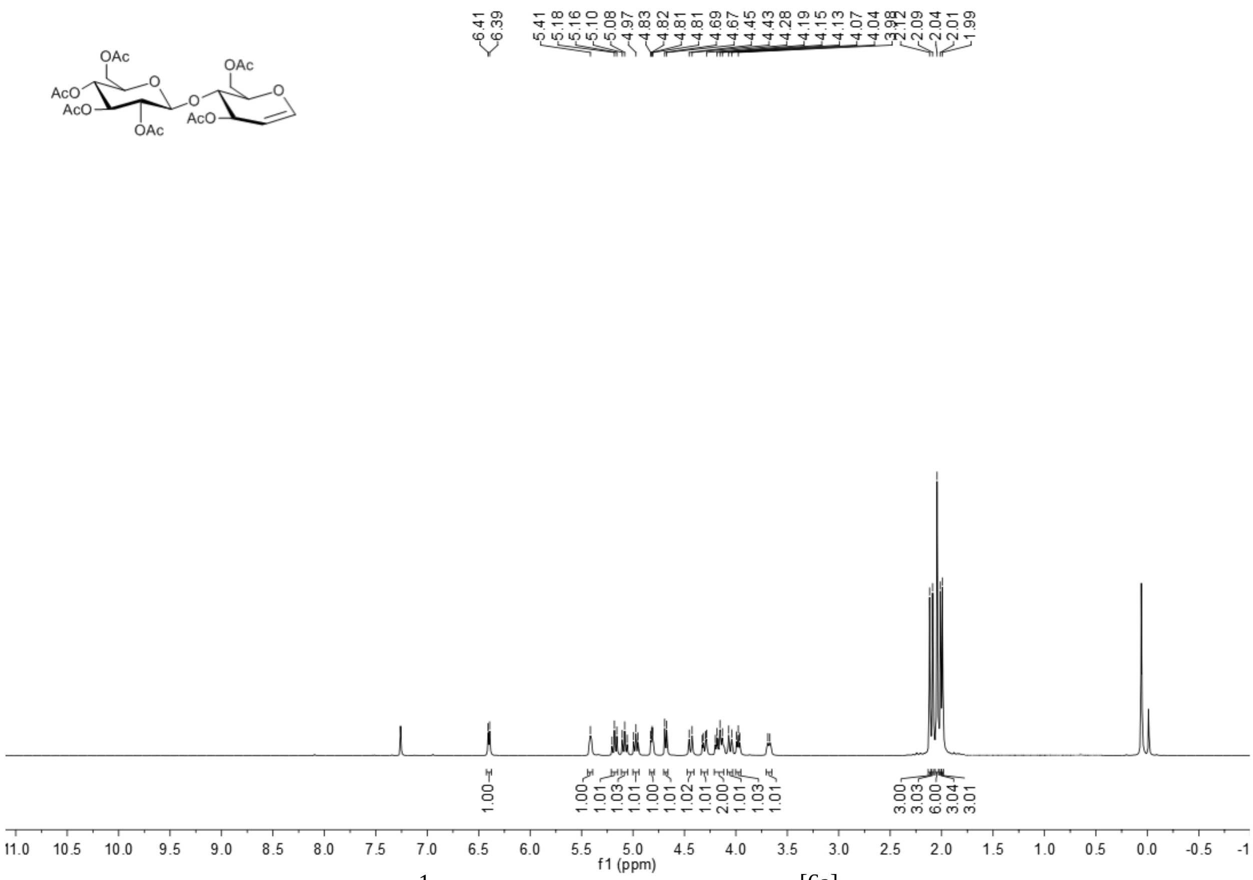

^{1}H NMR ($CDCl_3$-*d*) of **3s**[6a]

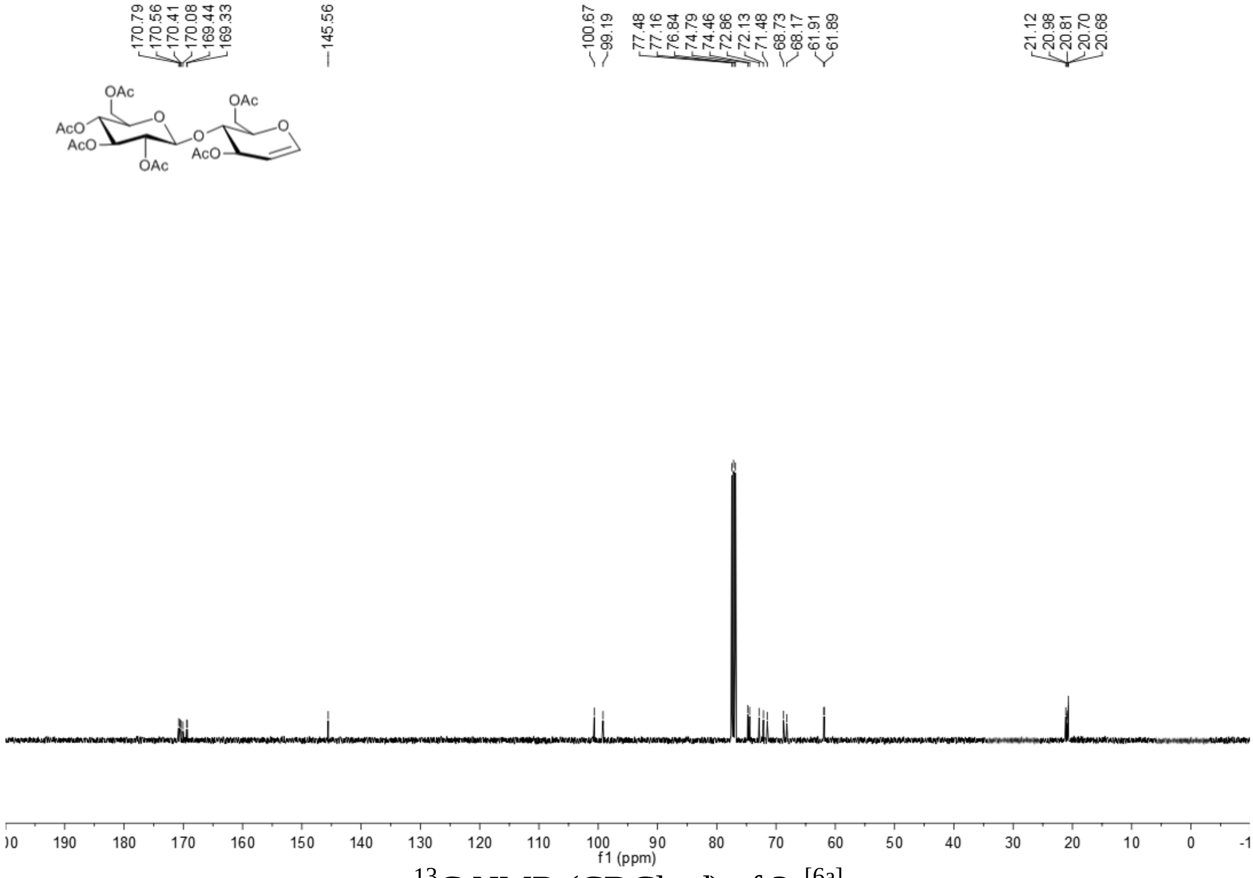

^{13}C NMR ($CDCl_3$-*d*) of **3s**[6a]

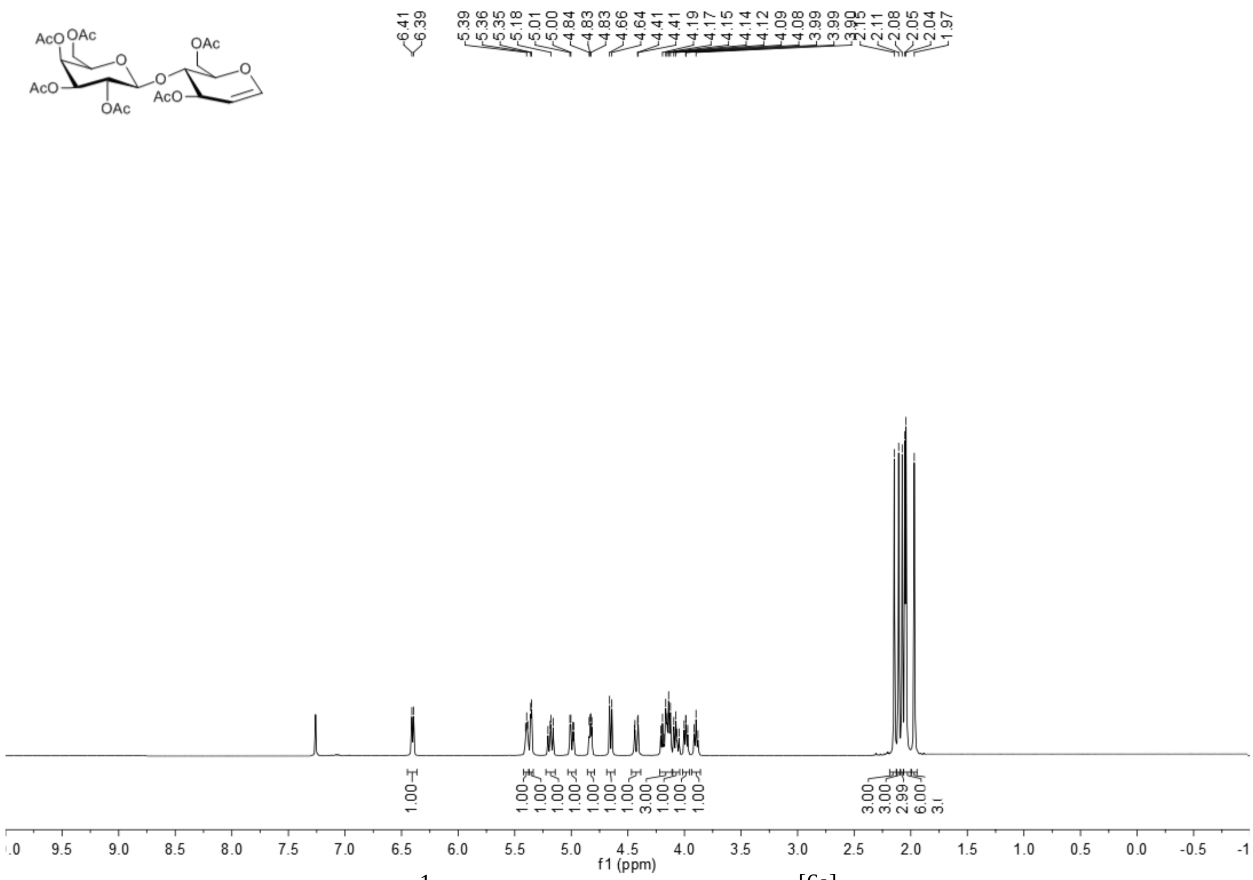

^{1}H NMR ($CDCl_3$-*d*) of **3t**[6a]

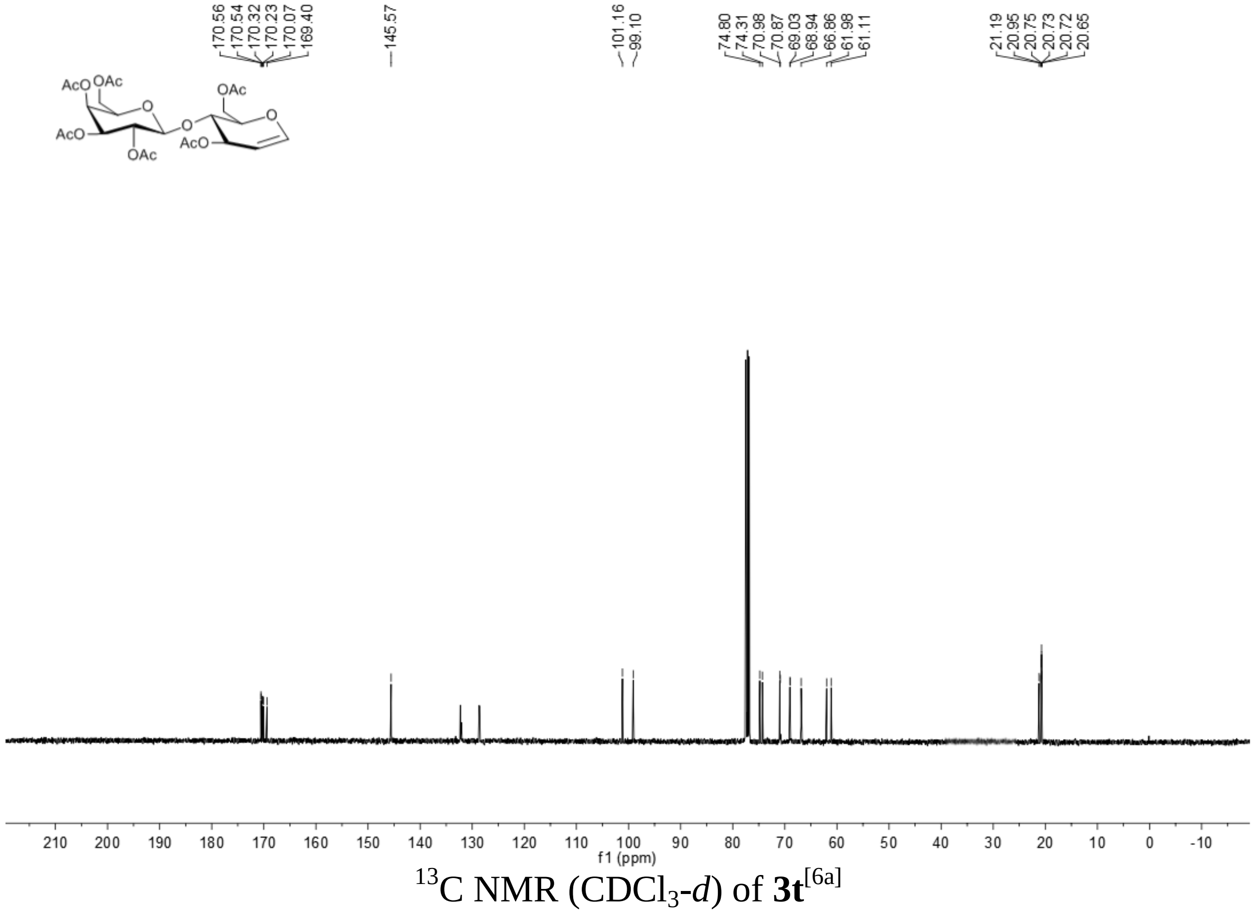

^{13}C NMR ($CDCl_3$-*d*) of **3t**[6a]

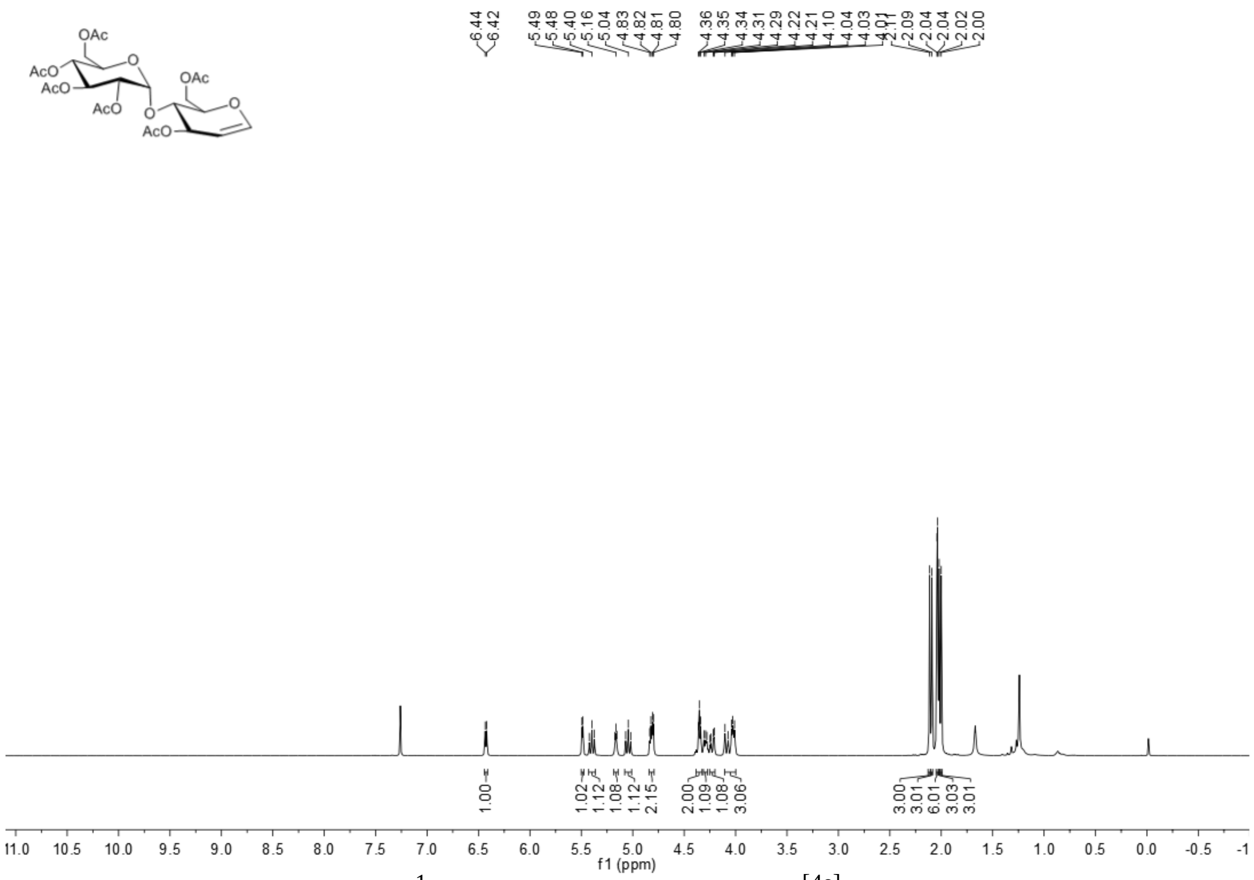

^{1}H NMR ($CDCl_3$-*d*) of **3u**[4e]

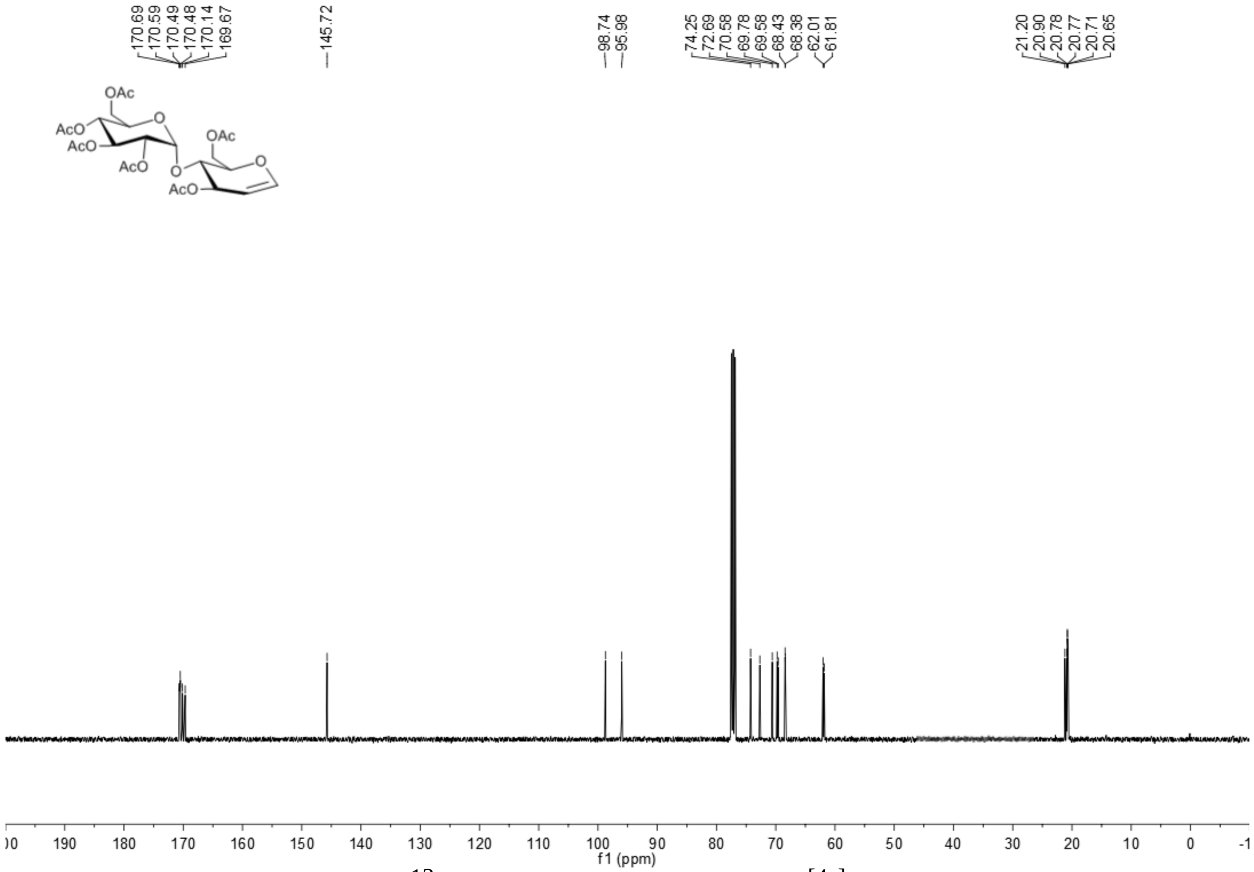

^{13}C NMR ($CDCl_3$-*d*) of **3u**[4e]

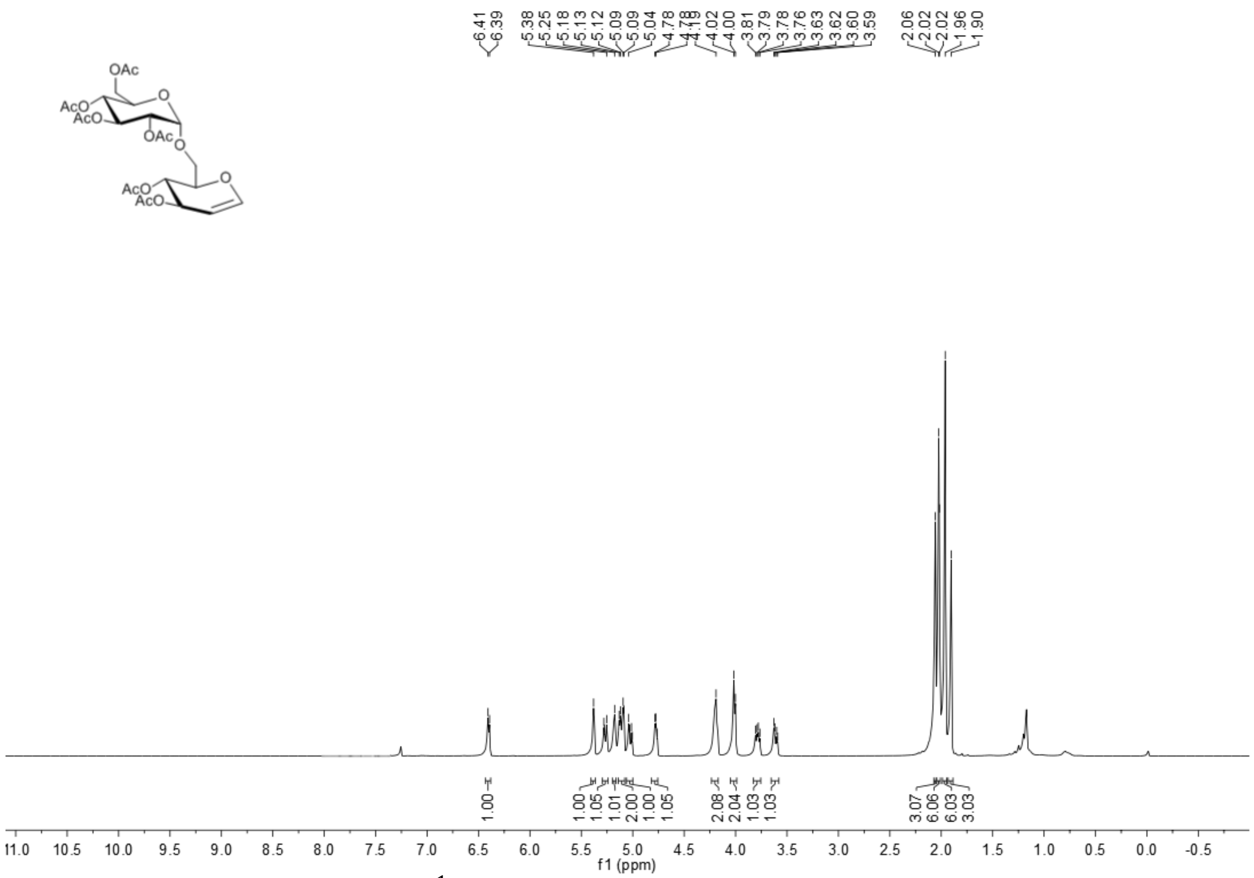

^{1}H NMR ($CDCl_3$-*d*) of **3v**

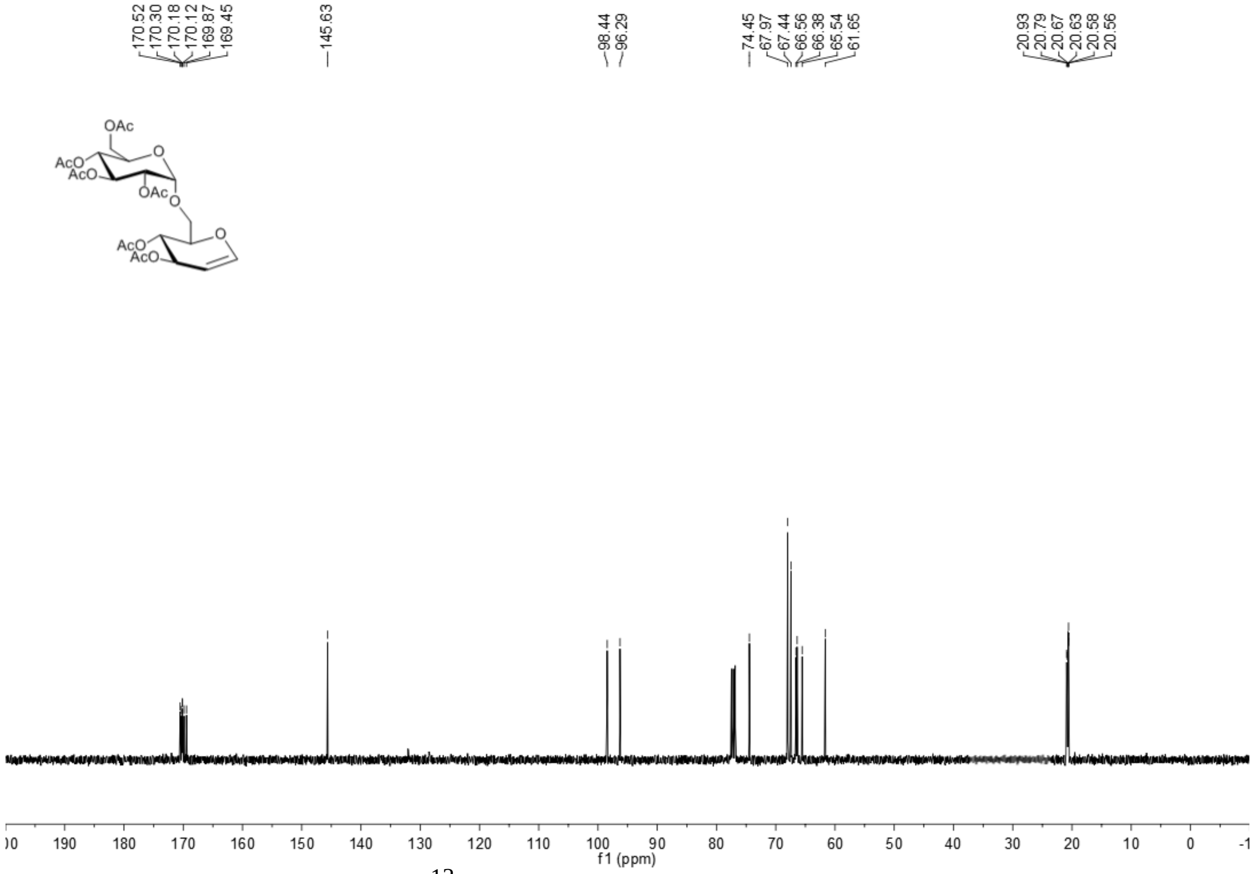

^{13}C NMR ($CDCl_3$-*d*) of **3v**

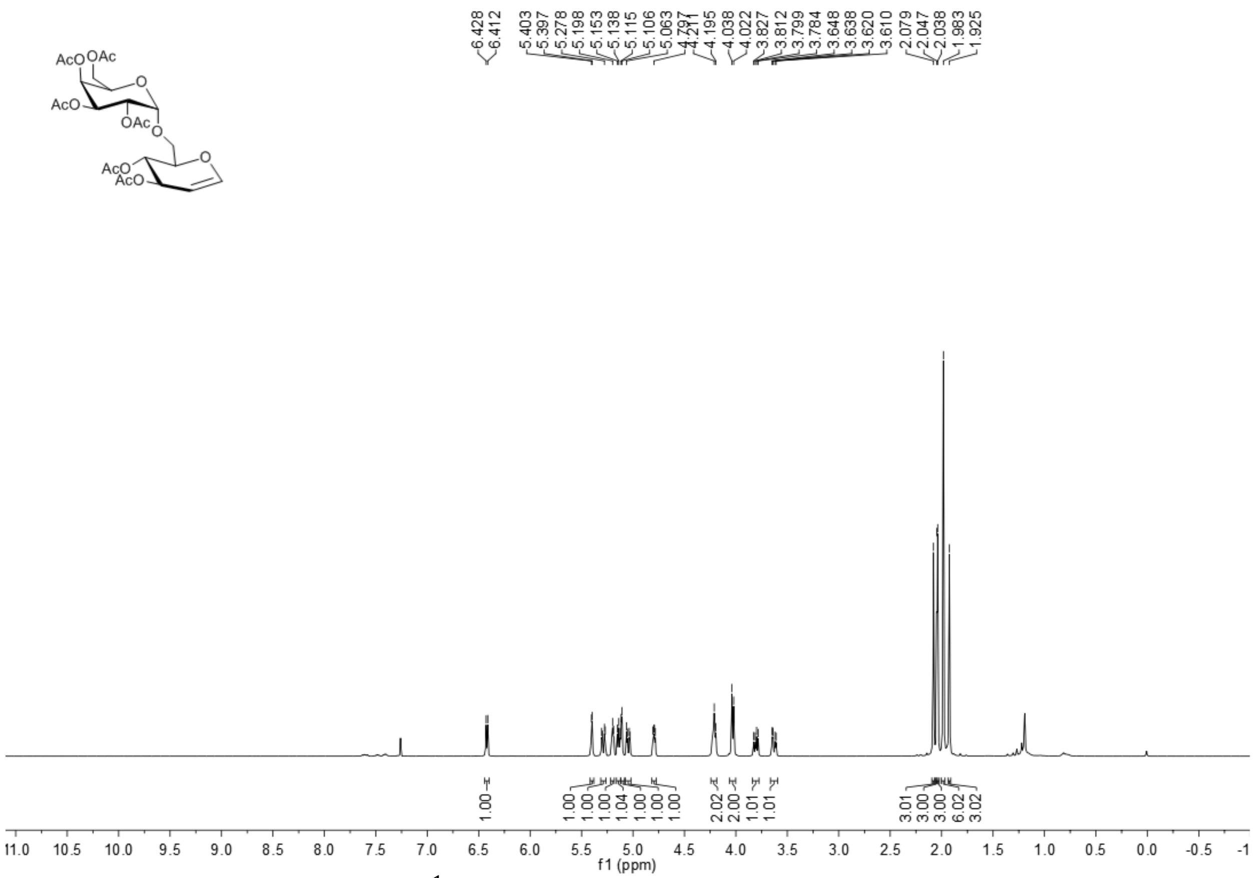

^{1}H NMR ($CDCl_3$-*d*) of **3w**

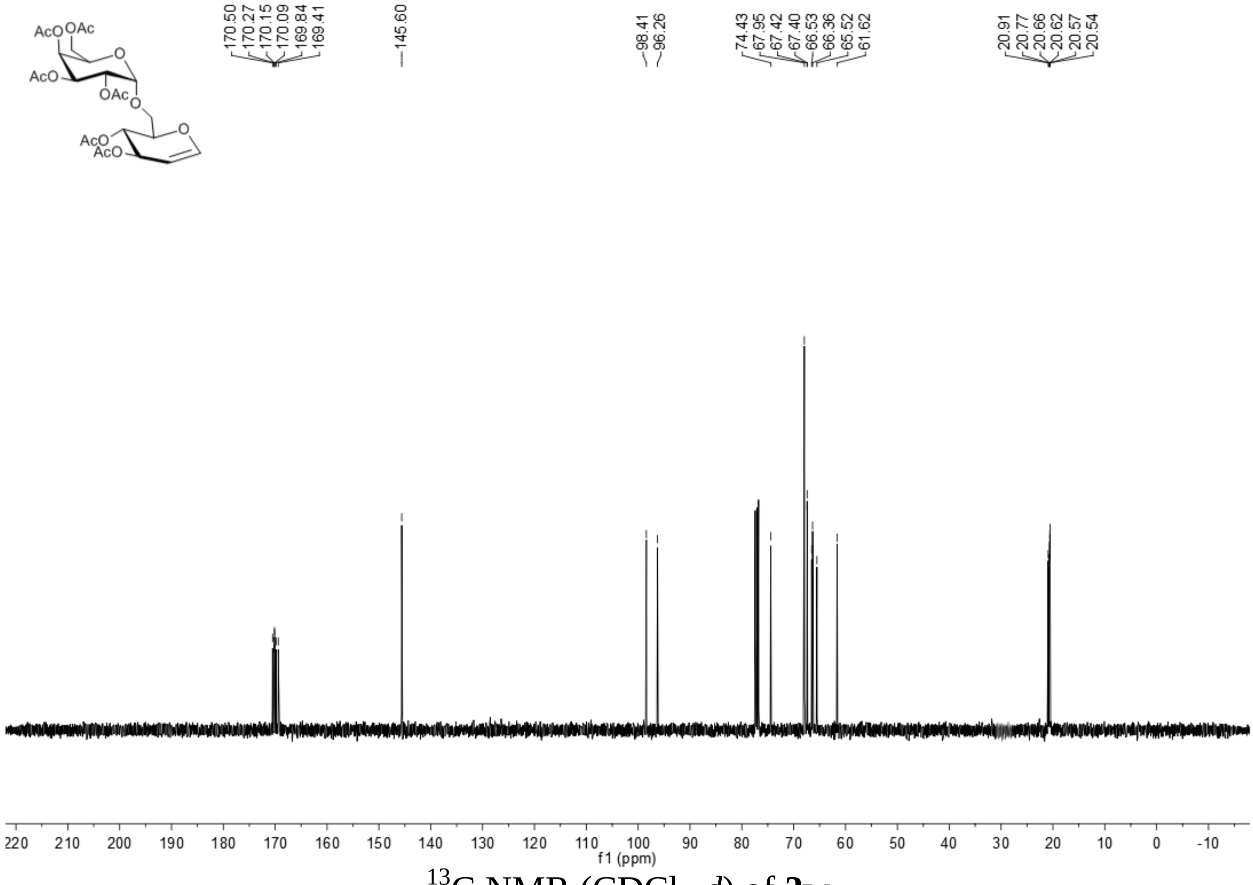

^{13}C NMR ($CDCl_3$-*d*) of **3w**

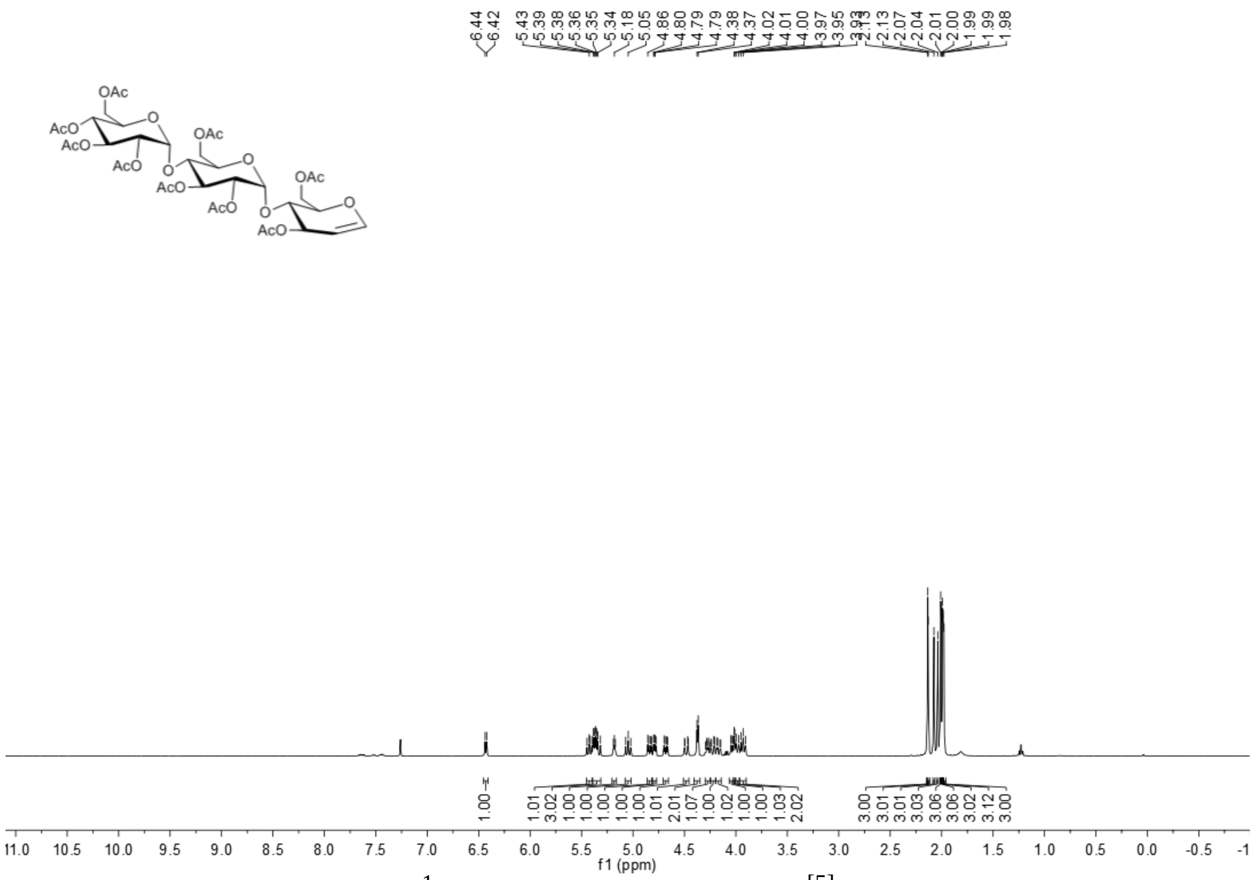

^{1}H NMR ($CDCl_3$-*d*) of **3x**[5]

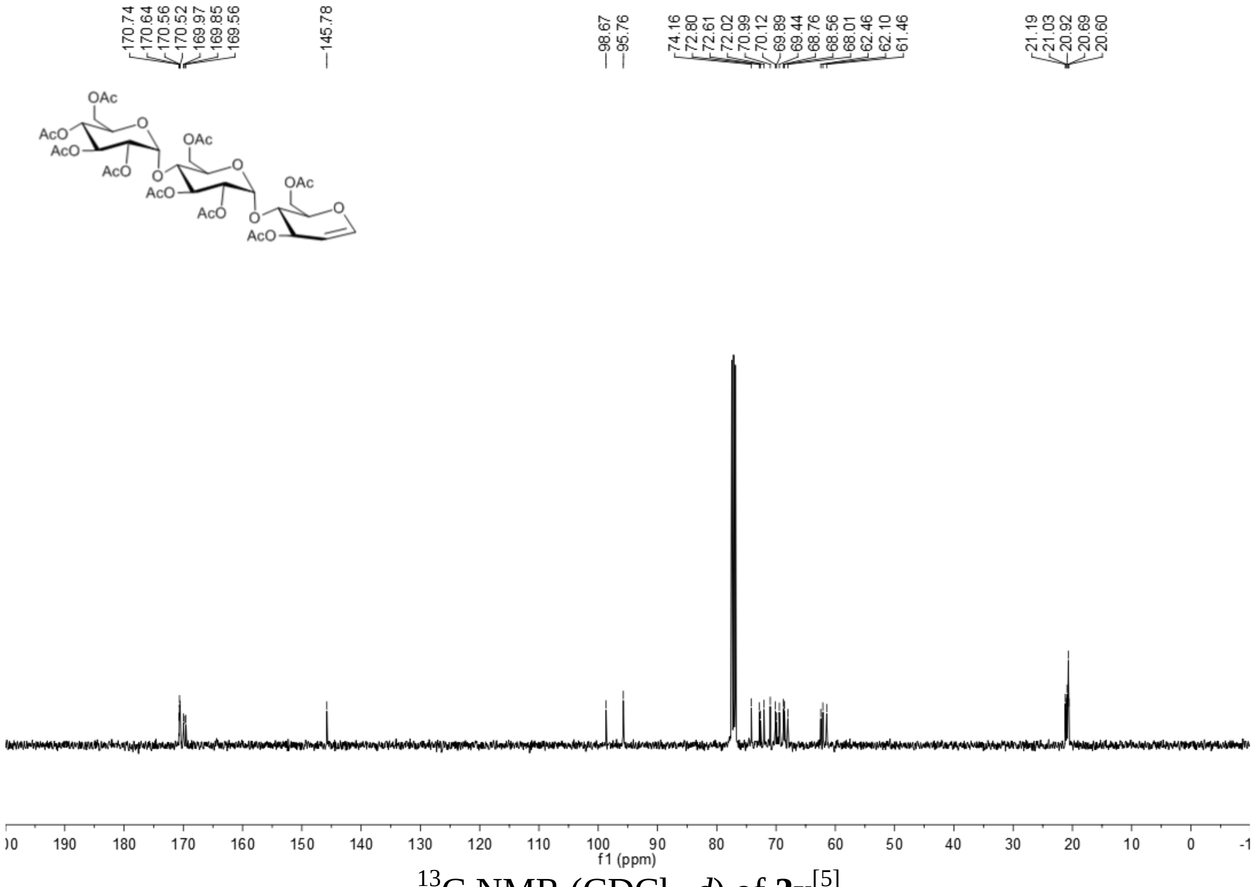

^{13}C NMR ($CDCl_3$-*d*) of **3x**[5]

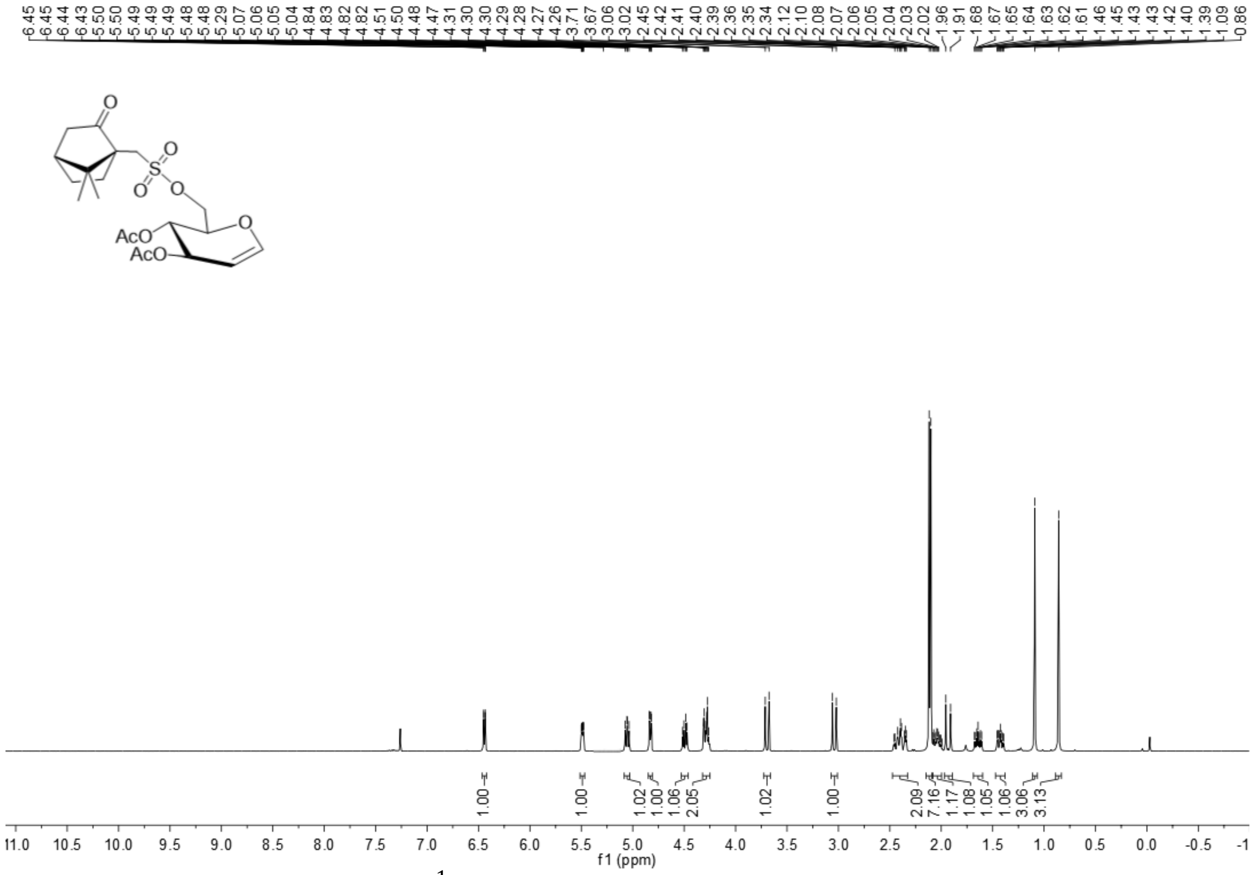

^{1}H NMR ($CDCl_3$-*d*) of **3y**

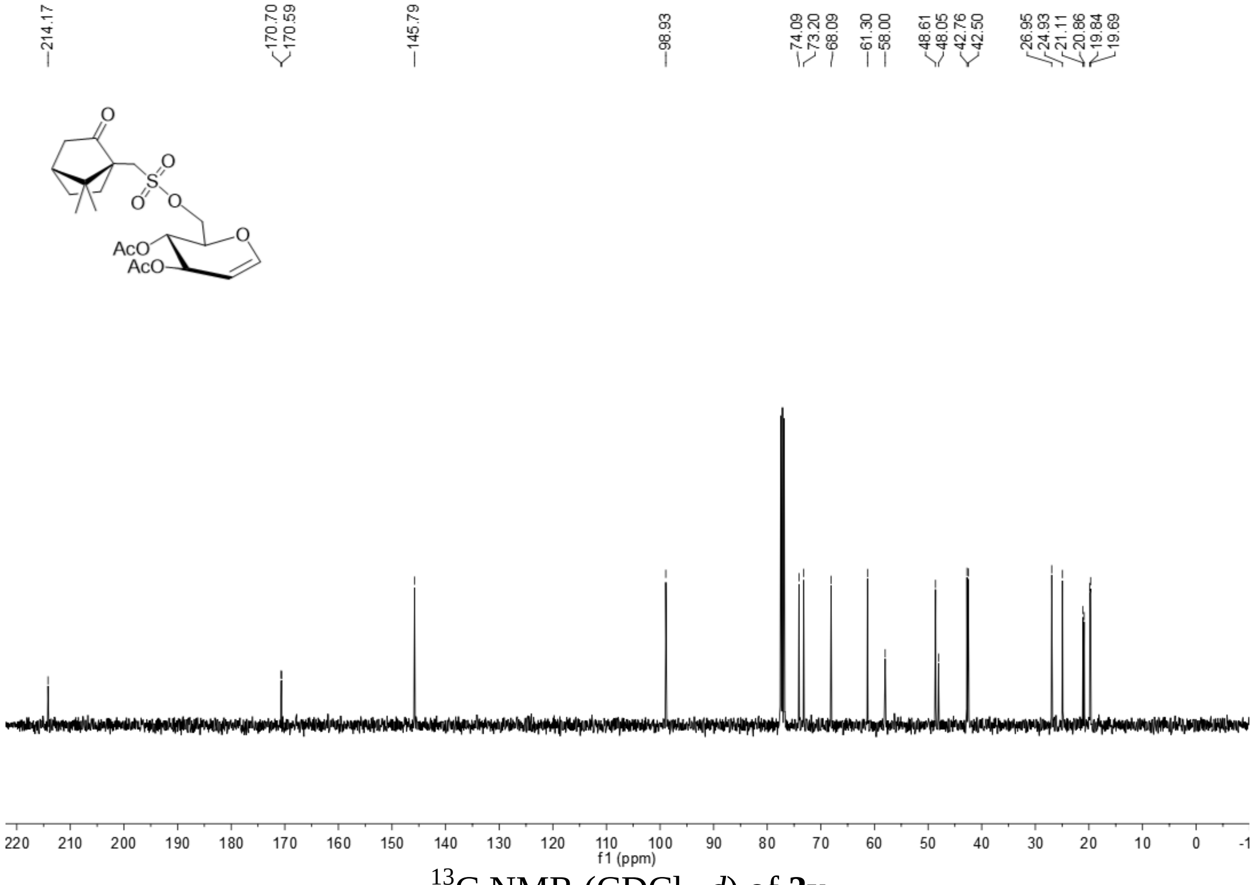

^{13}C NMR ($CDCl_3$-*d*) of **3y**

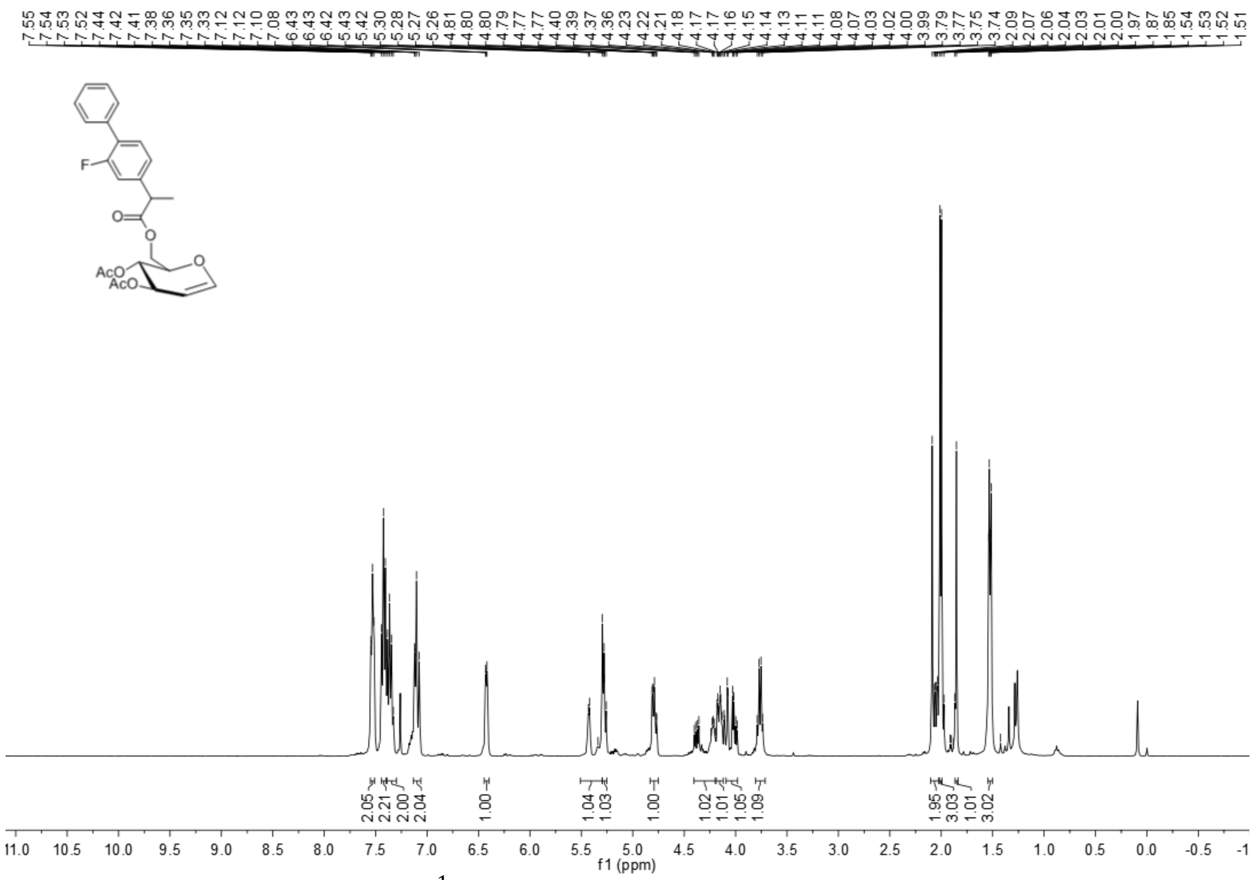

^{1}H NMR ($CDCl_3$-*d*) of **3z**

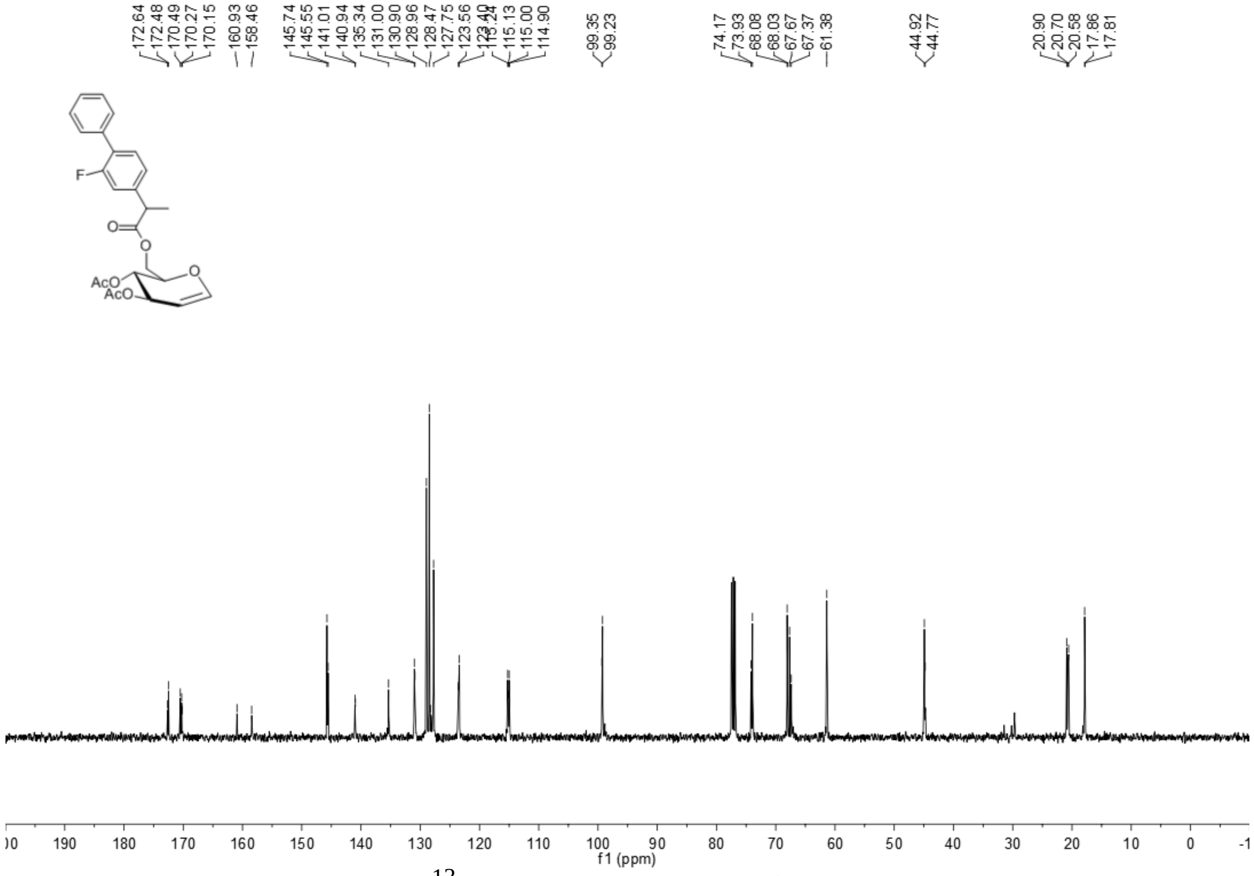

^{13}C NMR ($CDCl_3$-*d*) of **3z**

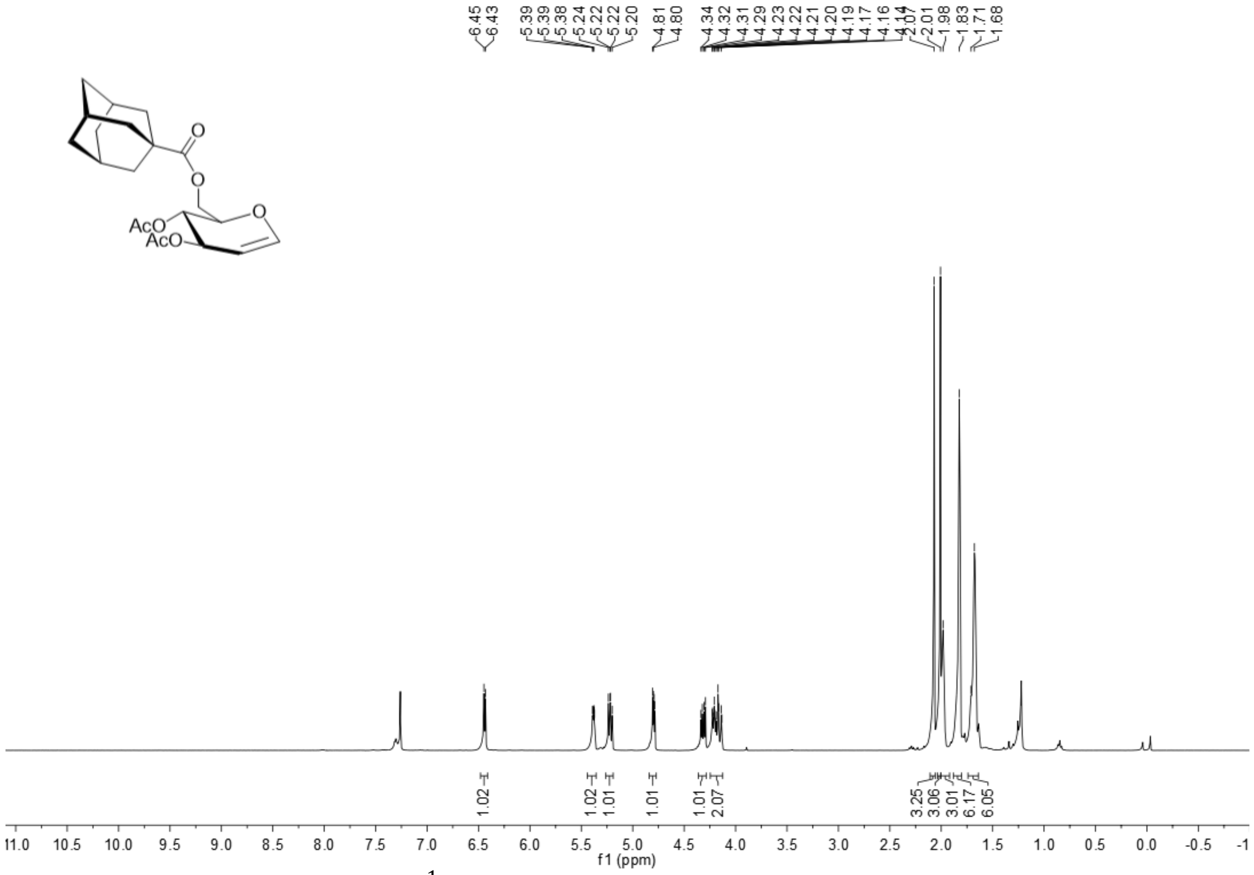

^{1}H NMR ($CDCl_3$-*d*) of **3aa**

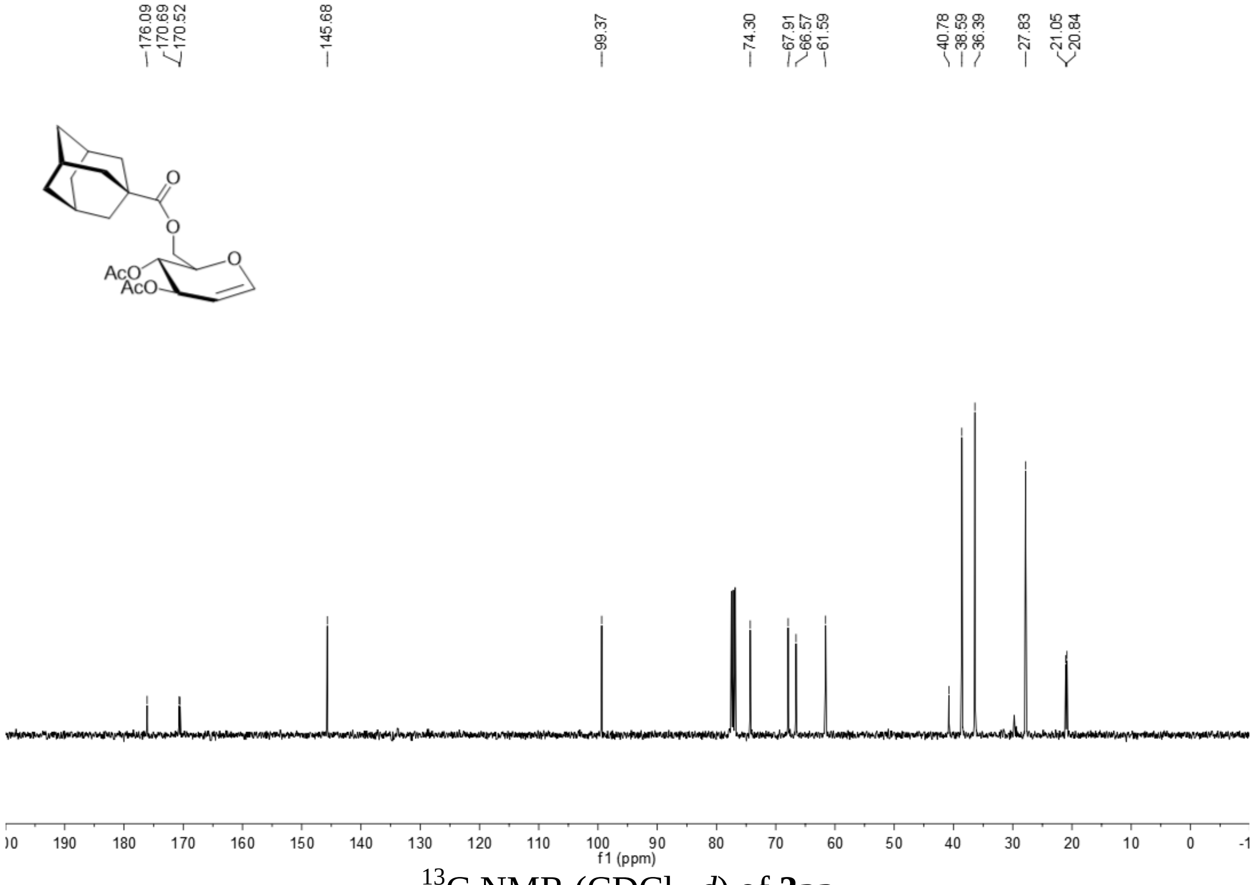

^{13}C NMR ($CDCl_3$-*d*) of **3aa**

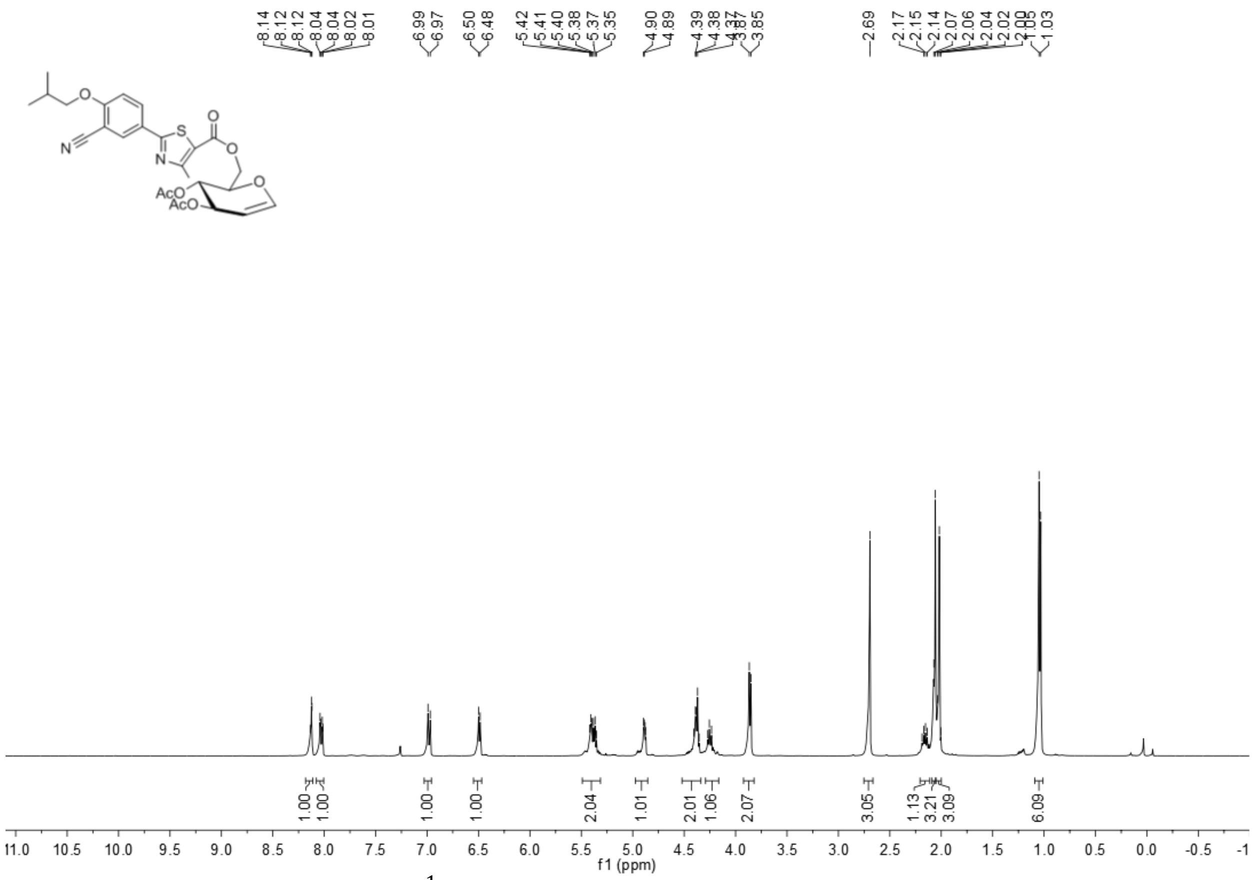

^{1}H NMR ($CDCl_3$-*d*) of **3ab**

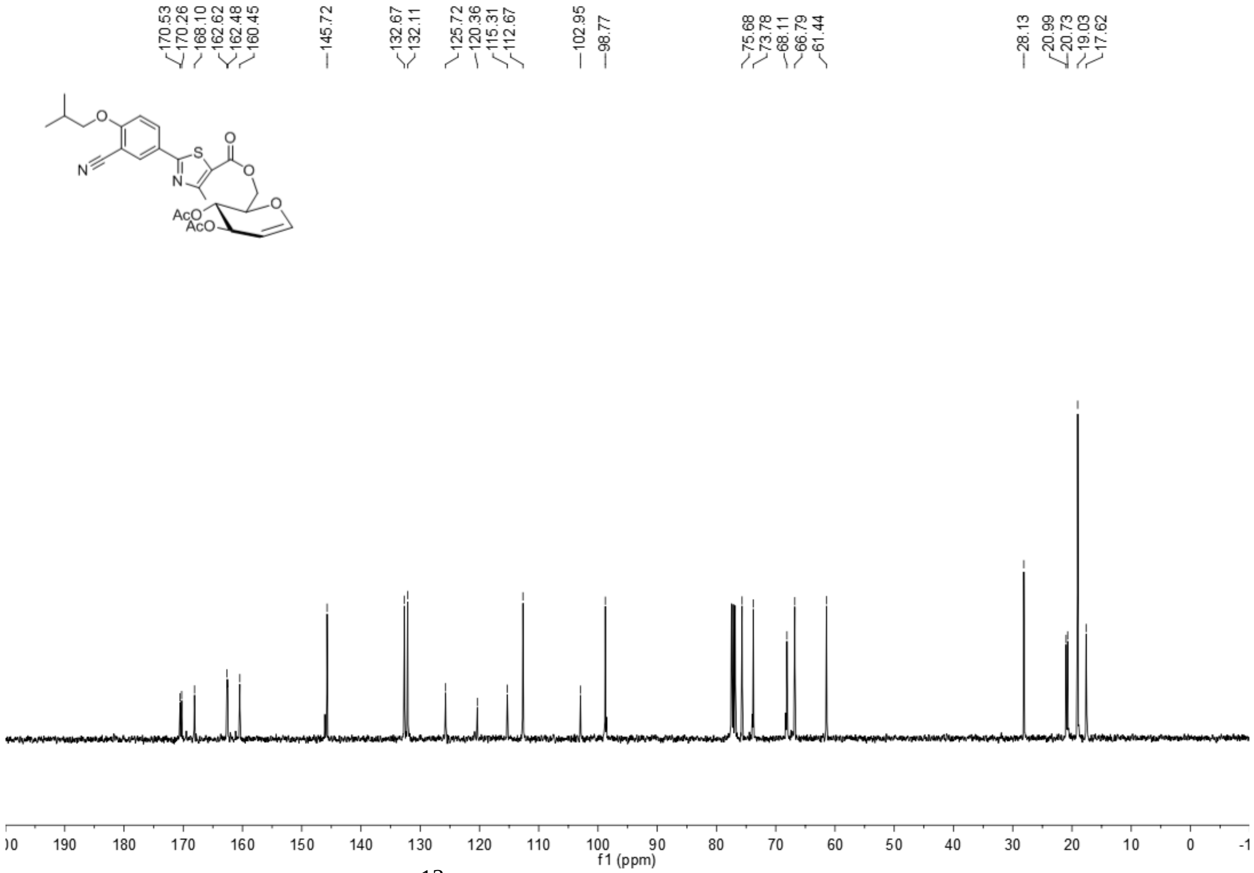

^{13}C NMR ($CDCl_3$-*d*) of **3ab**

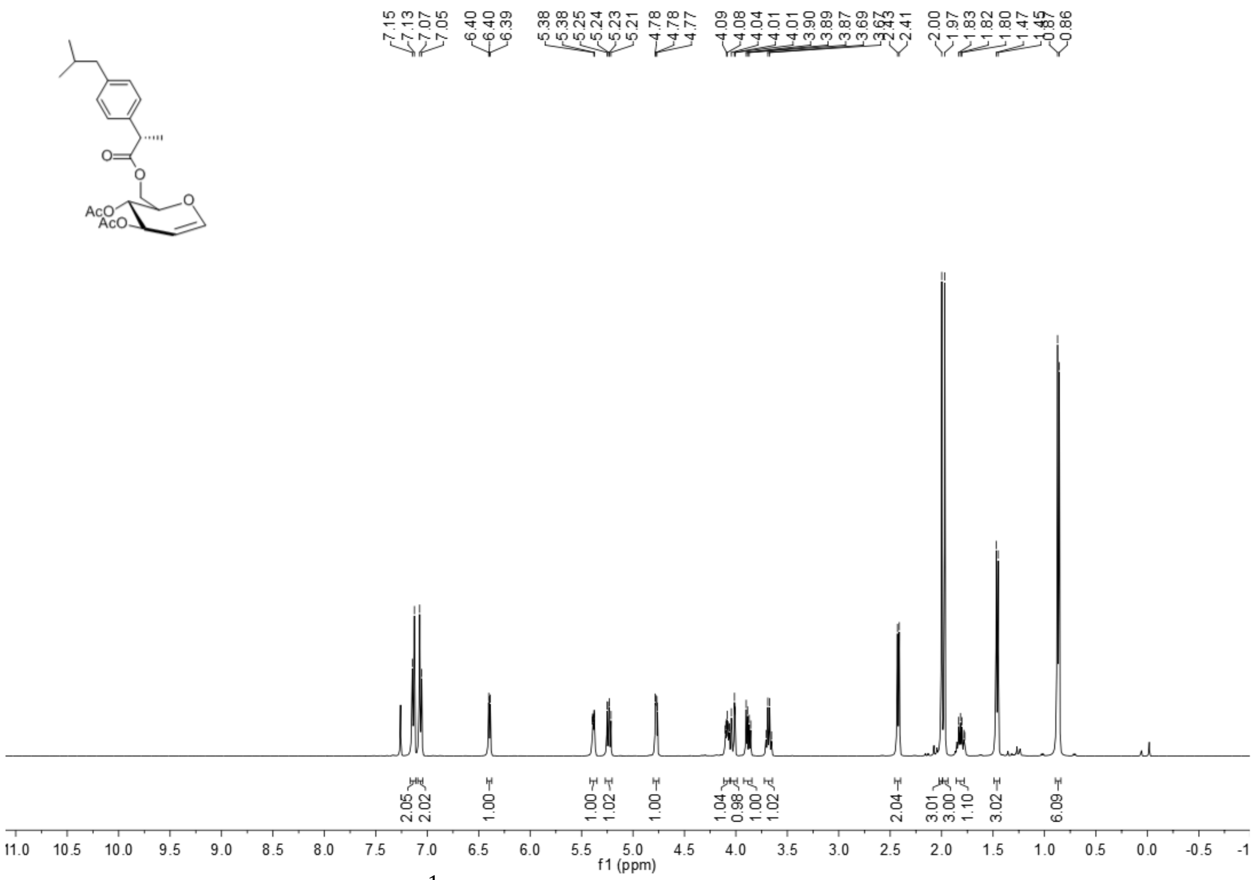

^{1}H NMR ($CDCl_3$-*d*) of **3ac**

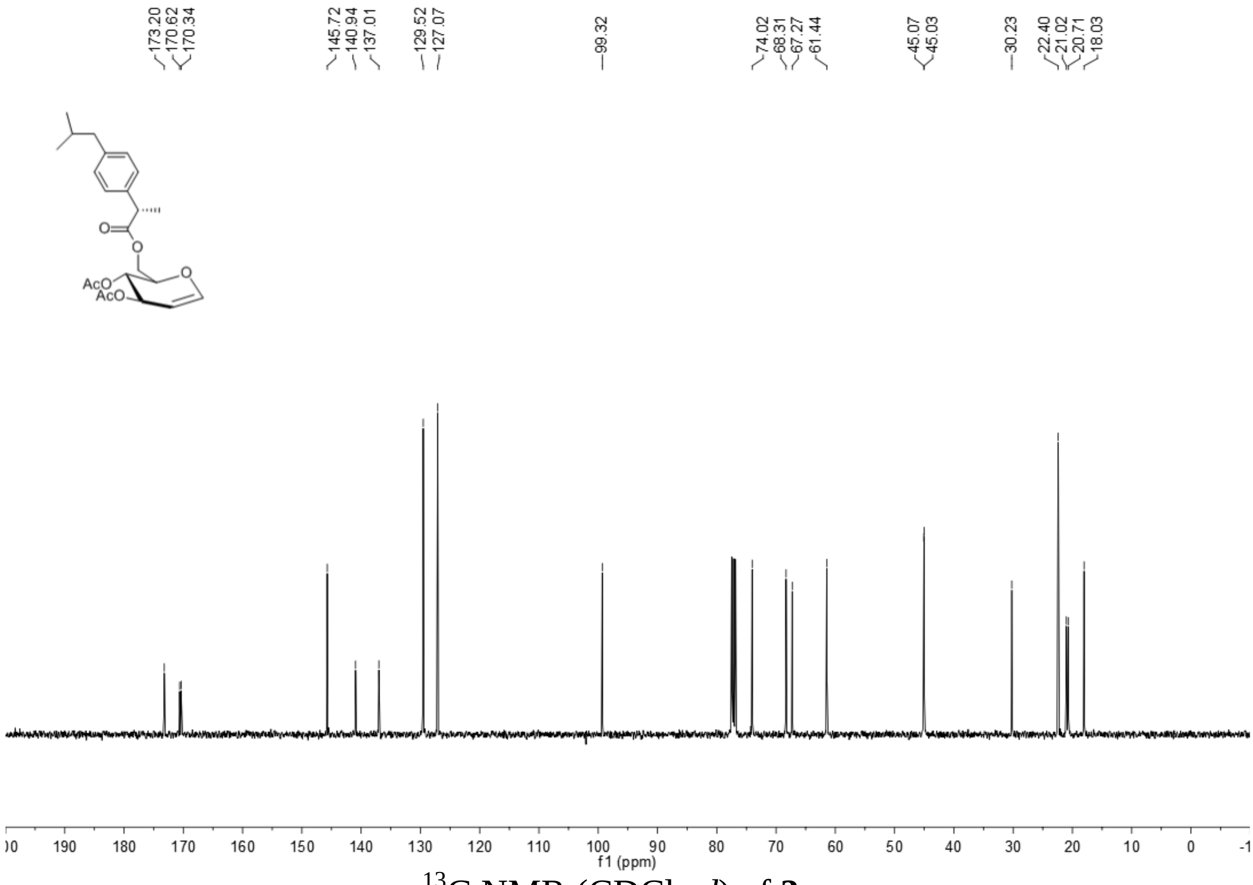

^{13}C NMR ($CDCl_3$-*d*) of **3ac**

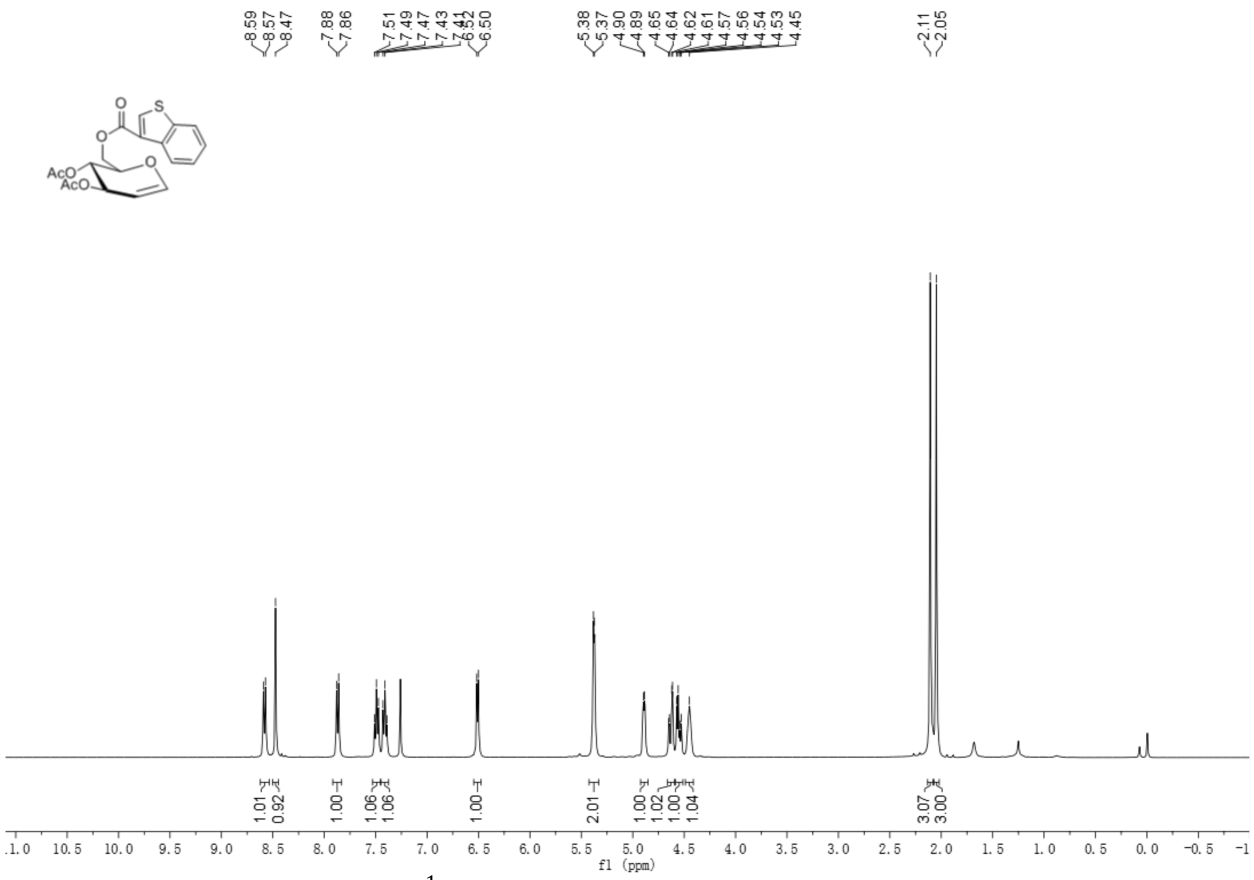

^{1}H NMR (CDCl$_3$-*d*) of **3ad**

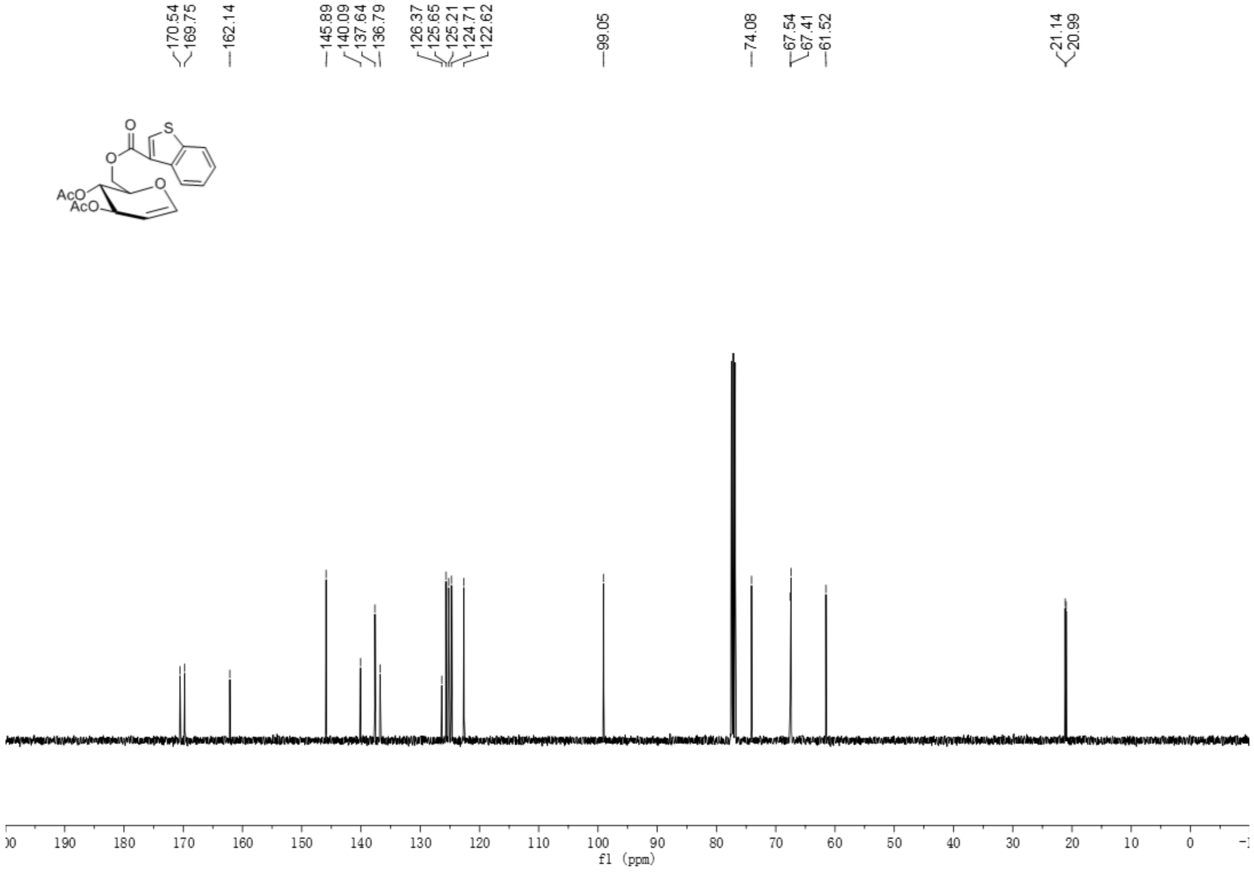

^{13}C NMR (CDCl$_3$-*d*) of **3ad**

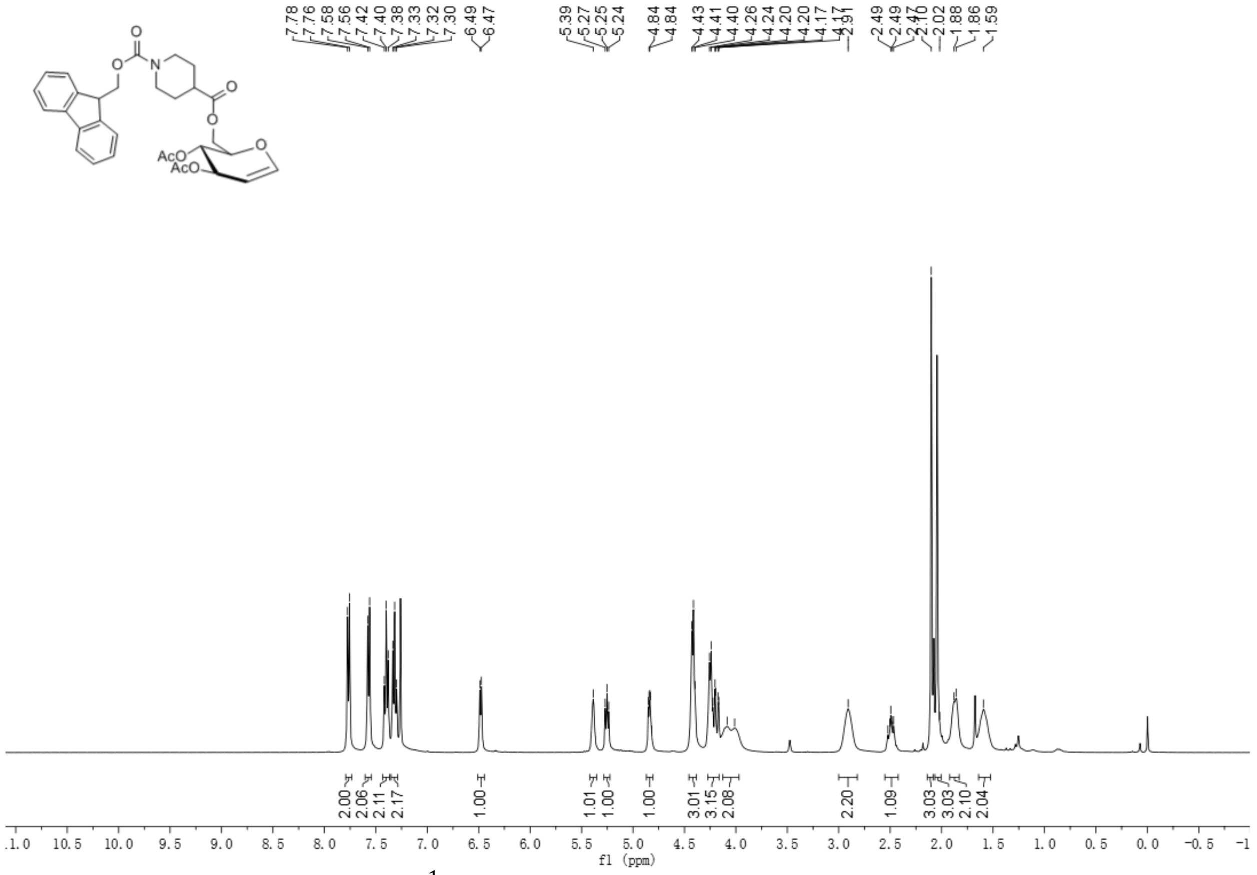

^{1}H NMR ($CDCl_3$-*d*) of **3ae**

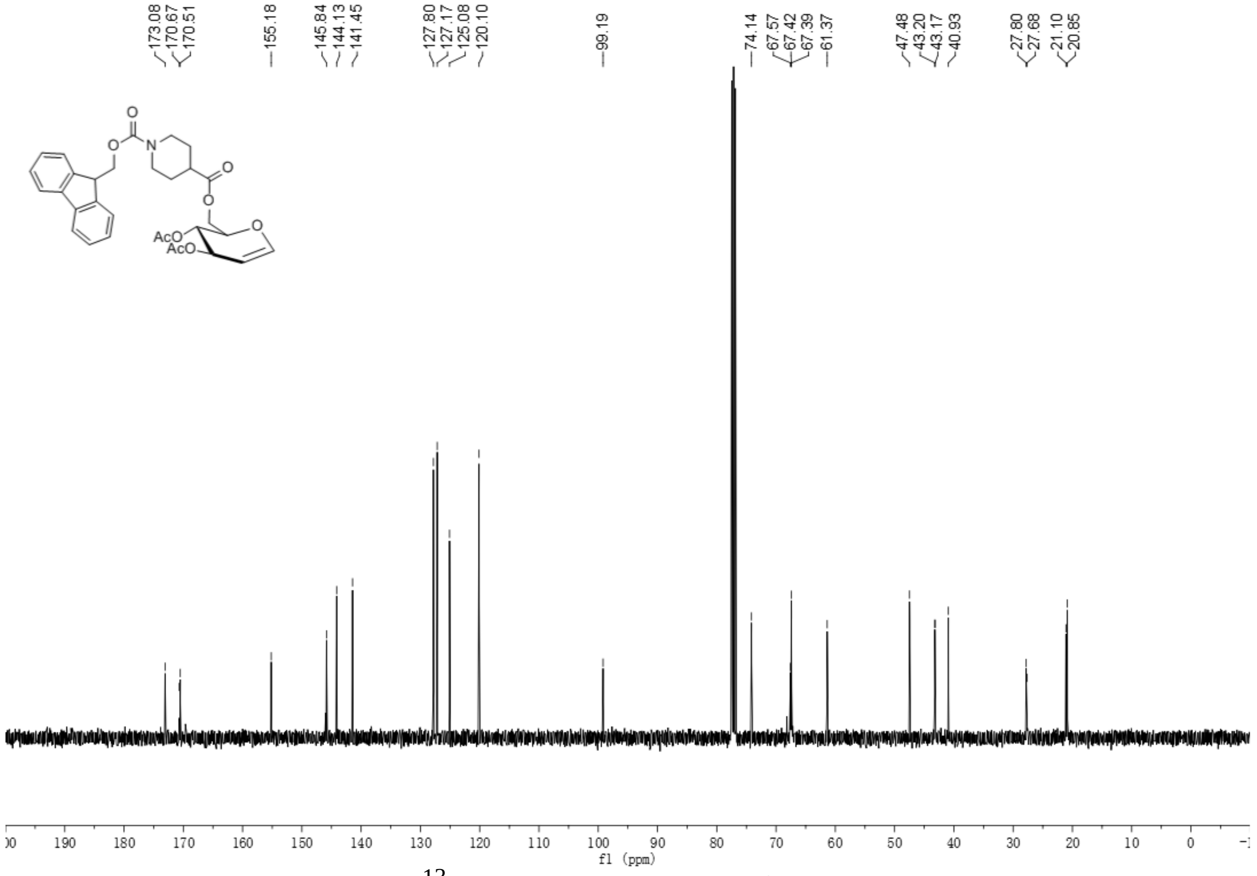

^{13}C NMR ($CDCl_3$-*d*) of **3ae**

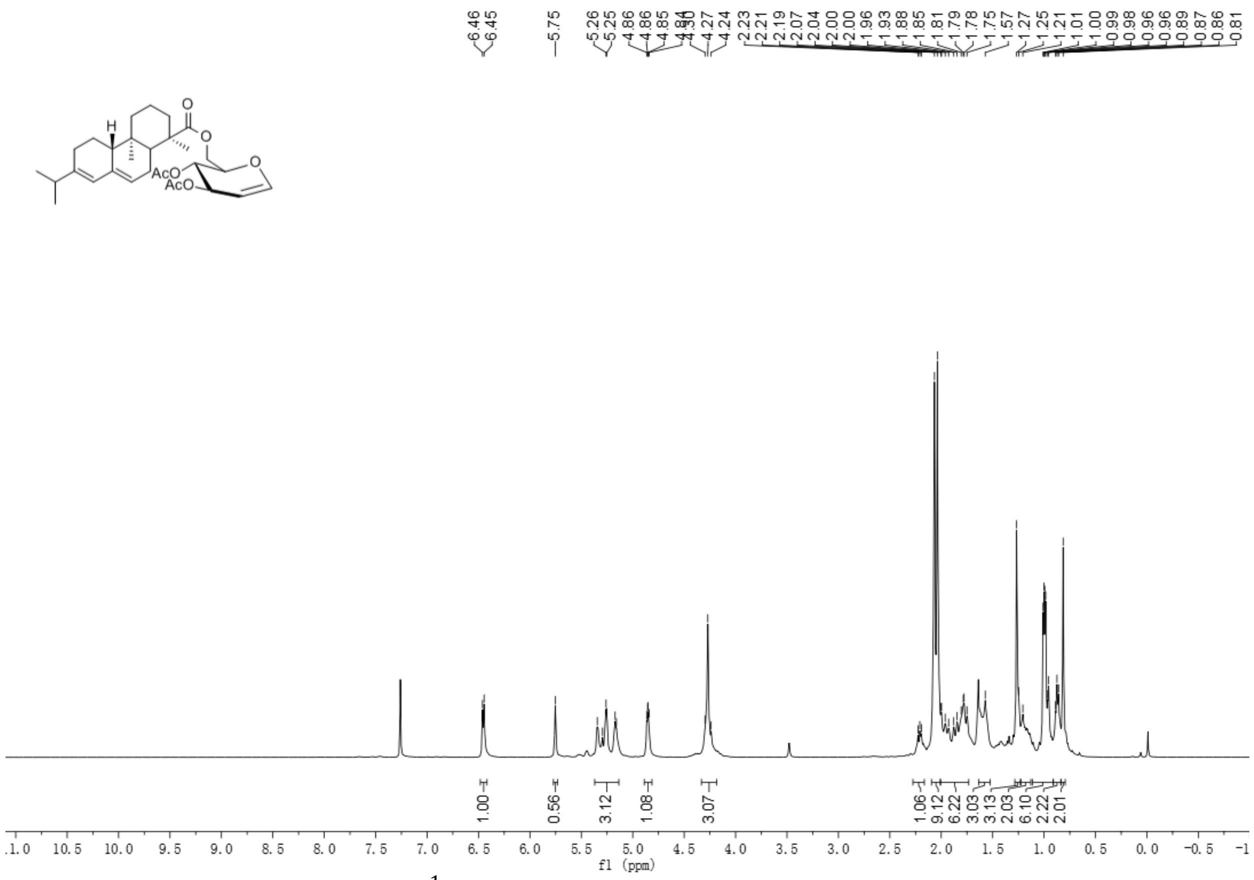

^{1}H NMR ($CDCl_3$-*d*) of **3af**

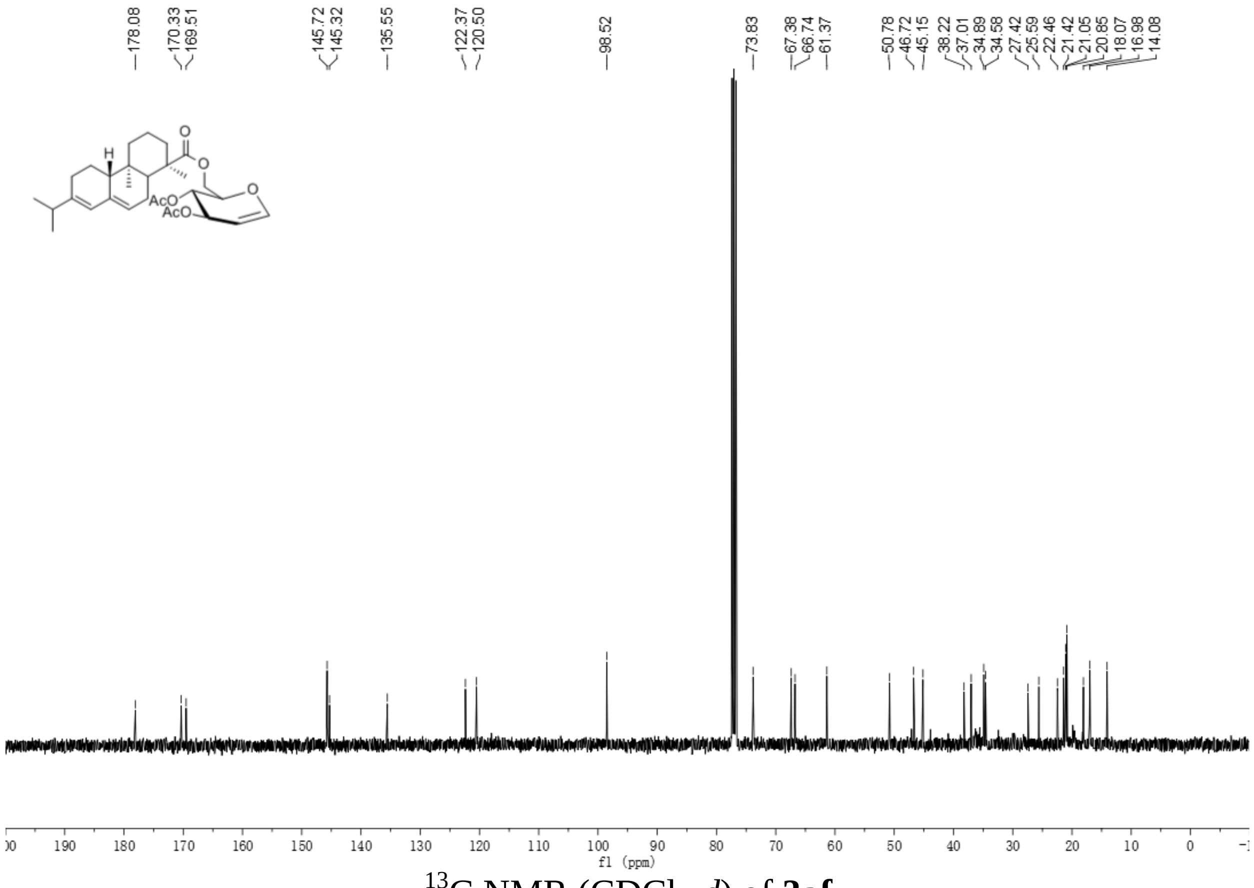

^{13}C NMR ($CDCl_3$-*d*) of **3af**

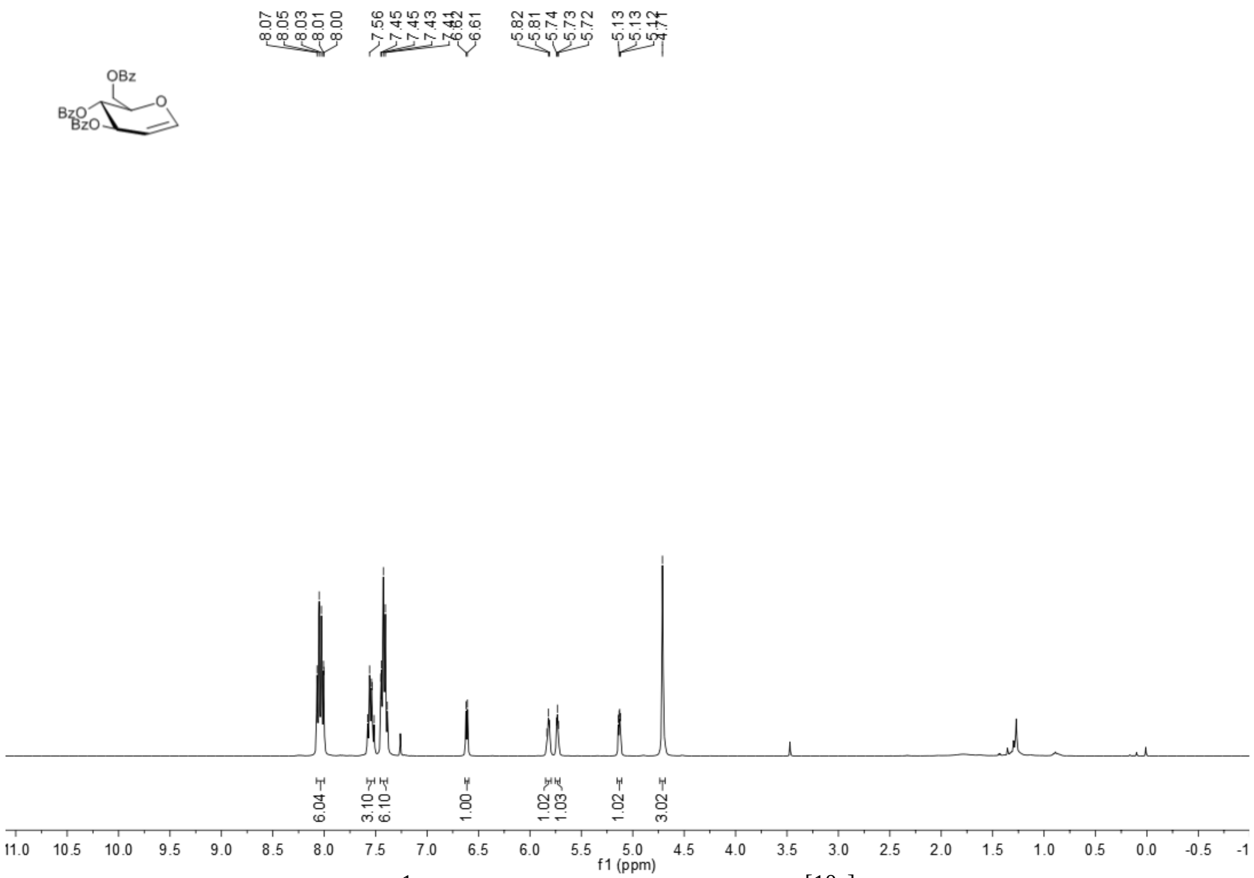

^{1}H NMR ($CDCl_3$-*d*) of **3ag**[10c]

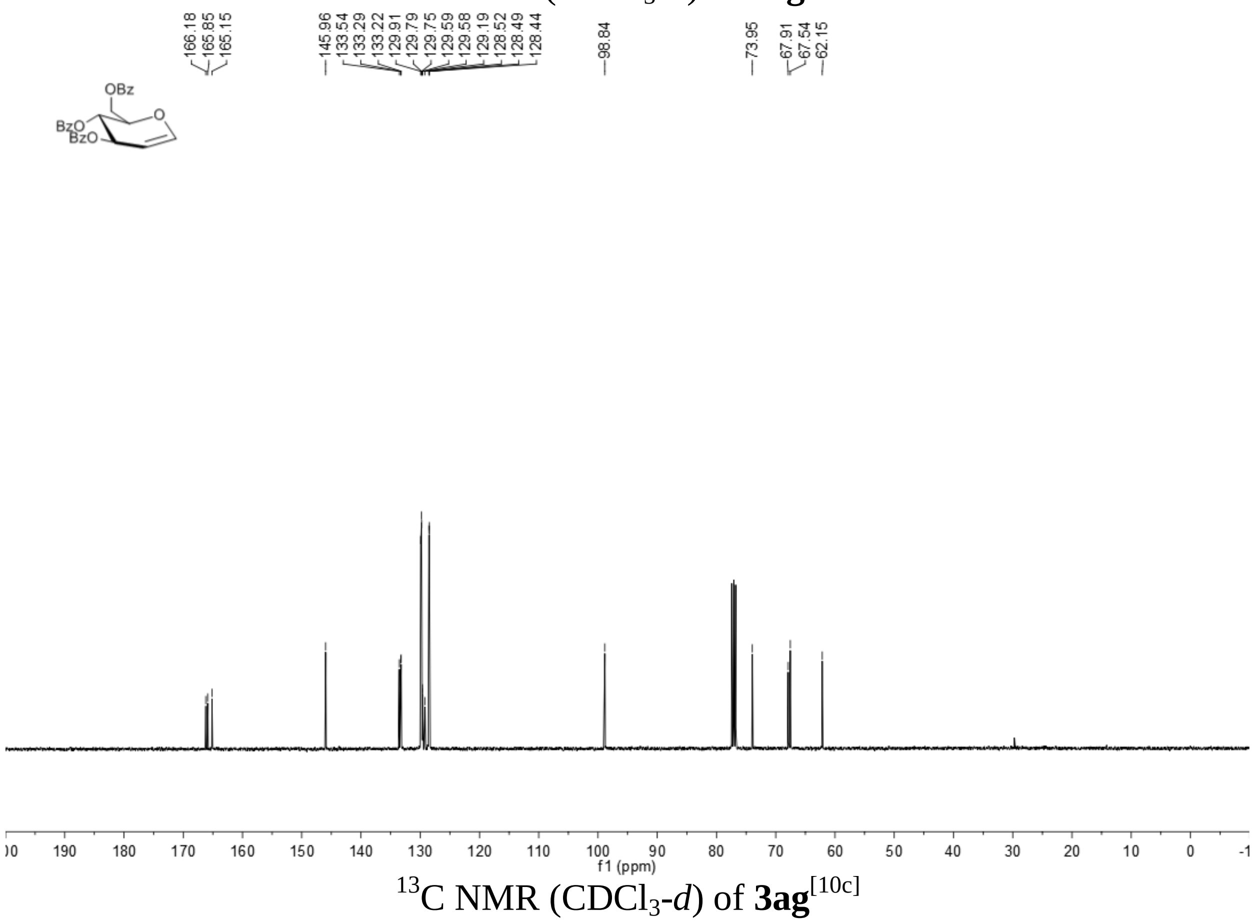

^{13}C NMR ($CDCl_3$-*d*) of **3ag**[10c]

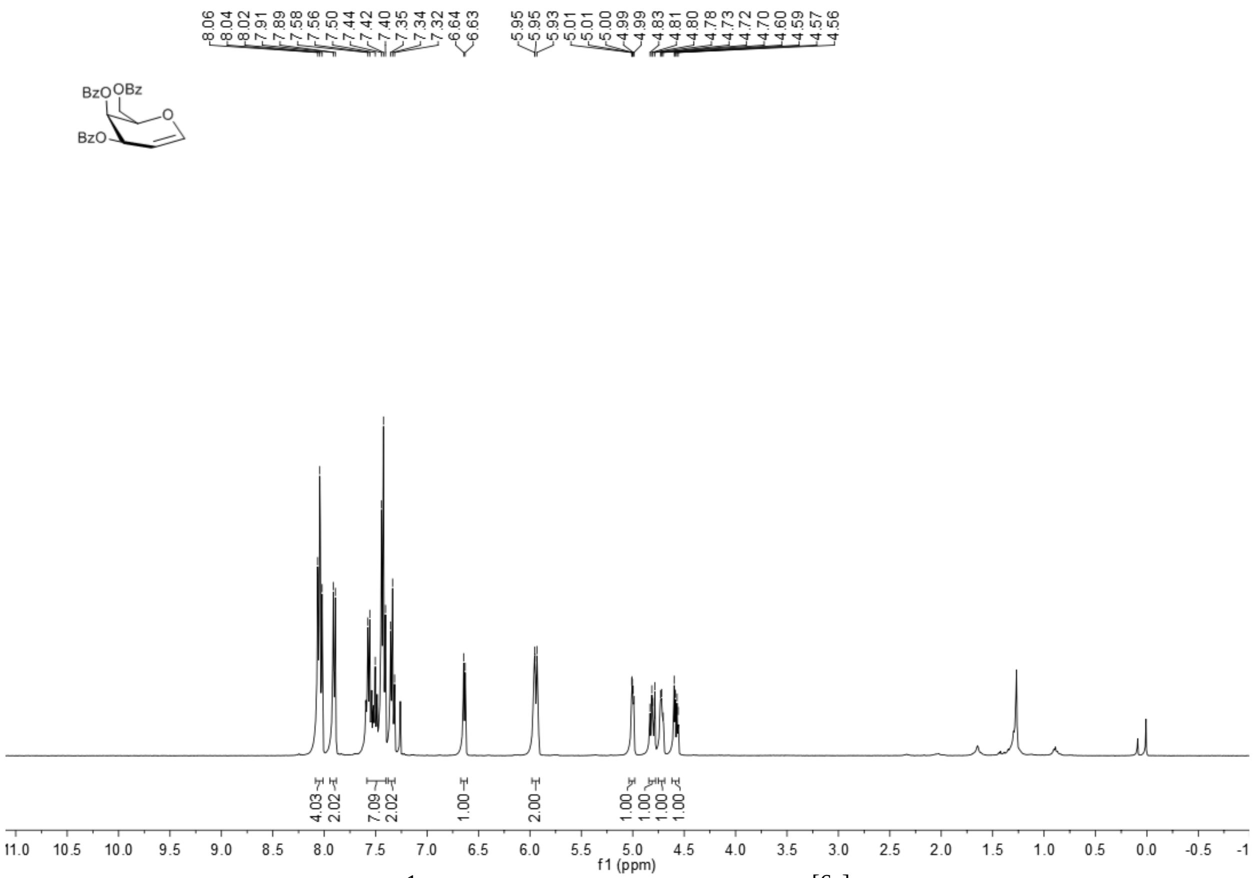

^{1}H NMR ($CDCl_3$-*d*) of **3ah**[6a]

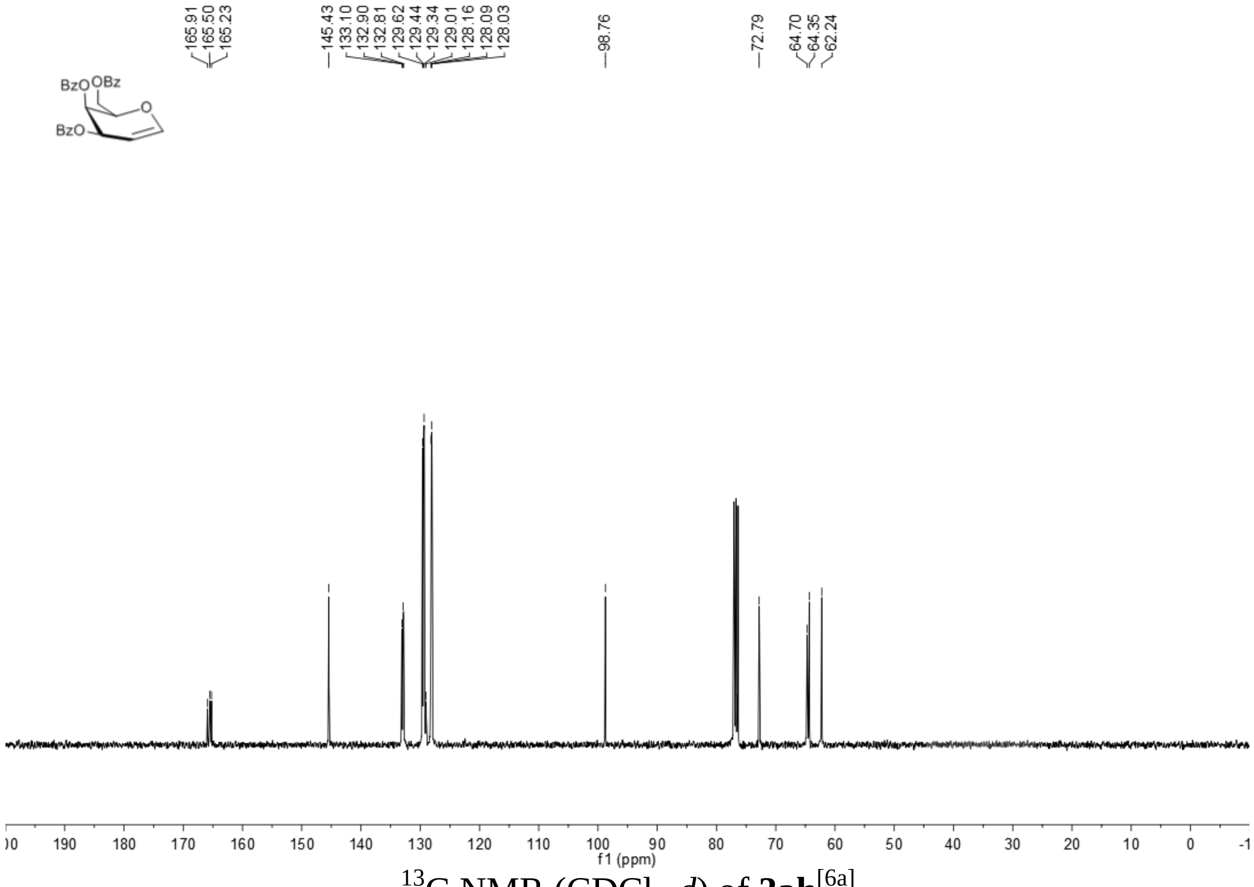

^{13}C NMR ($CDCl_3$-*d*) of **3ah**[6a]

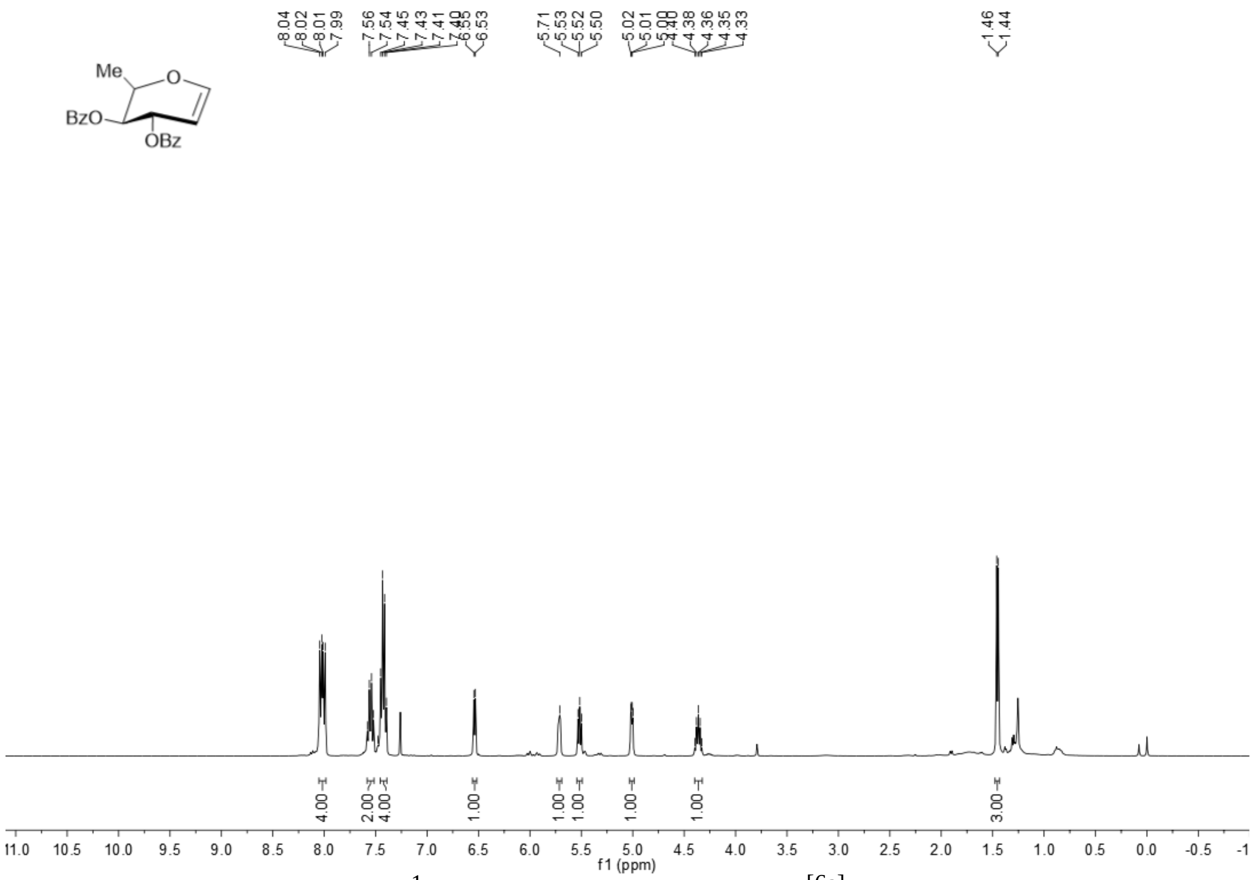

^{1}H NMR ($CDCl_3$-*d*) of **3ai**[6a]

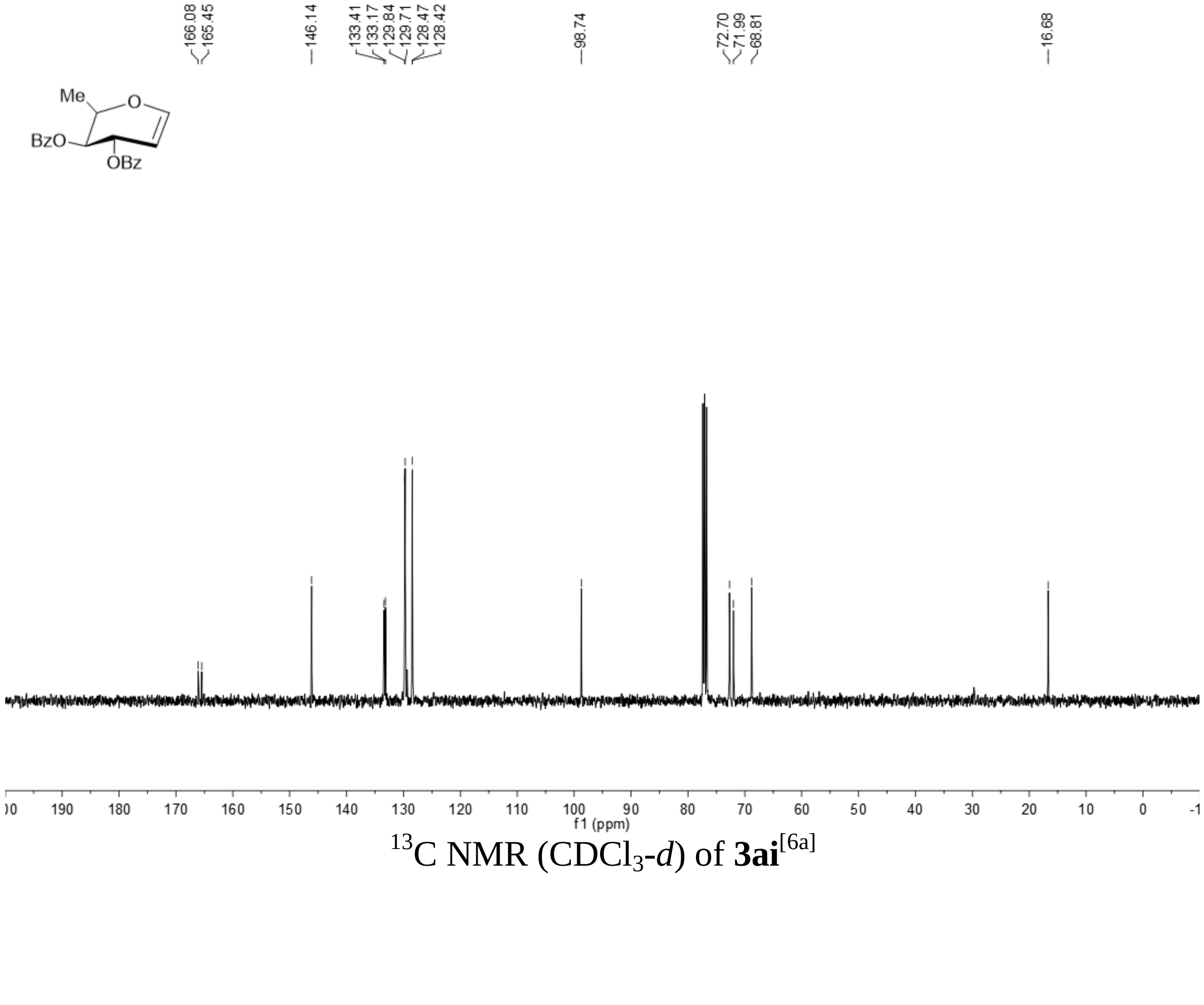

^{13}C NMR ($CDCl_3$-*d*) of **3ai**[6a]

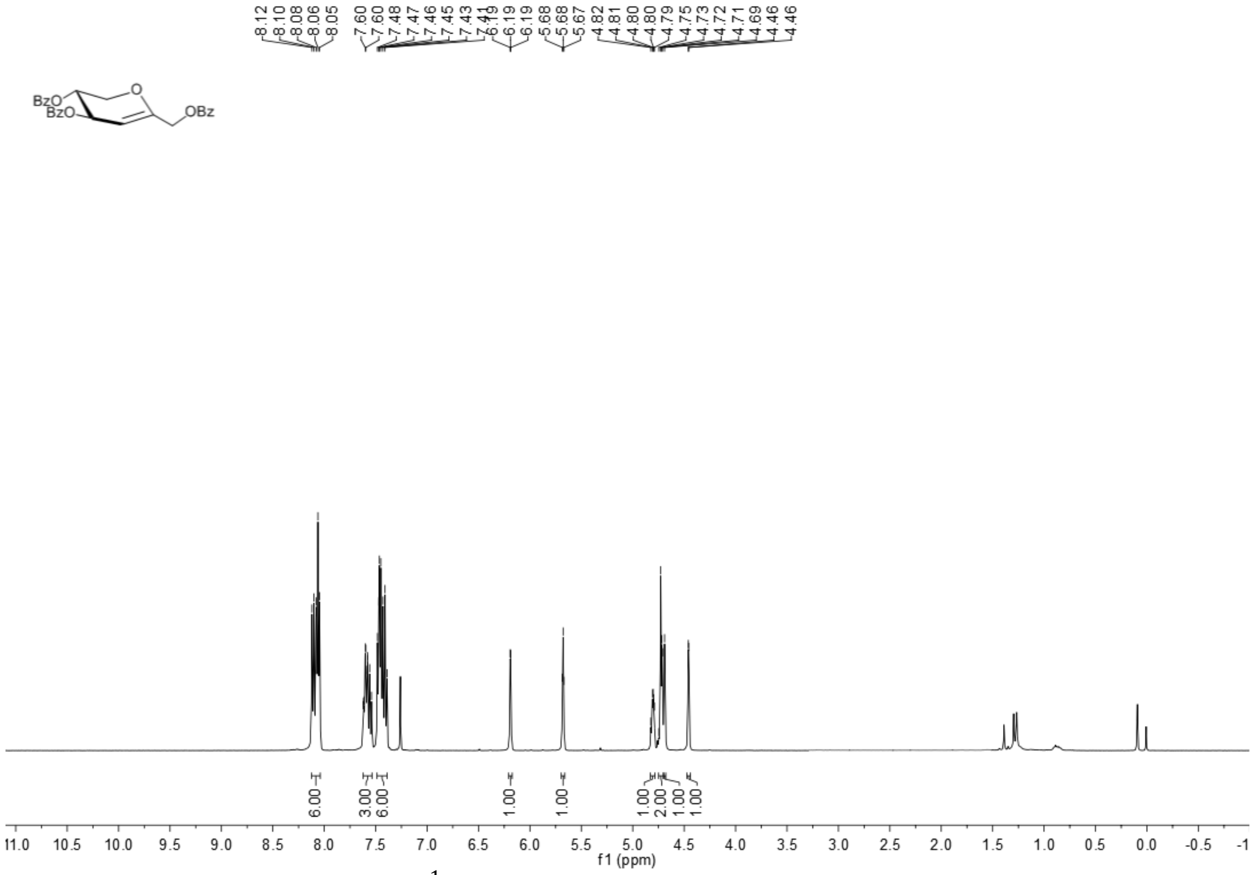

^{1}H NMR ($CDCl_3$-*d*) of **3aj**

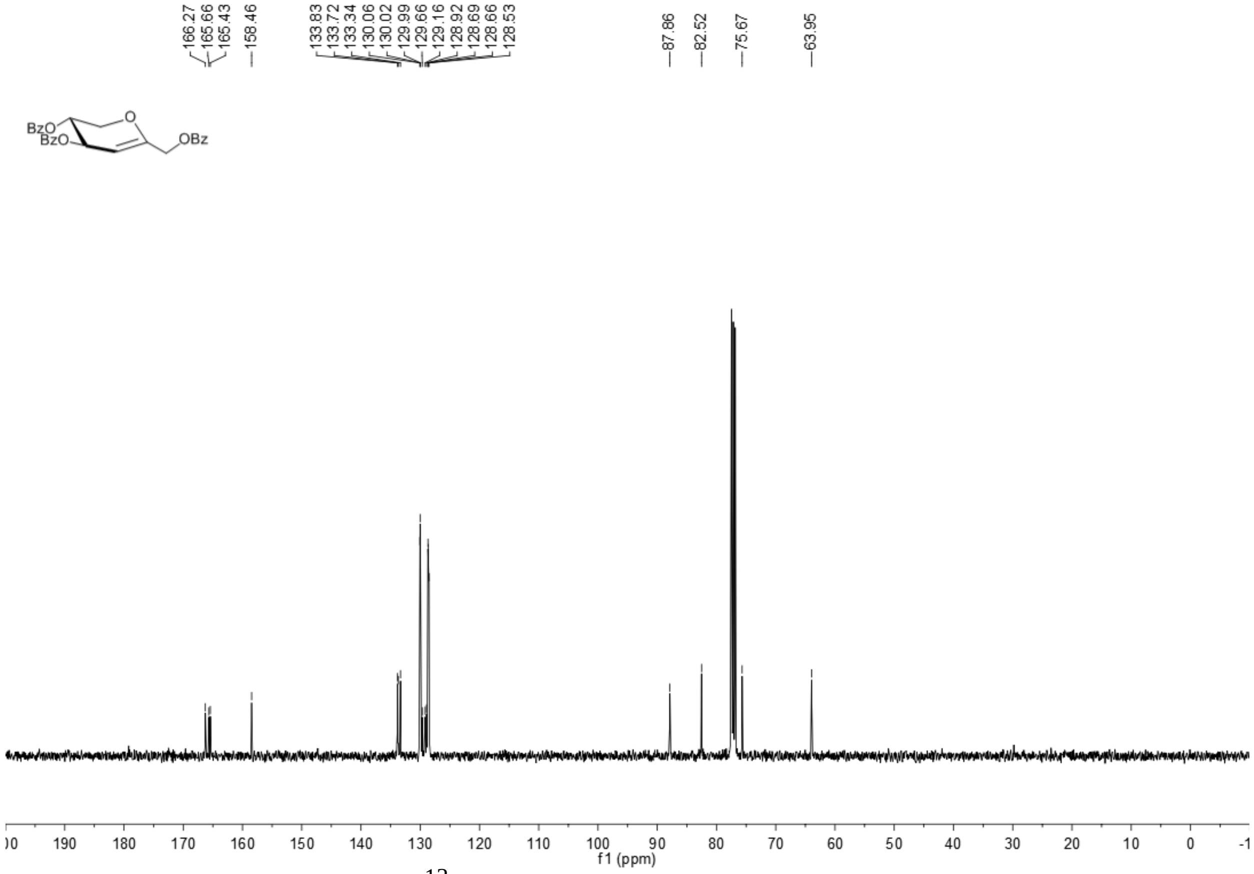

^{13}C NMR ($CDCl_3$-*d*) of **3aj**

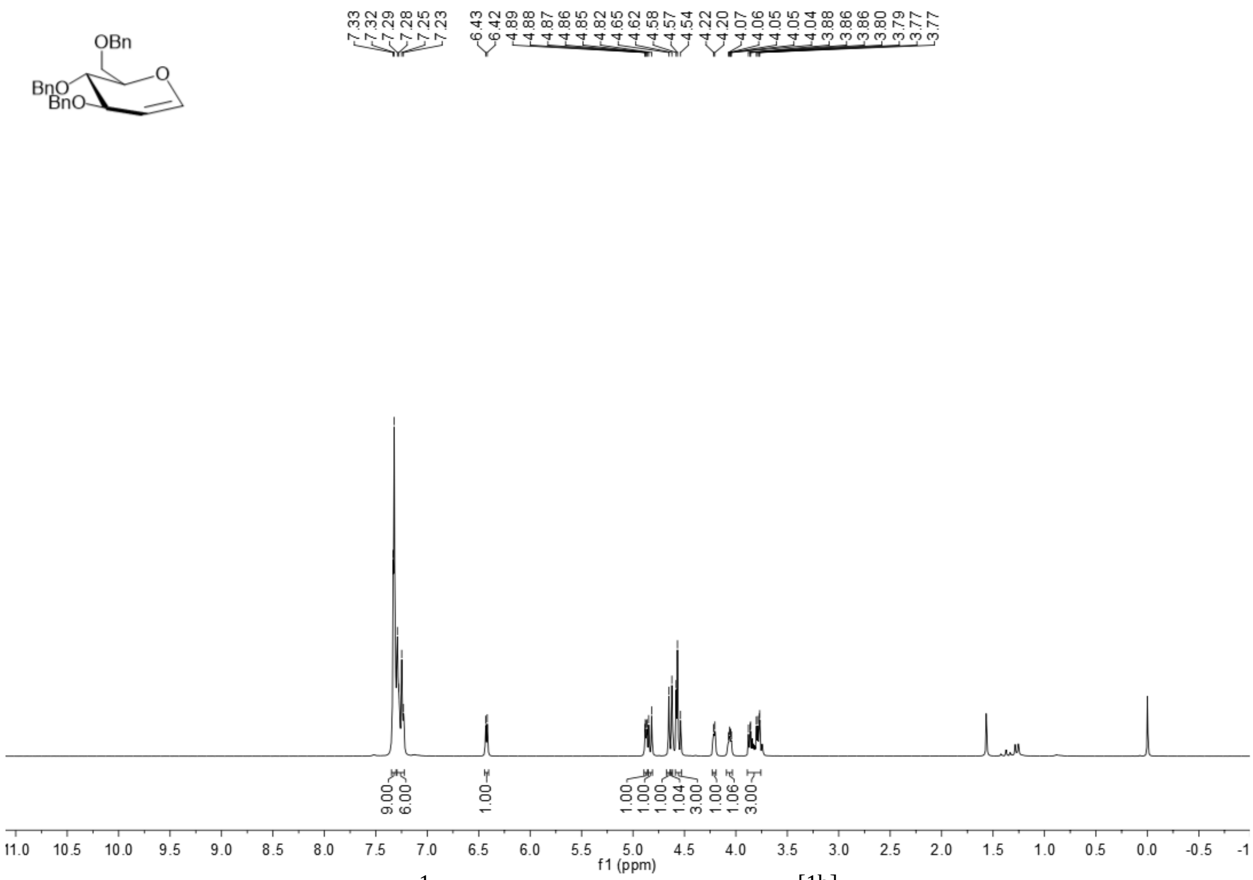

^{1}H NMR ($CDCl_3$-*d*) of **3f**[1b]

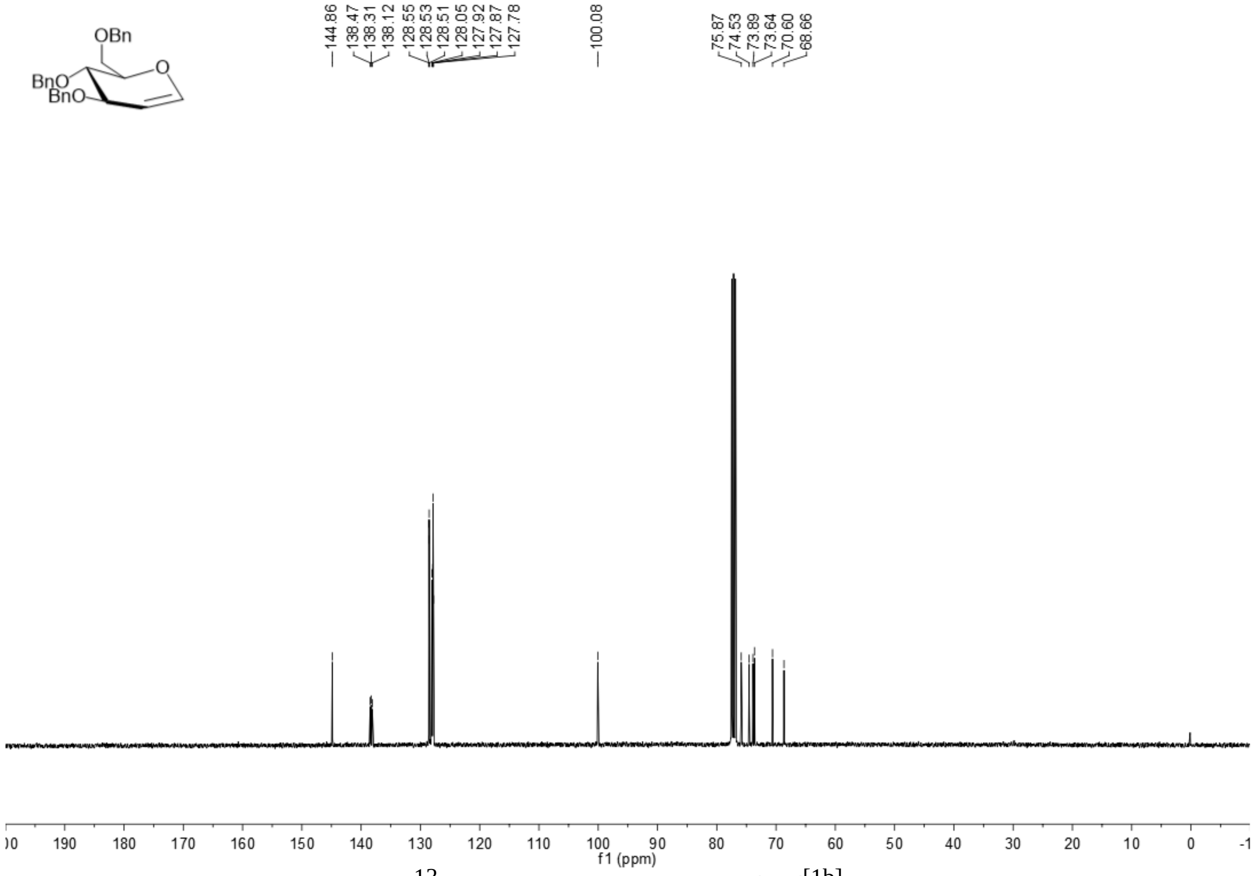

^{13}C NMR ($CDCl_3$-*d*) of **3f**[1b]

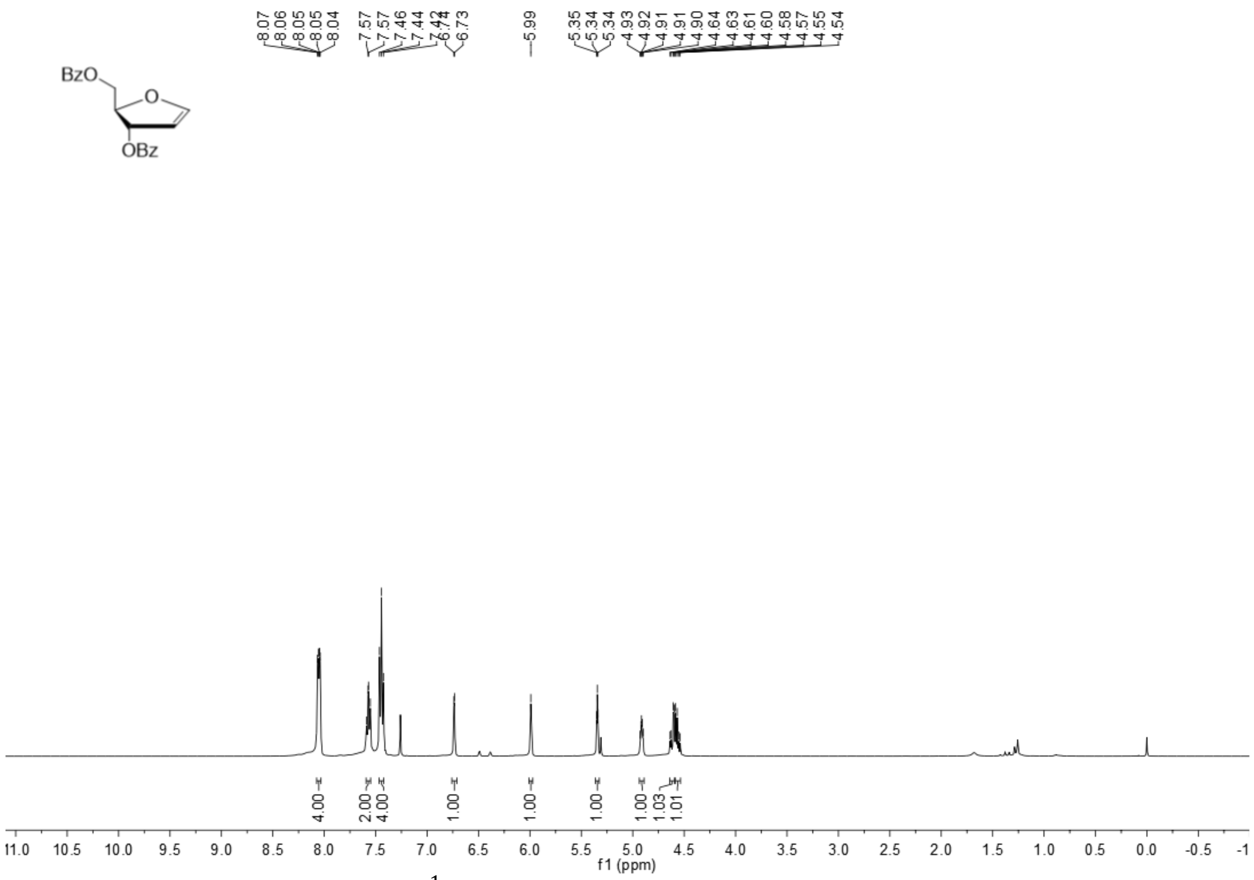

^{1}H NMR ($CDCl_3$-*d*) of **3m**

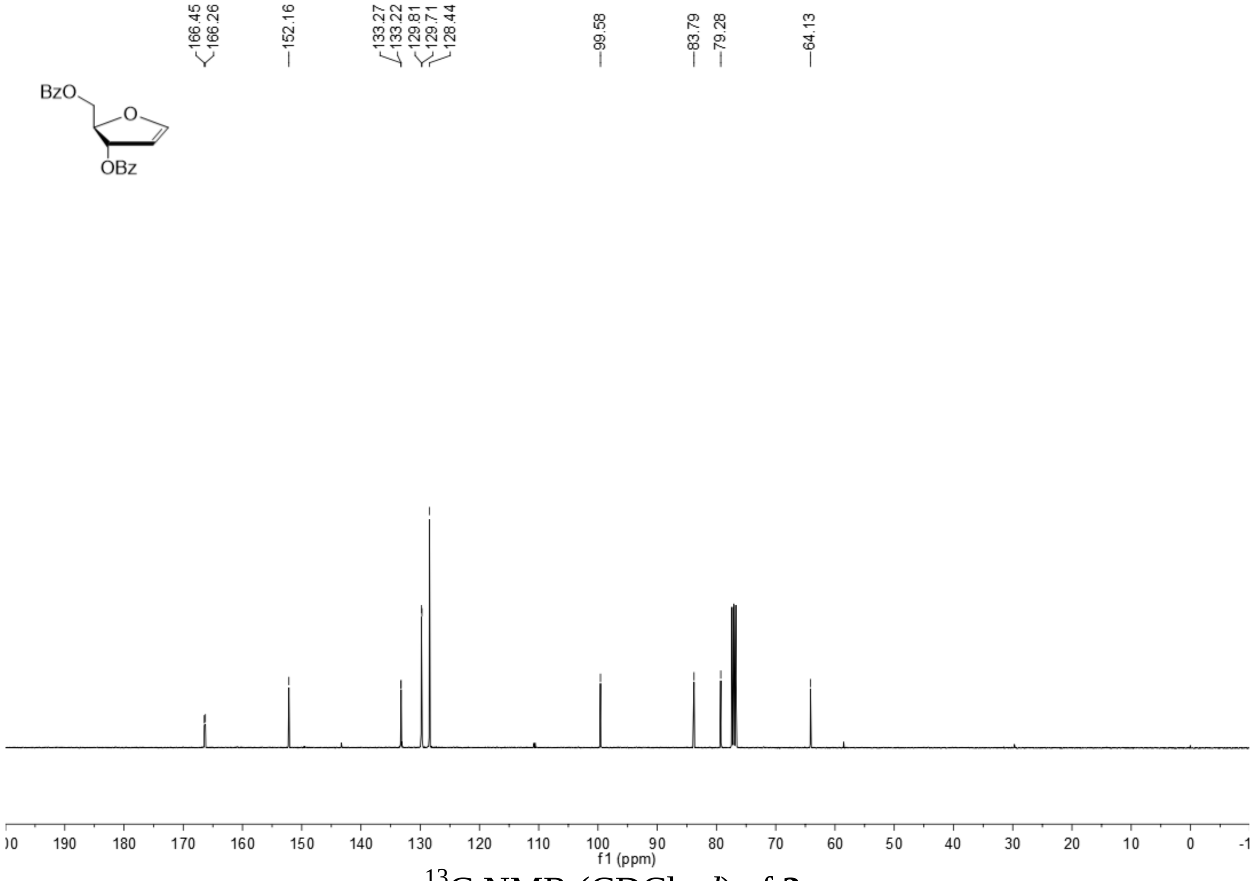

^{13}C NMR ($CDCl_3$-*d*) of **3m**

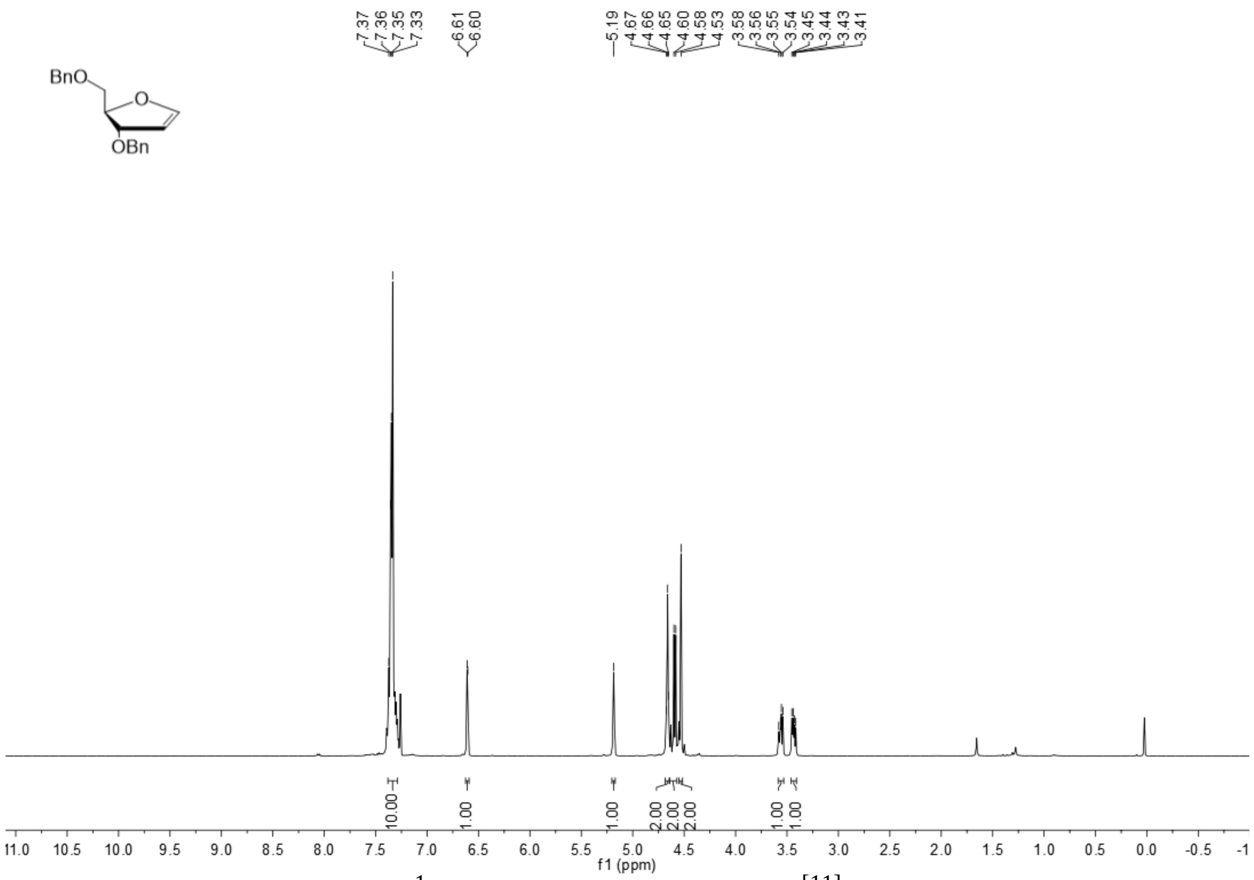

^{1}H NMR ($CDCl_3$-*d*) of **3n**[11]

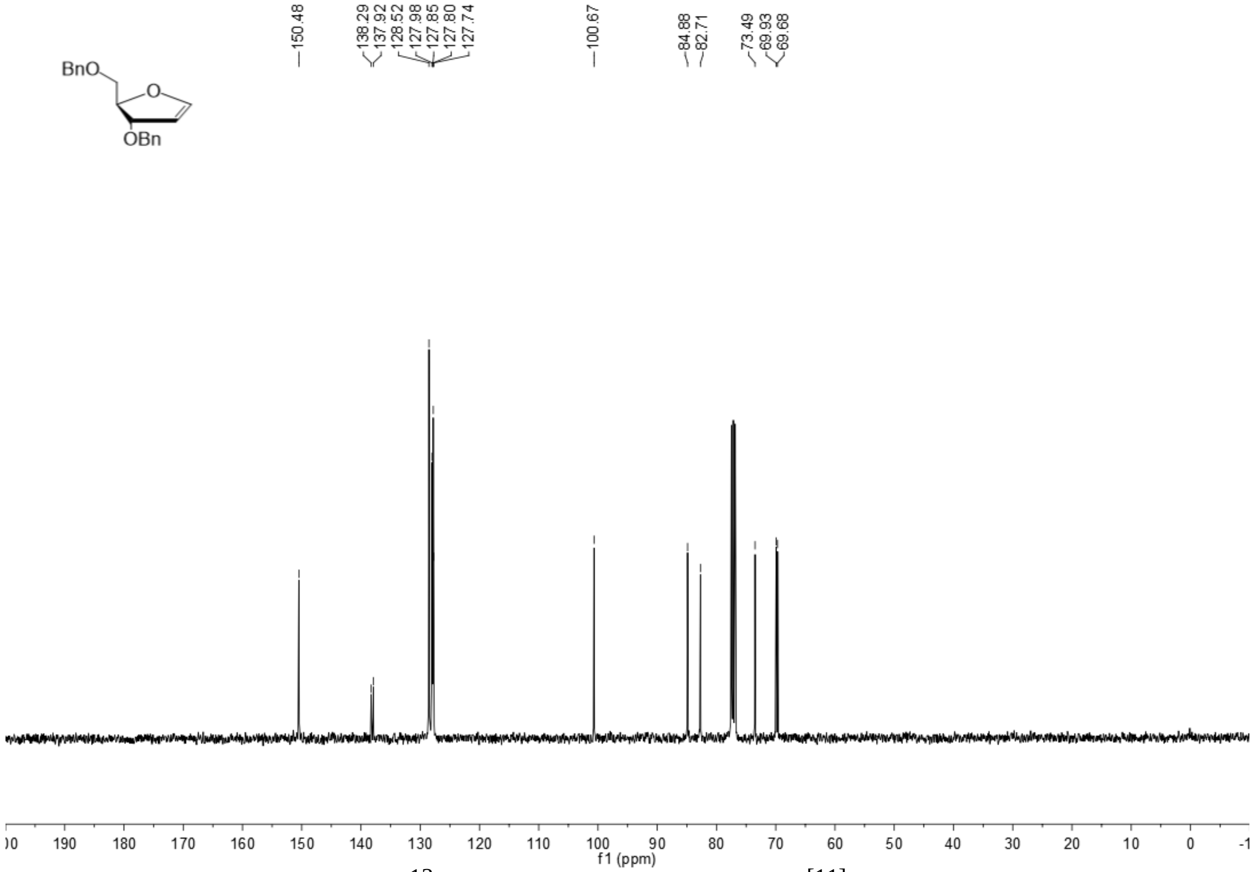

^{13}C NMR ($CDCl_3$-*d*) of **3n**[11]

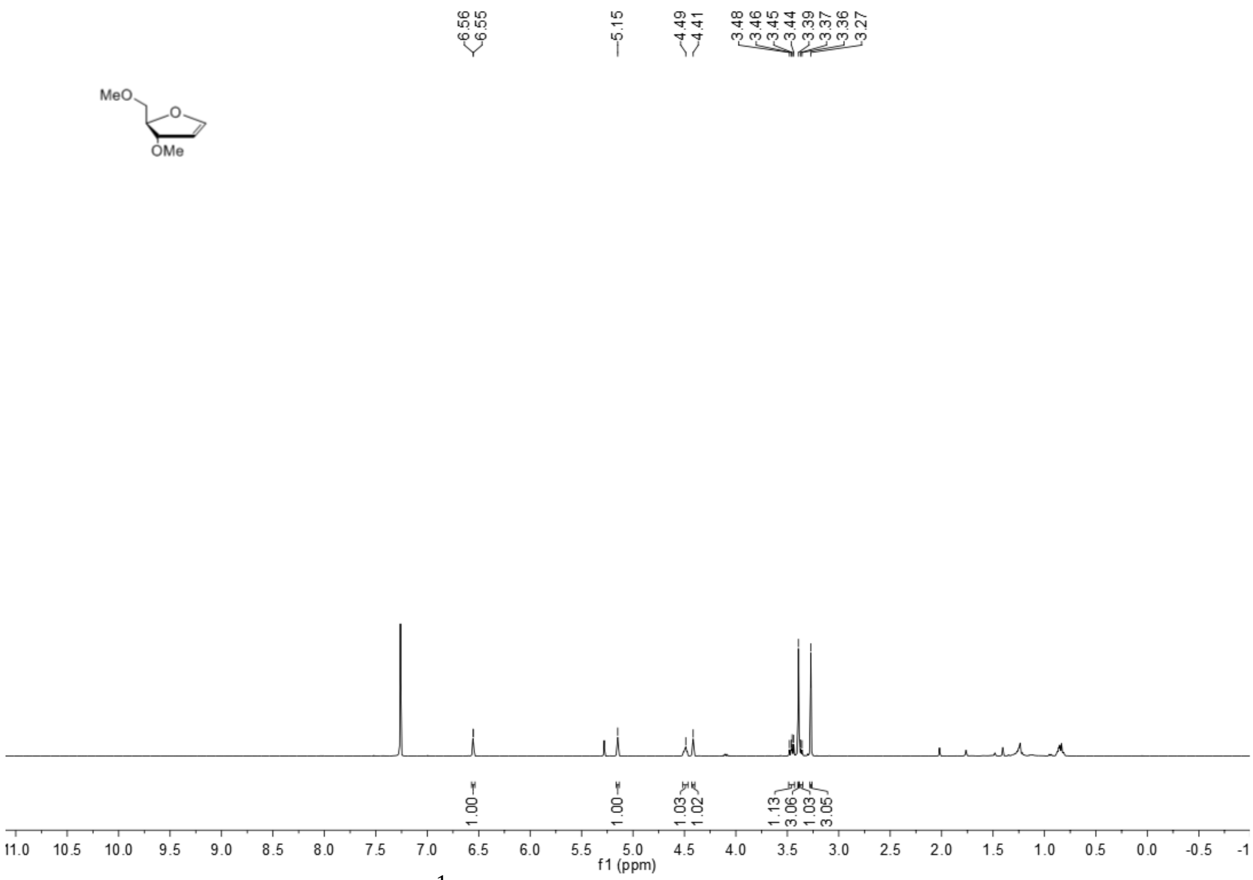

^{1}H NMR (CDCl$_3$-*d*) of **3o**

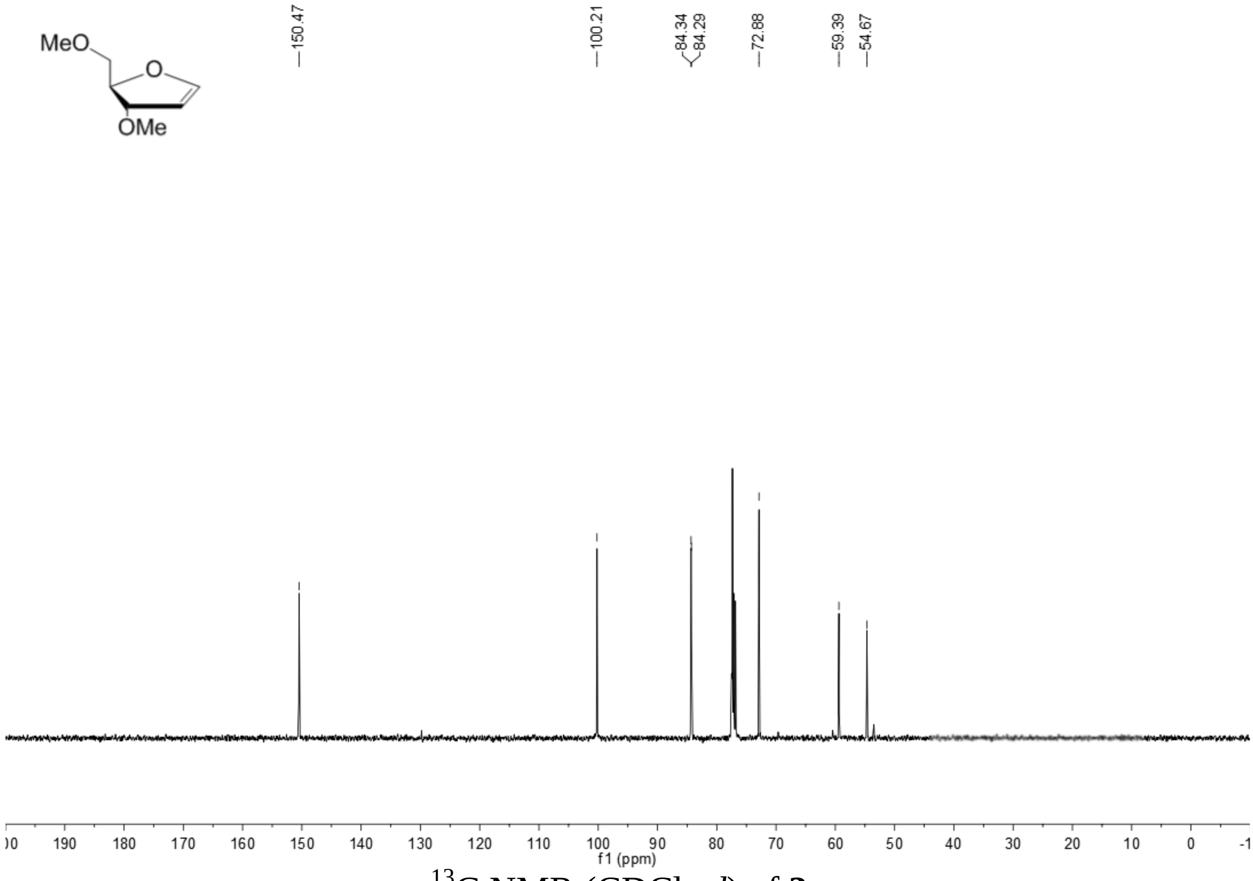

^{13}C NMR (CDCl$_3$-*d*) of **3o**

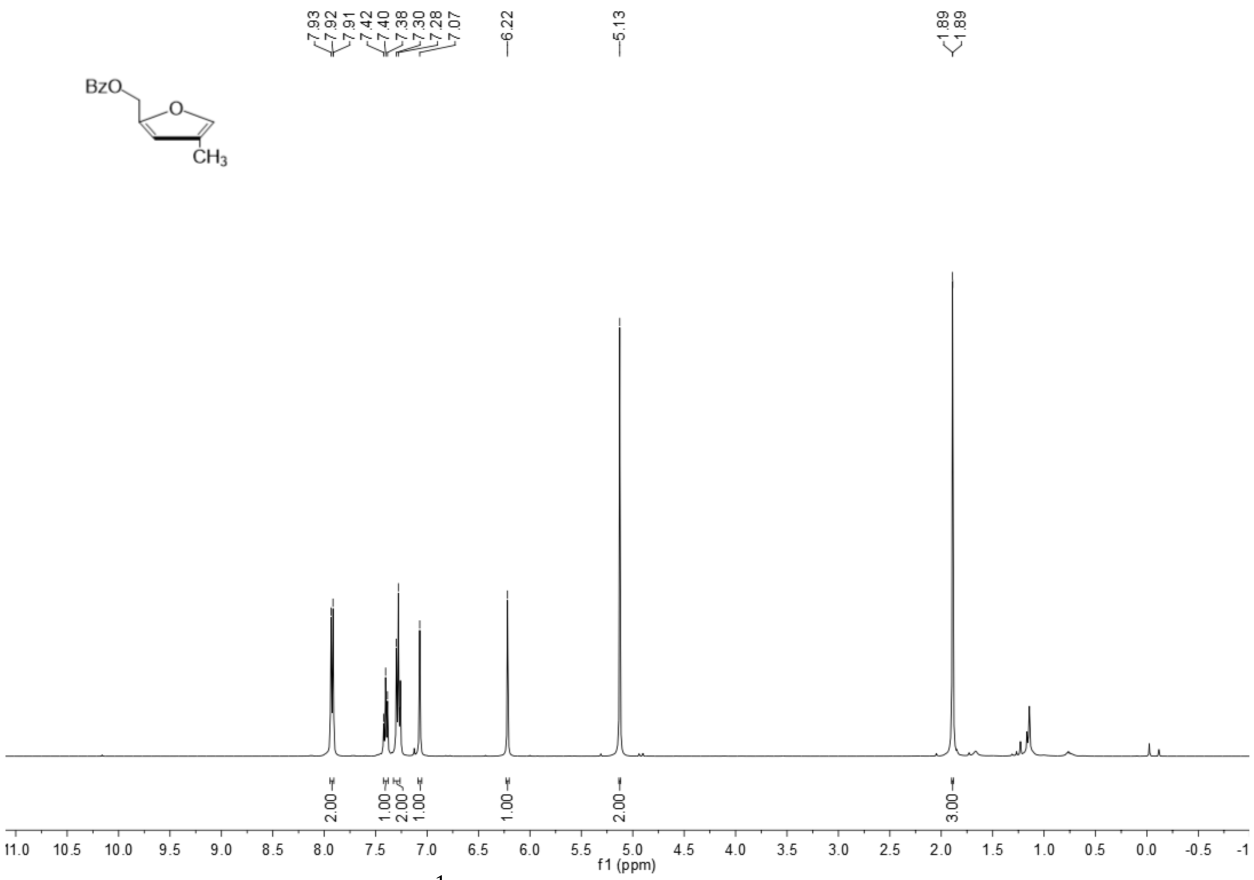

^{1}H NMR ($CDCl_3$-*d*) of **4p**

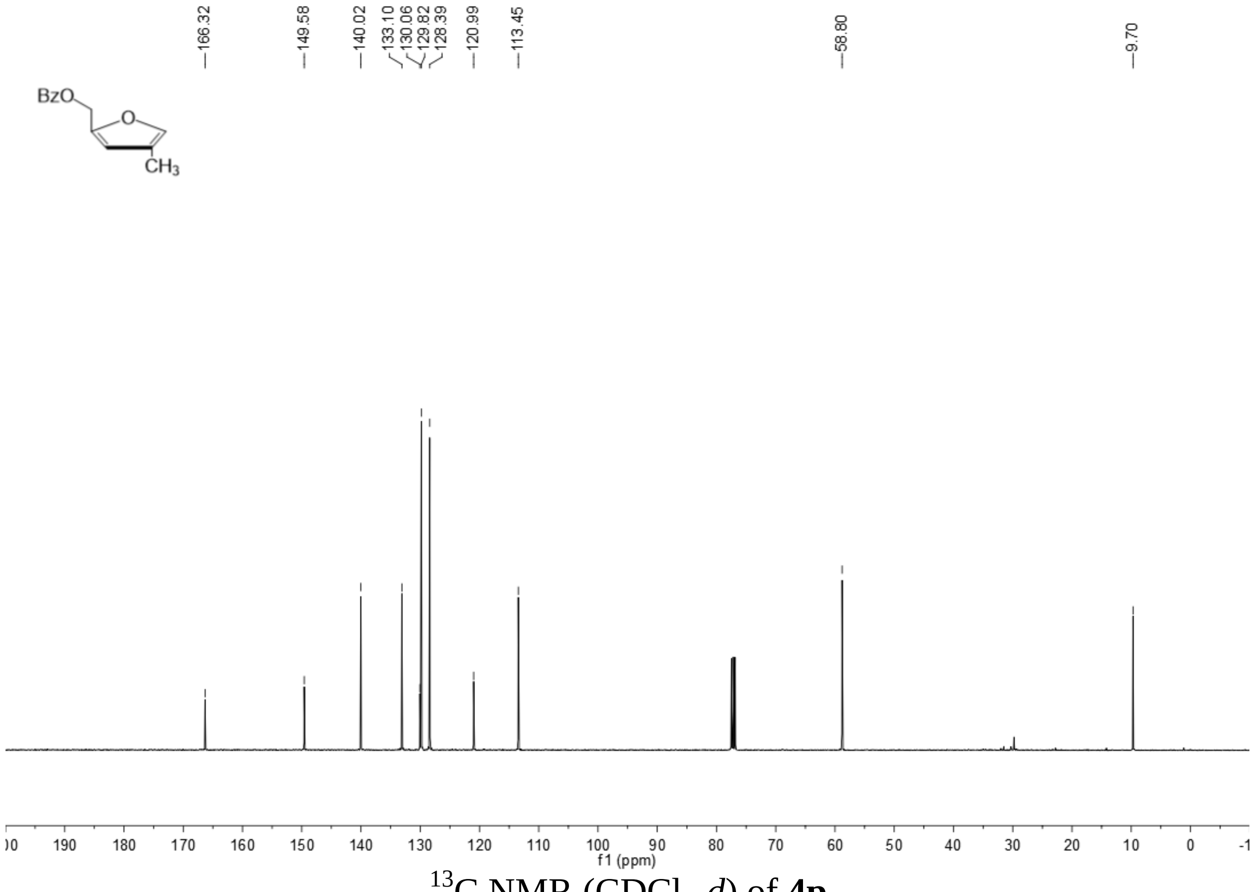

^{13}C NMR ($CDCl_3$-*d*) of **4p**

14. Copies of ^{1}H NMR, ^{13}C NMR Spectra of Compounds 1*D*-3.

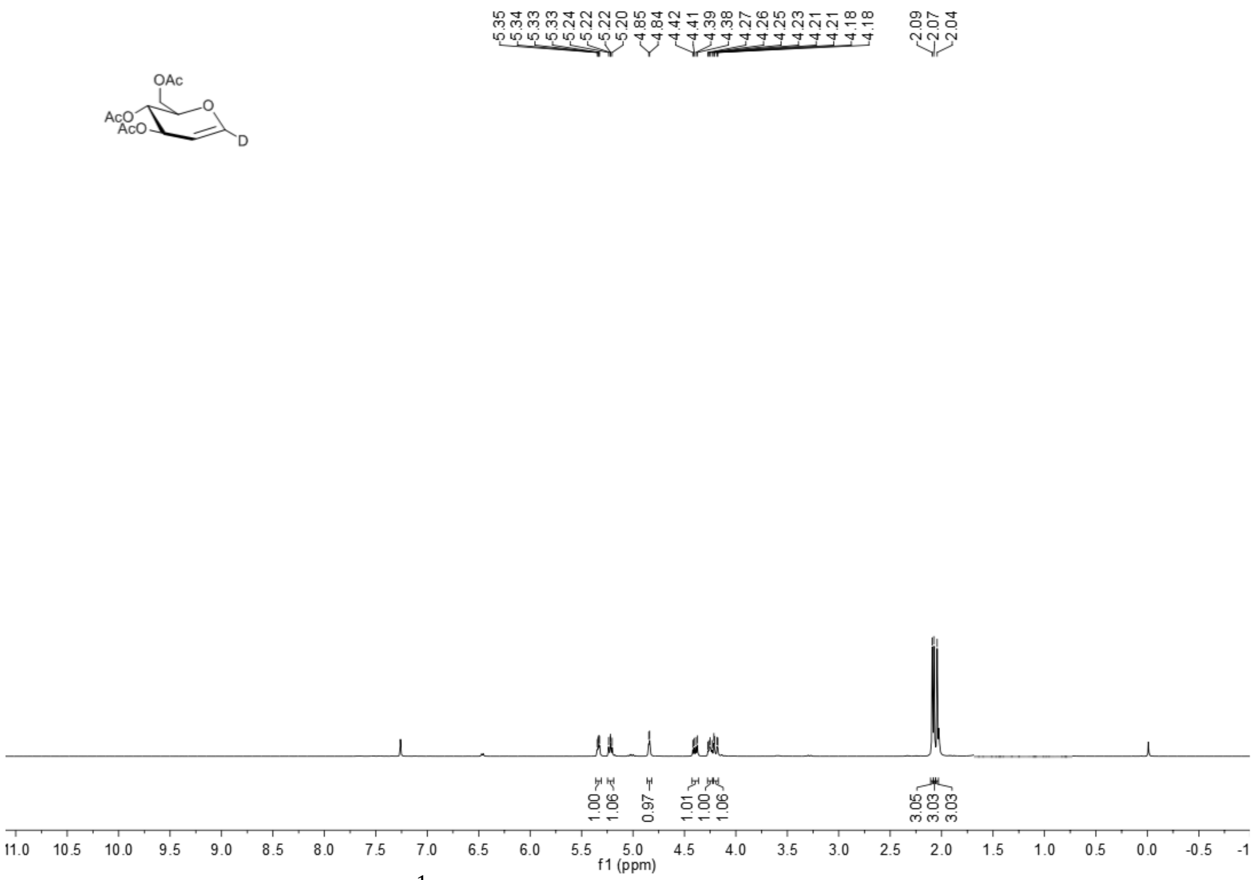

^{1}H NMR ($CDCl_3$-*d*) of ***D*-3a**

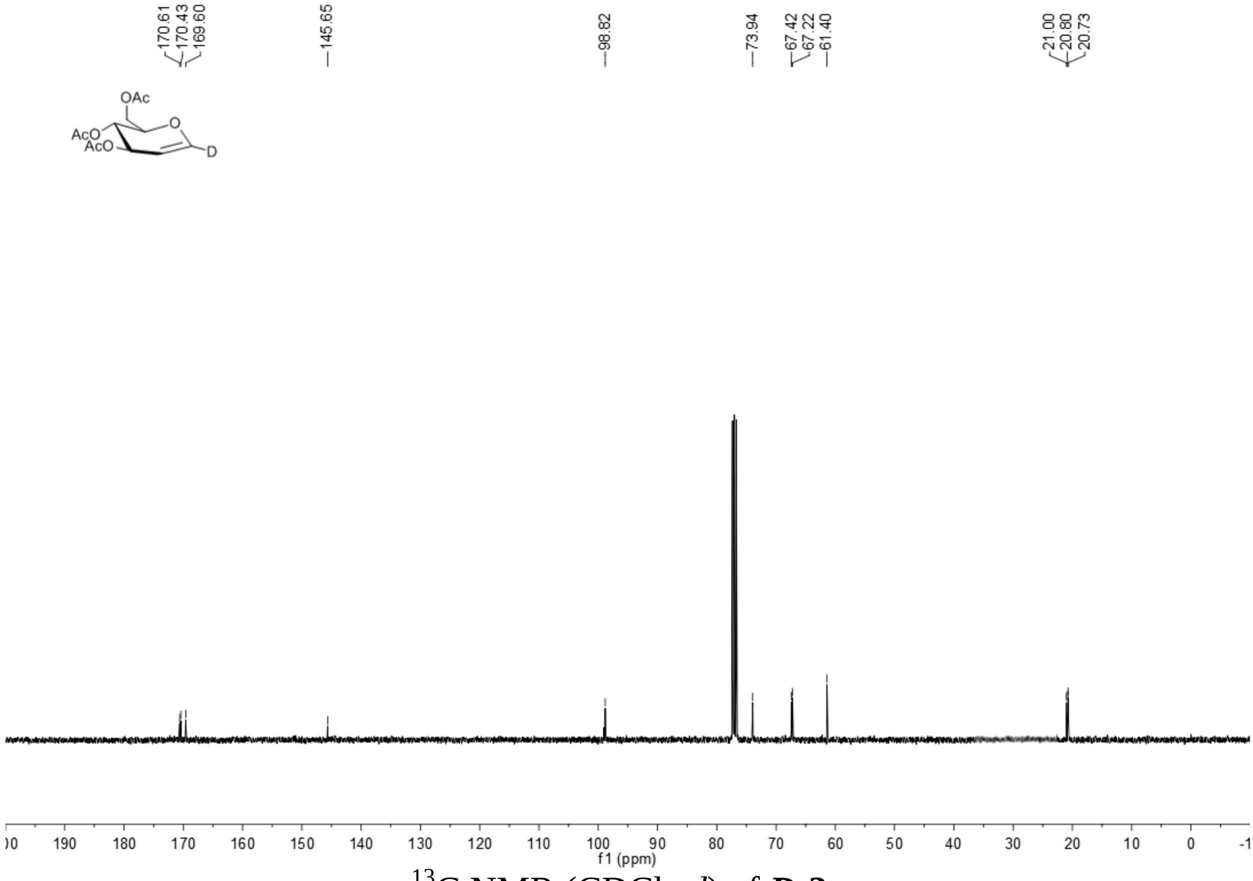

^{13}C NMR ($CDCl_3$-*d*) of ***D*-3a**

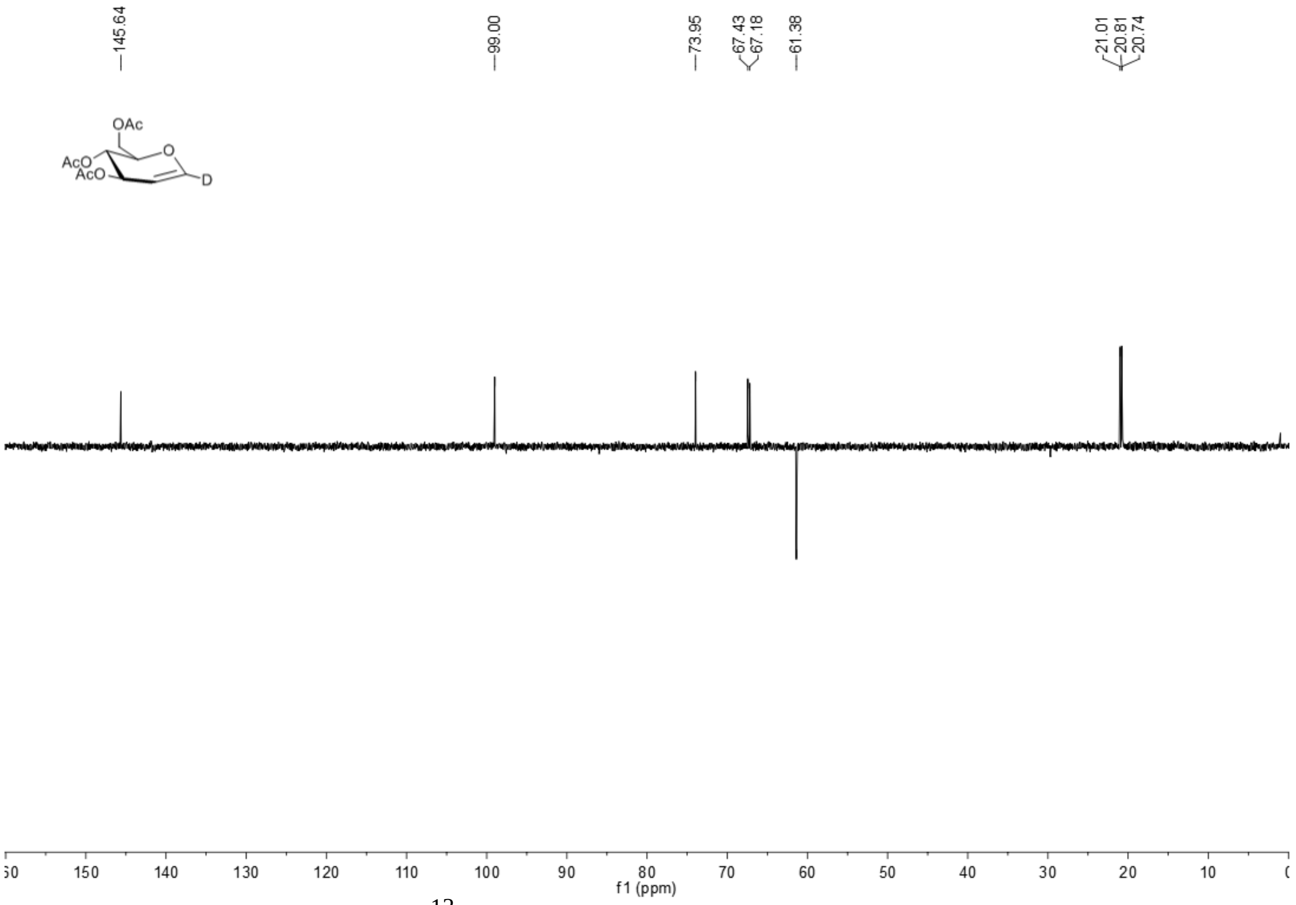

^{13}C NMR ($CDCl_3$-*d*) of ***D*-3a**

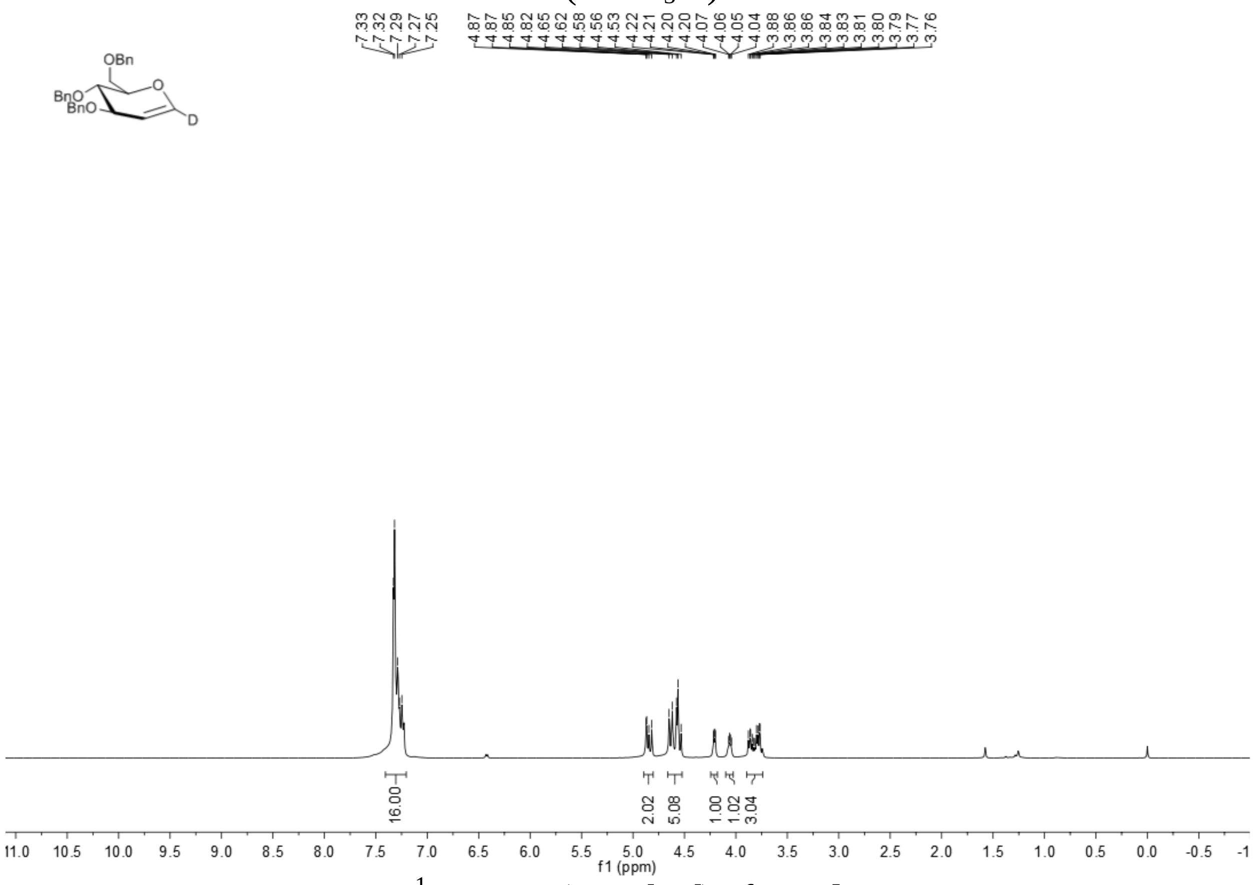

^{1}H NMR ($CDCl_3$-*d*) of ***D*-3d**

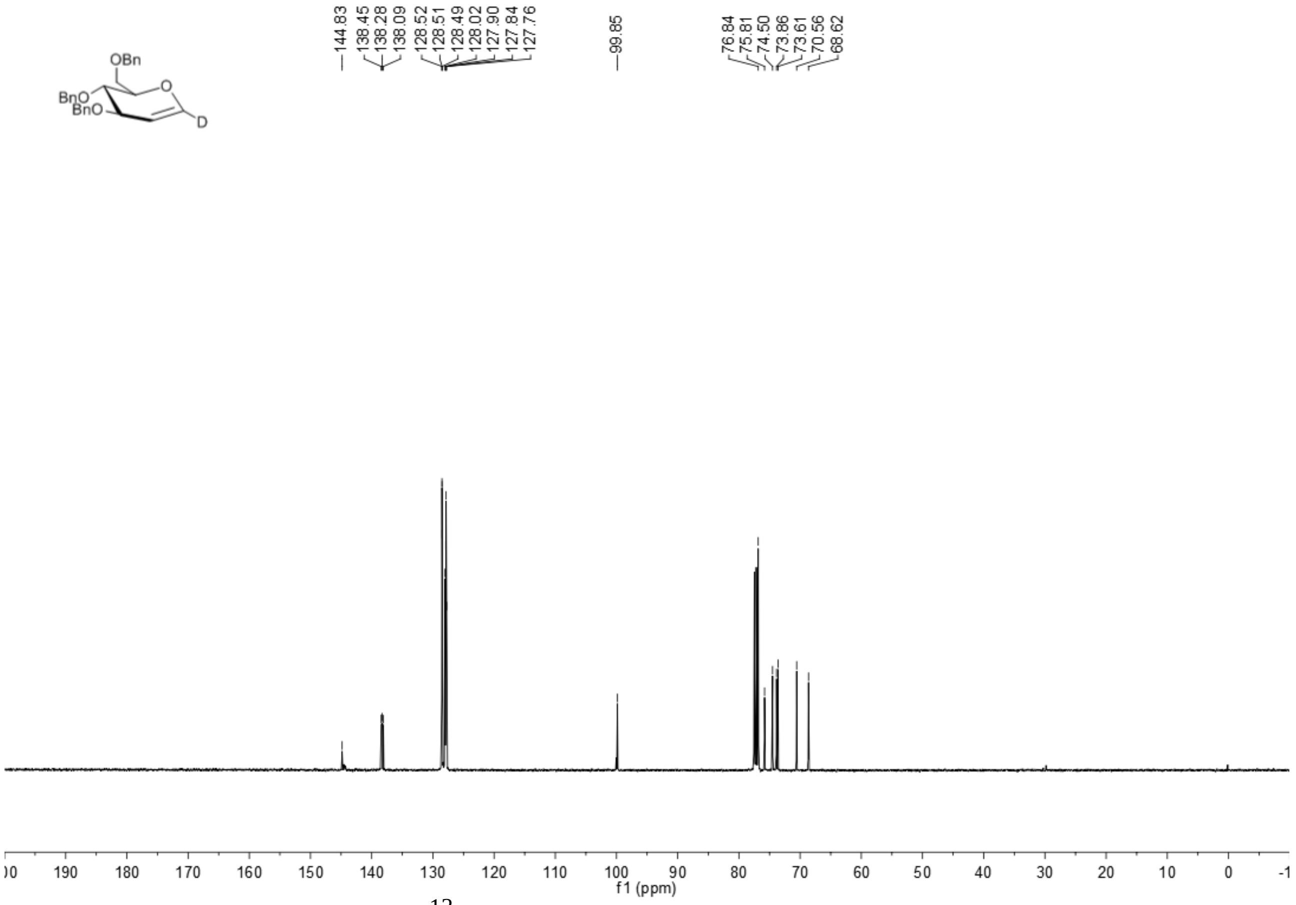

^{13}C NMR ($CDCl_3$-*d*) of ***D*-3d**

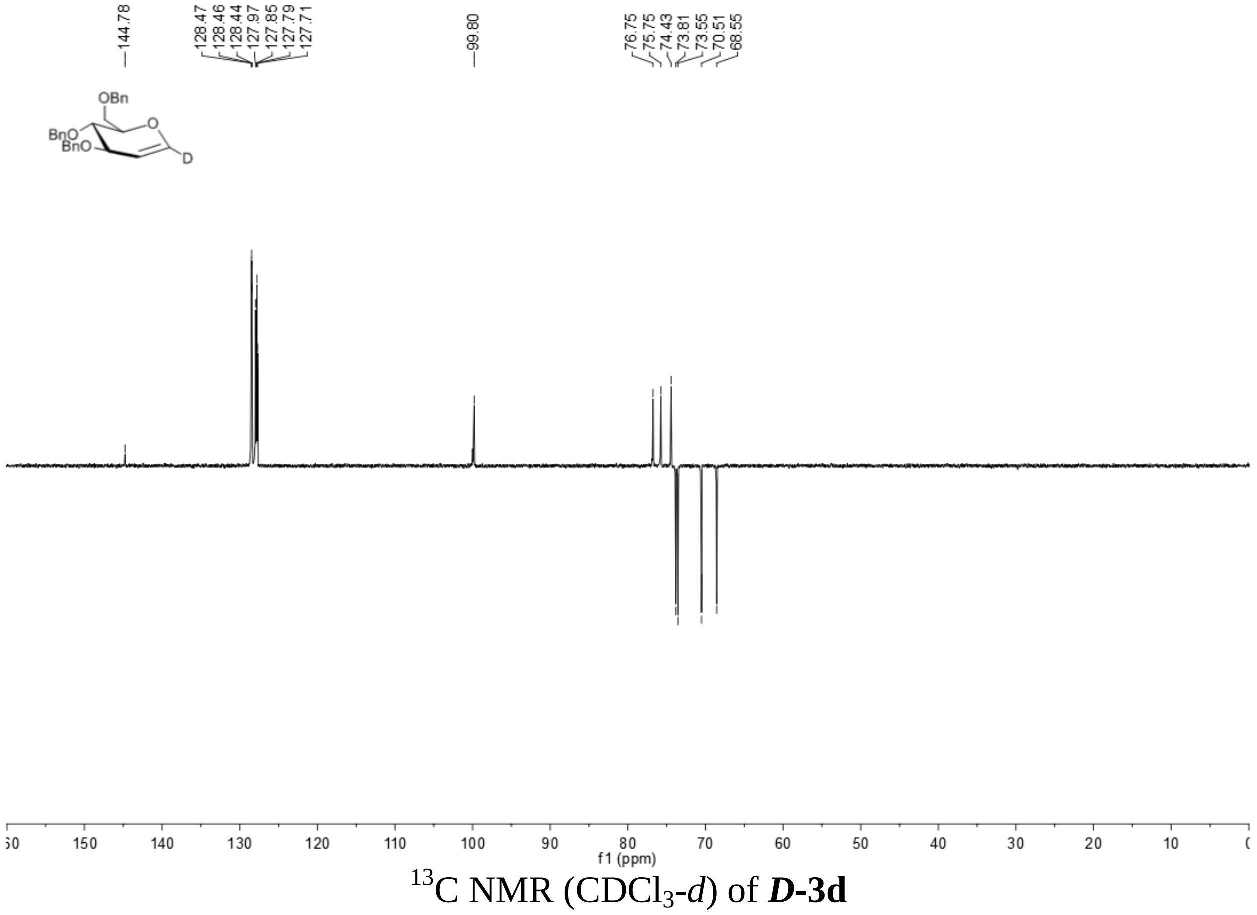

^{13}C NMR ($CDCl_3$-*d*) of ***D*-3d**

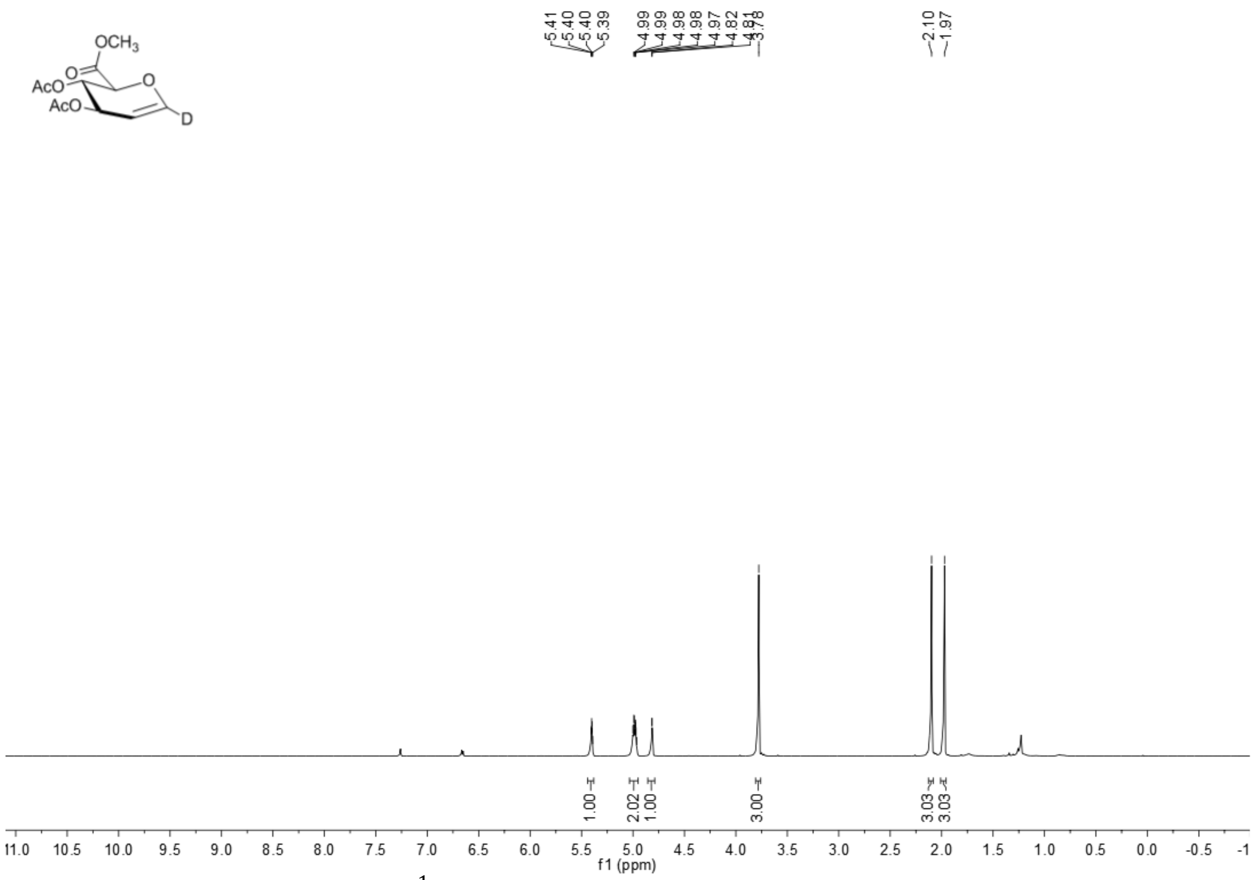

^{1}H NMR ($CDCl_3$-*d*) of ***D*-3e**

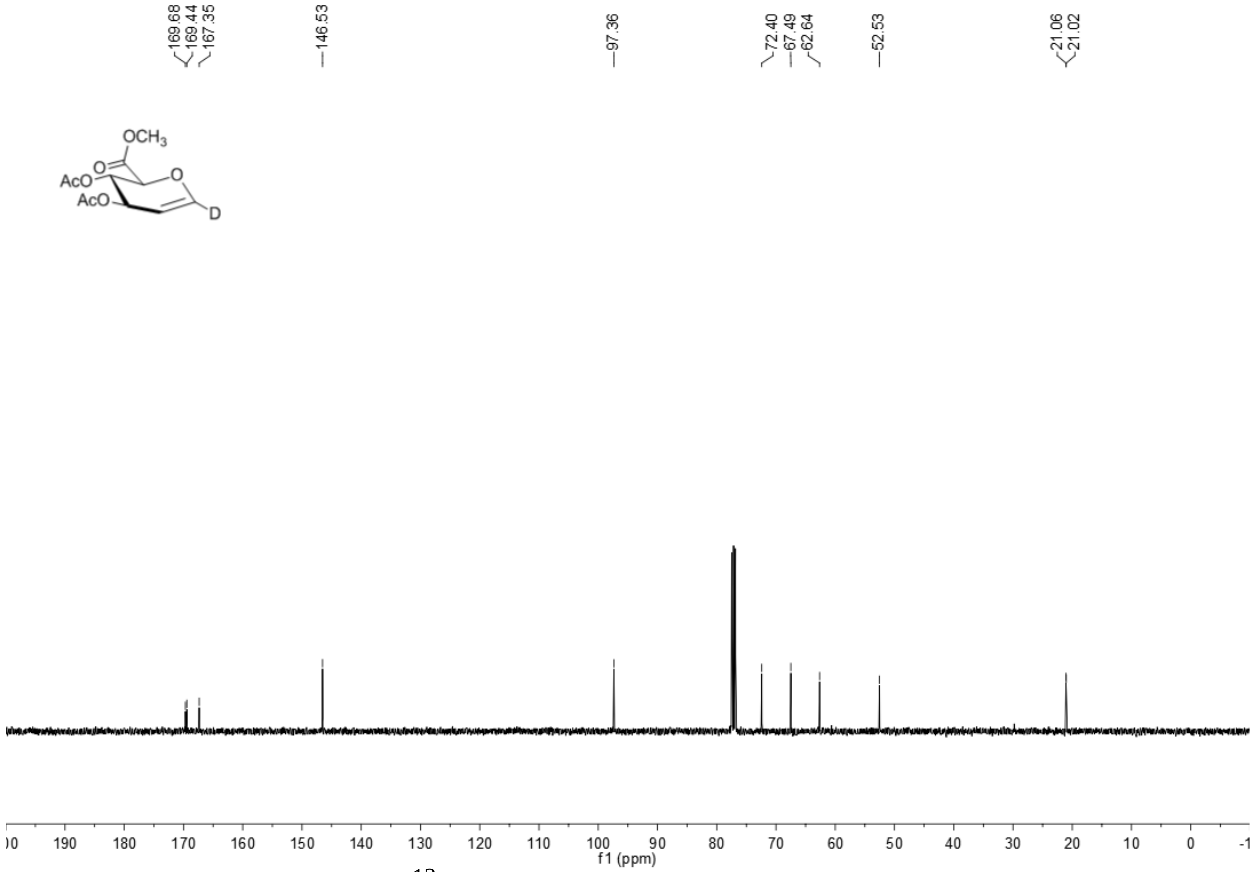

^{13}C NMR ($CDCl_3$-*d*) of ***D*-3e**

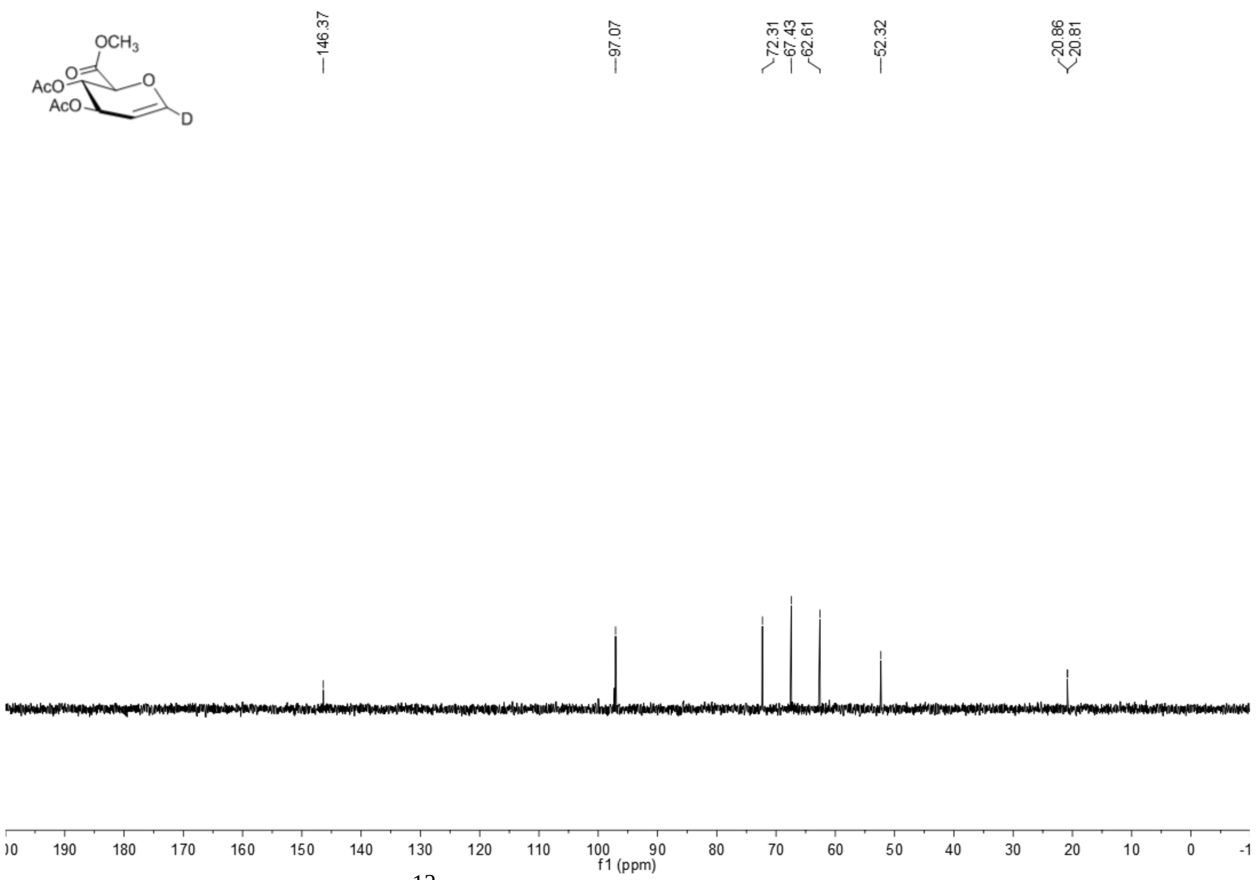

^{13}C NMR ($CDCl_3$-*d*) of ***D*-3e**

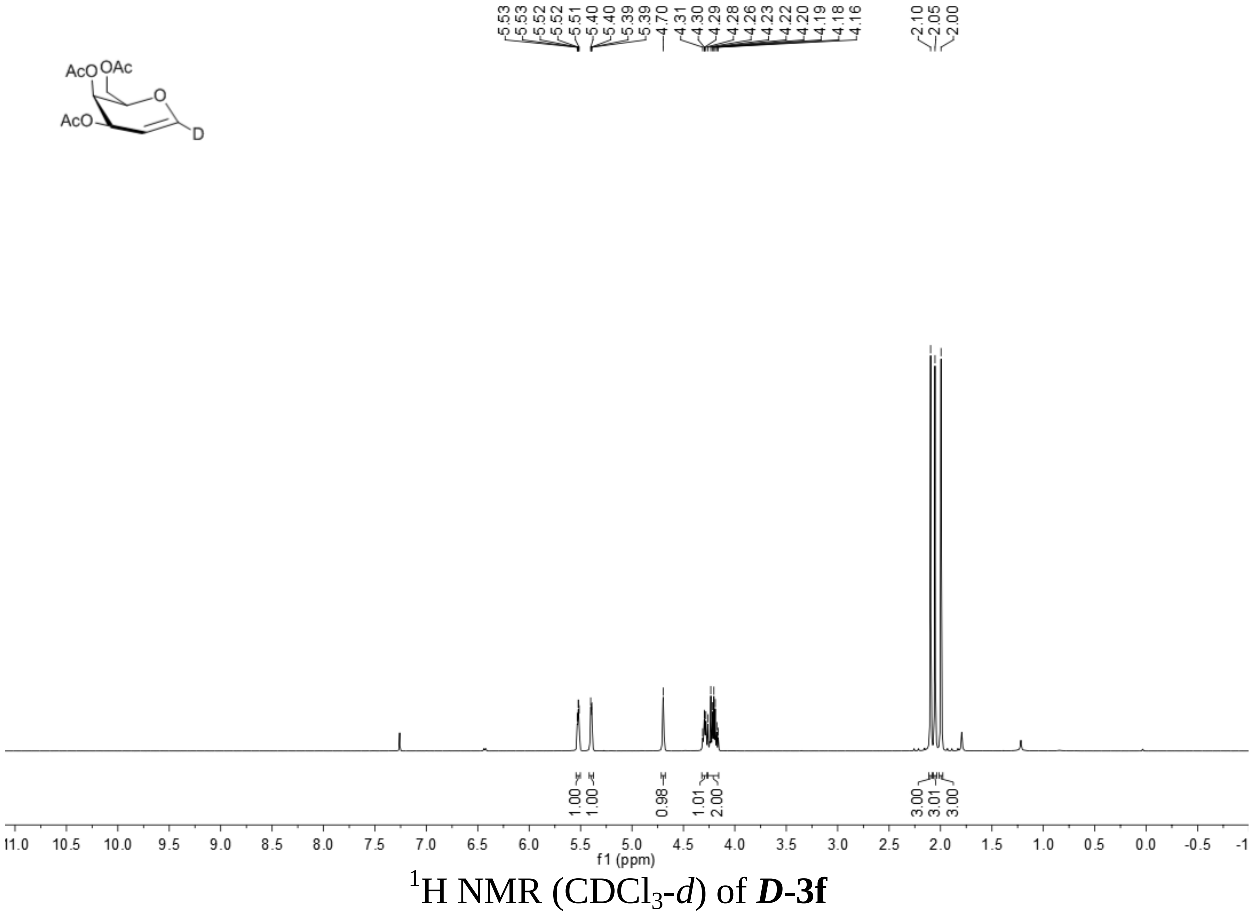

^{1}H NMR ($CDCl_3$-*d*) of ***D*-3f**

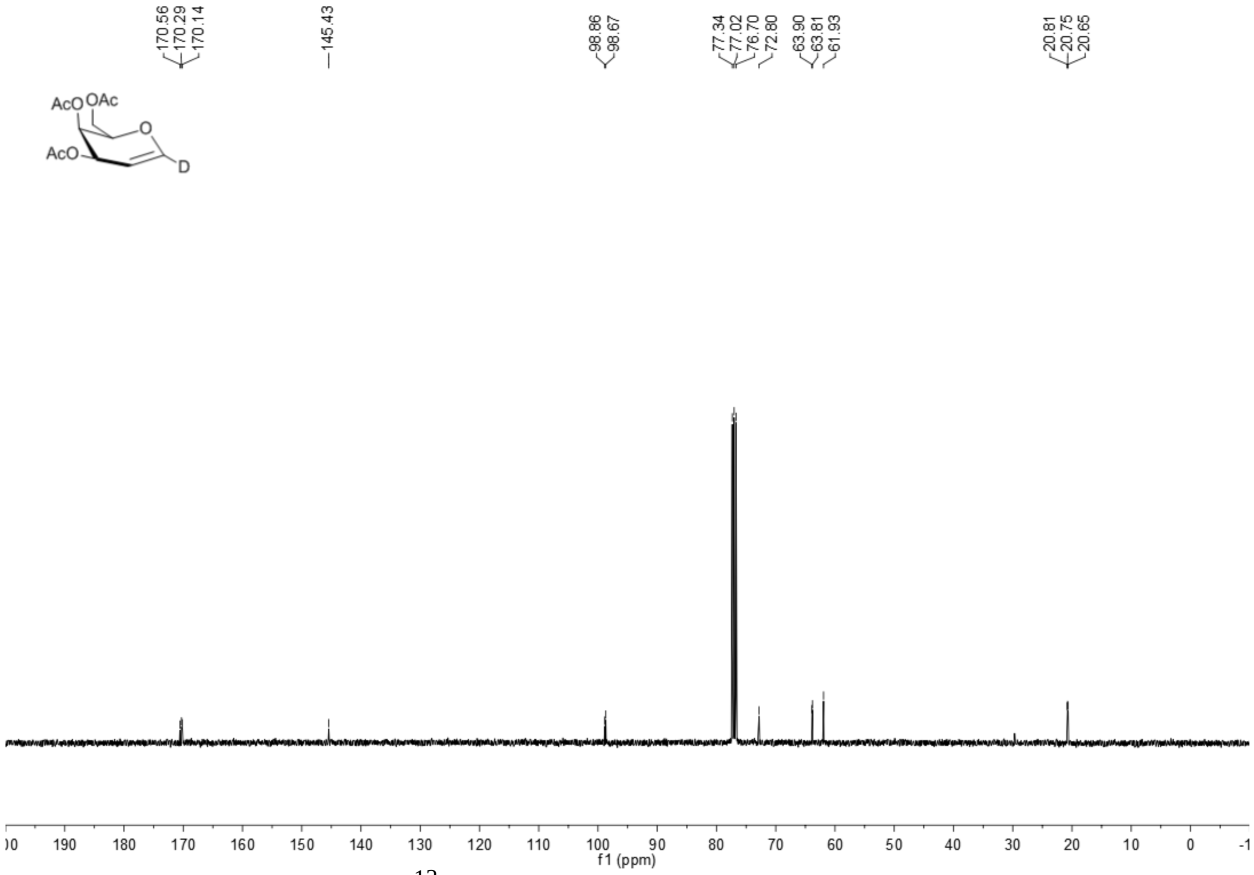

^{13}C NMR ($CDCl_3$-*d*) of ***D*-3f**

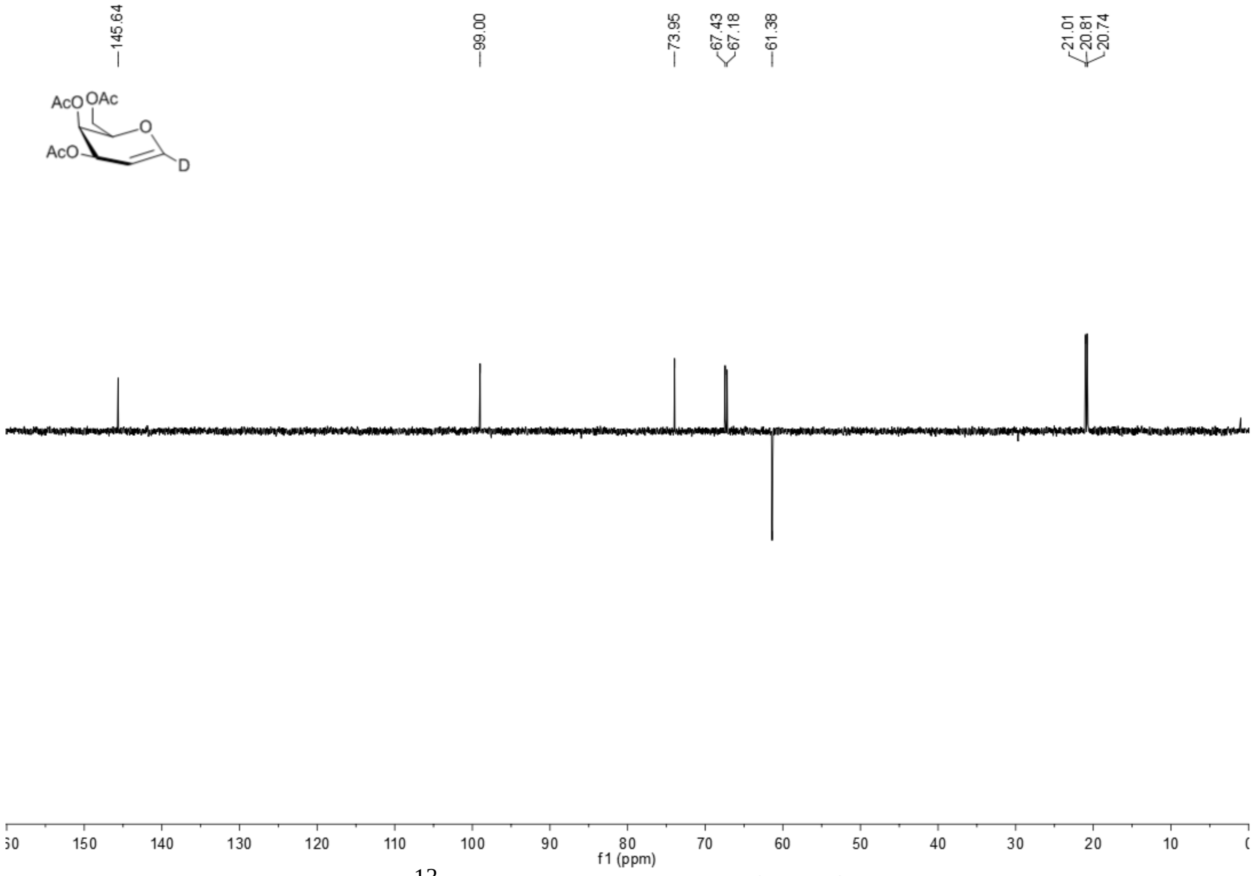

^{13}C NMR ($CDCl_3$-*d*) of ***D*-3f**

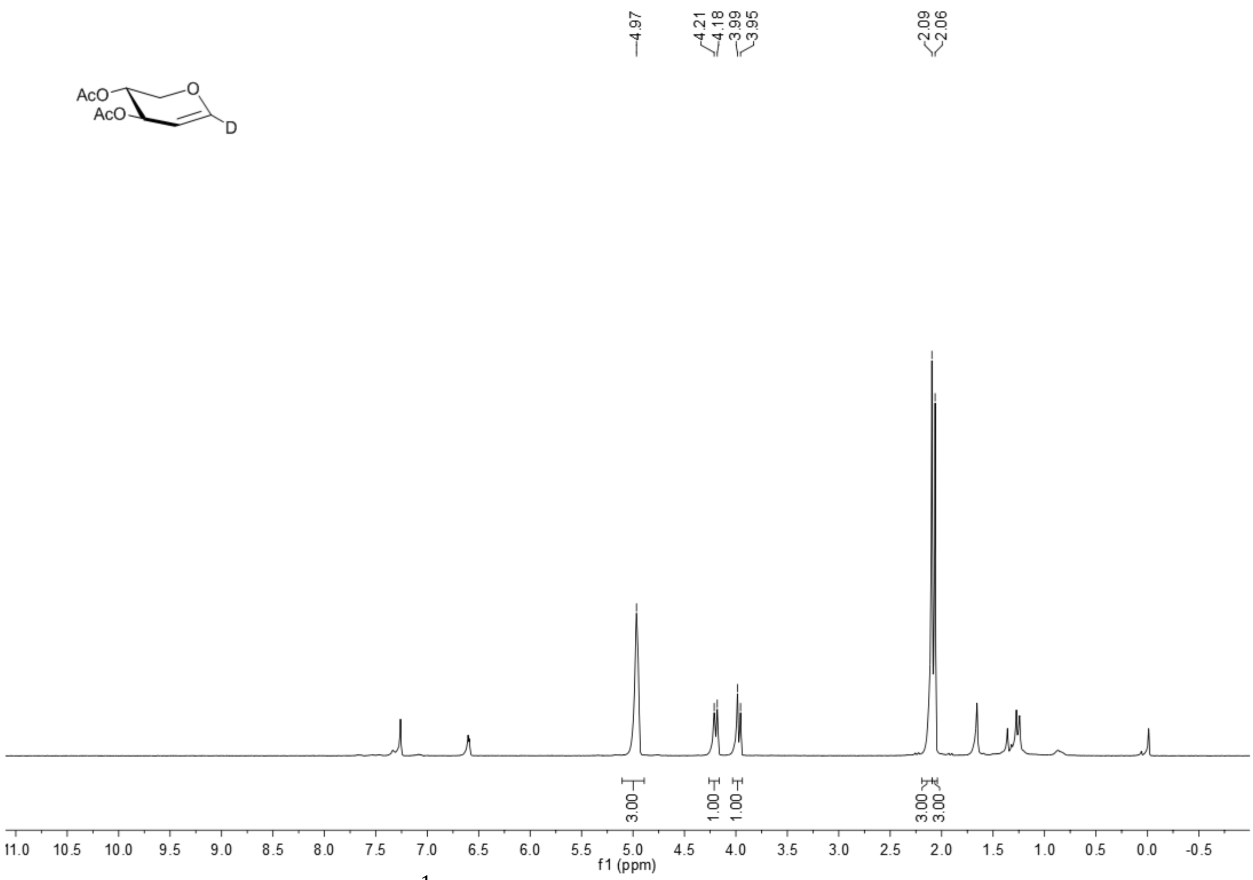

^{1}H NMR ($CDCl_3$-*d*) of ***D*-3i**

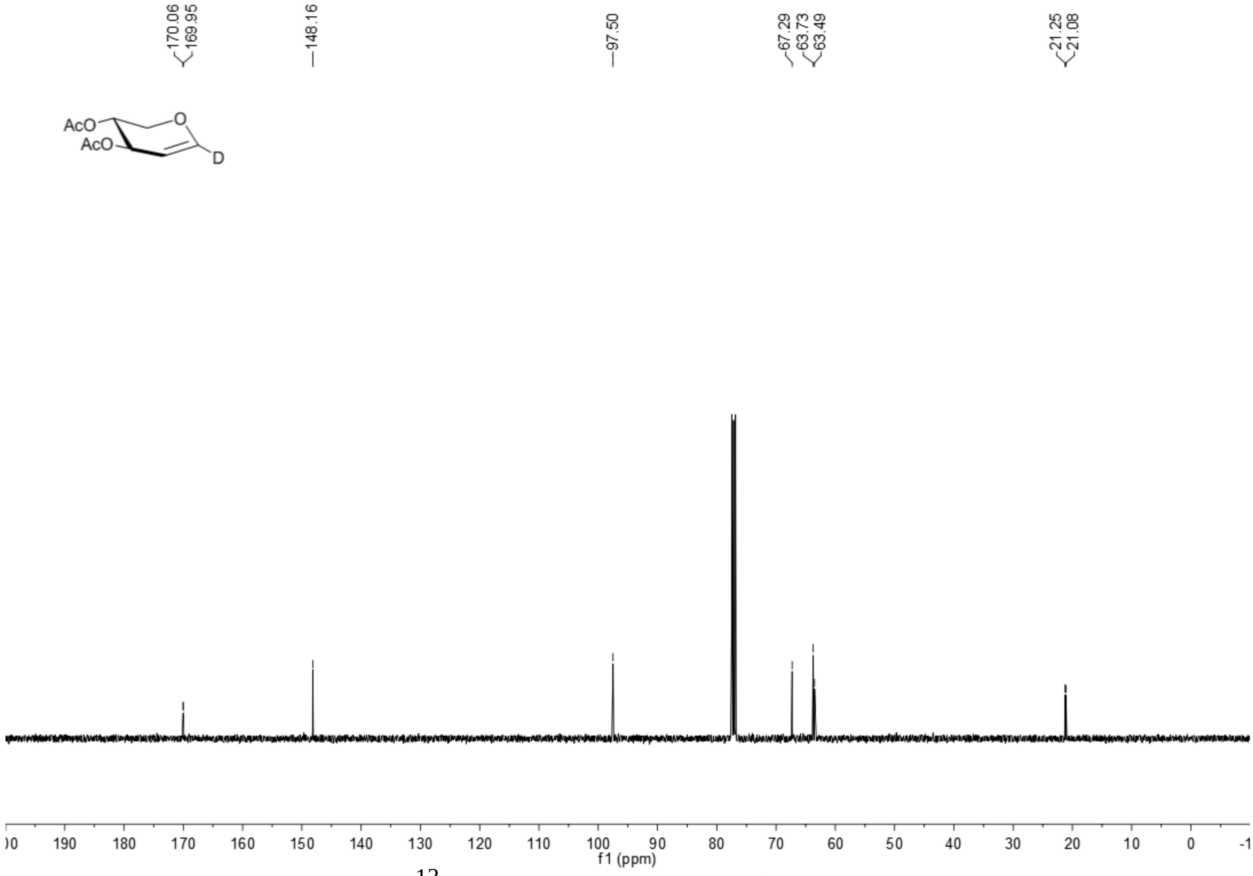

^{13}C NMR ($CDCl_3$-*d*) of ***D*-3i**

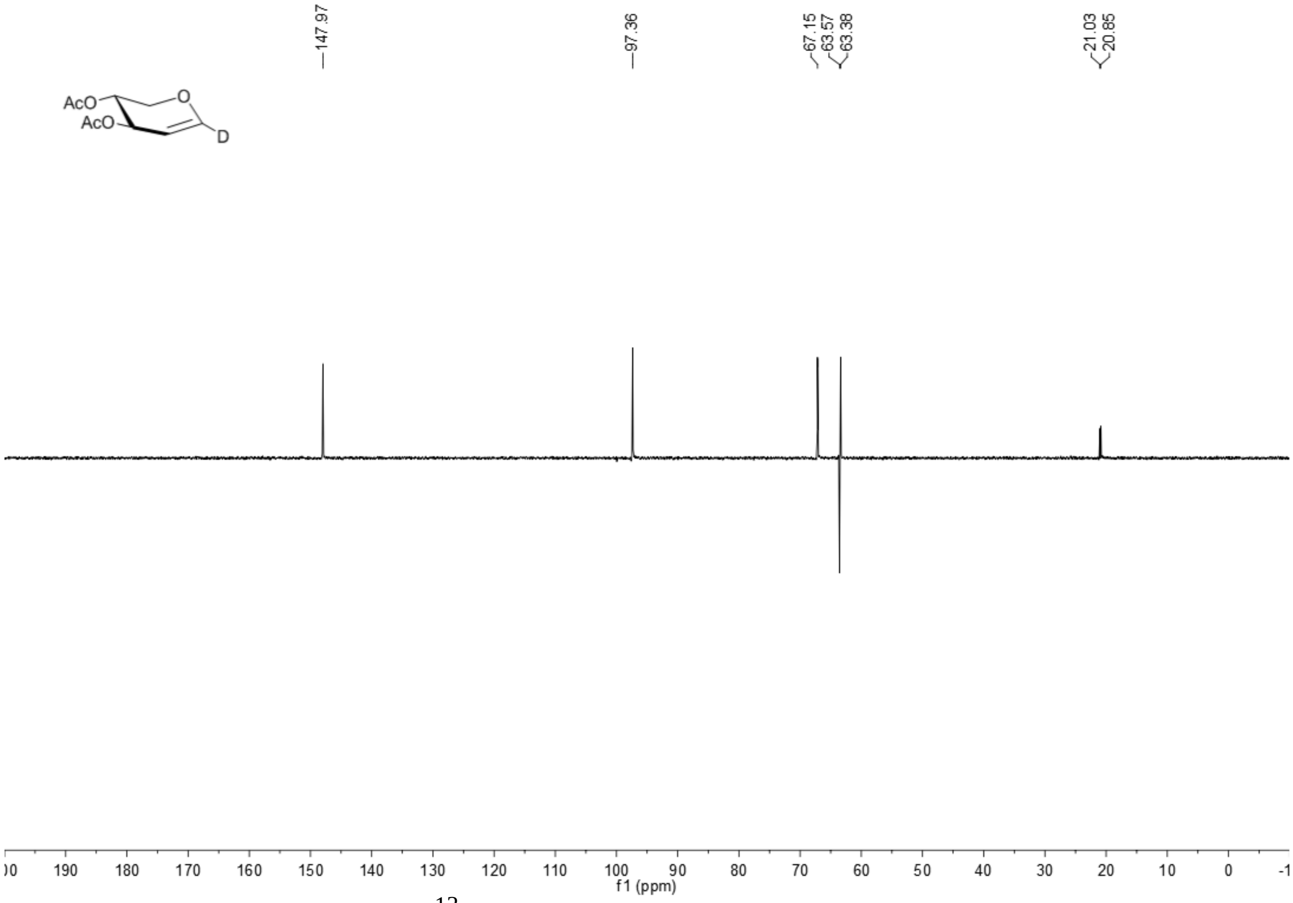

^{13}C NMR ($CDCl_3$-*d*) of ***D*-3i**

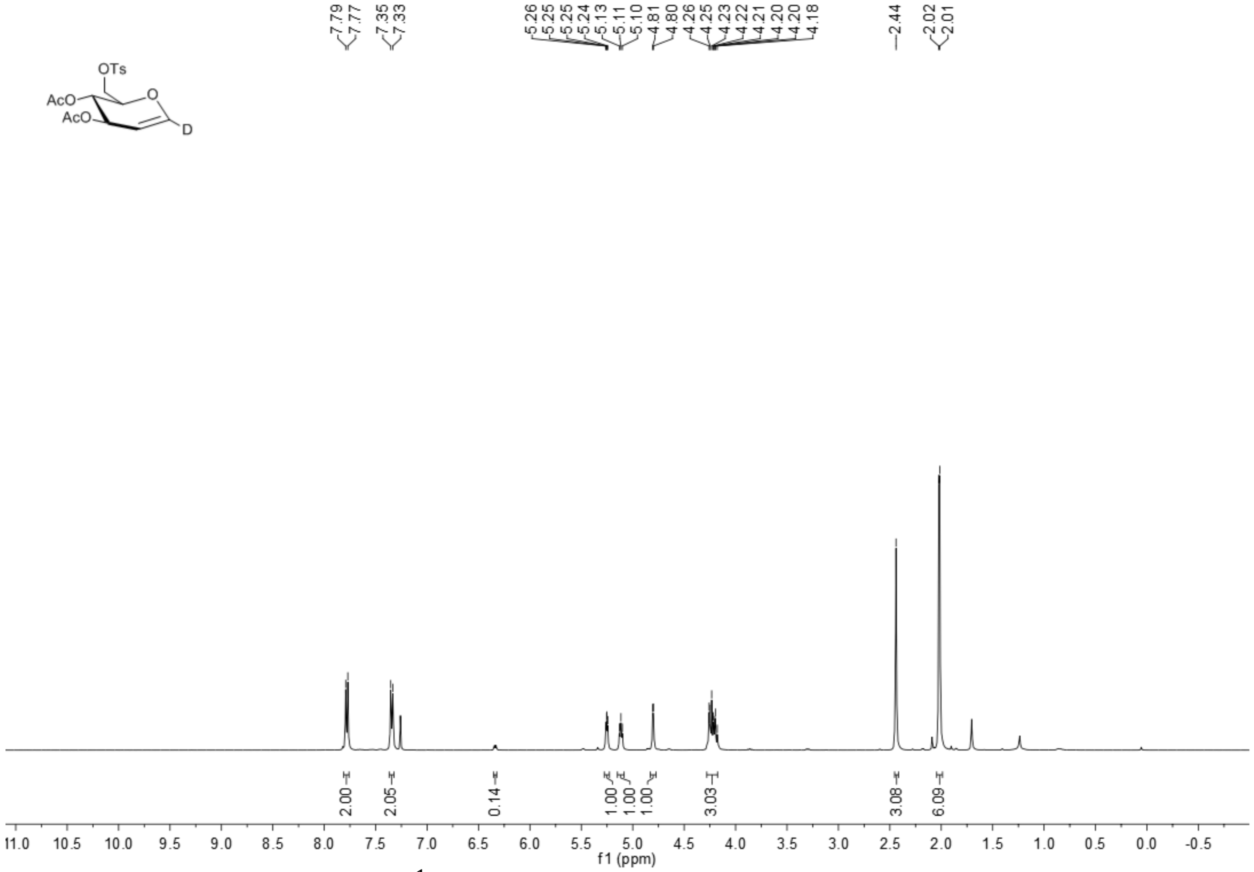

^{1}H NMR ($CDCl_3$-*d*) of ***D*-3b**

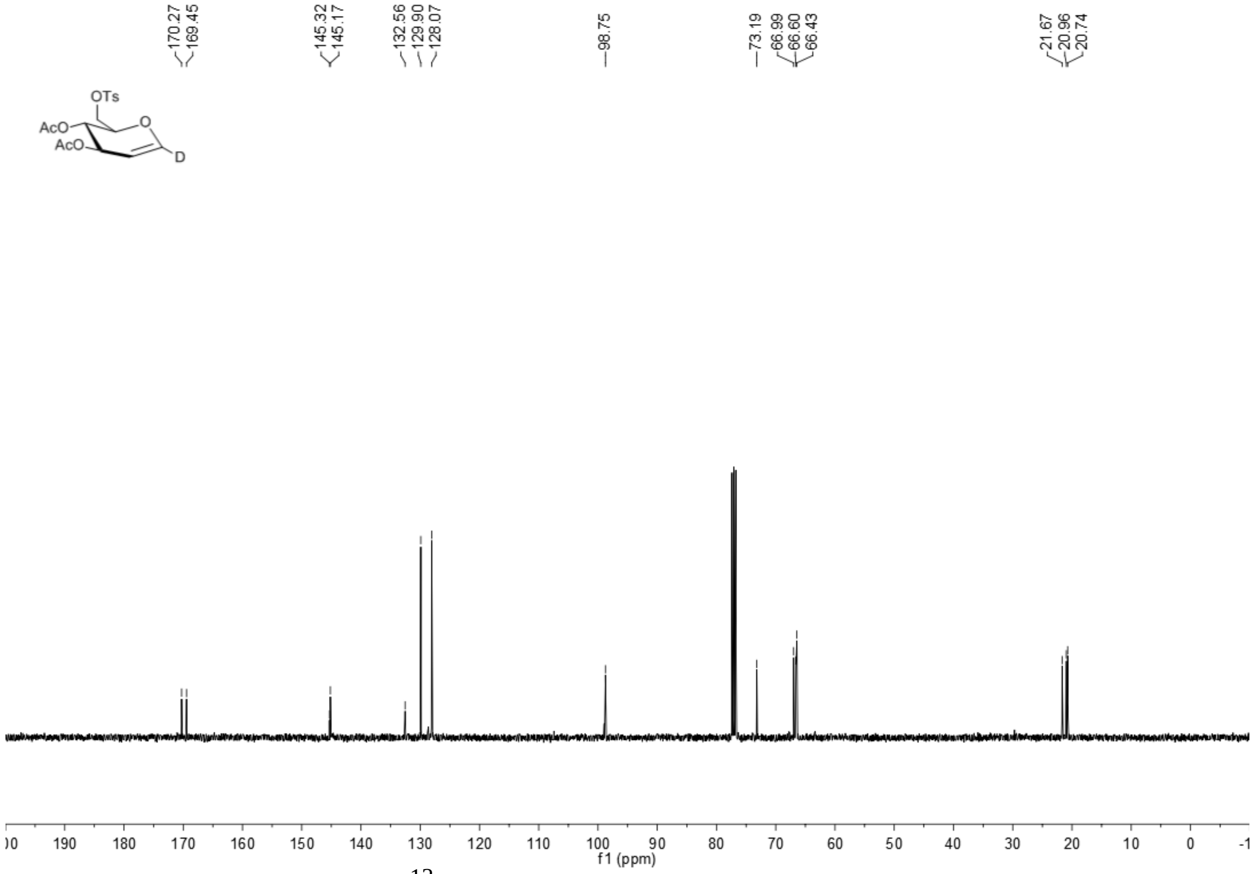

^{13}C NMR ($CDCl_3$-*d*) of ***D*-3b**

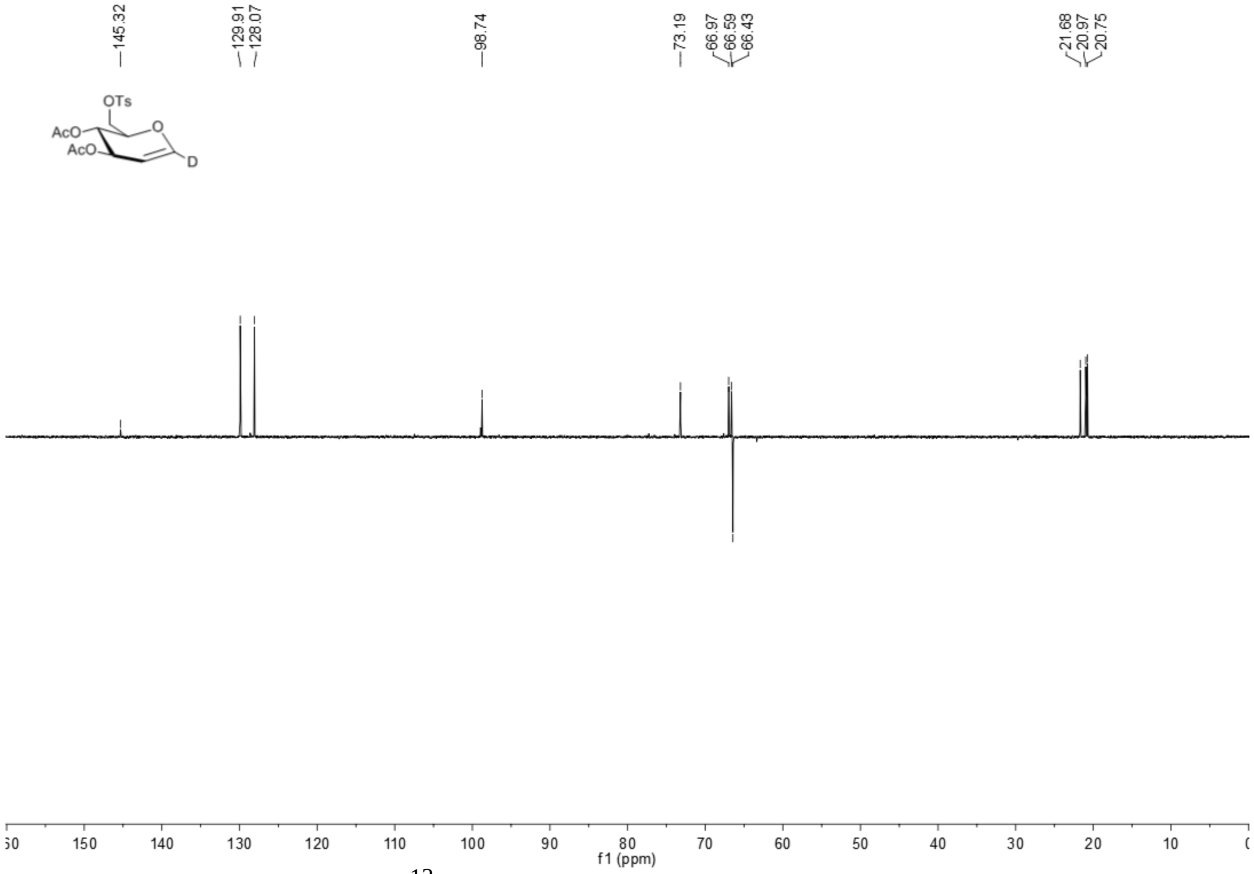

^{13}C NMR ($CDCl_3$-*d*) of ***D*-3b**

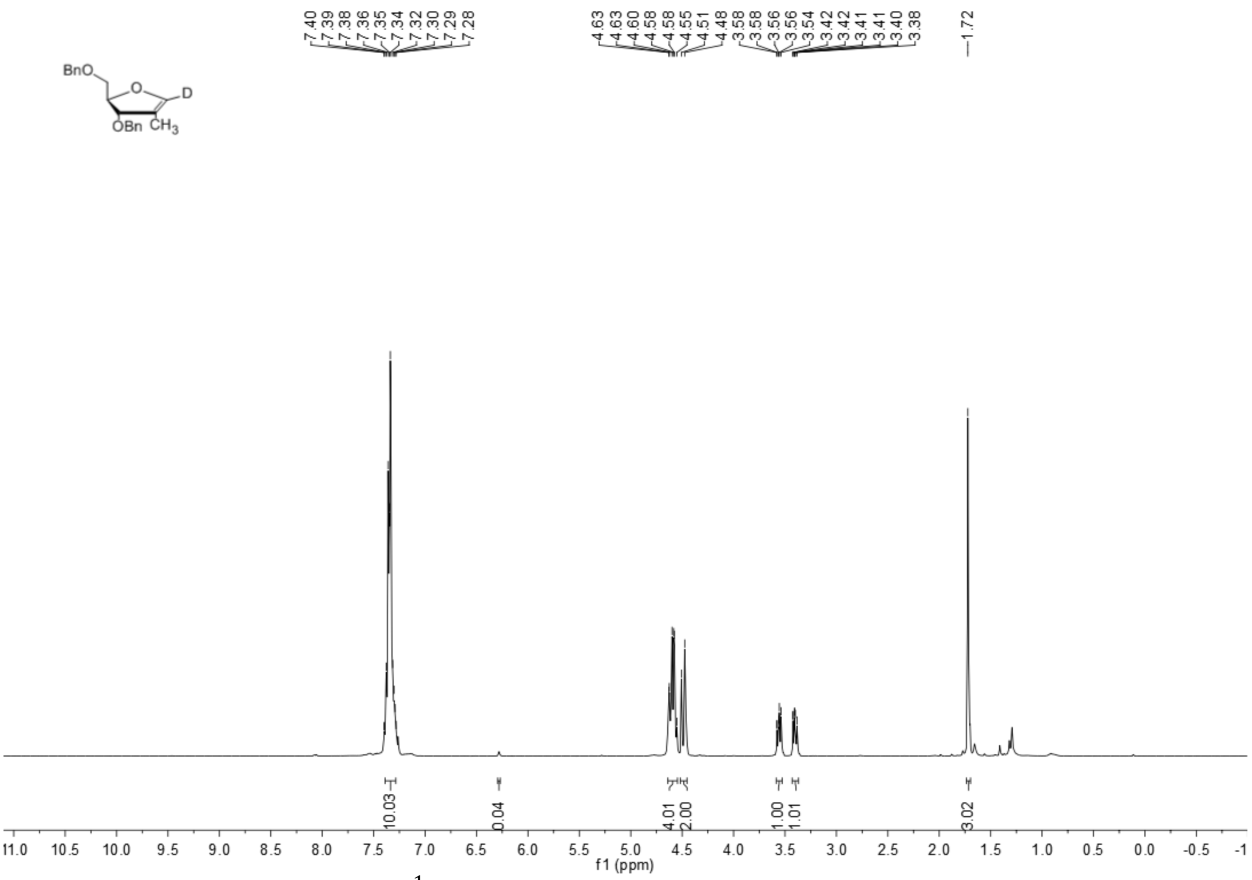

^{1}H NMR ($CDCl_3$-*d*) of ***D*-3q**

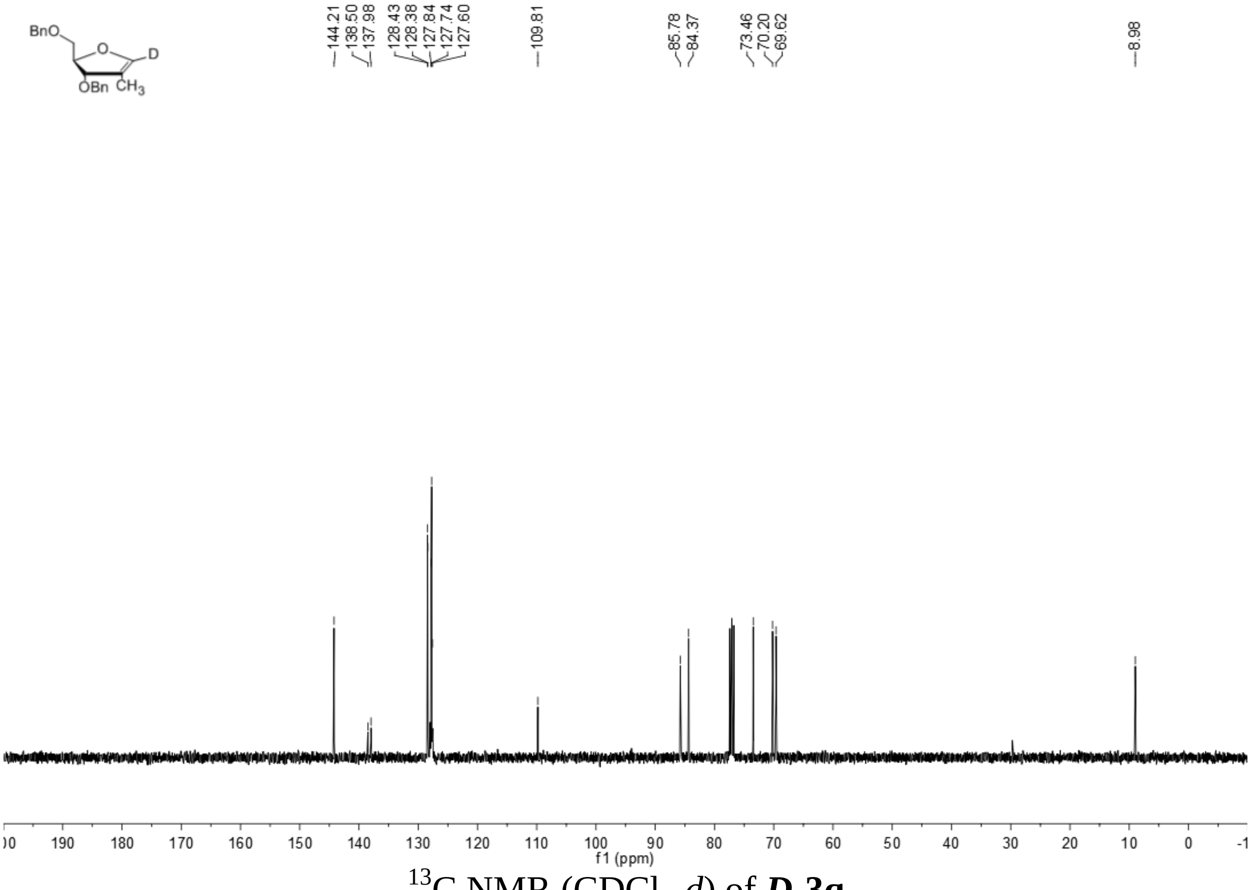

^{13}C NMR ($CDCl_3$-*d*) of ***D*-3q**

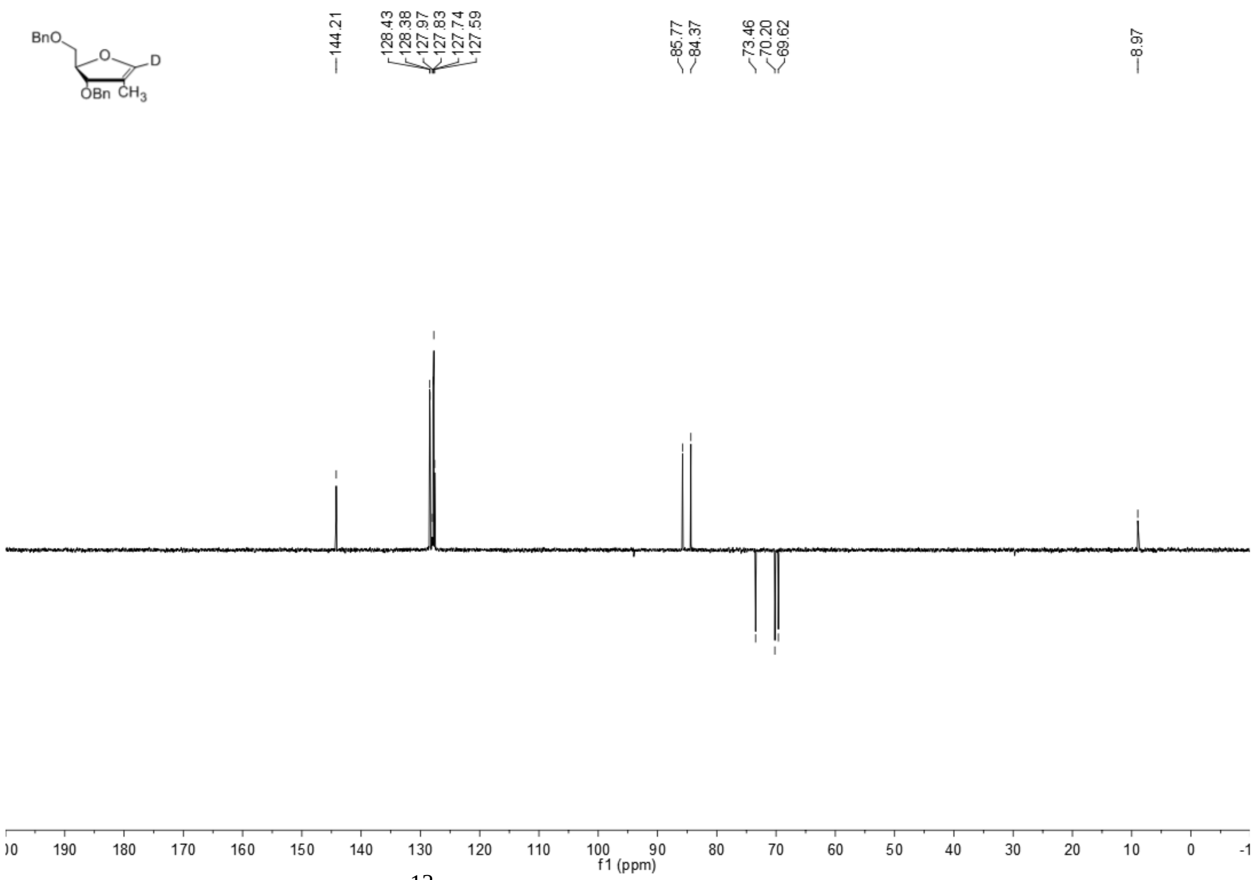

^{13}C NMR ($CDCl_3$-*d*) of ***D*-3q**

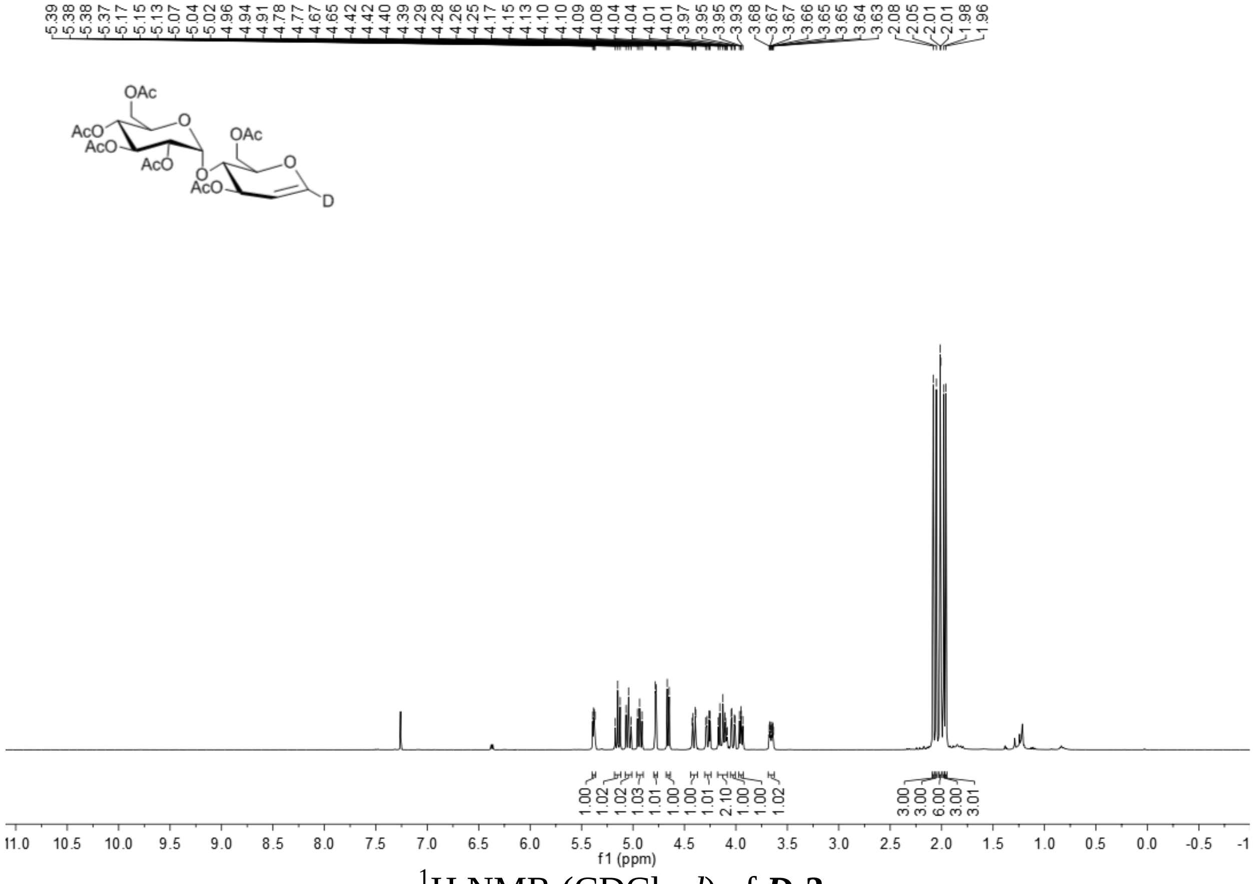

^{1}H NMR ($CDCl_3$-*d*) of ***D*-3s**

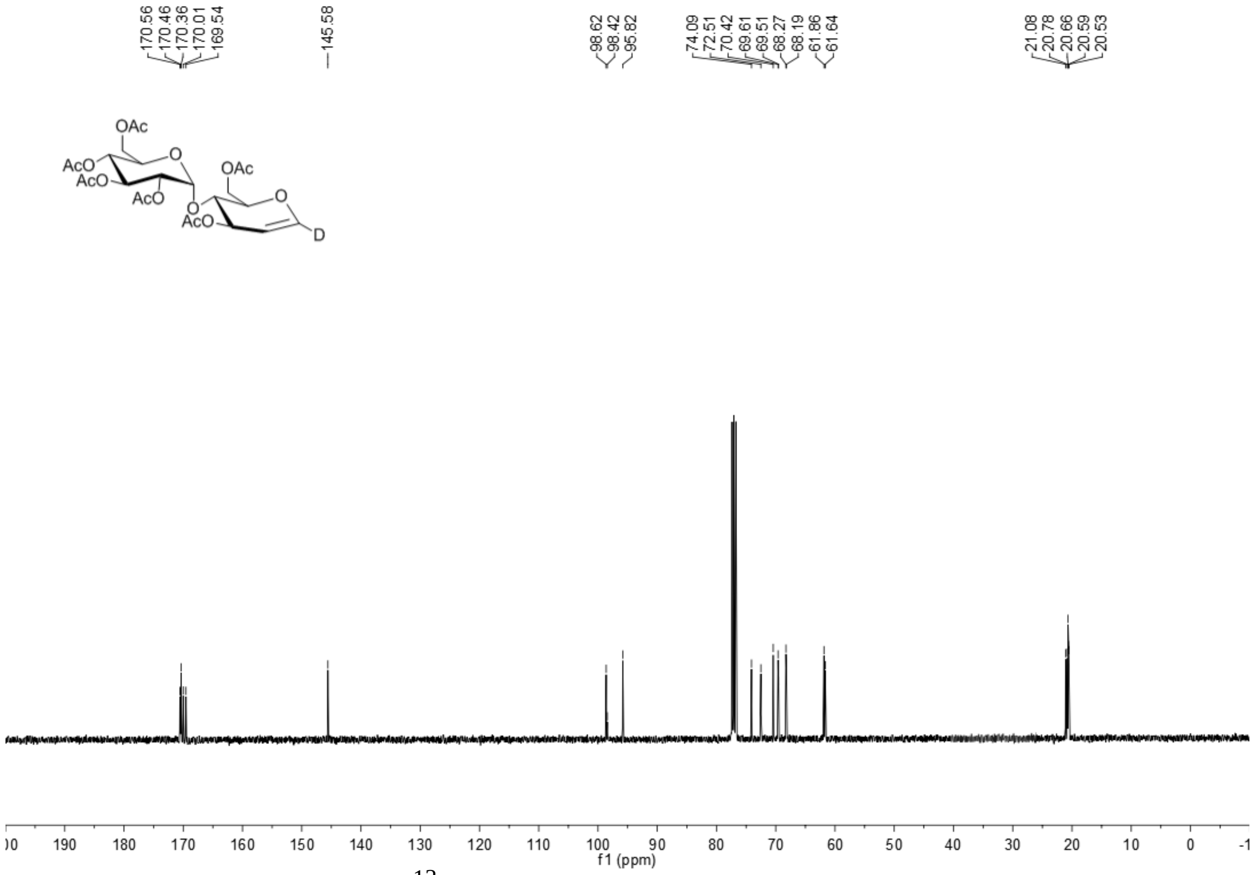

^{13}C NMR ($CDCl_3$-*d*) of ***D*-3s**

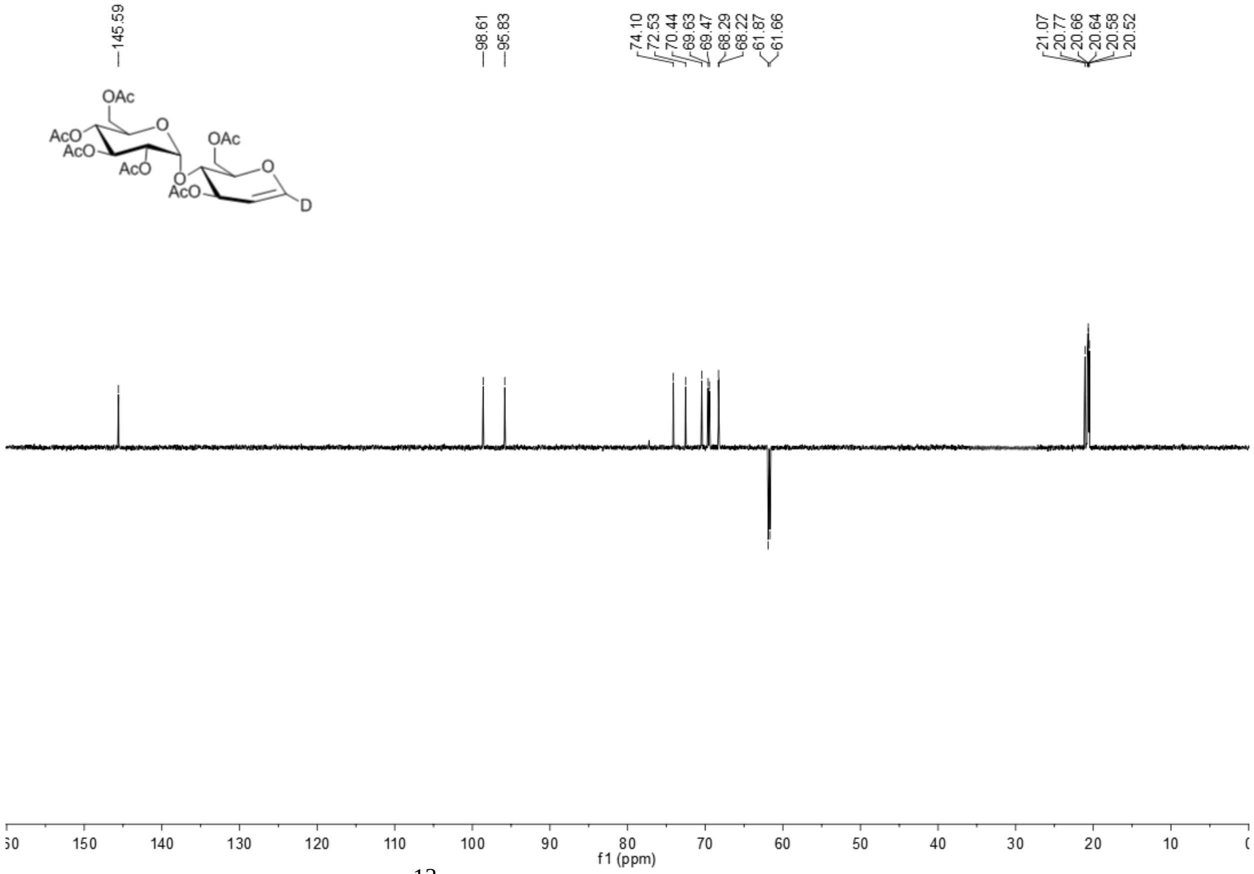

^{13}C NMR ($CDCl_3$-*d*) of ***D*-3s**

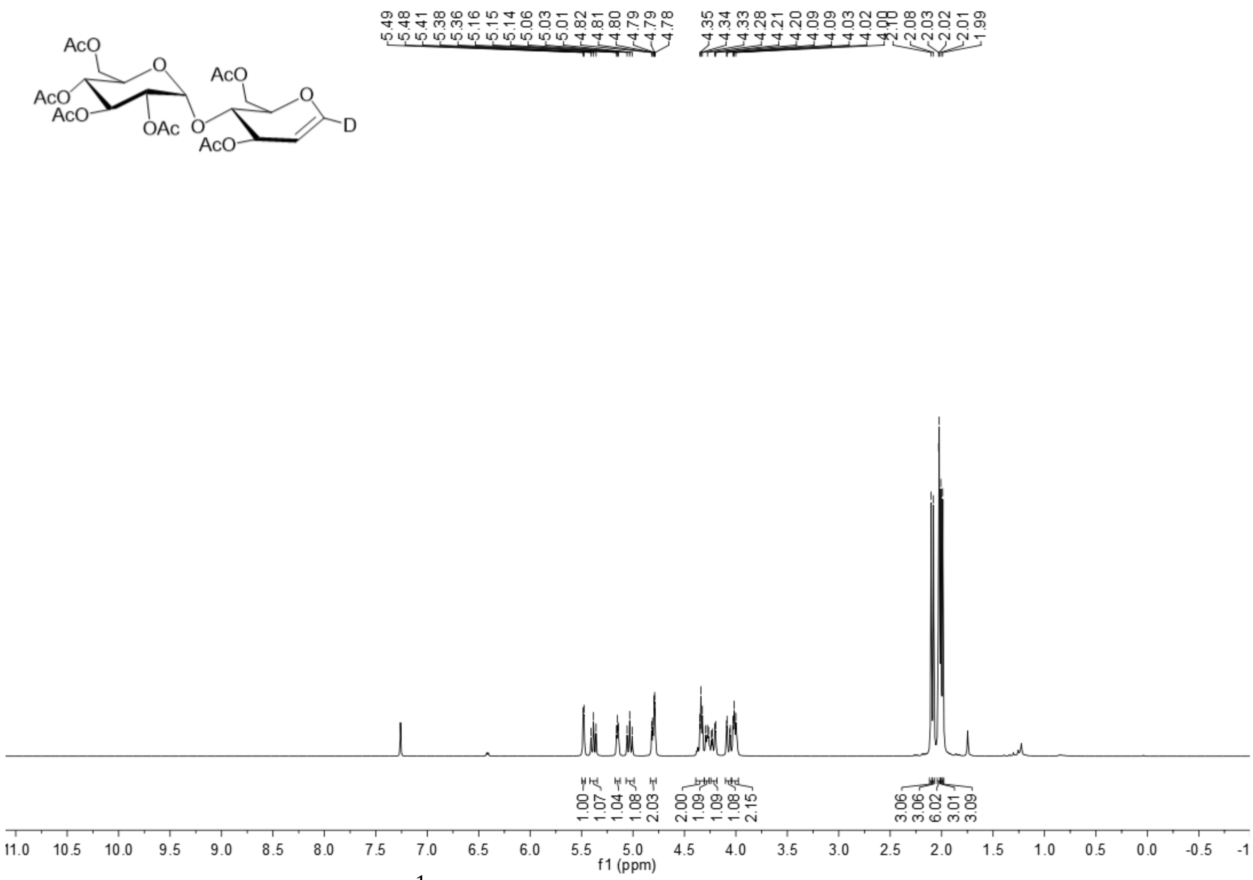

^{1}H NMR ($CDCl_3$-*d*) of ***D*-3u**

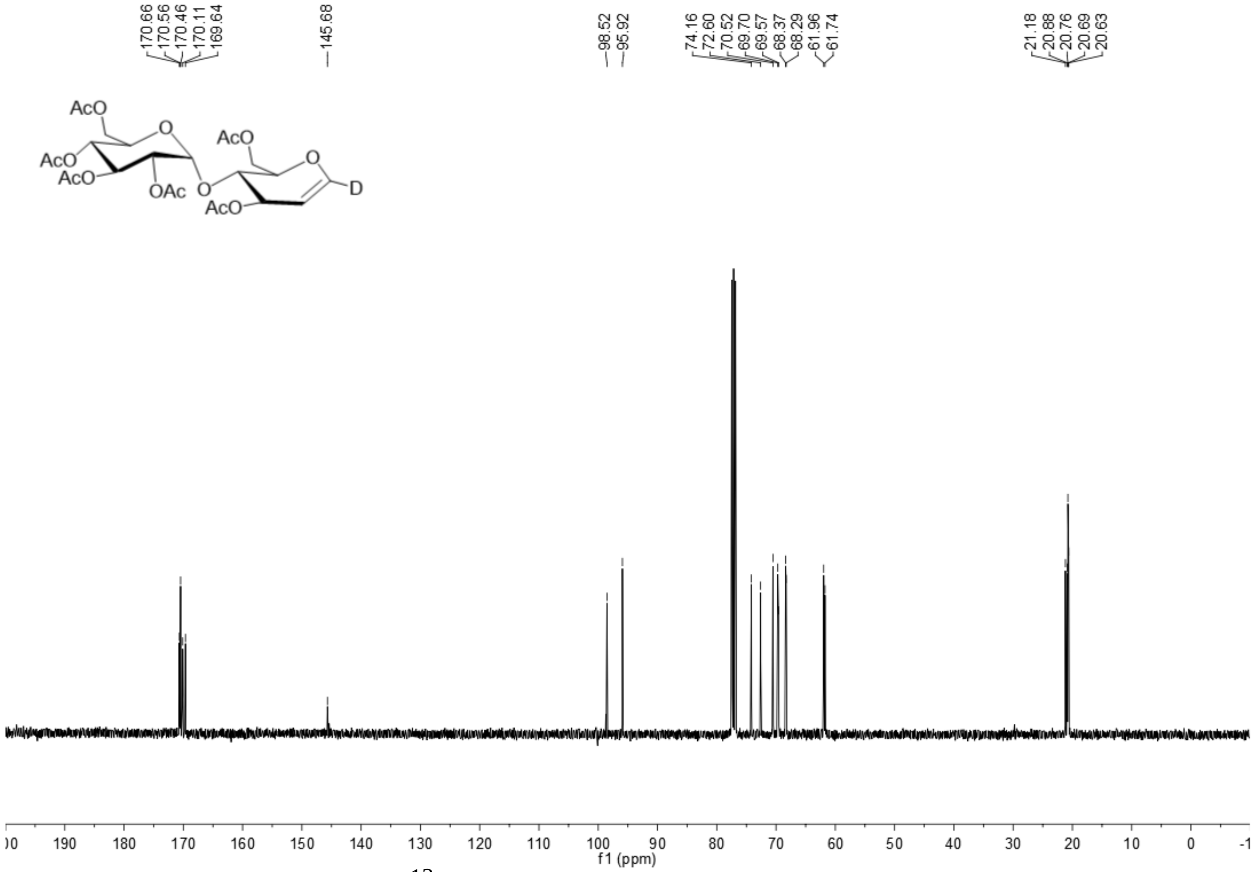

^{13}C NMR ($CDCl_3$-*d*) of ***D*-3u**

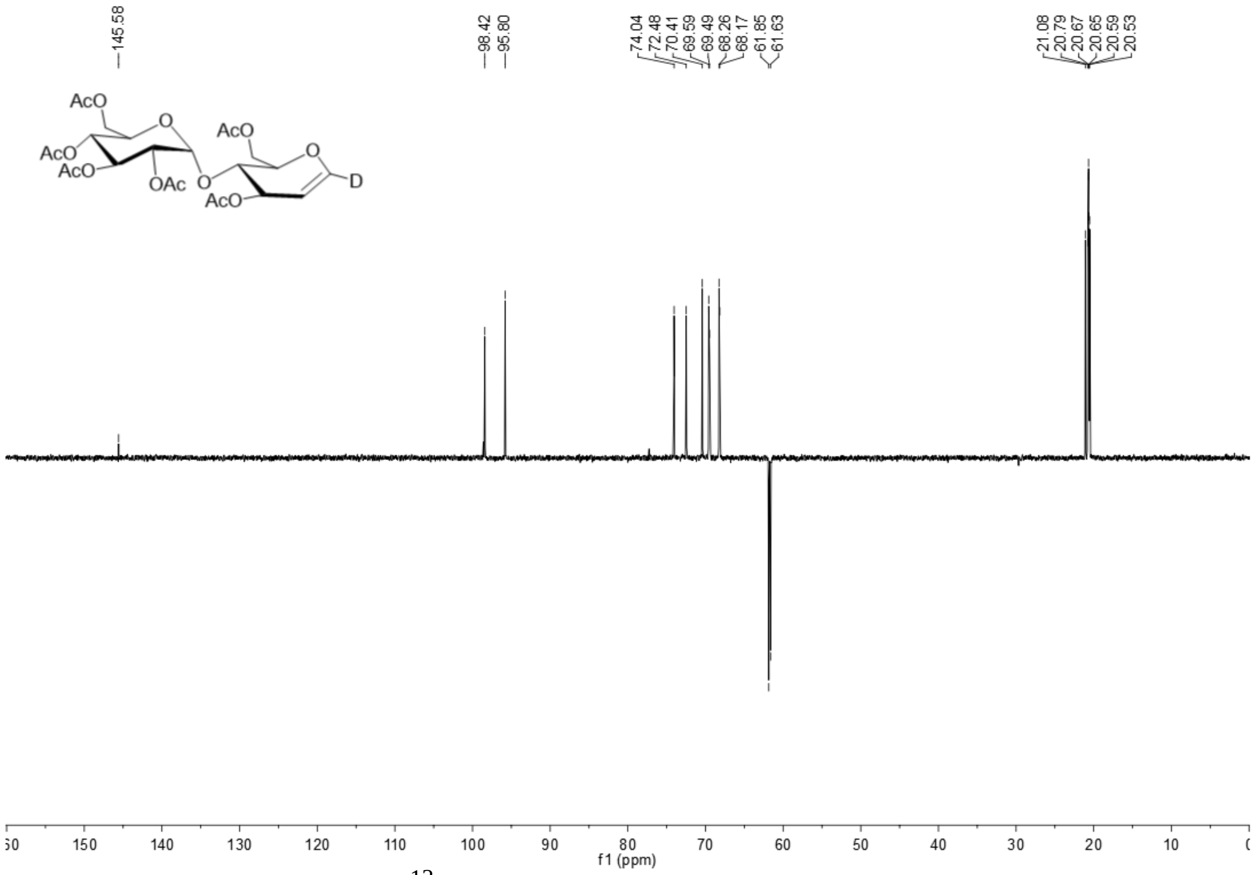

^{13}C NMR ($CDCl_3$-*d*) of ***D*-3u**

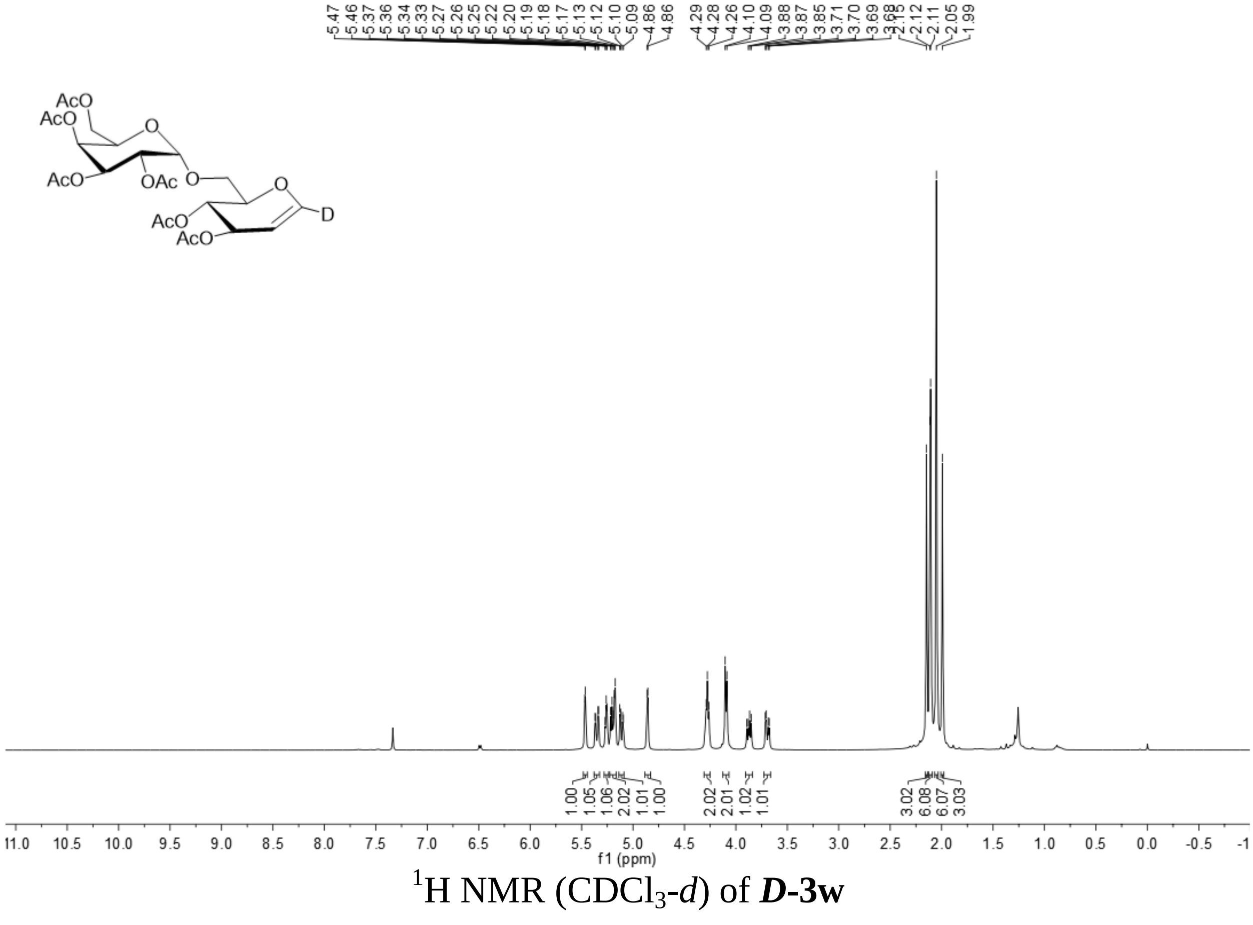

^{1}H NMR ($CDCl_3$-*d*) of ***D*-3w**

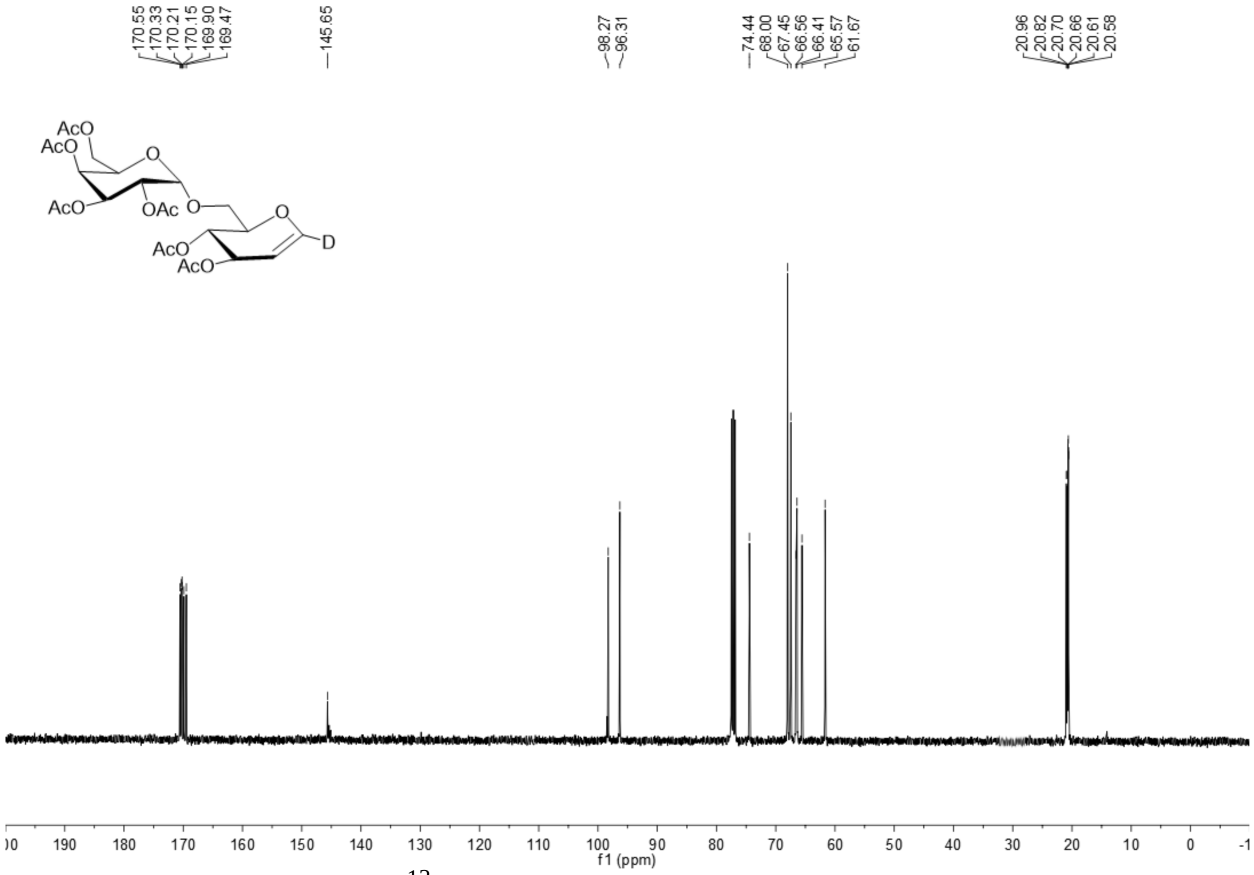

^{13}C NMR ($CDCl_3$-*d*) of ***D*-3w**

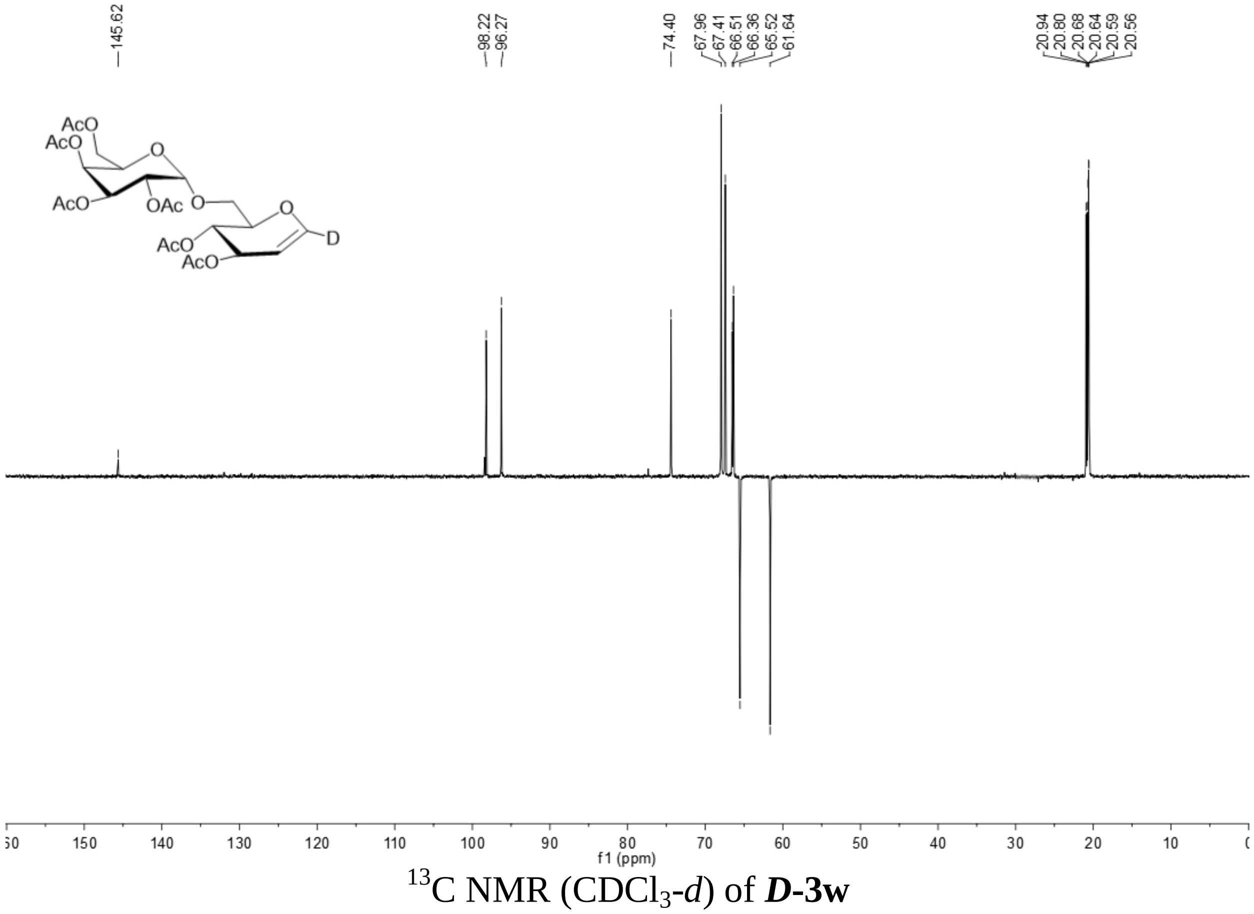

^{13}C NMR ($CDCl_3$-*d*) of ***D*-3w**

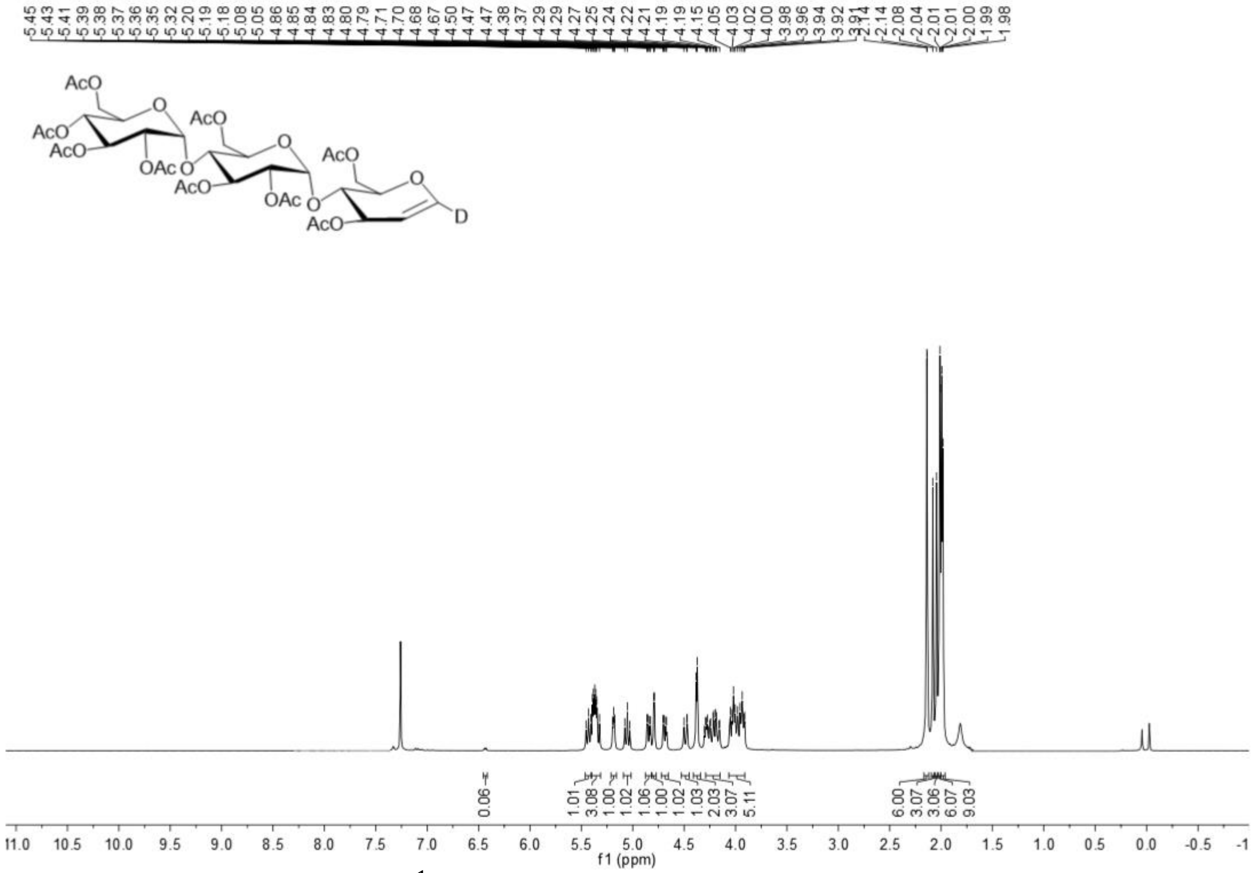

^{1}H NMR ($CDCl_3$-*d*) of ***D*-3x**

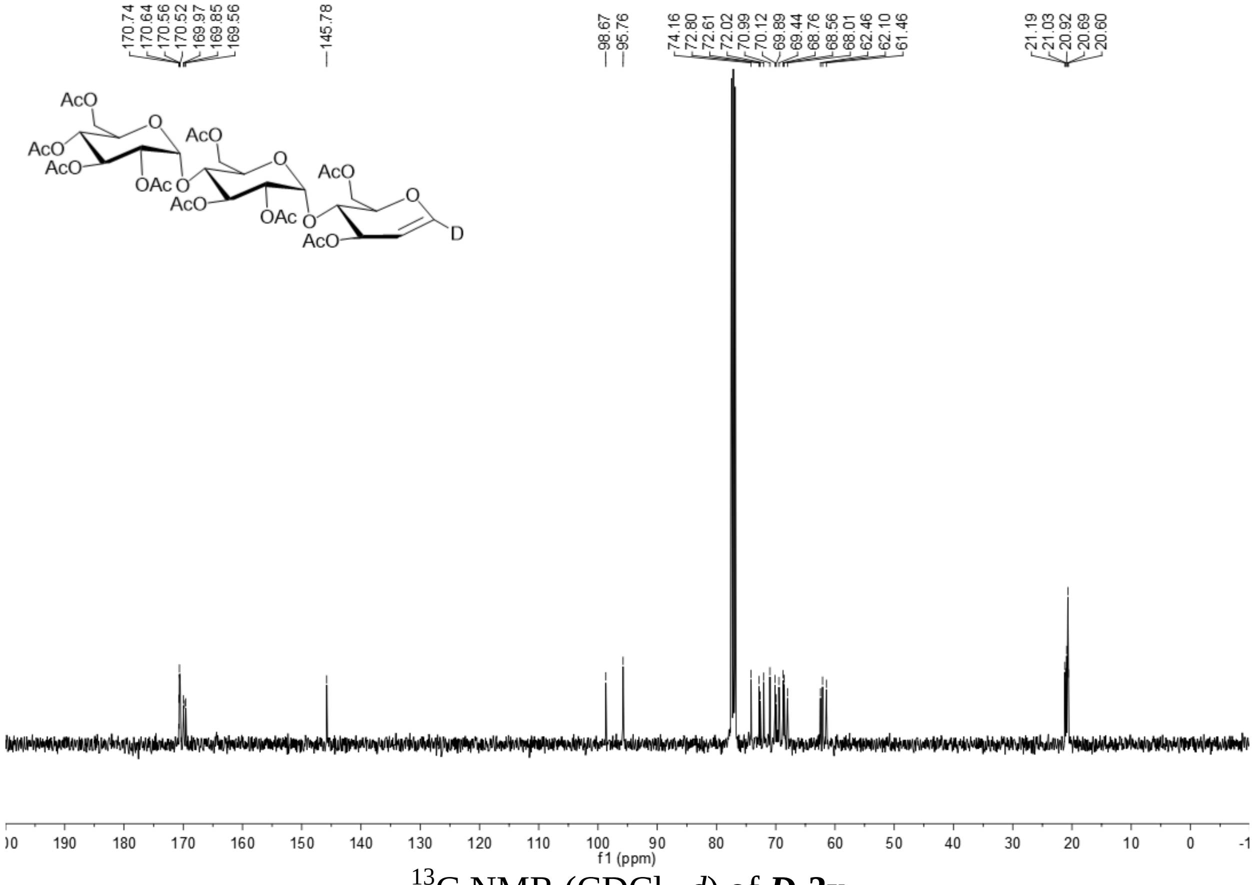

^{13}C NMR ($CDCl_3$-*d*) of ***D*-3x**

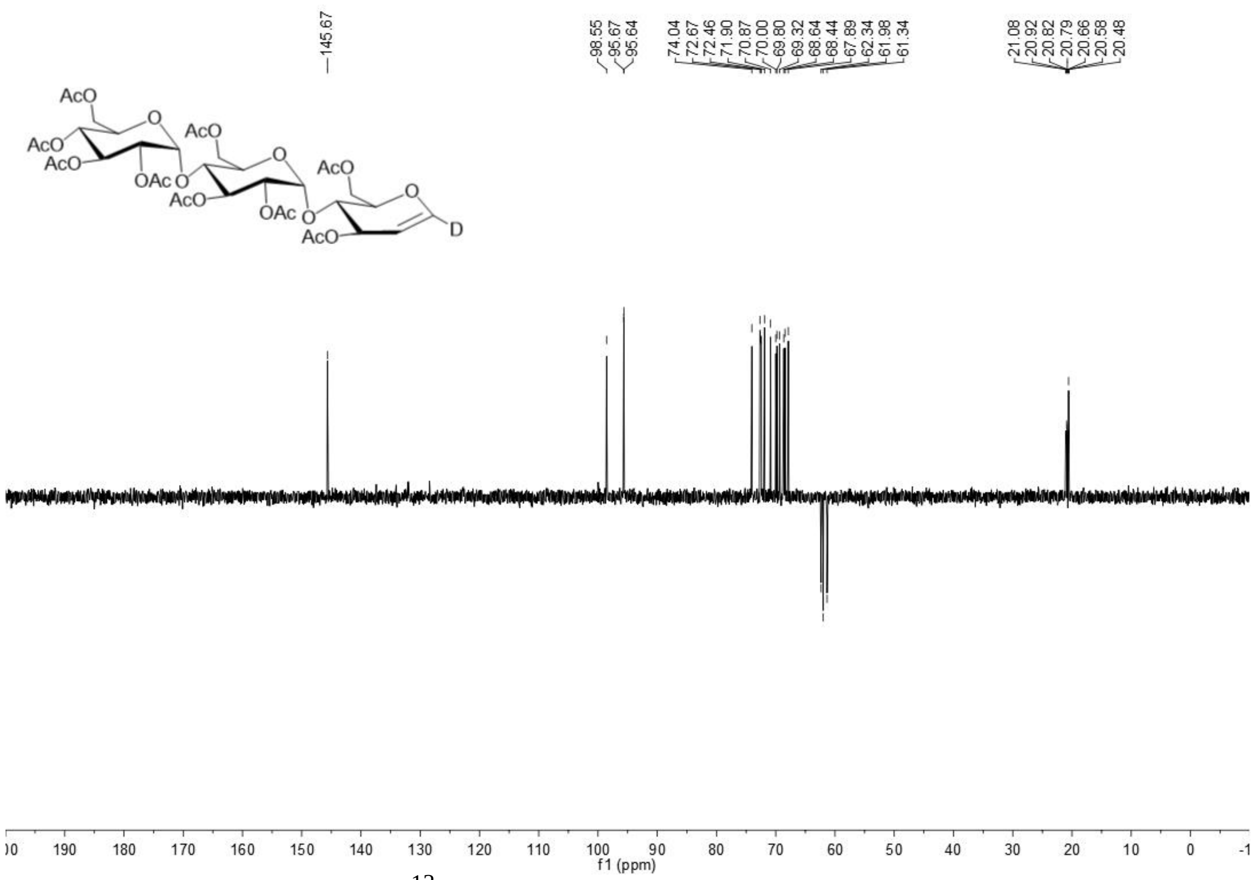

^{13}C NMR ($CDCl_3$-*d*) of ***D*-3x**

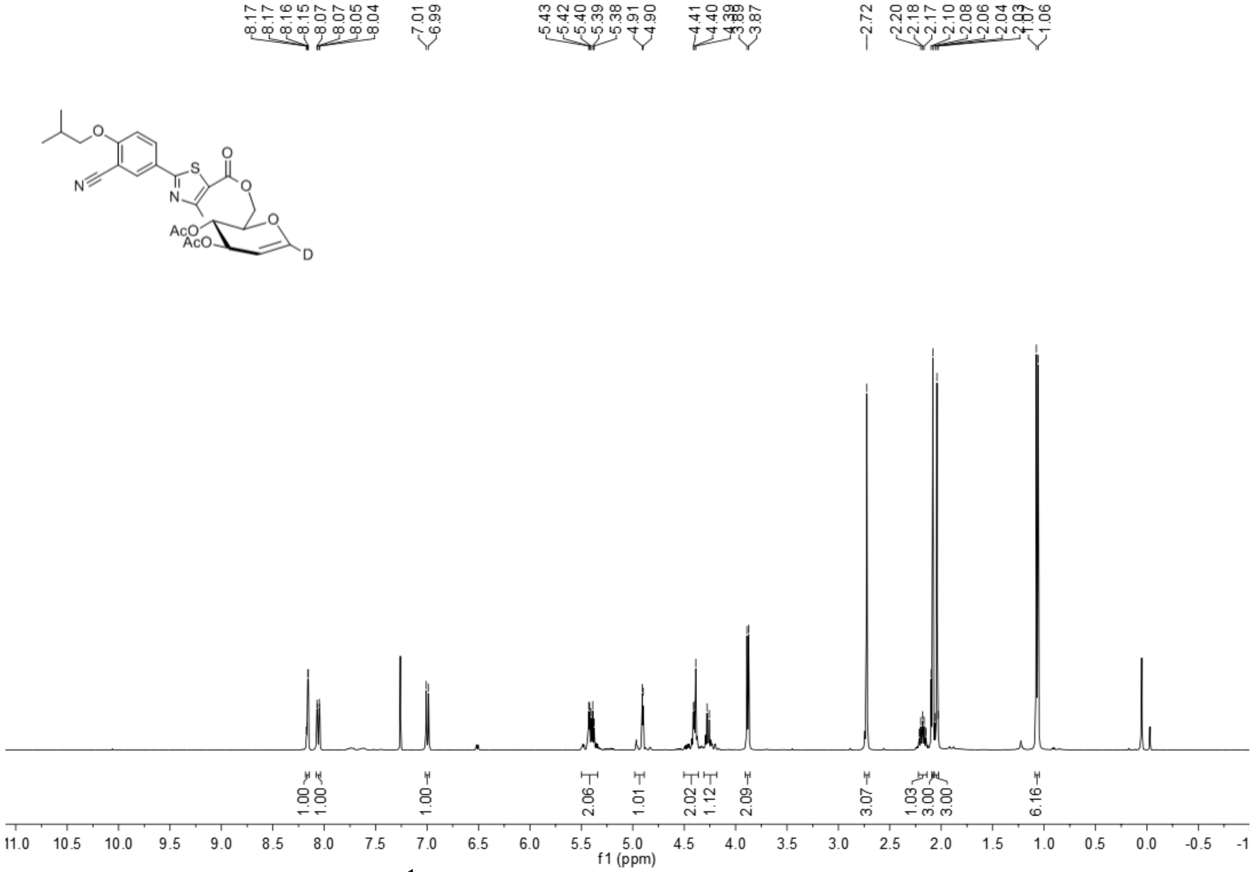

^{1}H NMR ($CDCl_3$-*d*) of ***D*-3ab**

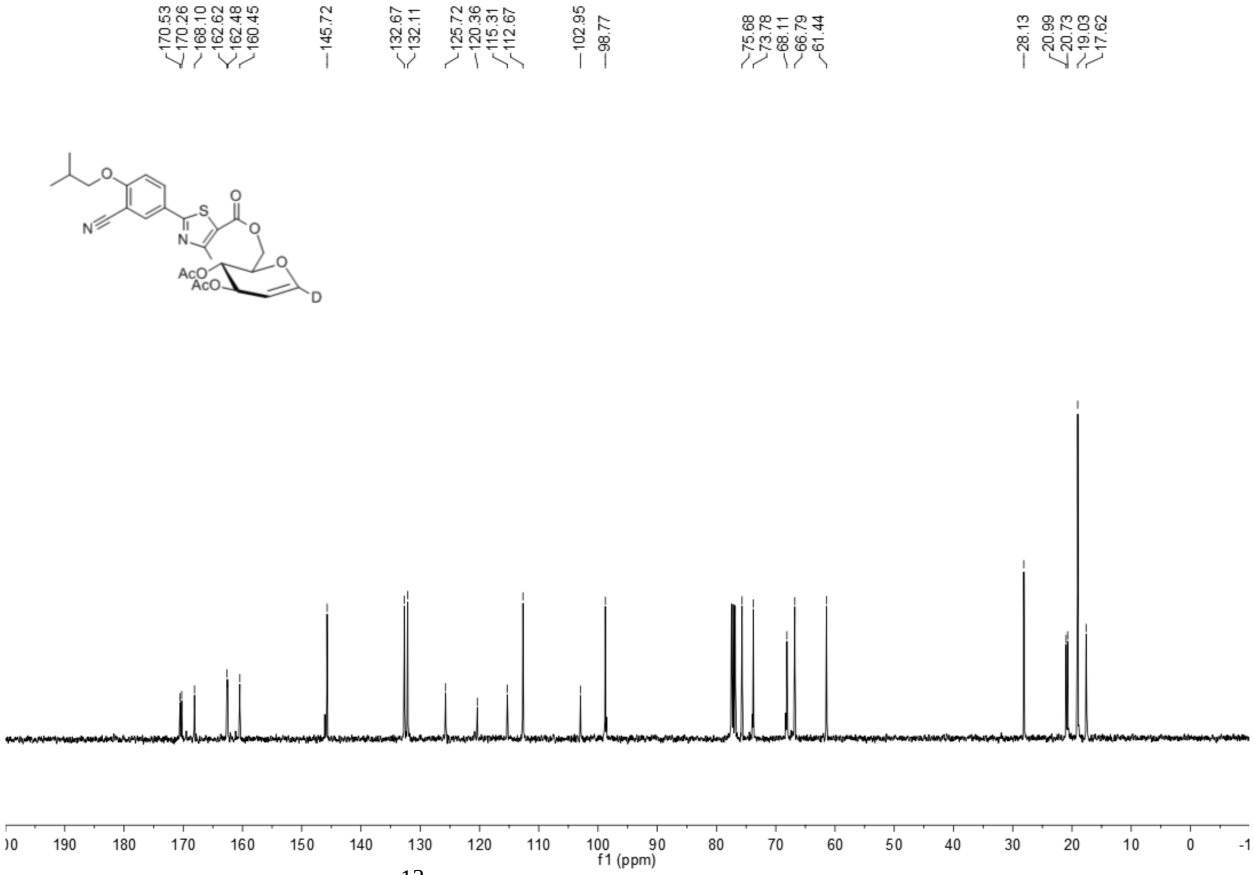

^{13}C NMR ($CDCl_3$-*d*) of ***D*-3ab**

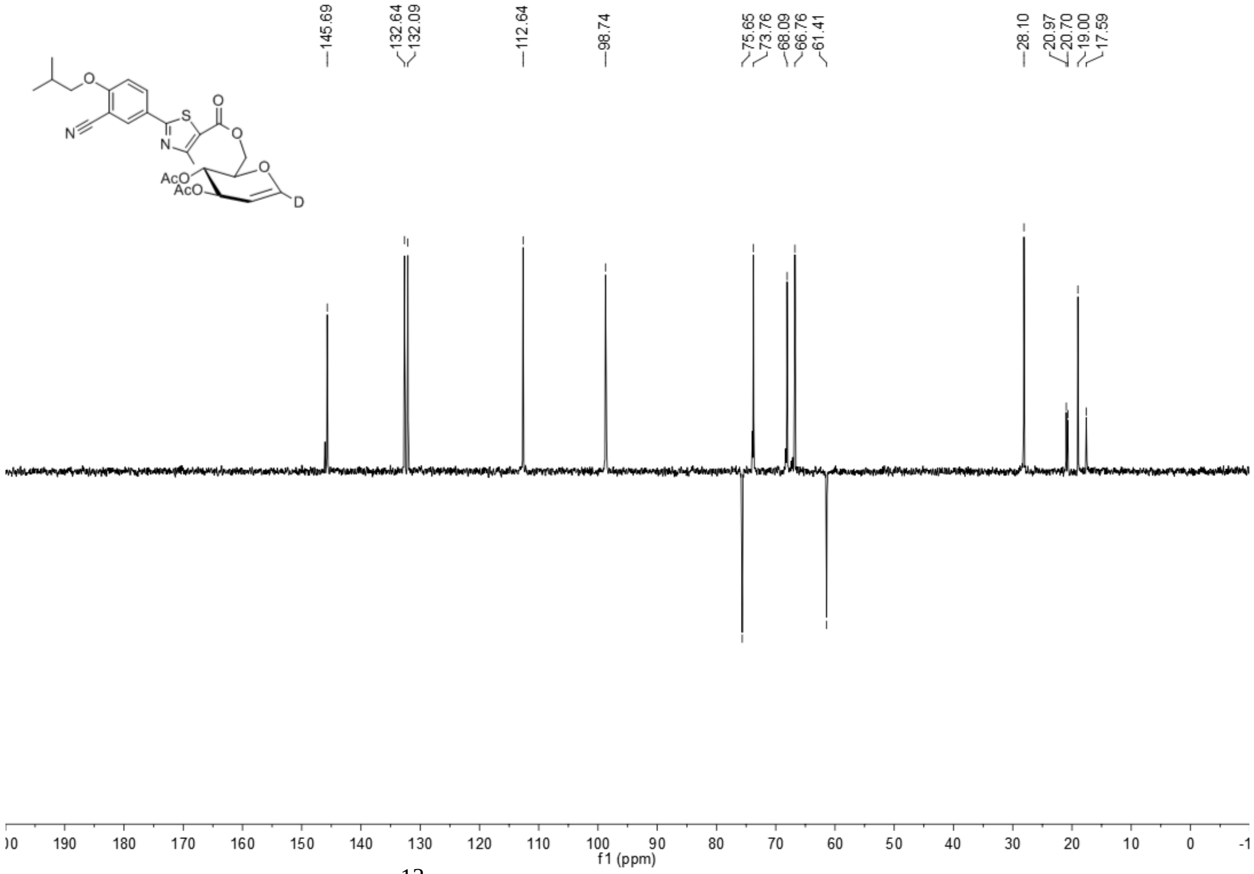

^{13}C NMR ($CDCl_3$-*d*) of ***D*-3ab**

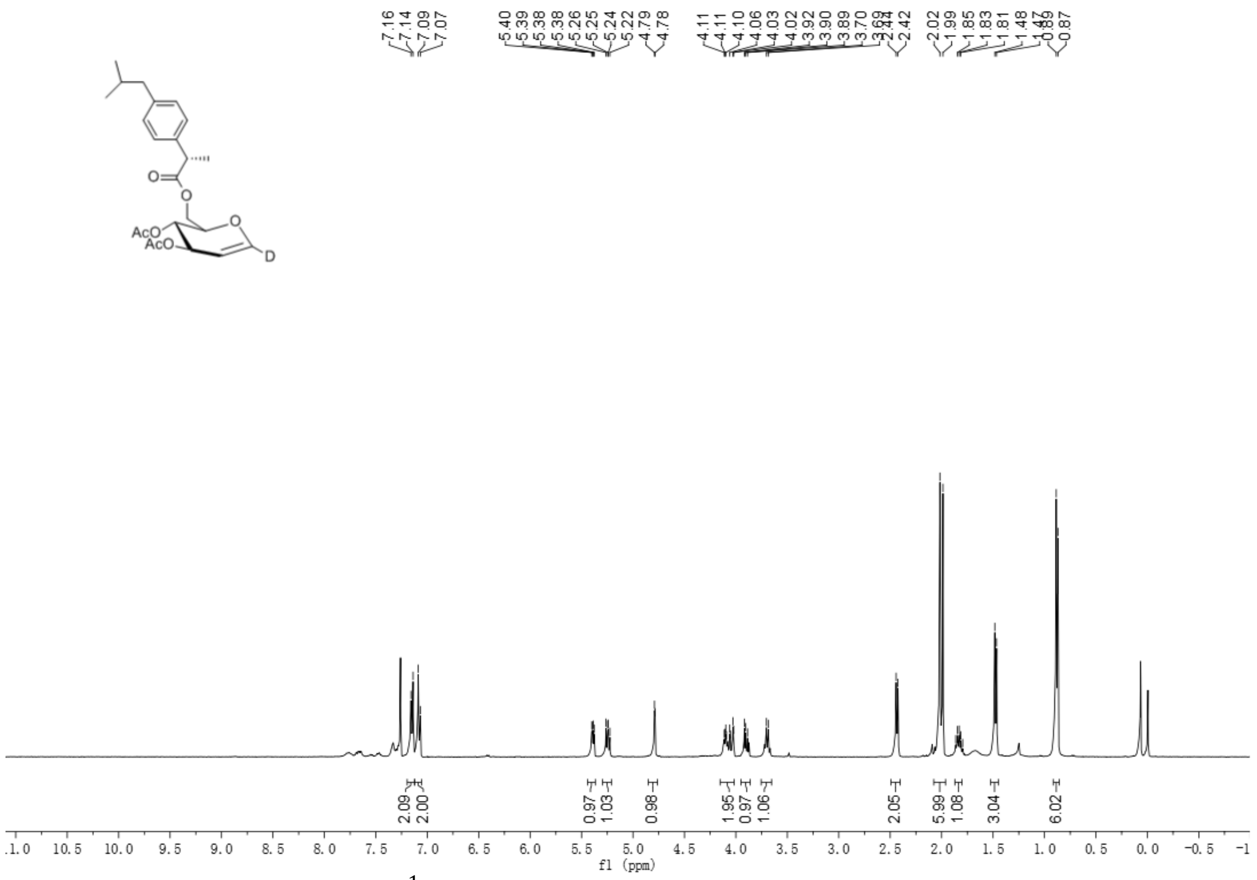

^{1}H NMR (CDCl$_3$-*d*) of ***D*-3ac**

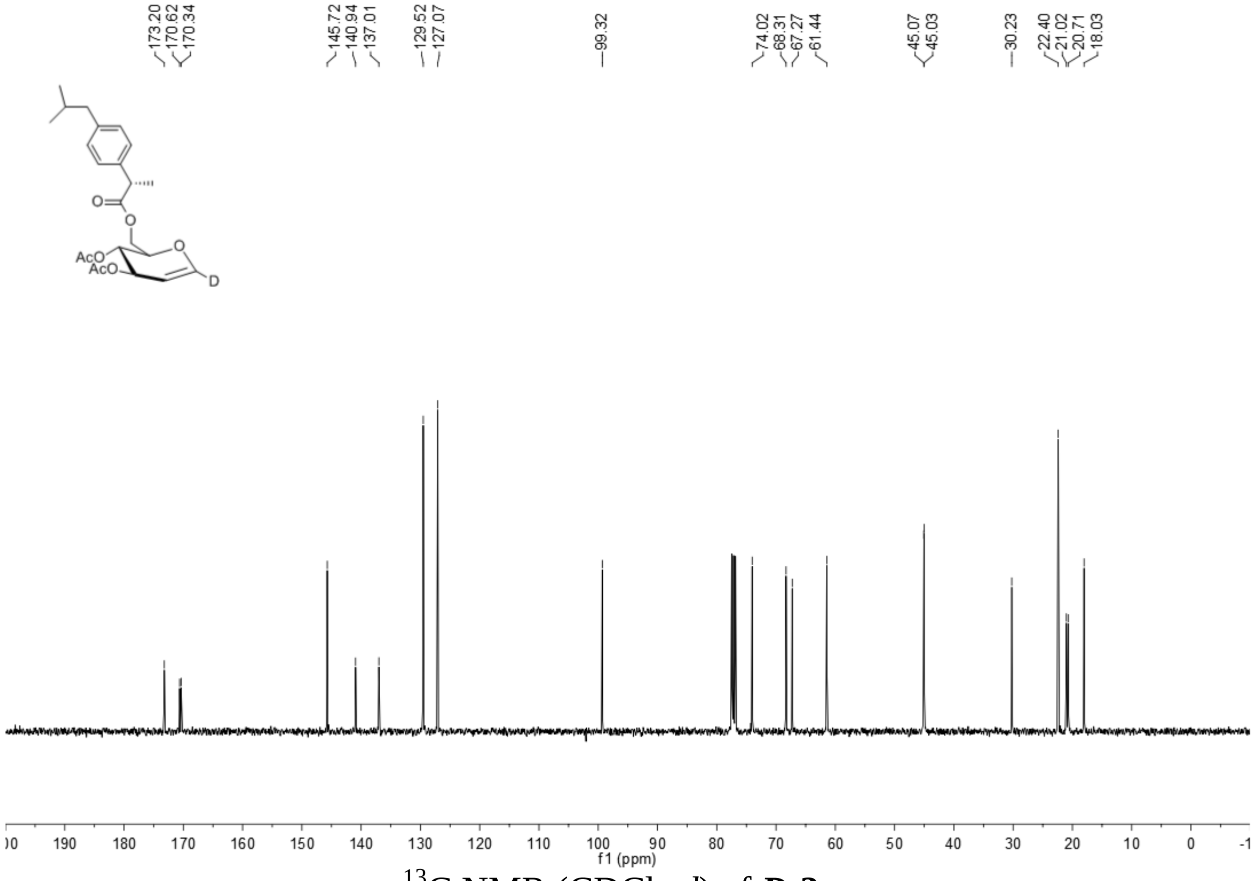

^{13}C NMR (CDCl$_3$-*d*) of ***D*-3ac**

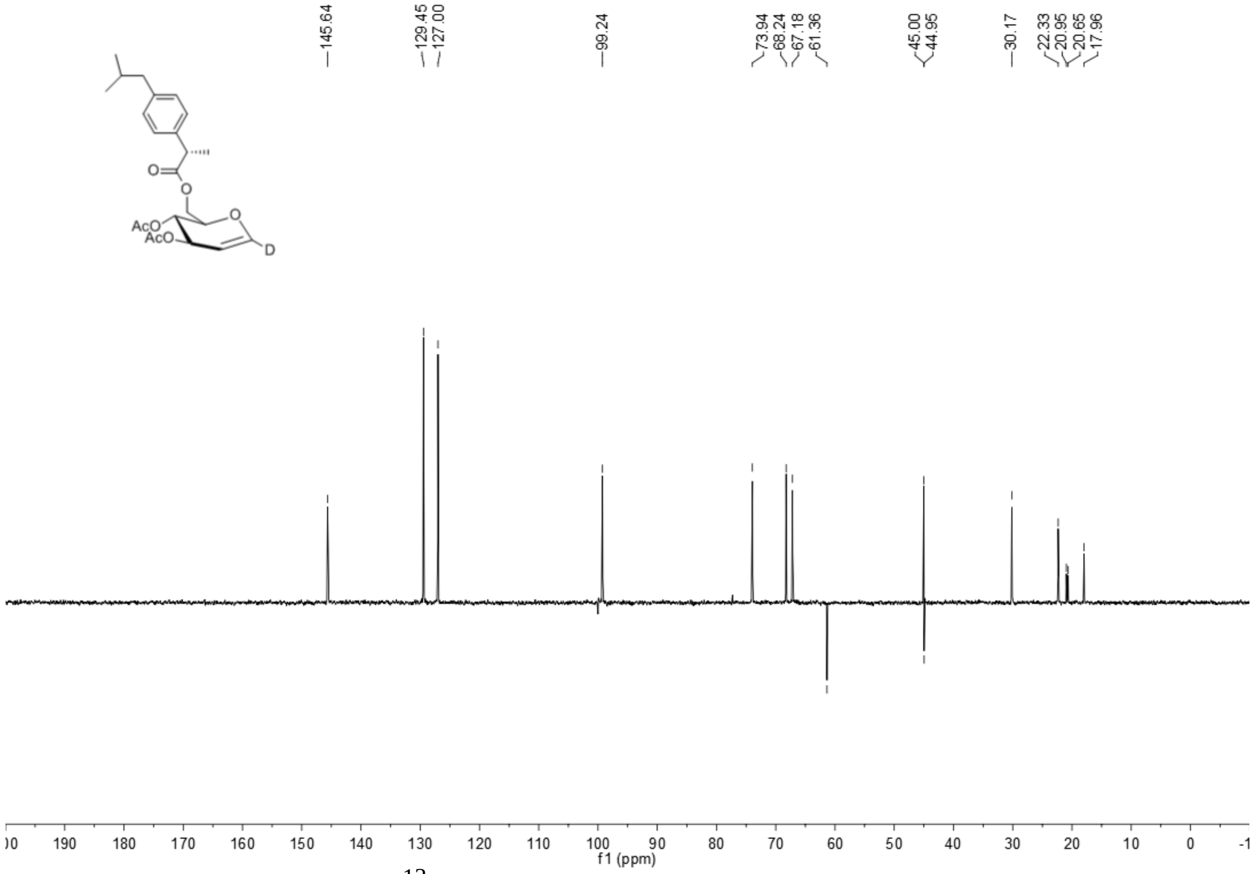

^{13}C NMR ($CDCl_3$-*d*) of ***D*-3ac**

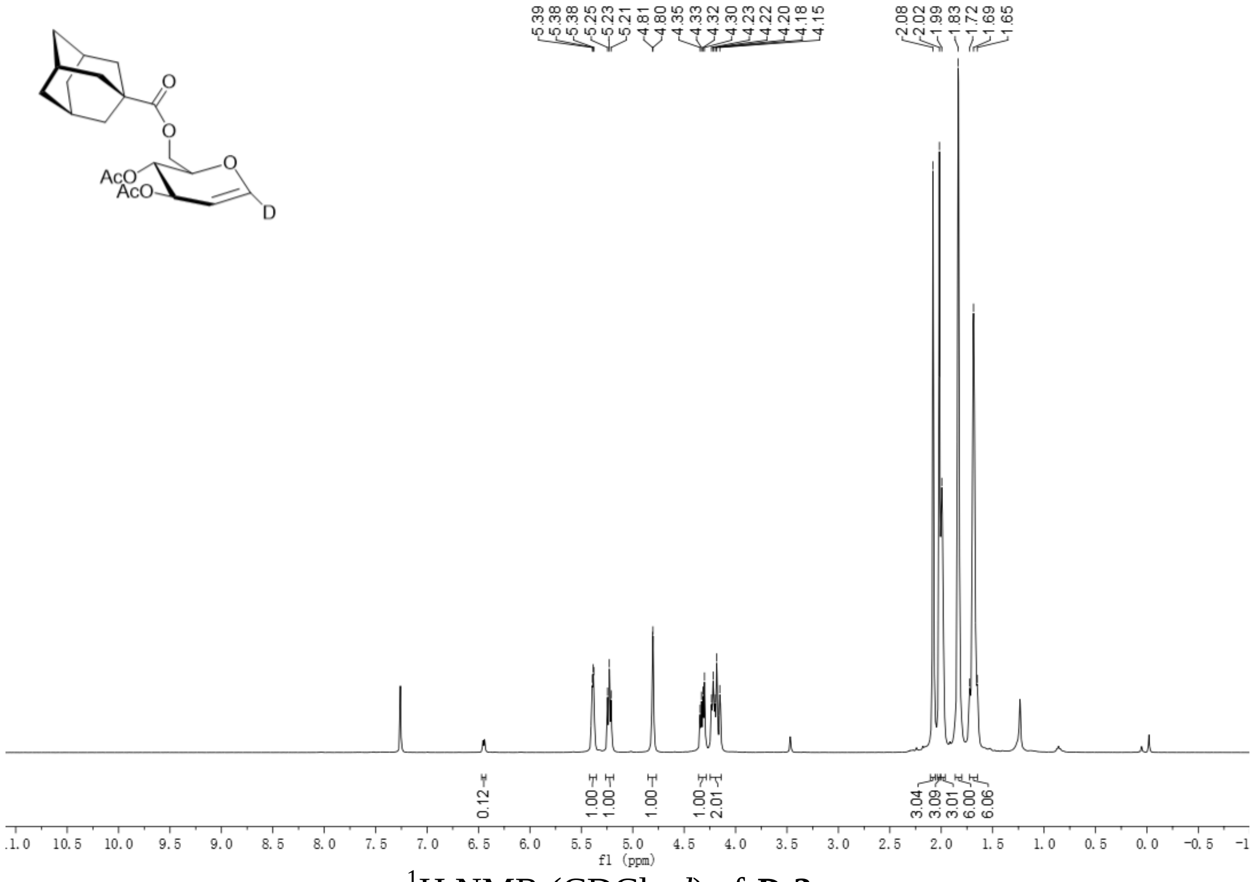

^{1}H NMR ($CDCl_3$-*d*) of ***D*-3aa**

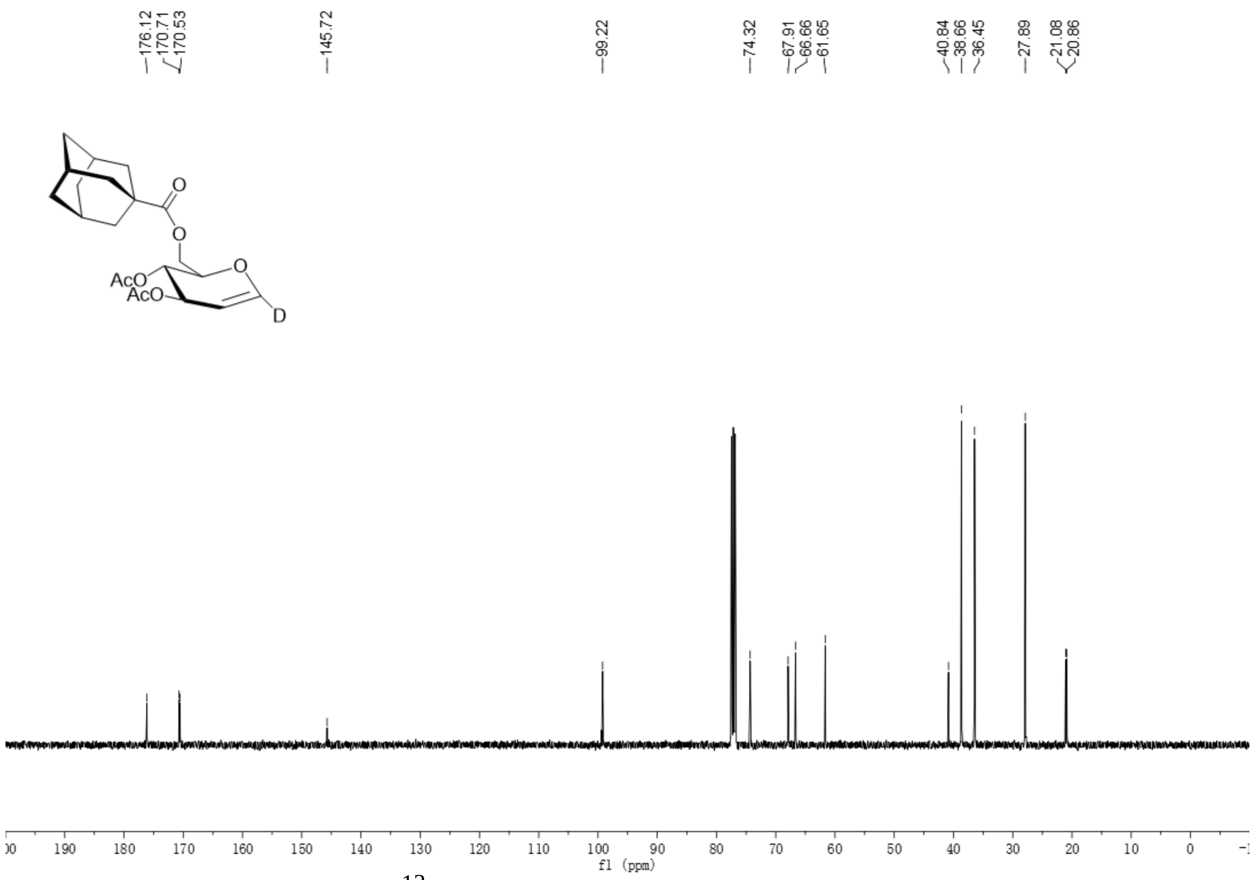

^{13}C NMR ($CDCl_3$-*d*) of ***D*-3aa**

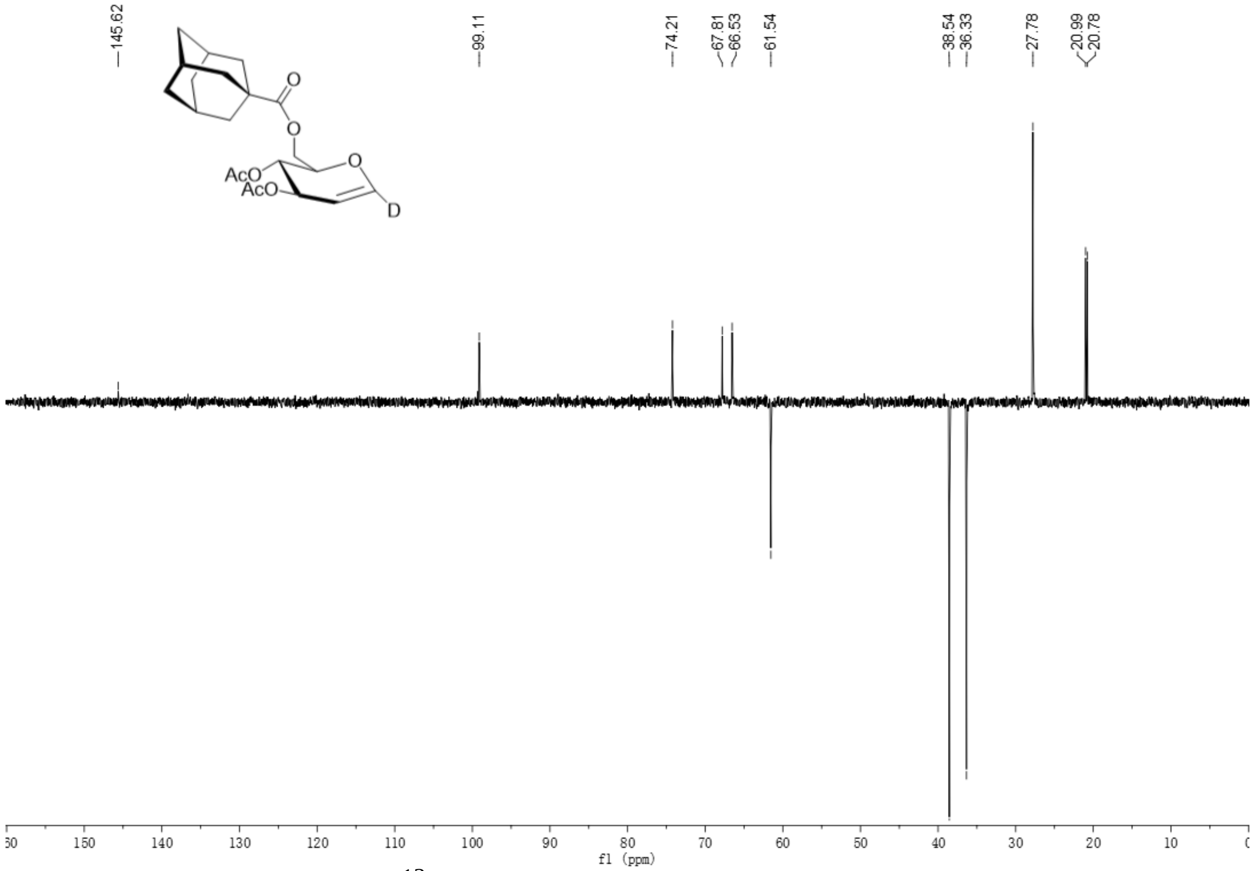

^{13}C NMR ($CDCl_3$-*d*) of ***D*-3aa**
